# On the Scope of DCAF1-Recruiting PROTACs Degrading Protein Kinases

**DOI:** 10.1021/acs.jmedchem.6c00383

**Published:** 2026-06-17

**Authors:** Janik Weckesser, Nebojša Miletić, Saran Aswathaman Sivashanmugam, Uli Ohmayer, Martin Steger, Bachuki Shashikadze, Paul Gehrtz, Andrea Unzue Lopez, Ansgar Wegener, Timo Yoshua Dietz, Tobias Hammann, Ingo V. Hartung, Lewis Elson, Václav Němec, Martin Peter Schwalm, Bikash Adhikari, Elmar Wolf, Andreas Krämer, Susanne Müller, Henrik Daub, Stefan Knapp

**Affiliations:** † Institute for Pharmaceutical Chemistry, Department of Biochemistry, Chemistry and Pharmacy, 9173 Goethe University Frankfurt, Max-von-Laue-Straße 9, Frankfurt 60438, Germany; ‡ Structural Genomics Consortium, Buchmann Institute for Molecular Life Sciences, Goethe University Frankfurt, Max-von-Laue-Straße 15, Frankfurt 60438, Germany; § German Cancer Consortium (DKTK), partner site, Frankfurt/Mainz 60438, Germany; ∥ FCI Frankfurt Cancer Center (FCI), Georg-Speyerhaus, Frankfurt am Main 60438, Germany; ⊥ NEOsphere Biotechnologies GmbH, Fraunhofer Str. 1, Martinsried 82152, Germany; # 2792 Merck Healthcare KGaA, Darmstadt 64293, Germany; ∇ Institute of Biochemistry, University of Kiel, Kiel 24118, Germany

## Abstract

Proteolysis-targeting chimeras (PROTACs) have emerged as a novel drug modality, but their development currently relies on a limited number of E3 ligase ligands, primarily targeting CRBN and VHL. Conversely, current validation studies on novel E3 ligase ligands are typically limited to a few highly degradable targets, like BRD4. Here, we used our previously established workflow for E3 ligase ligand validation, employing promiscuous kinase PROTACs, to evaluate the potential of recruiting DCAF1 for PROTAC development. Our study revealed the DCAF1-dependent degradation of a diverse set of kinases, which were validated in orthogonal assays. In a comparative analysis, we identified a significant overlap between the degradable kinome of DCAF1- versus CRBN-recruiting PROTACs, suggesting alternative design strategies for PROTACs using available structurally diverse DCAF1 ligands. Moreover, ubiquitinomics analysis of the PROTAC-induced ubiquitination patterns provided insight into substrate- and isoform-selective degradation. The presented data will establish DCAF1-recruiting PROTACs as a versatile design strategy for future degrader development.

## Graphical abstract

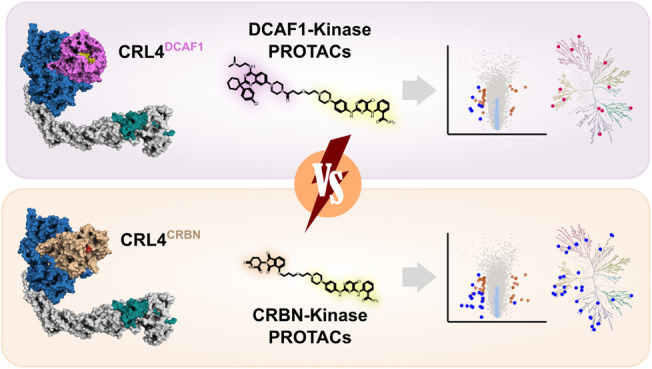

## Introduction

Proteolysis Targeting Chimeras (PROTACs) are heterobifunctional molecules that catalyze the ubiquitination and subsequent degradation of proteins of interest (POIs) by harnessing the ubiquitin-proteasome system (UPS). PROTACs have become a powerful targeting strategy in both drug discovery and the development of tool compounds enabling selective target degradation to study biological processes at the cellular level, including organoids, tissues, and *in vivo*.^1–4^ PROTACs have a tripartite structure consisting of a POI ligand and an E3 ligase ligand that are connected by a chemical linker. However, the development of potent PROTACs typically requires extensive and often empirical optimization of linkers, often referred to as “linkerology”, posing a challenge to medicinal chemists.^5,6^ Stable ternary complexes have been described as a key feature for successful degradation.^7–9^ The stability of ternary complexes is mainly determined by the linker, the linker attachment point, optimal POI and E3 ligase orientation mediated by the PROTAC as well as compatible protein surfaces interacting at the POI/E3 ligase interface resulting in synergistic assembly. Currently, PROTACs with few exceptions employ only two E3 ligases, Cereblon (CRBN) and the von Hippel-Lindau (VHL) E3 ligase. However, the efficacy of degrading specific POIs depends on the E3 ligase selected for PROTAC design.^10,11^ Therefore, the degradable fraction of the proteome may largely be determined by the targeted E3 ligase, suggesting that some POIs may not be efficiently degraded or may remain completely inaccessible for degrader development using the current repository of validated E3 ligase ligands. In addition to increasing the degradable proteome, the cell- and tissue-type restricted expression profiles of novel E3 ligases could enable the development of spatially controllable tissue-specific PROTACs for precision medicine. Some cancers require certain E3 ligases for sustaining cancer cell growth or cancer progression. Targeting these E3 ligases for PROTAC development may reduce the risk of developing resistances due to genetic silencing, as already observed in instances of prolonged PROTAC treatment.^12–14^ Consequently, ligands have been developed for several E3 ligases, thereby expanding the toolbox for PROTAC development.^15^ However, the utility and scope of these ligands for developing PROTACs that degrade certain target families has generally not been assessed beyond a few highly degradable proteins, such as bromodomain-containing protein 4 (BRD4).

Hence, novel E3 ligase ligands have only rarely found broader application beyond the initial POIs they have been validated for.^16^ In a recent landmark paper, Donovan and Ferguson et al. employed promiscuous kinase inhibitors to map the “degradable” kinome accessible through CRBN-ligand-based PROTACs,^17^ which has been complemented by our workflow (Figure 1a) comprising target engagement assays, toxicity evaluation and proteomics for the evaluation of the now well-established VHL ligands across the kinome in a proof-of-principle study.^18^

**Figure 1:**
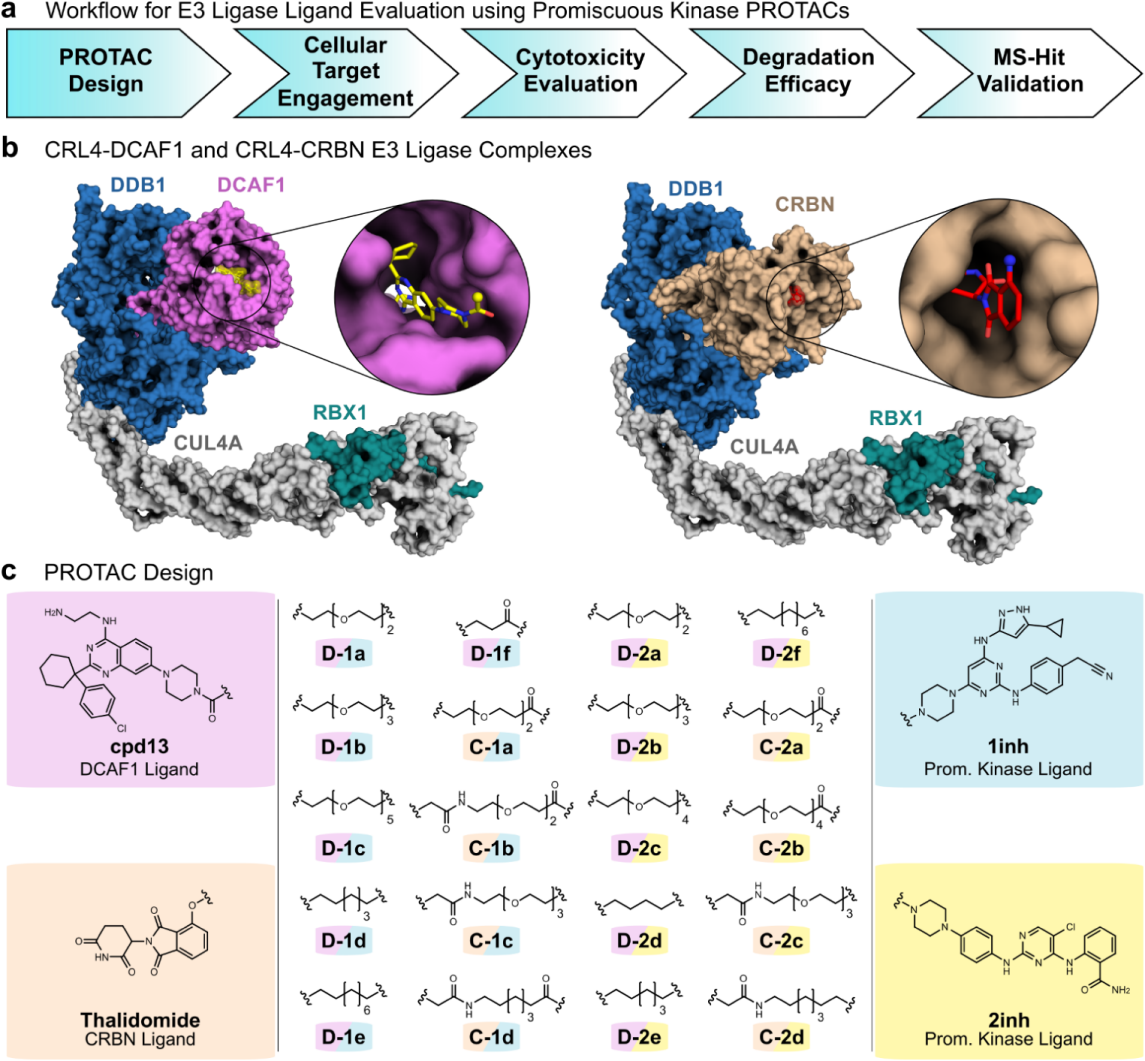
Architecture of the DCAF1 and CRBN E3/E2 ligand complexes and evaluation and design strategy for both PROTAC series. (a) Workflow for the evaluation of E3 ligase ligands. (b) Illustration of the CRL4DCAF1 (left) and CRL4CRBN (right) E3 ligase complexes as well as the binding modes of cpd13 and the thalidomide derivative pomalidomide (figures based on PDB-ID: 7OKQ, 8OO5, 4CI3).^29–31^ Individual subunits of the multiprotein complexes are colored as follows: RBX1 (teal), CUL4A (gray), DDB1 (blue), DCAF1 (pink), and CRBN (beige). The linker attachment point for each ligand is shown as a sphere in each close-up. (c) Overview of the chemical structures for all synthesized DCAF1- and CRBN-recruiting promiscuous kinase PROTACs. The syntheses are shown in Figures S40–45.

Inspired by recent publications of DCAF1 ligands,^19–22^ we explored the degradable kinome with DCAF1-based PROTACs. DCAF1 associates with Cullin 4 (CUL4), the adaptor protein DDB1 and the E2-binding protein RBX, similar to the one assembled within the CRBN ligase complex (Figure 1b). However, in contrast to CRBN, DCAF1 harbors a structurally diverse E3 substrate recruitment domain of the WDR family. Moreover, contrary to CRBN, DCAF1 is also essential to a wide array of cancer cells.^23,24^ Target-specific PROTACs have been developed harnessing DCAF1 for the degradation of BRD9, WDR5 and the kinases BTK and LIMK1.^25,26^ Some of these targets, such as WDR5, have only been moderately degraded as shown by their relatively low maximum percentage of degradation values (*D*_max_; endogenous WDR5:23–49%^25^) and the broader applicability of utilizing DCAF1 for PROTAC development therefore remains unclear. In the present study, we used the DCAF1 ligand **cpd13** with the highly promiscuous kinase ligands **1inh** and **2inh** (Figure 1c) for the synthesis of a series of promiscuous kinase PROTACs to shed light on the degradable kinome targeted by these degrader systems.^18^ We analyzed the degradable target spaces of the DCAF1-recruiting PROTACs using quantitative proteomics as well as ubiquitinomics and compared the degradable kinomes of matched sets of DCAF1- and CRBN-recruiting PROTACs. We selected CRBN as a benchmark because of its well-documented ability to degrade a wide range of kinases.^17^ Our analysis revealed a significant overlap in the target space of degraded proteins, suggesting that DCAF1 ligands could serve as a versatile alternative to the well-established CRBN ligand systems. The diversity of reported DCAF1 ligands and the dependence of cancer cell survival and proliferation on DCAF1 make this E3 ligase an attractive alternative for the development of next-generation PROTACs.^27,28^

## Results and Discussion

The reported structure of the DCAF1-DDB1-CUL4-RBX1 complex (PDB-ID: 7OKQ)^29^ and a model of the DDB1-CRBN complex (PDB-ID: 4CI3)^30^ provided structural insights into the E3/E2 ligase architecture of the two E3 ligases compared in our study. As expected, superimposing the structural models revealed conserved subunit arrangements and similar positions of the two diverse E3-substrate binding domains (Figure 1b). We designed a series of PROTACs with two broad spectrum kinase ligands for each of the two E3 ligases and profiled these degraders using our previously established workflow for E3 ligase ligand evaluation.^18^ In addition to rational degrader design, this workflow comprised target engagement assays, evaluation of cellular toxicity, mass-spectrometry (MS)-based proteomic analyses, and detailed mechanistic validation of the identified active PROTACs (Figure 1a).

Following the general tripartite structure of PROTACs, either **cpd13** or 4-hydroxy thalidomide was coupled to one of two selected broad-spectrum kinase inhibitors, **1inh** or **2inh**, by employing various PEG and alkyl linkers of different lengths (Figure 1c). For the development of DCAF1 PROTACs, linker moieties were installed at the piperazine nitrogen of **cpd13** exclusively via amide-based chemistry. The amide moiety has been reported to contribute to DCAF1 binding affinity and the used linker attachment strategy has already been validated in a recent publication.^22,26^ For the CRBN-recruiting PROTACs, 4-hydroxy thalidomide was either coupled directly or via an inserted acetate moiety to the respective linkers.^17^ Analogous to the DCAF1-recruiting PROTACs, the resulting 4-hydroxy thalidomide linker conjugates were coupled to either **1inh** or **2inh** via the tertiary amine exit vector. While focusing mostly on highly flexible linkers to probe the most suitable linker length, we also hoped that the flexibility of these moieties would maximize the number of kinases that were degraded. A proportion of the conjugates were coupled to the kinase inhibitors through amide attachment chemistry. Structural rigidity has been hypothesized to facilitate the formation of particularly stable ternary complexes. Thus, the inclusion of more rigid linkers would be advisable for the development of target-selective PROTACs. Collectively, a series of 20 structurally diverse promiscuous kinase PROTACs (syntheses are shown in Figures S40-45) was generated, covering ligands binding to DCAF1 and CRBN, respectively.

### DCAF1-Recruiting PROTACs Engaged with Kinases in Live Cells

The synthesized series of PROTACs and the respective kinase parent inhibitors, **1inh** and **2inh**, were subsequently profiled in cellular target engagement experiments. To this end, the compounds, together with positive controls, the CRBN-recruiting **JB300** (AURKA)^32^ and the VHL-recruiting **BI-0319** (PTK2),^33^ were profiled using NanoBRET cellular target engagement assays^3,34,35^ against two representative kinase targets, AURKA and PTK2. We compared NanoBRET data in live and permeabilized cells, which allowed us to assess membrane penetration of the PROTACs as indicated by the EC_50_ (perm.)/EC_50_ (int.) ratios (Figure 2a,c, Figure S1-3, Table S1). The parent ligands, **1inh** and **2inh**, differed in cellular target affinity (AURKA/PTK2 [μM]: 0.51 ± 0.02/1.07 ± 0.42 and 0.03 ± 0.01/0.04 ± 0.01, respectively), allowing us to investigate target engagement within a range of binding potencies. As expected, all PROTACs showed significantly weaker target engagement in intact cells compared to the corresponding parent inhibitors, **1inh** and **2inh**. This finding was at least partially attributed to less favorable cell permeability, as indicated by the EC_50_ ratios of live and permeabilized cells. In addition, PROTACs derived from **2inh** showed generally stronger engagement with both kinases, in both intact and permeabilized cells. Some PROTACs even surpassed the positive controls **JB300** and **BI-0319**. Interestingly, the EC_50_ ratios indicated a generally better cell penetration of the **2inh**-series over the series derived from **1inh**. It is noteworthy that the CRBN-recruiting PROTACs derived from **2inh** exhibited generally poorer cell penetration compared to the DCAF1-recruiting PROTACs. Nevertheless, the majority of the PROTACs interacted tightly with both of the chosen representative kinases in cellulo, with the exception of PROTACs **D-1d-f** and **C-1d**, which all contained alkyl linkers. Poor solubility was identified as the main reason for the particularly weak target engagement of PROTAC **D-1f**, as this compound was found to precipitate in the assay, thus rendering it unsuitable for further cellular investigations.

**Figure 2:**
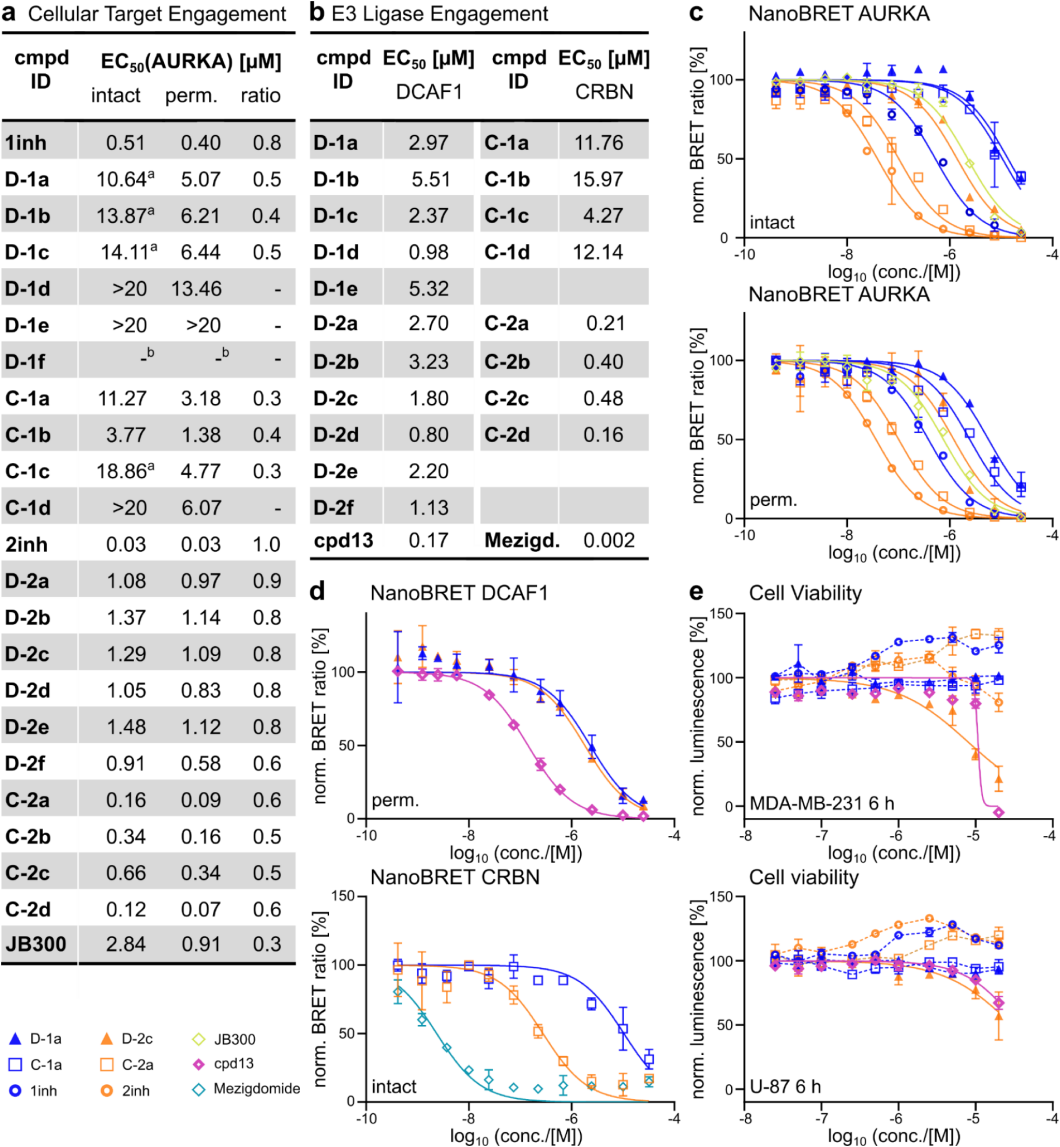
Cellular POI engagement, E3 ligase engagement and effects on cell viability. (a) All PROTACs, parent ligands and the positive control JB300 were tested against AURKA in intact and digitonin-treated cells in the NanoBRET target engagement assay. EC50 values were calculated from biological replicates (*n* = 4). Standard deviations are given in Table S1. (b) NanoBRET target engagement assay against DCAF1 and CRBN. EC50 values were calculated from biological replicates (*n* = 4). Standard deviations are given in Table S2. Cpd13 and Mezigdomide were utilized as positive controls. (c) Representative NanoBRET dose–response curves of selected PROTACs and controls. Displayed results represent the mean of technical replicates (*n* = 2), the error bars indicate the standard deviation. Dose-response curves for all experiments including the second biological replicate are shown in Figure S1–3. (d) Representative E3 ligase NanoBRET dose–response curves for selected PROTACs and controls. Shown data represent the mean of technical replicates (*n* = 4), error bars indicate the standard deviation. Dose-response curves for all experiments including the second biological replicate are shown in Figure S4–5. (e) Representative cell viability (CellTiter-GLO) dose-response data for selected PROTACs. Displayed results represent the mean of technical replicates (*n* = 4) and error bars indicate the standard deviation. Dose-response curves for all experiments including the second biological replicate are shown in Figure S7–9. Calculated IC_50_ values are shown in Table S3. Data points that do not follow a sigmoidal curve shape are indicated by dotted lines. ^a^Estimated EC_50_ values based on extrapolation. ^b^D-1f precipitated during the assay (data were not evaluated).

The majority of the DCAF1-recruiting PROTACs were found to engage with the E3 ligase with low single-digit micromolar affinities in permeabilized cells, with the exception of PROTACs **D-1a** and **D-1f** (Figure 2b,d, Figure S4, Table S2). However, no significant DCAF1 binding was detected for any of the PROTACs in intact cells (data not shown). Interestingly, this behavior has also been observed in our previously published study for VHL-recruiting PROTACs that still catalyzed POI degradation, suggesting that partial E3 ligase occupancy is sufficient for degradation in live cells. Likewise, the CRBN-recruiting PROTACs generally exhibited potent engagement with CRBN in permeabilized cells. Here, E3 ligase engagement was significantly more potent when compared to the respective DCAF1 PROTACs (Figure 2b,d; Figure S5, Table S2). Thus, the DCAF1 ligand could still be further optimized for cellular activity. In summary, with few exceptions, the developed PROTACs of the two targeted E3 ligase systems effectively formed binary complexes with POIs as well as E3 ligases.

Erroneous conclusions about degrader efficacy are sometimes drawn from cellular studies conducted under conditions that influence cellular viability, because of the difficulty to distinguish between cell viability- and PROTAC-induced protein degradation.^34,36^ Therefore, all PROTACs as well as the parent kinase inhibitors, **1inh** and **2inh**, were evaluated for their effect on cell viability in CellTiter-GLO assay on the two cell lines used, the breast cancer cell line MDA-MB-231 and the glioblastoma cell line U-87 after 6 and 24 h, respectively (Figure 2e, Figure S7–8, Table S3). The parent inhibitors **1inh** and **2inh** did not affect cell viability in any of the cell lines after 6 h. No effect on viability was observed for **1inh** or the corresponding DCAF1-based PROTACs after 6 h. DCAF1-based PROTACs derived from **2inh** showed a significant effect at elevated concentrations, presumably due to the broad-spectrum kinase activity, with the exception of PROTAC **D-2e** and **D-2f**. However, the majority of both DCAF1-recruiting PROTAC series was found to affect cell viability at high PROTAC concentrations after 24 h, presumably indicating a broad on-target activity, again particularly pronounced for the **2inh**-derived PROTACs. Intriguingly, the CRBN-recruiting PROTACs exerted a generally lower cell toxicity than their DCAF1-recruiting counterparts. To elucidate whether the observed toxicity of the DCAF1-recruiting PROTACs was at least partially attributable to DCAF1 inhibition, **cpd13** was also evaluated in both cell lines (Figure 2e, Figure S9, Table S3). However, **cpd13** only affected cell viability at the highest concentration tested at both time points. Based on these data, a 1 μM concentration and a 6-h treatment period were determined as suitable experimental conditions for the subsequent proteomic experiments, avoiding potential unspecific toxicity-induced contributions to protein degradation.

### PROTACs Induce DCAF1-Dependent Degradation of Multiple Kinases

The synthesized PROTACs were subsequently profiled in quantitative MS-based proteomic experiments to investigate the degraded target space (degradome). To assess the impact of cell line-restricted differences like kinase and E3 ligase expression levels on degradation efficacy, the proteomic experiments were conducted in MDA-MB-231 (breast carcinoma-derived) and U-87 (glioblastoma-derived) cells. Treatment with the parent inhibitors **1inh** and **2inh** did not cause kinase degradation under the used treatment conditions as shown in our previously reported study.^18^

We found that the designed PROTACs exhibited broad activity across the kinome in MDA-MB-231 cells. As expected, PROTAC activity was highly dependent on linker moieties with the most active PROTACs inducing the degradation of ten or more kinases whereas no activity was detected for **D-2f** (Figure 3a, Figure S10-12, Table S4). Additionally, no correlation was observed between degradation efficacy and linker composition (PEG vs alkyl). Consistent with our previous study, the PROTACs derived from kinase parent inhibitor **2inh** showed a broader degradation efficacy compared to the **1inh**-derived degraders. This difference in activity was presumably due to the broader target engagement of **2inh** and its improved cell penetrance, as indicated by the more favorable NanoBRET ratios. However, surprisingly, the most active PROTAC, **D-1c**, was derived from kinase parent inhibitor **1inh**, inducing the degradation of 34 kinases. In addition to kinases, a proportion of the PROTACs also degraded a few other nonkinase proteins, of which many were known kinase interactors, such as INCENP,^37^ which is a regulator and interactor of AURKA, -B and −C, or cyclins such as CCNE2, which tightly bind to CDK family members and have been reported to be degraded by PROTACs targeting cyclin-dependent kinases.^38,39^

**Figure 3:**
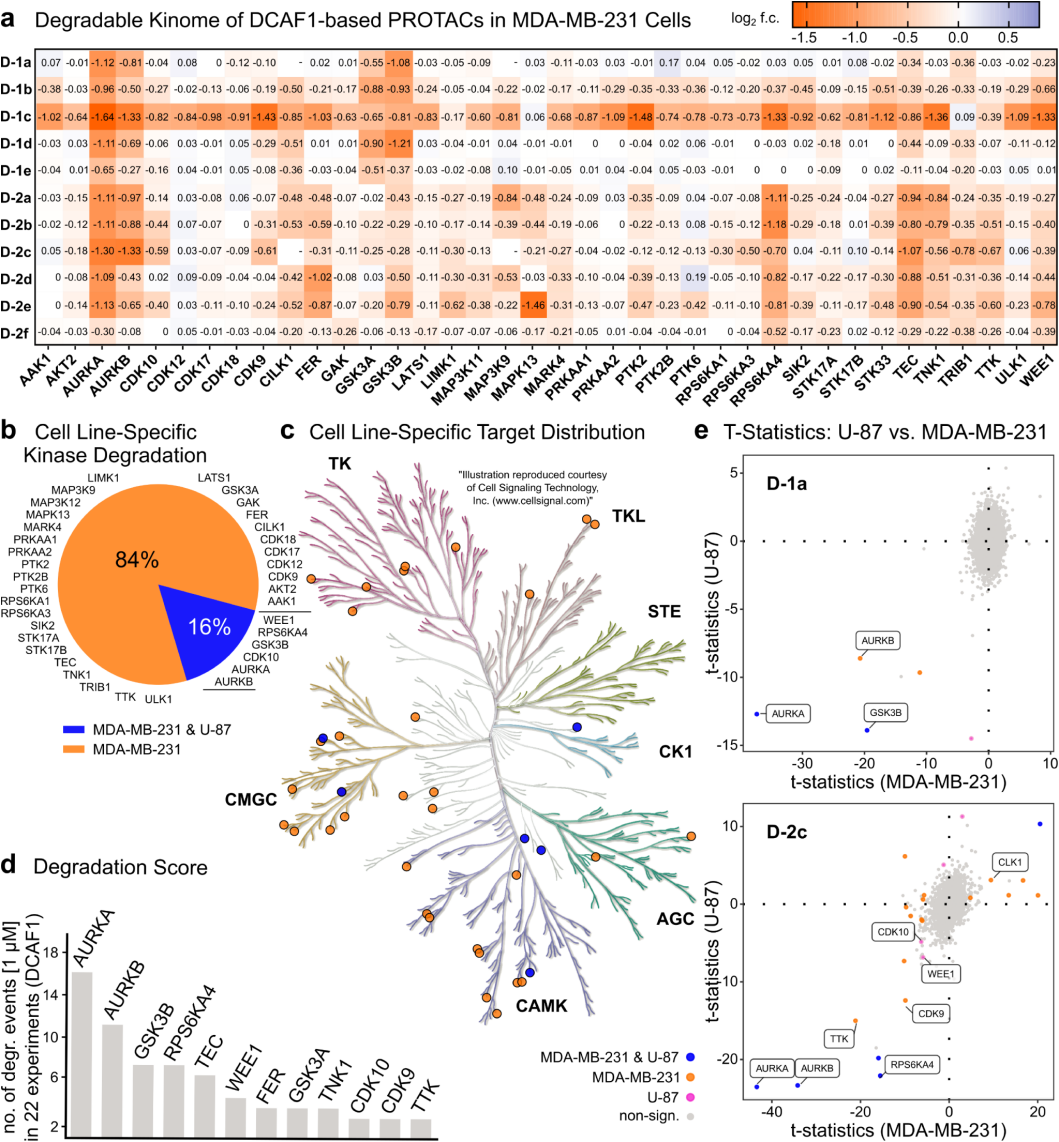
DCAF1-mediated kinase degradation is cell line-specific. (a) Log_2_ fold changes (f.c.) of all kinases hit by at least one DCAF1-based PROTAC in MDA-MB-231 cells with a moderated adjusted p-value <0.01 and log_2_ fold change ≤ −0.6 (b) Cell line-specific degradome of the DCAF1-recruiting PROTACs. (c) Distribution of degraded kinases over the kinome depending on the cell line.^40^ (d) Number of observed degradation events for each kinase in a total of 22 independent proteomics experiments with DCAF1-recruiting PROTACs. Displayed kinases were degraded in at least two experiments. (e) T-statistic comparison of the degraded kinome in U-87 and MDA-MB-231 cells for D-1a and D-2c.

Proteomics data measured in U-87 cells revealed striking differences in degradation efficacy of the PROTACs as compared to the MDA-MB-231 cells (Figure S13-15, Table S4). In general, the U-87 cells were less responsive to treatments with the DCAF1-recruiting PROTACs, with only six PROTACs, **D-1a**, **D-1b**, **D-1d**, **D-2a**, **D-2b,** and **D-2c**, inducing significant kinase degradation at 1 μM PROTAC concentration. This finding correlated with the lower expression levels of DCAF1 in U-87 cells, as determined by proteomic measurements of DMSO-treated samples of both cell lines (log_2_ fold change DCAF1 (U-87/MDA-MB-231) = −1.34). Essentially, 16% of the kinases degraded by at least one DCAF1-recruiting PROTAC were degraded in both cell lines. Meanwhile, 84% were exclusively degraded in MDA-MB-231 cells, and no kinase target was exclusively degraded in U-87 cells. (Figure 3b). While kinases across several kinase subfamilies were degraded in both cell lines by multiple PROTACs, AURKA and AURKB were the most frequently and most potently degraded kinases (Figure 3c,d).

The differences in kinase expression and degradation efficacy for individual kinases in both cell lines did not correlate. This observation is highlighted by the representative analysis of PROTACs **D-1a** and **D-2c** (Figure 3e, Table S5). Furthermore, the degradation efficacy in both cell lines was remarkably different for some of the PROTACs. For instance, PROTAC **D-1c**, which degraded the most kinases in MDA-MB-231 cells, was inactive in U-87 cells. Degradation efficacies of the PROTACs also did not correlate with cellular target engagement. Moreover, PROTAC **D-1c** was the most potent AURKA degrader, yet it had the weakest affinity for this target among the series of PROTACs (Figure 2a). These comparisons highlight once again that the multilayered degradation mechanism of PROTACs goes far beyond the initial binary binding affinity for the POI and the E3 ligase.^8,41,42^

Encouraged by the proteomic results, we selected PROTACs **D-1a** and **D-2c** as representative PROTACs for further investigation and designed negative controls that no longer bound to DCAF1. In the original report by Schröder and Renatus et al., the primary amine of **cpd13** was dimethylated to disrupt binding to DCAF1.^26^ However, we found that the dimethylated ligand **cpd13-N** had still considerable affinity for DCAF1 (EC_50_ = 3.65 ± 0.35 μM). Based on the published crystal structure of **cpd13** in complex with DCAF1 (PDB-ID: 8OO5),^31^ we assumed that the replacement of the dimethyl moiety with a sterically more demanding *tert*-butoxycarbonyl (Boc) group would result in more efficient disruption of DCAF1 binding. We therefore synthesized **cpd13** ^**n.c.**^ and tested it in our NanoBRET assay (Figure S4, Table S2). As expected, **cpd13** ^**n.c.**^ did no longer bind to DCAF1 and was used to synthesize two negative control PROTACs, **D-1a** ^**n.c.**^ and **D-2c** ^**n.c.**^, which were profiled using the same workflow (Figure 4a, Figure S4,S6and S9, Table S1-3). PROTAC **D-1a** strongly degraded the kinases AURKA and AURKB as well as GSK3B but not the closely related isoform GSK3A. The PROTAC **D-2c** had a broader activity, degrading AURKA and AURKB, the Ribosomal Protein S6 Kinase A4 (RPS6KA4), the cyclin dependent kinase 9 (CDK9), the tyrosine kinase TEC, the threonine and tyrosine kinase (TTK) and remarkably the pseudokinase TRIB1 (Tribbles Pseudokinase 1). However, the related pseudokinase TRIB3 was upregulated (Figure 4b). Gratifyingly, neither of the matching negative control PROTACs induced the degradation of kinases (Figure 4c). Likewise, DCAF1 ligand **cpd13** and control compound **cpd13** ^**n.c.**^ did not affect proteome-wide kinase levels (Figure S16-17), which collectively confirmed DCAF1-dependent degradation of kinases by degraders **D-1a** and **D-2c**.

**Figure 4:**
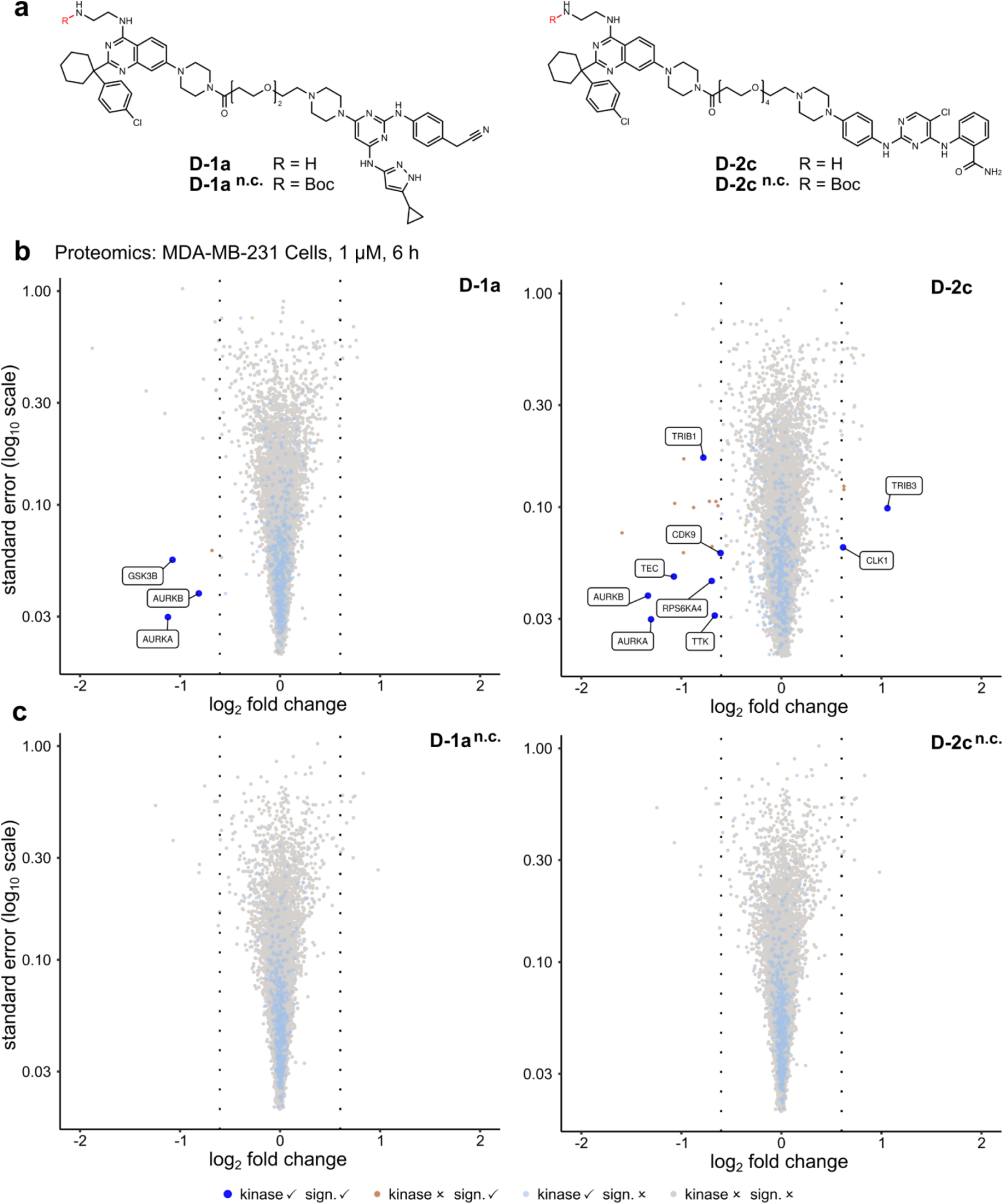
DCAF1-recruiting PROTACs D-1a and D-2c are active kinase degraders. (a) Chemical structure of D-1a, D-2c, and the corresponding negative controls D-1a^n.c^. and D-2c^n.c^. MDA-MB-231 cells were treated with 1 μM of either D-1a or D-2c for 6 h (b) or the corresponding negative controls D-1a^n.c^. and D-2cn.c. (c), respectively. Significantly up- or downregulated protein kinases are shown with blue dots and are labeled whereas nonkinase proteins are highlighted with orange dots (moderated adjusted p-value <0.01; dotted lines: log_2_ fold change ≤ −0.6 or ≥ + 0.6). Levels of proteins that were not significantly changed are shown in light blue (kinases) and gray (nonkinase proteins). The results shown are the mean of biological replicates (*n* = 3). The complete data set for all PROTACs can be found in supplementary file 2.

We next profiled PROTACs **D-1a** and **D-2c** at different concentrations using MS-based proteomics in both cell lines. Both PROTACs were profiled at a lower concentration of 0.5 μM to assess if potentially degraded kinases were masked by the hook effect at the screening concentration of 1 μM. In addition, we also tested the PROTACs at higher concentration (5 μM) to identify degraded kinase targets with high DC_50_ values, potentially expanding the degradable kinome accessible through optimized DCAF1 ligands. Lowering the PROTAC concentration to 0.5 μM resulted only in a reduced number of degraded kinases for both PROTACs. This effect was particularly pronounced for the PROTAC **D-2c** in MDA-MB-231 cells, in which only three kinases were significantly degraded compared to seven significantly degraded kinases at 1 μM (Figure S18-19). These data suggested that no targets were missed due to the hook effect. Conversely, a significantly larger number of kinases were degraded in both cell lines when cells were treated at a concentration of 5 μM. This increase was particularly evident in U-87 cells where only two kinases (AURKA and B) were degraded at 0.5 μM, whereas nine kinases and the AURK regulator INCENP were detected at 5 μM for PROTAC **D-2c**. The degraded kinases largely overlapped at higher PROTAC concentration in both cell lines. A dose-dependent increase in degradation efficacy with increasing PROTAC concentration was observed for kinases like AURKA, AURKB, CDK9, GSK3A, and PTK2, allowing the estimation of DC_50_ values from proteomics data (Figure 5a, Figure S20). The combined data suggested that a similar target spectrum was degraded at different PROTAC concentrations in both cell lines. Kinase targets that were only detected at high PROTAC concentrations may be efficiently degraded after further optimization of the degraders using more selective kinase ligands. However, we did not study the developed PROTACs at higher concentrations than 5 μM due to potential complications arising from PROTAC-induced effects on cellular viability and PROTAC solubility.

**Figure 5:**
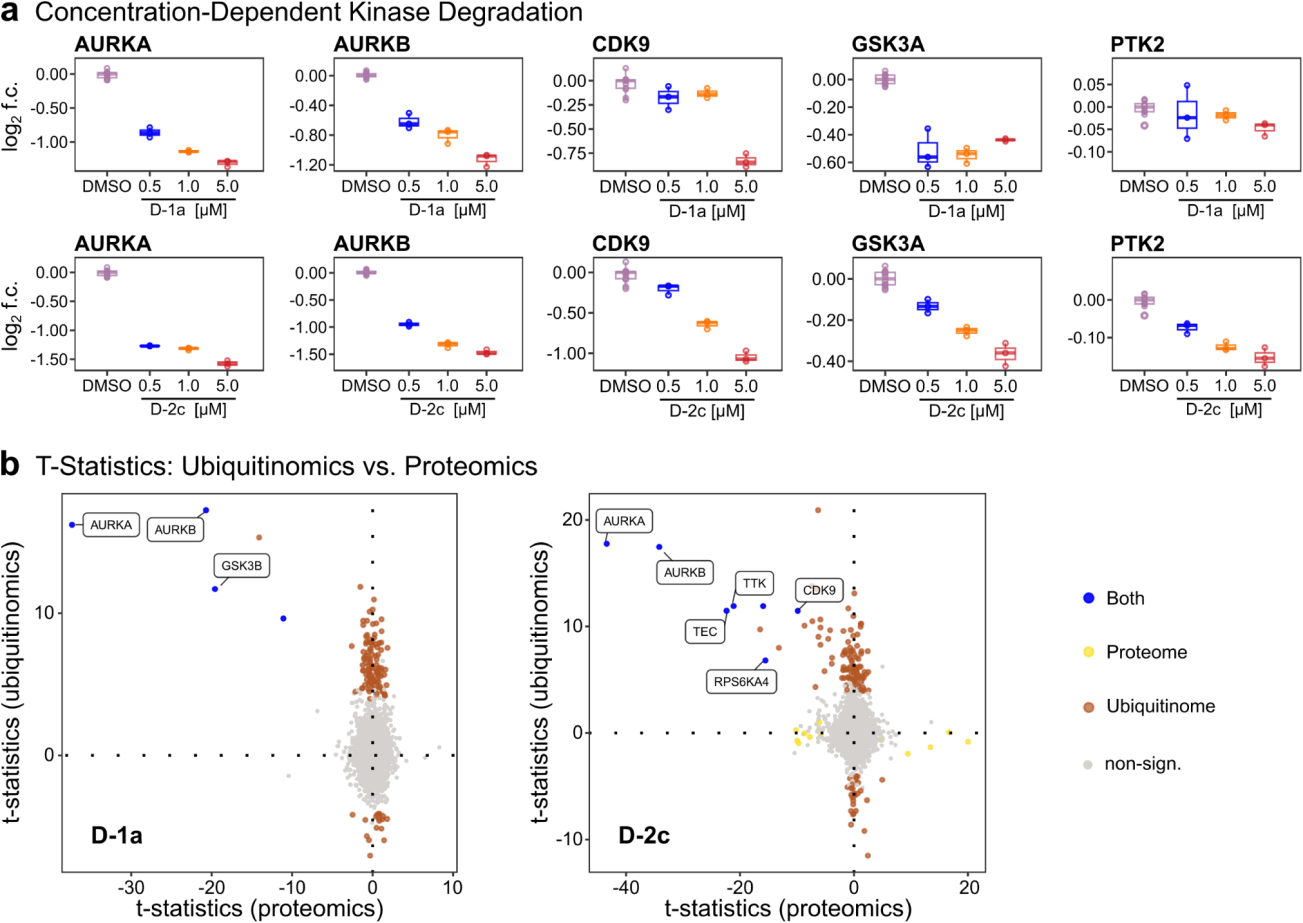
DCAF1-based PROTACs D-1a and D-2c induced dose-dependent kinase degradation and ubiquitination. (a) Dose-dependent log_2_ fold changes (proteomics) for selected kinases induced by PROTACs D-1a and D-2c in MDA-MB-231. Results for U-87 cells are given in Figure S20. (b) T-statistic comparison of the altered ubiquitinomes and proteomes for D-1a and D-2c. The ubiquitinomics data is available in supplementary file 2.

PROTACs **D-1a** and **D-2c** and the mostly inactive PROTACs **D-1e** and **D-2f** were subsequently studied using quantitative ubiquitin-diGLY proteomics experiments (ubiquitinomics) to investigate proteome-wide ubiquitination events. We were particularly keen on correlating the observed kinase degradation with kinase ubiquitination as well as elucidating potential correlations between the sites of ubiquitination with the degradation efficacy for individual kinases. Such comparisons might help explaining isoform-selective degradation. As MDA-MB-231 cells were more sensitive to kinase degradation by the DCAF1-recruiting PROTACs, the ubiquitinomics experiments were performed in this cell line at 1 μM PROTAC concentration. Since ubiquitinoylation is faster than the resulting POI degradation, an earlier time point (30 min) was chosen as PROTAC treatment period (Figure S21). Indeed, kinase ubiquitination was found to correlate well with kinase degradation (Figure 5b). Interestingly, lysine modifications were detected for additional kinases for which no significant degradation was observed in the proteomic experiments. This suggests that the ubiquitination chain length, -frequency or -chain connectivity was insufficient for inducing kinase degradation. In addition, no lysine modification was observed for PROTACs **D-1e** and **D-2f**, which were essentially inactive in our proteomic study, suggesting that these degraders possibly failed to form productive ternary complexes capable of inducing target degradation (Figure S22-23).

### DCAF1- and CRBN-Recruiting PROTACs Have Partially Overlapping Degradable Kinomes

To compare the efficacy of the DCAF1- and CRBN-recruiting PROTACs, analogous CRBN-ligand-based PROTACs were investigated in proteomic experiments under identical conditions (Figure 6a, Figure S24–27). Strikingly, the observed differences in degradation efficacies between the CRBN- and DCAF1-recruiting PROTACs were kinase parent inhibitor-dependent. In general, the CRBN-recruiting PROTACs derived from **1inh**, **C-1a-d**, exhibited similar kinome-wide degradation efficacies as their DCAF1-recruiting counterparts, **D-1a-f** (Table S4). Although **C-1b** was the most potent *in cellulo* kinase binder across the **1inh**-derived PROTAC series, it did not induce any kinase degradation in either cell line. Interestingly, PROTAC **C-1a**, which only contained a different spacer moiety at the CRBN ligand, induced the degradation of several kinases in both cell lines. The series of degraders based on **2inh**, **C-2a-d**, degraded significantly more kinases than their DCAF1-based counterparts, **D-2a-f**. Conversely to their DCAF1-recruiting counterparts, U-87 cells were found to be more responsive to treatment with the CRBN-recruiting PROTACs **C-2a-d**, with generally more degraded kinases across the degrader series. A comparison of DMSO-treated samples of both cell lines revealed slightly higher levels of CRBN in U-87 cells (log_2_ fold change (U-87/MDA-MB-231) = 0.57), which potentially contributed to the observed trend. Interestingly, the degradable target spaces between the cell lines were highly similar for both CRBN-recruiting PROTAC series, as demonstrated by the comparative analysis of the t-statistics between the cell lines for the representative PROTACs **C-1a** and **C-2a** (Figure S28).

**Figure 6:**
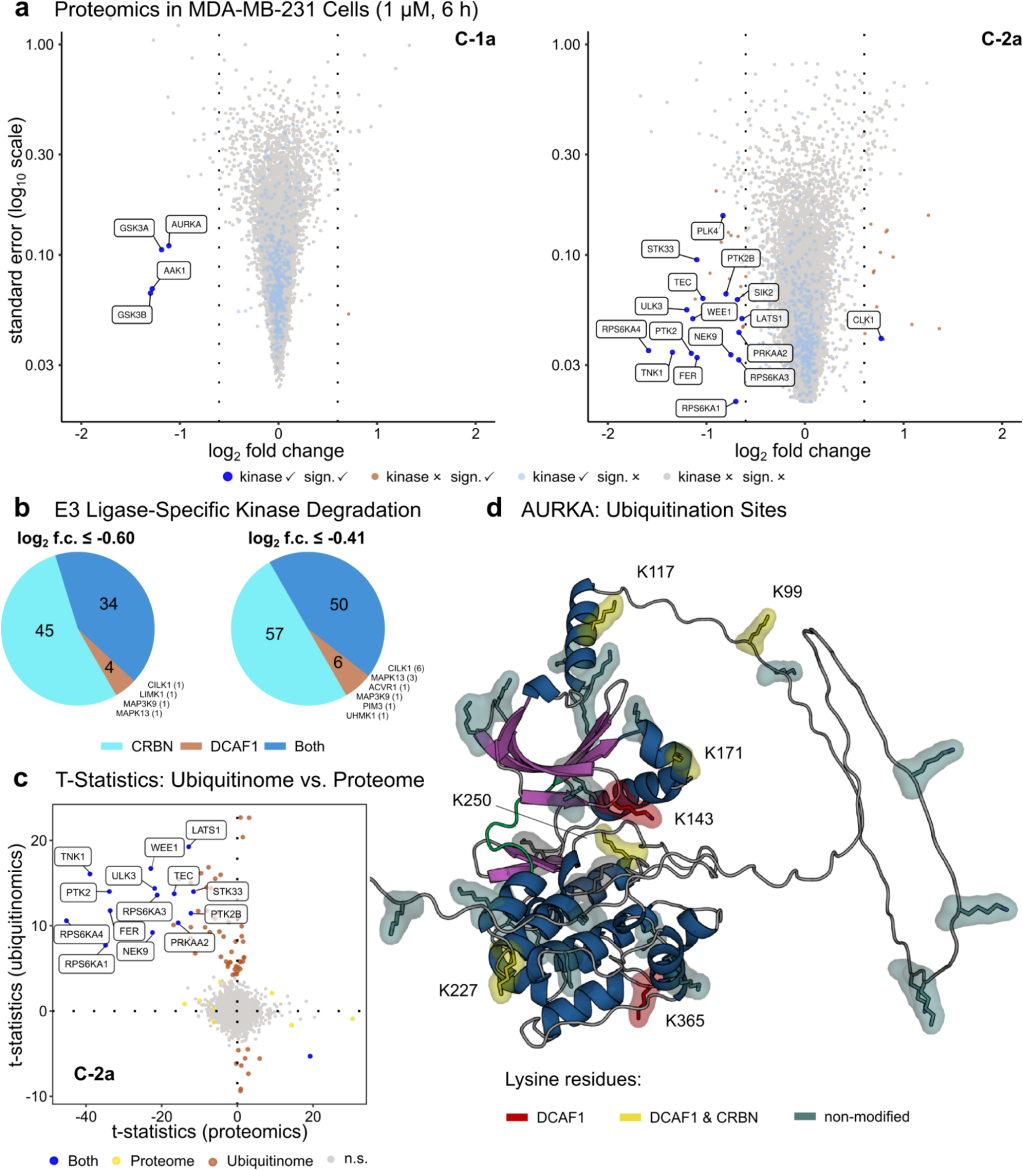
Comparing the degradomes and ubiquitinomes between the DCAF1 and CRBN E3 ligase systems. (a) MDA-MB-231 cells were treated with 1 μM of either C-1a or C-2a for 6 h, respectively. Significantly up- or protein kinases are shown as blue dots and are labeled whereas nonkinase proteins are highlighted as orange dots (p-values <0.01; log_2_ fold change ≤ -0.6 or ≥ 0.6). Protein levels that were not significantly changed are shown in light blue (kinases) and gray (nonkinase proteins). Shown data are mean values of biological replicates (*n* = 3). The complete data set is included in supplementary file 2. (b) Comparison of the degradomes of all DCAF1- and all CRBN-recruiting PROTACs within the applied fold change threshold. Kinases exclusively degraded are highlighted in orange (DCAF1-based PROTACs), light blue (CRBN-based PROTACs) and dark blue (both). (c) T-statistic comparison of the altered ubiquitinomes and proteomes for C-2a. (d) Visualized lysine ubiquitination sites on a truncated AlphaFold model of human AURKA (AF–O14965-F1-model_v4 ^45,46^ ; shortened by 13 amino acids (N-term) including K5, which was not ubiquitinated. Residues that were only modified with D-1a and D-2c are shown in red, residues modified with D-1a, D-2c and C-2a are shown in yellow. Nonmodified residues are depicted in blue.

Comparing the respective degradable target spaces of the DCAF1- and CRBN-recruiting PROTACs provided valuable insights into the E3 ligase-restricted target spaces of the degradable kinome. Of the 83 kinases that were degraded considering all proteomic experiments, 34 kinases were found to be degraded by both the DCAF1- as well as the CRBN-recruiting PROTACs, while the degradation of 45 kinases was only detected using CRBN-recruiting PROTACs. CILK1, LIMK1, MAP3K9 (MLK1) and MAPK13 (p38δ) were exclusively degraded by the DCAF1-recruiting PROTACs in our study (Figure 6b). This finding indicated a similar, albeit more target-selective degradation of DCAF1 compared to CRBN. Interestingly, six DCAF1-recruiting PROTACs did induce degradation of CILK1, which has not been reported to be targeted by PROTACs or any small molecule before. Similar to the DCAF1-recruiting PROTACs, AURKA was identified as one of the most degradable kinases together with AAK1 using CRBN-recruiting PROTACs (Figure S29). For CRBN-recruiting PROTACs, degradation was also observed for RNF166, ZNF91, ZNF692 and ZNF827, presenting reported neo-substrates of 4-hydroxy thalidomide.^43,44^

We also investigated the proteome-wide ubiquitination induced by the representative CRBN-recruiting PROTAC **C-2a** in MDA-MB-231 cells. Significant ubiquitination of lysine residues at the chosen time point was observed for 14 of the 16 degraded kinases, with the exceptions of SIK2 and PLK4, respectively (Figure 6c, Figure S30). In order to compare the ubiquitination efficacy of **C-2a** to those of the DCAF1-recruiting PROTACs, **D-1a** and **D-2c**, we selected a set of 16 kinases as representative targets, which were both degraded and ubiquitinated by **C-2a** and at least one of the DCAF1-recruiting PROTACs (Figure S31–33). For the majority of the degraded kinases, multiple diGLY-ε-lysine remnants were detected, with specific ubiquitination sites partially overlapping between the two E3 ligase systems. A clear correlation between the degradation efficacy and ubiquitination frequency of the same kinase was found. For instance, for DCAF1-recruiting PROTAC **D-2c**, ubiquitination of five lysines of PTK2 was observed, which was however insufficient to induce significant degradation of PTK2. In contrast, CRBN-recruiting PROTAC **C-2a** mediated the ubiquitination of 11 lysines, resulting in significant degradation of PTK2. In another instance, DCAF1-recruiting PROTACs **D-1a** and **D-2c** induced potent degradation of AURKA, while CRBN-recruiting PROTAC **C-2a** induced only weak AURKA degradation, which correlated with the scarce ubiquitination frequency of this PROTAC. Mapping the detected ubiquitination sites on the structure of AURKA revealed that lysine ubiquitination predominantly occurred at residues located on the opposite side of the hinge region at which the PROTACs’ interaction with the E3 ligase took place. Ubiquitination sites were largely restricted to structured regions of the protein (Figure 6d). Interestingly, while lysines K99, K117, K171, K227 and K250 were ubiquitinated by both E3 ligases, K143 and K365 were exclusively ubiquitinylated by DCAF1 engagement.

### PROTACs D-1a and D-2c Induce Kinase Degradation in a DCAF1- and Cullin-Dependent Manner

We next quantitatively characterized the dose- and time-dependent degradation of DCAF1- and CRBN-recruiting PROTACs using an nLuc-based assay that was established for a representative set of kinases. To this end, five stable K562-based luciferase reporter cell lines were used, each expressing nanoLUC fusions of AURKA^nLuc^, AURKB^nLuc^, CDK9^nLuc^, GSK3A^nLuc^ and PTK2^nLuc^, respectively.^47^ However, probably due to elevated expression levels in the stable cell lines, kinase degradation was found to be induced at generally higher PROTAC concentrations as compared to proteomic experiments across all five reporter cell lines (Figure S34). This hypothesis was further supported by the reduced degradation efficacy for the target-selective PROTACs that served as positive controls including **JB300** ^32^ (AURKA), **PT-65** ^48^ (GSK3A) and **BI-0319** ^33^ (PTK2). An onset of the typical hook effect was observed for some of the CRBN-recruiting PROTACs (**C-2a-d**), when tested against AURKA and PTK2 at higher concentrations. Nevertheless, almost complete degradation of AURKA was achieved by employing longer treatment times in the case of the representative PROTACs **D-1a** and **D-2c** (Figure S35).

We subsequently profiled four PROTACs representative of each series, namely **D-1a**, **D-2c**, **C-1a,** and **C-2a**, in dose–response experiments in Western blot assays to detect endogenous AURKA levels, as a representative kinase as AURKA^nLuc^ was degraded by all four PROTACs. MDA-MB-231 cells were treated in a 9-point dose-response series of the respective PROTACs (6-h treatment). Furthermore, we also included both parent kinase inhibitors, **1inh** and **2inh**, negative controls **D-1a** ^**n.c.**^ and **D-2c** ^**n.c.**^ and cotreatments with neddylation inhibitor MLN4924 as mechanistic control experiments to confirm that the kinases were degraded in a PROTAC- and UPS-dependent manner (Figure 7a-b). As hypothesized, all four PROTACs displayed a pronounced shift toward higher degradation potency as compared to the nLuc-based experiments due to target overexpression. Interestingly, the two most potent PROTACs, **D-2c** and **C-2a**, had DC_50_ values that were similar or better than the optimized and target-selective AURKA degrader **JB300**, revealing double-digit nanomolar DC_50_ values. Furthermore, while PROTAC **C-2a** induced more potent degradation of AURKA in comparison to its DCAF1 counterpart, **D-2c**, DCAF1-recruiting PROTAC **D-1a,** exhibited a DC_50_ value that was more potent than its CRBN-recruiting counterpart **C-1a**. While the parent kinase inhibitor **2inh** did not affect AURKA levels, treatment with **1inh** led to slight decreased AURKA levels; however, this effect was not observed in Jurkat cells.^18^ Neither of the negative controls, **D-1a** ^**n.c.**^ and **D-2c** ^**n.c.**^, affected AURKA levels, suggesting that AURKA degradation of the matching PROTACs, **D-1a** and **D-2c**, was indeed PROTAC- and DCAF1-mediated. Furthermore, cotreatment with the neddylation inhibitor MLN4924 led to complete rescue of AURKA protein levels, confirming the cullin-E3 ligase dependency of the observed AURKA degradation (Figure 7b, Figure S37). To quantify the observed AURKA degradation, we additionally tested these PROTACs in MV4–11 cells expressing tagged AURKA^HiBiT^ (Figure 7c-d, Figure S38, Table S6). All of the tested PROTACs induced dose-dependent AURKA^HiBiT^ degradation in the MV4–11 HiBiT cells. Interestingly, PROTAC-induced toxicity was slightly enhanced in the HiBiT reporter cell line in comparison to the nLuc reporter cells (Figures S36and S39). While the HiBiT data was mostly consistent with the proteomic data in respect of relative degrader potency, AURKA^HiBiT^ degradation was more pronounced than that of the endogenous protein kinase in the Western blot and proteomics experiments for most of the PROTACs, a phenomenon that was already observed previously for this reporter system.^18^ Most of the DCAF1-recruiting PROTACs induced comparable AURKA^HiBiT^ degradation as their CRBN-recruiting counterparts and PROTAC **D-2c** (DC_50_ = 17 ± 1 nM) matched the AURKA potency of positive control **JB300** (DC_50_ = 19 ± 3 nM).

**Figure 7:**
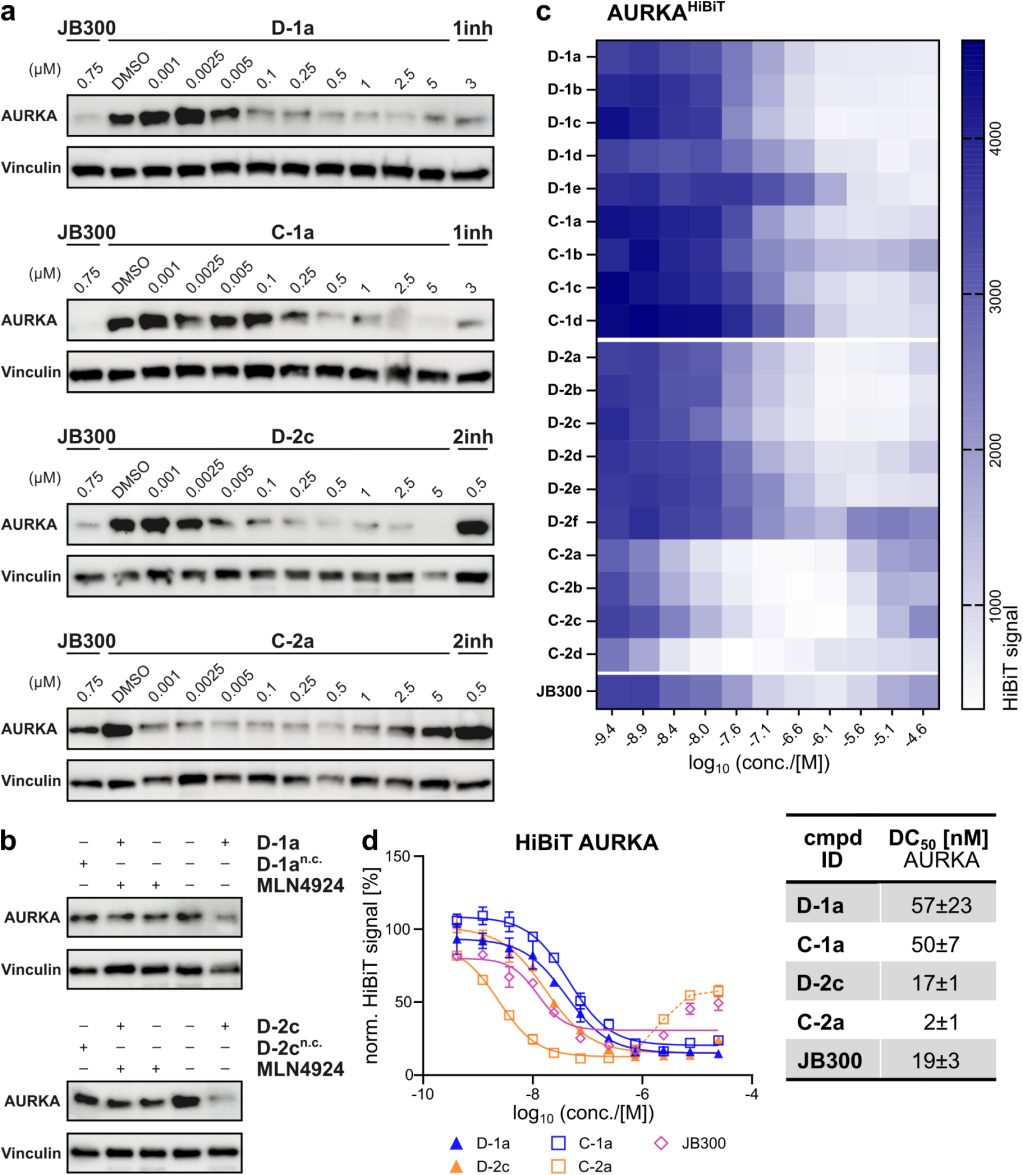
DCAF1-recruiting PROTACs are highly efficient AURKA degraders. (a) For the Western Blotting, MDA-MB-231 cells were treated with the indicated concentrations and compounds for 6 h. (b) Western Blotting in MDA-MB-231 cells after 6 h with D-1a, D-1a^n.c^., D-2c, D-2c^n.c^. (0.5 μM) and MLN4924 (1.0 μM). (c) All PROTACs were profiled in MV4–11 AURKA^HiBiT^ reporter cells after 6 h. The HiBiT signal represents the mean of biological replicates (*n* = 4). (d) Exemplary AURKA^HiBiT^ dose–response curves for selected PROTACs and the positive control JB300. Displayed results represent the mean of technical duplicates and error bars indicate the standard deviation. Dose response curves for all experiments including the second biological replicate are shown in Figure S38. All DC_50_ values are included in Table S6.

## Conclusion

The field of targeted protein degradation is growing rapidly due to the new and highly attractive properties of PROTACs in translational research and as chemical tools for target validation. Despite reports on the development of new E3 ligase ligands, the field has primarily focused on well-established E3 ligases such as CRBN and VHL. We believe that this focus is mainly based on the broad validation of both PROTAC systems, including the well-established medicinal chemistry, known positions for efficient linker attachment points and the documented large target space that is accessible by these two degrader systems.^17^ In contrast, new E3-ligands, such as for KEAP1,^49^ have been reported to have a narrow scope or they have only been validated targeting highly degradable POIs.^50^ Many of the current model systems for PROTAC development, particularly BRD4, are necessary for cell survival. Often, the high concentrations of PROTAC required by new E3-ligase ligand systems may trigger off-target toxicity and E3 ligase-independent target degradation.^34,51^ To this end, we recently developed a workflow that integrates target engagement and a general toxicity assessment, as well as the use of promiscuous kinase ligands to engage a large target space.^18^ Kinases are well-suited for such studies, as this target family is large, and many promiscuous ligands have been reported that can be used for the development of degraders that engage with many targets.^17^

DCAF1 is a Cullin-RING E3 ubiquitin ligase (CRL) with a similar subunit arrangement as CRBN, but it has a structurally diverse E3 ligase domain that belongs to the WD family.^29–31^ For this domain, chemically very diverse ligands have been developed.^19–22^ This diverse chemistry space offers more opportunities for PROTAC design compared to the glutarimide-based ligands of the CRBN system. Another interesting aspect of DCAF1 is its role in cancer cell proliferation. This suggests that, unlike CRBN-recruiting PROTACs, DCAF1 cannot simply be inactivated by E3 downregulation for instance by promoter methylation which has been identified as a resistance mechanism.^12,14^ DCAF1 would therefore be attractive for degrader development in oncology. However, DCAF1 ligands have only been used thus far for a very limited target space.^25,26^ Here, we report that DCAF1-recruiting PROTACs induce potent degradation of a large diversity of kinases. A side-by-side comparison with CRBN-based PROTACs revealed a similar target space within the kinase family. We therefore speculate that, apart from the PROTAC and recruited E3 ligase itself, also the E2/E3 structural arrangement may play an important role in determining which targets can be efficiently degraded.

Cell line sensitivity depended on DCAF1 expression levels, and dose-dependent proteomic experiments showed that higher PROTAC concentrations degraded a similar set of kinases in both studied cell lines, MDA-MB-231 and U-87. Interestingly, our concentration-dependent proteomic data also allowed an estimation of DC_50_ values for the degraded target that matched values determined for the targets selected for further follow up studies. However, no correlation was evident between degradation efficacy and either kinase engagement or cell line-specific kinase expression levels. Significantly more kinases were degraded by the CRBN-recruiting PROTAC series, which we assume might be a direct consequence of the tighter E3 ligase binding and cell penetration of the more optimized CRBN-ligand system. In terms of oral administration, the currently available DCAF1 ligands are at a disadvantage compared to CRBN ligands due to their suboptimal physicochemical properties (Table S7). The total polar surface area (TPSA) and H-bond acceptor (HBA) values of **cpd13** are well within the rule-of-5 space. However, the molecular weight (MW), rotatable bonds (RB) and unsaturated H-bond donor (HBD) counts of **cpd13** formally exceed the so-called property budget for a PROTAC according to the oral absorption framework recently developed by Hornberger and Araujo.^52,53^ Together, these considerations and the comparably lower affinity of **cpd13 toward DCAF1**, as indicated by our BRET-based target engagement assays, might present a potential limitation. This limitation likely reduced the overall kinase target space of the DCAF1-based PROTACs in our study. However, since **cpd13** is one of the first reported DCAF1 ligands, we are confident that future development campaigns will address these liabilities and produce highly potent DCAF1 ligands with more favorable physicochemical properties. It is noteworthy that no neo-substrate recruitment or molecular glue-like activity was observed for neither the DCAF1-recruiting PROTACs nor the DCAF1 ligand, **cpd13**, on a proteome-wide level. In contrast, degradation of known thalidomide neo-substrates was detected for the CRBN-recruiting PROTACs. In this regard, **cpd13** is at an advantage compared to thalidomide. However, neo-substrate degradation by CRBN has been addressed by the recent development of optimized CRBN ligands.^54^ Our data suggest that targeting DCAF1 is a versatile alternative to CRBN-based PROTACs and we hope that this study will pique interest in this E3-ligase for degrader development in the future.

## Experimental Section

### NanoBRET

Gene encoding full-length AURKA (Promega, NV1041), PTK2 (Promega, NV1921), CRBN (Promega, N2741) and DCAF1 (NanoLuc fusion tag cloned to C terminus, prepared in-house) proteins cloned in frame with NanoLuc fusion tag, were transfected into HEK293T cells using FuGENE HD (Promega, E2312) following manufacture’s protocol and proteins were allowed to express for 20 h at 37 °C and 5% CO_2_. Additionally, for CRBN and DCAF1 nanoBRET, DDB1 (human DNA damage-binding protein) expression vector (Promega, N2761) was cotransfected along with the E3 ligase vector, to improve the overall assay quality. Ten μL of transfected cells after trypsinization and resuspending in Opti-MEM (Life Technologies, 31985070) were dispensed into each well of the 384-well plate (Greiner 781207) at a cell density of 2 * 10^5^ cells/mL. For dose–response BRET measurements, compounds at various concentrations, immediately followed by Tracer K10 (Promega, Tracer DB ID: T000008) for AURKA and PTK2, CRBN Tracer (Promega, Tracer DB ID: T000018) for CRBN and DCAF1 Tracer (generated in-house/see synthesis protocols, Tracer K_D_: 1 μM) for DCAF1, at an optimum K_D_ concentration chosen from TracerDB (tracerdb.org)^55^ were pipetted using an Echo acoustic dispenser (Labcyte). The system was allowed to equilibrate for 2 h at 37 °C and 5% CO_2_ prior to BRET measurements. To measure BRET signal, the NanoBRET NanoGlo Substrate (Promega, N1573) was added as per the manufacturer’s protocol, and filtered luminescence was measured on a PHERAstar plate reader (BMG Labtech) equipped with a luminescence filter pair (450 nm BP filter (donor) and 610 nm LP filter (acceptor)). For lysed NanoBRET measurement, 25 nL of Digitonin (0.05 μg/μL) was pipetted to each well using Labcyte and the plate was incubated for 5 min at 37 °C and 5% CO_2_. After incubation, the BRET signals were measured by PHERAstar plate reader following the same procedure as intact NanoBRET measurement. Competitive displacement data were then normalized to controls and was graphed using GraphPad Prism 9 software employing a normalized 3-parameter curve fit with the following equation:

$$Y=100/\left(1+10^{\wedge }\left(X-\log IC_{50}\right)\right)$$

### Cell Titer Glo

PROTAC toxicity was determined using the CellTiter-Glo 2.0 Cell Viability Assay (Promega, G9241) following manufacture’s protocol. Ten μL of cells at a cell density of 2 * 10^5^ cells/mL were seeded into individual wells of white 384-well plate (Greiner 781207) and the cells were allowed to equilibrate for 1 h at 37 °C and 5% CO_2_. After equilibration, PROTACs at various concentrations were titrated using an Echo acoustic dispenser (Labcyte) and was incubated for respective time point at 37 °C and 5% CO_2_. Equal volume of assay reagent namely 10 μL was added to each well and incubated for 10 min at room temperature (RT). Filtered luminescence was measured on a PHERAstar plate reader (BMG Labtech) and data was evaluated using GraphPad Prism 9 software employing a normalized curve fit with the following equation:

$$Y=\mathrm{Bottom}+\left(\mathrm{Top}-\mathrm{Bottom}\right)/\left(1+10^{\wedge }{((\log EC_{50}‐X)}^{*}\mathrm{HillSlope}\right))$$

### Proteomics Cell Culture, Drug Treatments, and Sample Preparation

MDA-MB-231 (ATCC: HTB-26) and U-87 (ATCC: HTB-14) cells were cultured in DMEM (Thermo Fisher: 11965084) and EMEM (ATCC: 30–2003), respectively, each supplemented with 10% fetal bovine serum (FBS; Thermo Fisher, 26140079) and 1% penicillin/streptomycin (Thermo Fisher, 15140122). Cells were seeded at a density of 40,000 cells per well in 96-well plates (μClear, PS, F-bottom; Greiner Bio-One: 655097) in a final volume of 200 μL per well. Plates were incubated at 37 °C and 5% CO_2_ overnight to allow for cell attachment. Once at ∼80% confluency, cells were treated with respective compounds at aforementioned concentrations for 6h. Media was removed and cells were washed twice with 1X DPBS (Thermofisher: 14190144) to eliminate residual serum presence. After washing, plates were stored at −80 °C to induce cell lysis. Cell lysis and sample preparation was performed as described recently.^56^

### Ubiquitinomics Cell Culture, Drug Treatments, and Sample Preparation

Cell treatment and sample preparation were done according to our recently established protocol with a few modifications.^57^ In brief, MDA-MB-231 cells were cultured in 6-well plates and treated for 30 min with the specified compounds in quadruplicates, followed by lysis with SDC buffer. Protein concentrations were determined using the BCA assay (Merck-Millipore) and the proteins were digested overnight at 37 °C using 100:1 protein:trypsin ratio (Promega). After digestion, immunoprecipitation (IP) buffer (50 mM MOPS pH 7.2, 10 mM Na_2_HPO_4_, 50 mM NaCl) was added to the samples together with K-GG antibody-bead conjugate (Kit #59322 from Cell Signaling Technology), followed by a 2 h incubation on a rotor wheel. Beads washing and peptide elution were performed according to manufacturer’s instructions. The peptide eluate was desalted using in-house prepared, 200 μL two plug C18 StageTips (3 M EMPORE).^58^

### LC-MS Measurements (Proteomics, Ubiquitinomics)

About 800 ng or 50% of the tryptic peptides (for proteomics and ubiquitinomics, respectively) were loaded on 30 cm reversed phase columns (75 μm inner diameter, packed in-house with ReproSil Saphir C18 1.5 μm resin [ReproSil Saphir, Dr. Maisch GmbH]). The column temperature was maintained at 60 °C using a column oven. A Vanquish Neo UHPLC system (ThermoFisher) was directly coupled online with the mass spectrometer (timsTOF HT for proteomics, timsTOF Ultra 2 for ubiquitinomics, Bruker) via a nanoelectrospray source, and peptides were separated with a binary buffer system of buffer A (0.1% formic acid (FA)) and buffer B (80% acetonitrile plus 0.1% FA), at a flow rate of 300 nL/min (Proteomics) or 250 nL/min (Ubiquitinomics) for 50 min (Proteomics) or 45 min (Ubiquitinomics). For Proteomics, the mass spectrometer was operated in diaPASEF mode, and the acquisition method consisted of a MS1 scan, followed by 18 MS2 scans (2 ion mobility windows, 100 ms of accumulation/ramp time) with variable window sizes.^59^ The TIMS ion mobility range was set from 1/K0 = 1.35 to 0.65 Vs cm^2^ and the capillary voltage was set to 1750 V. Ubiquitinomics data acquisition was performed in slice-PASEF mode using a scheme with 5 × 15 slices and an accumulation/ramp time of 200 ms.^60^

### Proteomics Data Processing

Proteomics MS raw files were analyzed using DIA-NN.^61^ Reviewed UniProt entries (human, SwissProt [9606]) were used as protein sequence database. One missed cleavage, a maximum of one variable modification and N-terminal excision of methionine were allowed in case of proteomics. Carbamidomethylation of cysteines was set as fixed modification and methionine Oxidation (UniMod: 35) as variable modification. For ubiquitinomics, one missed cleavage and two variable modifications ((K-GG (UniMod:121) and N-terminal acetylation (UniMod:1)) were allowed. Carbamidomethylation of cysteines was set as fixed modification. – tims-scan was added as additional command. All raw data processing was performed in library-free mode.

### Biostatistics Data Analysis

DIA-NN outputs were further processed with R. The data matrix was filtered for valid values (precursor q-value ≤ 0.01) in 50% of all samples, or in 2/3 of the replicates of DMSO controls. These rules were selected to remove incomplete quantifications across all samples.

Significance testing of log_2_-transformed intensities was performed with LIMMA;^62^ compound treatments were compared to the DMSO controls. Multiple testing-corrected q-values <0.01 or <0.05 were considered as statistically significant, for proteomics and ubiquitinomics, respectively.

### Luciferase Assay

For the initial PROTAC screen, we opted for a scalable luminescent reporter setup using lentiviral expression systems where, open reading frames (ORF) of 5 kinases namely AURKA, AURKB, PTK2, GSK3A and CDK9, are expressed as Nanoluciferase fusions in K562 cells. The Nluc, K562 based reporter cell lines were obtained as a kind gift from Winter lab (Dr. Georg Winter, CeMM).^47^ The respective kinase expressing K562 reporter cells in FBS+PS supplemented RPMI (Gibco, 11875093), of 11 μL were dispensed into each well of a 384-well plate (Greiner, 781207) at a cell density of 3.2 * 10^5^ cells/mL. The cells were allowed to equilibrate overnight at 37 °C and 5% CO_2_. For dose–response degradation measurements, compounds at 11 various concentrations (Concentration Range: 0.41 nM–32.18 μM) were pipetted using an Echo acoustic dispenser (Labcyte), followed by 6 h incubation at 37 °C and 5% CO_2_. To measure luminescence, 5 μL of NanoBRET NanoGlo Substrate + Extracellular NanoLuc InHiBiTor (Promega, N2162) diluted in Opti-MEM, was added to each well as per the manufacturer’s protocol and was incubated at room temperature for 45 min prior to measurement. Luminescence was measured on a PHERAstar FSX plate reader (BMG Labtech) and raw luminescence signals were subsequently normalized for intraplate effects with DMSO (100%) and RPMI Media (0%) wells. Data was evaluated using GraphPad Prism 9 software employing a normalized curve fit with the following equation:

$$Y=\mathrm{Bottom}+\left(\mathrm{Top}-\mathrm{Bottom}\right)/\left(1+10^{\wedge }{((\log EC_{50}‐X)}^{*}\mathrm{HillSlope}\right))$$

### Cellular Degradation Kinetics

The K562 reporter cell line (AURKA^NLuc^) in FBS+PS supplemented RPMI (Gibco, 11875093) were harvested and the medium was exchanged to CO2-independent medium (Gibco, 18045–054). Endurazine substrate (Promega, N2570), according to manufacturer’s protocol was added to the medium exchanged cells, followed by dispensing 11 μL at a cell density of 1.5 * 10^5^ cells/mL into each well of a 384-well plate (Greiner, 781207) and incubated for 1.5 h, prior kinetic measurement for substrate activation. After incubation, compounds were titrated using an Echo acoustic dispenser (Labcyte) and the plate was sealed with BreathEasy plate seal (Sigma-Aldrich, Z380059) and continuously measured every 17 min for 61 h at 37 °C in a PHERAstar FSX plate reader (BMG Labtech) using the LUM plus module. Raw luminescence signals were subsequently normalized for intraplate effects with DMSO (100%) treated wells and the data was evaluated using GraphPad Prism 9 software, employing one phase exponential decay using the following equation

$$\left(Y={\left(Y0-\mathrm{NS}\right)}^{*}\exp\left(-K^{*}X\right)+\mathrm{NS}\right)$$

### Western Blotting

MDA-MB-231 cells were seeded in 6 well plates (Greiner) with 2 mL of cells/well with the cell count of 1*10^6^ cells/mL. The following day, cells were treated with compounds or DMSO for 6 h and then lysed with RIPA Buffer (50 mM HEPES pH 7.9, 140 mM NaCl, 1 mM EDTA, 1% Triton X-100, 0.1% SDS, 0.1% sodium deoxycholate) containing protease and phosphatase inhibitors (Sigma) at 4 °C for 20 min. The lysates were centrifuged for 15 min at 16000*g* and the supernatant was collected to perform the Western-Blot. Protein quantification was performed using bicinchoninic acid (BCA) assay. The SDS samples were prepared by adding 7 μL of 2.5X Laemmli buffer to 25 μg of protein from each cell lysate and was heated at 95 °C for 5 min. The samples were separated by a 10% BIS-TRIS polyacrylamide gel at 150 V for 80 min followed by transfer to PVDF membranes (Millipore) at 350 mA for 140 min. The membranes were blocked using 5% (wt/vol) BSA (in TBS-T, 20 mM Tris-HCl, pH 7.5, 150 mM NaCl and 0.1% (v/v) Tween 20) at room temperature for 1 h. The blots were incubated with respective primary antibodies at 1:1000 dilution (Cell Signaling technologies, Aurora A (D3E4Q) and Millipore Sigma, Vinculin (V4505)) overnight at 4 °C. The blots were washed thrice with TBST and incubated with 1:5000 dilution of horseradish peroxidase (HRP)-labeled respective secondary antibodies (Cell Signaling technologies, Antirabbit (7074S) and Anti-Mouse (7076S)) for 1 h. The blots were washed again with TBST for three times and were incubated with chemiluminescent HRP substrate (Bio-Rad) for 2 min. The blots were imaged and the signals were detected by Amersham ImageQuant 800 (Cytiva).

### HiBiT Assay

To investigate the degradation efficacy of PROTACs, MV4–11 cells stably expressing AURORA-A-HiBiT (MV4–11 ^AURORA–HiBiT^) under control of a PGK promotor were used.^32^ The MV4–11 AURKA^HiBiT^ cells in FBS+PS supplemented RPMI (Gibco, 11875093), of 11 μL were dispensed into each well of a 384-well plate (Greiner, 781207) at a cell density of 3.2 * 10^5^ cells/mL. The cells were allowed to equilibrate overnight at 37 °C and 5% CO_2_. For dose–response degradation measurements, compounds at 11 various concentrations (Concentration Range: 0.41 nM–32.18 μM) were pipetted using an Echo acoustic dispenser (Labcyte), followed by 6 h incubation at 37 °C and 5% CO_2_. After incubation, HiBiT Lytic detection reagent was prepared by dilution of LgBiT protein (1:100) and lytic substrate (1:50) in Lytic detection buffer (Promega, N3040) as per the manufacturer’s protocol. For detection, 10 μL of the prepared mix was added to the treated cells and incubated for 10 min at room temperature. Luminescence was measured on a PHERAstar FSX plate reader (BMG Labtech) and raw luminescence signals were subsequently normalized for intraplate effects with DMSO (100%) and RPMI Media (0%) wells. Data was evaluated using GraphPad Prism 9 software employing a normalized curve fit with the following equation:

$$Y=\mathrm{Bottom}+\left(\mathrm{Top}-\mathrm{Bottom}\right)/\left(1+10^{\wedge }{\left(\log EC_{50}‐X\right)}^{*}\mathrm{HillSlope}\right))$$

### Chemistry

All commercial chemicals and solvents were used without further purification. All reactions were performed under inert atmosphere (Ar). Reactions were monitored by thin layer chromatography using silica-coated ALUGRAM Xtra SIL G UV254 plates from Macherey Nagel. Product purification was performed on a PuriFlash Flash Column Chromatography System from Interchim using prepacked silica columns (PF-30SIHP-JP (30 μm), PF-50SIHP-JP (50 μm), PF-15C18HP (15 μm), PF-30C18HP (30 μm) or PF-15NH2 (15 μm)) or by preparative HPLC which was carried out on an Agilent 1260 Infinity II device using an Eclipse XDB-C18 (Agilent, 21.2 × 250 mm, 7 μm) reversed phase column. A suitable gradient (flow rate 21 mL/min.) was used, with 0.1% TFA in water (A) and 0.1% TFA in acetonitrile (B), as a mobile phase. The synthesized compounds were characterized by ^1^H NMR and ^13^C NMR spectroscopy. NMR spectra were measured in DMSO-*d*_6_, CDCl_3_ or DCMd_2_ on a Bruker AV500 or DPX600 spectrometer. Chemical shifts (δ) are reported in parts per million (ppm). Determination of the compound purity and/or mass by LCMS was carried out on an Agilent 1260 Infinity II device with a 1260 DAD HS detector (G7117C; 254 nm, 310 or 320 nm) and a LC/MSD device (G6125B, ESI pos. 100–1000) with a Poroshell 120 EC-C18 (Agilent, 3 × 150 mm, 2.7 μm) reversed phase column using 0.1% formic acid in water (A) and 0.1% formic acid in acetonitrile (B) as a mobile phase (methods A-D), on an Agilent 1260 Infinity II device, with a 1260 MWD detector (G7165A; 320 nm) on an Eclipse XDB-C18 (Agilent, 4.6 × 250 mm, 5 μm) reversed phase column using 0.1% TFA in water (A) and 0.1% TFA in acetonitrile (B) as a mobile phase (method E,F), on an Agilent 1200 series device with a Chromolith RP-18e 50–4.6 mm using 0.05% formic acid in water (A) and 0.04% formic acid in acetonitrile (B) as a mobile phase (method G) or on a Waters Acquity UPLC device (BSM + SM + CM-A + PDA + SQD2) with a Kinetex EVO-C18 1.7 μm 50–2.1 mm using 0.05% formic acid in water (A) and 0.04% formic acid in acetonitrile (B) as a mobile phase (method H). The compound purity was determined by LCMS (UV) at 254 nm, 310 or 320 nm and all compounds used for further biological characterizations showed >95% purity except for C-1d (91%), The following gradients were used: Method A: 0 min. 5% B – 2 min. 80% B – 5 min. 95% B – 7 min. 95% B (flow rate of 0.6 mL/min.). Method B: 0 min. 5% B – 0.6 min. 5% B – 4.2 min. 55% B – 7.4 min. 90% B – 9 min. 100% B – 11 min. 100% B (flow rate of 0.6 mL/min.). Method C: 0 min. 5% B – 2.8 min. 75% B – 7.2 min. 100% B – 7 min. 100% B (flow rate of 0.6 mL/min.). Method D: 0 min. 5% B – 0.4 min. 5% B – 8 min. 100% B – 10 min. 100% B (flow rate of 0.6 mL/min.). Method E: 0 min. 2% B – 0.4 min. 5% B – 7 min. 40% B – 20 min. 98% B (flow rate of 1.0 mL/min.). Method F: 0 min. 2% B – 2 min. 2% B – 10 min. 100% B – 15 min. 2% B – 17 min. 2% B – 20 min (flow rate of 1.0 mL/min.). Method G: 0 min 1% B – 2 min. 99% B – 2.5 min 99% B (flow rate of 3.3 mL/min.).

Method H: 0 min 1% B – 1 min 99% B – 1.3 min 99% B (flow rate of 0.9 mL/min.). For MS­(ESI+) spectra, samples were measured directly from TLC using TLC-MS interface 2 from Camag or injected without chromatography with a mobile phase flow rate of 400 μL/min (500:500:1 Methanol/Water/Formic Acid) and analyzed with a Thermo MSQ Plus mass spectrometer in both positive (0–0.75 min) and negative ionization modes (0.76–1.6 min) using a scan range of 100–2000 *m*/*z*. Spectra were analyzed in Thermo Qual Browser. High resolution (Orbitrap) measurements (Method 1) were executed on a MALDI LTQ XL Orbitrap spectrometer from Thermo Scientific or on a Exploris 480 Thermo (Bremen, Germany) mass spectrometer equipped with a heated electrospray source (HESI) and coupled to a liquid chromatography system Vanquish VF-P10-A binary pump, VF-A10-A auto sampler which was set to 10 °C and which was equipped with a 25 μL injection syringe and a 100 μL sample loop. Instead of a column a 0.18 mm, 600 mm length capillary was installed within the column compartment VH-C10-A. For automated direct infusion 2.0 μL sample was injected using a flow gradient with pure acetonitrile and water with 0.1% formic acid. For monitoring two full scan modes were selected with the following parameters. Polarity: positive; scan range: 100 to 1500 *m*/*z*; resolution: 480,000; AGC target: “Standard”; maximum IT: “Auto”. General settings: sheath gas flow rate: 20; auxiliary gas flow rate 5; sweep gas flow rate: 1; spray voltage: 3.5 kV; capillary temperature: 325 °C; S-lens RF level: 50; auxiliary gas heater temperature: 125 °C. For negative mode, all values were kept instead of the spray voltage which was set to 2.5 kV. Accurate MS (ESI+) measurements (Method 2) were executed on a MicrOTOF qII (ESI+) using the Hystar and OTOF control software from Bruker with a Thermo Ultimate 3000 using a flow of 200 μL/min with 50% acetonitrile and 50% water with 0.1% formic acid. Lockmass correction was performed with Bruker Data Analysis software with Carbamezepine (*m*/*z* = 237.10224 [M + H]^+^), Flunarizine (*m*/*z* = 405.21368 [M + H]^+^) and Reserpine (*m*/*z* = 609.28066 [M + H]^+^). All NMR, UV and mass spectra can be found in Figure S46-180. The synthesis of some of the intermediates was published earlier in ACS Chem. Biol. 2025, 20(2), 507–521^18^ (1inh, S-7, S-12, 2inh, S-28, S-45, S-47 and S-49) and in EMBO Rep. 2025^51^ (4-hydroxythalidomide and S-23).

### Synthesis of Parent Inhibitors and Tracer

#### 7-Bromo-2-[1- (4-chlorophenyl)­cyclohexyl]-3,4-dihydroquinazolin-4-one (**S-14**)

In a round-bottomed flask 1-(4-Chlorophenyl)­cyclohexanecarboxylic acid (**S-13**) (9.50 g; 37.8 mmol) was dissolved in THF (140 mL). Then 1-chloro-N,N,2-trimethyl-1-propenylamine (7.00 mL; 52.9 mmol) was added to the mixture, and it was stirred under argon at rt for 1 h. In another round-bottomed flask 2-amino-4-bromo-benzamide (8.30 g; 37.9 mmol) was dissolved in THF (140 mL) and TEA (15.7 mL; 113 mmol). The solution was added to the reaction mixture by a dropping funnel. The resulting mixture was stirred at rt for 5 h. Then the reaction mixture was evaporated to dryness and redissolved in EtOH (190 mL). A NaOEt solution, 21 wt % in EtOH (33.9 mL; 90.7 mmol) was added to the solution and it was left stirring at 50 °C for 16 h. The reaction mixture was diluted with deionized water (200 mL), extracted with EtOAc (3 × 250 mL), dried over Na_2_SO_4_, filtered, and evaporated to dryness. The crude product was purified by chromatography. The title compound was isolated as an orange solid (17.8 g; 59%). LCMS: R_t_ = 1.05 min (method H). MS­(ESI+) [*m*/*z*]: calculated = 419.1 [M+3H]^3+^; found = 419.1 [M + H]^+^.

#### *tert*-Butyl N-[2-(7- bromo-2-[1-(4-chlorophenyl)­cyclohexyl]­quinazolin-4-ylamino)­ethyl]­carbamate (**S-15**)

7-Bromo-2-[1-(4-chlorophenyl)­cyclohexyl]-3,4-dihydroquinazolin-4-one (**S-14**) (4.37 g; 5.72 mmol) and PyBOP (7.36 g; 13.7 mmol) were dissolved in DMF (44.0 mL). Then DIPEA (3.00 mL; 17.1 mmol) was added and the reaction mixture was left stirring under argon at rt for 3 h. Subsequently N-Boc-ethylenediamine (1.85 mL; 11.4 mmol) was added to the mixture and stirring was continued for 3 h. The reaction mixture was diluted with deionized water (100 mL), extracted with DCM (3 × 150 mL), dried over Na_2_SO_4_, filtered, and evaporated to dryness. The crude product was purified by chromatography. The title compound was isolated as a white powder (3.52 g; 86%). LCMS: R_t_ = 1.49 min (method H). MS­(ESI+) [*m*/*z*]: calculated = 561.2 [M+3H]^3+^; found = 561.1 [M + H]^+^.

#### *tert*-Butyl N-[2-({2-[1-(4-chlorophenyl)­cyclohexyl]-7-(piperazin-1-yl)­quinazolin-4-yl}­amino)­ethyl]­carbamate (**S-16**)

*tert*-Butyl N-[2-({7-bromo-2-[1-(4-chlorophenyl)­cyclohexyl]­quinazolin-4-yl}­amino)­ethyl]­carbamate (**S-15**) (4.20 g; 5.67 mmol), piperazine (2.44 g; 28.4 mmol) and sodium *tert*-butoxide 97% (0.67 g; 6.81 mmol) were dissolved in THF (35.0 mL). Then under argon RuPhos Pd G4 (762 mg; 851 μmol) was added and the mixture was left stirring at 70 °C for 4 h. The reaction mixture was diluted with deionized water (70 mL) and stirred for one further hour. The formed precipitation was filtered off with suction. The residue was dissolved in DCM and filtered off with suction again. The solution was absorbed onto Isolute and purified by flash chromatography to give the title compound as a yellowish solid (1.10 g; 31%). ^1^H NMR (500 MHz, DMSO-*d*_6_): δ = 8.04–7.90 (m, 2H), 7.47 (d, ^3^ *J* = 8.4 Hz, 2H), 7.30–7.20 (m, 3H), 6.95–6.89 (m, 2H), 3.57–3.52 (m, 4H), 3.51–3.44 (m, 2H), 3.24–3.13 (m, 6H), 2.85–2.78 (m, 2H), 1.97–1.89 (m, 2H), 1.59–1.51 (m, 2H), 1.50–1.44 (m, 3H), 1.43–1.29 (m, 10H) (Piperazine-NH not detectable due to water exchange). LCMS: R_t_ = 1.30 min (method G). MS­(ESI+) [*m*/*z*]: calculated = 565.3 [M + H]^+^; found = 565.2 [M + H]^+^.

#### 1-(4-(4-((2-Aminoethyl)­amino)-2-(1-(4-chlorophenyl)­cyclohexyl)­quinazolin-7-yl)-piperazin-1-yl)­ethan-1-one (**cpd13**)

A solution of acetic anhydride (5 μL, 0.049 mmol, 1.1 equiv) in 0.15 mL dry DCM was added dropwise to a solution of *tert*-butyl (2-((2-(1-(4-chlorophenyl)­cyclohexyl)-7-(piperazin-1-yl)­quinazolin-4-yl)­amino)­ethyl)­carbamate (**S-16**) (25 mg, 0.044 mmol, 1.0 equiv), 4-DMAP (1 mg, 0.009 mol, 0.2 equiv) and TEA (12 μL, 0.088 mmol, 2.0 equiv) in 0.15 mL dry DCM at 0 °C. After stirring the resulting reaction mixture at room temperature for 20 min, all volatiles were removed under reduced pressure. The obtained crude was loaded unto Celite and purified via flash column chromatography methanol/dichloromethane (0% → 5%) as eluent to give the Boc protected intermediate. The isolated intermediate was dissolved in 1.0 mL of a 25 vol % solution trifluoroacetic acid/dichloromethane. The resulting mixture was stirred at room temperature for 30 min. All volatiles were then removed under reduced pressure to give the title compound as yellow wax (quant.). ^1^H NMR (500 MHz, DMSO-*d*_6_): δ = 12.85 (bs, 1H), 9.87 (t, ^3^J = 5.0 Hz, 1H), 8.16 (d, ^3^J = 9.5 Hz, 1H), 8.19–8.07 (m, 3H), 7.50 (d, ^3^J = 8.7 Hz, 2H), 7.46 (dd, ^3^J = 9.5 Hz, ^4^J = 2.3 Hz, 1H), 7.40 (d, ^3^J = 8.7 Hz, 2H), 7.18 (d, ^4^J = 2.3 Hz, 1H), 3.97–3.93 (m, 2H), 3.63–3.57 (m, 4H), 3.57–3.52 (m, 2H), 3.49–3.44 (m, 2H), 3.20–3.13 (m, 2H), 2.80–2.73 (m, 2H), 2.13 (t, ^3^J = 10.7 Hz, 1H), 2.05 (s, 3H), 1.66–1.60 (m, 2H), 1.55–1.36 (m, 4H). ^13^C NMR (126 MHz, DMSO-*d*_6_): δ = 168.6, 165.7, 159.3, 154.4, 142.8, 141.0, 131.9, 128.6, 128.4, 125.5, 116.4, 102.0, 98.1, 48.9, 46.1, 45.9, 44.7, 37.9, 34.1, 24.9, 22.6, 21.2. LCMS: R_t_ = 3.79 min (method C); purity = 99%. MS­(ESI+) [*m*/*z*]: calculated = 507.26 [M + H]^+^; found = 507.25 [M + H]^+^. HRMS: (method 1): calculated = 507.2634 [M + H]^+^; found = 507.2627 [M + H]^+^.

#### *tert*-Butyl (2-((7-(4-acetylpiperazin-1-yl)-2-(1-(4-chlorophenyl)­cyclohexyl)­quinazolin-4-yl)­amino)­ethyl)­carbamate (**cpd13** ^**n.c.**^)

A solution of Acetic acid anhydride (9 μL, 97 μmol, 1.1eq) in DCM (300 μL) was added to a solution of **S-16** (50 mg, 88 μmol, 1.0eq), TEA (49 μL, 4.0eq) and 4-DMAP (2 mg, 18 μmol, 0.2eq) in DCM (300 μL) at 0 °C. The suspension was stirred at r.t. for 1 h and all volatiles were removed under reduced pressure. The crude product was purified using reversed phase flash column chromatography. The title compound was isolated as colorless oil (39 mg, 73%). ^1^H NMR (500 MHz, DCM-*d*_2_): δ = 7.59 (d, ^3^ *J* = 9.1 Hz, 1H), 7.46–7.43 (m, 2H), 7.21–7.17 (m, 2H), 7.07 (dd, ^3^ *J* = 9.1 Hz, ^4^ *J* = 2.5 Hz, 1H), 7.01 (d, J = 1.7 Hz, 1H), 6.52 (s, 1H), 5.10–5.03 (m, 1H), 3.77–3.70 (m, 2H), 3.68–3.58 (m, 4H), 3.43–3.38 (m, 2H), 3.38–3.34 (m, 2H), 3.34–3.30 (m, 2H), 2.81 (d, ^3^ *J* = 11.7 Hz, 2H), 2.10 (s, 3H), 2.07–1.98 (m, 2H), 1.65–1.48 (m, 6H), 1.39 (s, 9H). ^13^C NMR (126 MHz, DCM-*d*_2_): δ = 170.2, 169.3, 159.96, 158.0, 154.2, 152.6, 147.8, 131.5, 128.9, 128.3, 122.6, 116.6, 110.6, 106.7, 80.1, 50.3, 48.8, 48.7, 46.4, 43.6, 41.5, 40.8, 36.2, 28.6, 26.7, 24.0, 21.7. LCMS: R_t_ = 3.58 min (method C); purity = > 99%. MS­(ESI+) [*m*/*z*]: calculated = 607.3 [M + H]^+^; found = 607.3 [M + H]^+^. HRMS: (method 2): calculated = 607.3157 [M + H]^+^; found = 607.3167 [M + H]^+^.

#### 1-(4-(2-(1-(4-Chlorophenyl)­cyclohexyl)-4-((2-(dimethylamino)­ethyl)­amino)­quinazolin-7-yl)­piperazin-1-yl)­ethan-1-one (**cpd13-N**)

A solution **cpd13** ^**n.c.**^ (26.5 mg, 43.6 μmol, 1.0eq) in DCM/TFA (2/1, 3 mL) was stirred at r.t. for 1 h and all volatiles were removed under reduced pressure. The residue was dissolved DCM and the solvent was removed under reduced pressure (3x). Acetic acid (2.5 uL, 44 μmol, 1.0eq) and formaldehyde (6.8 uL, 92 μmol, 2.1eq, 37% in water) were added to a solution of the crude amine in MeOH (600 μL). The reaction mixture was stirred at r.t. for 2 h and NaBH_4_ (6.6 mg, 0.17 mmol, 4.0eq) was added and the solution was stirred at r.t. for 75 min. A saturated aqueous solution of NH_4_Cl and DCM was added, the layers were separated and the aqueous layer was extracted with DCM (3x). The combined organic layers were dried with MgSO_4_ and the solvent was removed under reduced pressure. The crude product was purified using preparative HPLC. The title compound was isolated as colorless solid (6 mg, 26%). ^1^H NMR (500 MHz, DCM-*d*_2_): δ = 7.59 (d, ^3^ *J* = 9.1 Hz, 1H), 7.46–7.42 (m, 2H), 7.21–7.16 (m, 2H), 7.06 (dd, ^3^ *J* = 9.0 Hz, ^4^ *J* = 2.5 Hz, 1H), 7.01 (d, ^4^ *J* = 2.5 Hz, 1H), 6.23 (t, ^3^ *J* = 4.7 Hz, 1H), 3.76–3.72 (m, 2H), 3.65–3.59 (m, 4H), 3.39–3.31 (m, 4H), 2.89–2.81 (m, 2H), 2.57 (t, ^3^ *J* = 6.0 Hz, 2H), 2.28 (s, 6H), 2.10 (s, 3H), 1.97 (t, ^3^ *J* = 10.3 Hz, 2H), 1.65–1.49 (m, 6H). ^13^C NMR (126 MHz, DCM-*d*_2_): δ = 170.2, 169.3, 159.6, 154.1, 152.7, 148.1, 131.5, 128.8, 128.28, 128.25, 122.5, 116.4, 110.7, 106.8, 58.3, 50.3, 48.9, 48.7, 46.4, 45.5, 41.5, 38.7, 36.3, 26.7, 24.1, 21.7. LCMS: R_t_ = 3.38 min (method C); purity = 98%. MS­(ESI+) [*m*/*z*]: calculated = 535.3 [M + H]^+^; found = 535.3 [M + H]^+^. HRMS: (method 2): calculated = 535.2947 [M + H]^+^; found = 535.2964 [M + H]^+^.

#### *tert*-Butyl (2-((7-(4-(2-(2-(2-(2-azidoethoxy)­ethoxy)­ethoxy)­acetyl)­piperazin-1-yl)-2-(1-(4-chlorophenyl)­cyclohexyl)­quinazolin-4-yl)­amino)­ethyl)­carbamate (**S-17**)

**S-16** (100 mg; 0.17 mmol) and 11-Azido-3,6,9-trioxaundecanoic acid (40.7 mg; 0.17 mmol) were dissolved in DMF (3.00 mL). Then pyridine (138 μL; 1.69 mmol) and 50% 2,4,6-tripropyl-1,3,5,2,4,6-trioxatriphosphinane 2,4,6-trioxide in DMF (593 μL; 1.01 mmol) were added and the solution was stirred at r.t. for 1.5 h. The reaction mixture was evaporated under reduced pressure. The residue was redissolved in DCM and extracted with deionized water. The combined organic layers were purified by chromatography. The title compound was isolated as a yellowish solid (88.0 mg; 64%). LCMS: R_t_ = 1.83 min (method G). MS­(ESI+) [*m*/*z*]: calculated = 780.4 [M + H]^+^; found = 780.2 [M + H]^+^.

#### *tert*-Butyl*N*-[2-({7-[4-(2-{2-[2-(2-aminoethoxy)­ethoxy]­ethoxy}­acetyl)­piperazin-1-yl]-2-[1-(4-chlorophenyl)­cyclohexyl]­quinazolin-4-yl}­amino)­ethyl]­carbamate (**S-18**)

*tert*-Butyl *N*-[2-({7-[4-(2-{2-[2-(2-azidoethoxy)­ethoxy]­ethoxy}­acetyl)­piperazin-1-yl]-2-[1-(4-chlorophenyl)­cyclohexyl]­quinazolin-4-yl}­amino)­ethyl]­carbamate (**S-17**) (88.0 mg; 0.11 mmol) was dissolved in methanol (6.50 mL). The solution was sonicated for 5 min and then flushed with argon for 5 min. Then nickel­(II) chloride (1.30 mg; 0.01 mmol) and sodium borohydride (10.4 mg; 0.27 mmol) were added and the solution was stirred at rt for 3 h protected from light. After incomplete conversion nickel­(II) chloride (1.30 mg; 0.01 mmol) and Sodium borohydride (10.4 mg; 0.27 mmol) were added and the solution was stirred at r.t. for further 2 h protected from light. The solvent was partially evaporated under reduced pressure. The residue was treated with water. The precipitate was filtered off by suction, washed with water and dried. The title compound was isolated as an off-white solid (41.0 mg; 39%). LCMS: R_t_ = 1.35 min (method G). MS­(ESI+) [*m*/*z*]: calculated = 377.7 [M/2+H]^+^; found = 377.6 [M/2+H]^+^.

#### *tert*-Butyl (2-((2-(1-(4-chlorophenyl)­cyclohexyl)-7-(4-(15-(5,5-difluoro-7-(1*H* **-**pyrrol-2-yl)-5*H* **-**4l4,5l4-dipyrrolo­[1,2-c:2“,1”-f]­[1,3,2]­diazaborinin-3-yl)-13-oxo-3,6,9-trioxa-12-azapentadecanoyl)­piperazin-1-yl)­quinazolin-4-yl)­amino)­ethyl)­carbamate (**S-19**)

12-(2-Carboxyethyl)-2,2-difluoro-4-(1H-pyrrol-2-yl)-1lambda5,3-diaza-2-boratricyclo­[7.3.0.0^{3,7}]­dodeca-1­(12),4,6,8,10-pentaen-1-ylium-2-uide (4.04 mg; 12.2 μmol) and HATU (6.97 mg; 18.3 μmol) were dissolved in DMF (250 μL), afterward *N*-methylmorpholine (4.07 μL; 36.7 μmol) was added and the solution was stirred at rt for 15 min *tert*-butyl N-[2-({7-[4-(2-{2-[2-(2-aminoethoxy)­ethoxy]­ethoxy}­acetyl)­piperazin-1-yl]-2-[1-(4-chlorophenyl)­cyclohexyl]­quinazolin-4-yl}­amino)­ethyl]­carbamate (**S-18**) (12.0 mg; 12.2 μmol) was dissolved in DMF (250 μL) and *N*-methylmorpholine (9.51 μL; 85.5 μmol) was added. Both solutions were unified and stirred at r.t. for 1 h. The reaction mixture was evaporated to dryness. The crude residue was purified by chromatography. The title compound was isolated as a dark blue solid (9.30 mg; 61%). LCMS: R_t_ = 1.95 min (method G). MS­(ESI+) [*m*/*z*]: calculated = 1065.5 [M + H]^+^; found = 1065.2 [M + H]^+^.

#### N-(2-(2-(2-(2-(4-(4-((2-Aminoethyl)­amino)-2-(1-(4-chlorophenyl)­cyclohexyl)­quinazolin-7-yl)­piperazin-1-yl)-2-oxoethoxy)­ethoxy)­ethoxy)­ethyl)-3-(5,5-difluoro-7-(1*H*-pyrrol-2-yl)-5*H*-4l4,5l4-dipyrrolo­[1,2-c:2“,1”-f]­[1,3,2]­diazaborinin-3-yl)­propenamide (**S-20**)

12-(2-{[2-(2-{2-[2-(4-{4-[(2-{[(tert-butoxy)­carbonyl]­amino}­ethyl)­amino]-2-[1-(4-chlorophenyl)­cyclohexyl]­quinazolin-7-yl}­piperazin-1-yl)-2-oxoethoxy]­ethoxy}­ethoxy)­ethyl]­carbamoyl}­ethyl)-2,2-difluoro-4-(1H-pyrrol-2-yl)-1lambda5,3-diaza-2-boratricyclo­[7.3.0.0^{3,7}]­dodeca-1­(12),4,6,8,10-pentaen-1-ylium-2-uide (**S-19**) (9.30 mg; 0.01 mmol) was dissolved in formic acid (1.00 mL) and stirred at 40 °C for 1 h. The solution was evaporated to dryness. The crude residue was purified by chromatography to give the title compound (1.60 mg; 19%) as a dark blue solid. LCMS: R_t_ = 1.29 min (method G). MS­(ESI+) [*m*/*z*]: calculated = 965.5 [M + H]^+^; found = 965.1 [M + H]^+^.

### Synthesis of Promiscuous Kinase PROTACs

#### *tert*-Butyl 3-(2-(2-(4-(2-((4-(cyanomethyl)­phenyl)­amino)-6-((5-cyclopropyl-1*H*-pyrazol-3-yl)­amino)­pyrimidin-4-yl)­piperazin-1-yl)­ethoxy)­ethoxy)­propanoate (**S-27**)

2-(4-((4-((5-cyclopropyl-1H-pyrazol-3-yl)­amino)-6-(piperazin-1-yl)­pyrimidin-2-yl)­amino)­phenyl)­acetonitrile **(S-7)** (200 mg, 0.385 mmol, 1.00 equiv) and potassium carbonate (160 mg, 1.16 mmol, 3.00 equiv) were suspended in 1.0 mL dry acetonitrile. A solution of *tert*-butyl 3-(2-(2-bromoethoxy)­ethoxy)­propanoate **(S-24)** (229 mg, 0.770 mmol, 2.00 equiv) in 1.0 mL dry acetonitrile was added and the reaction mixture was stirred at 80 °C overnight. After cooling to room temperature, all volatiles were removed under reduced pressure. The obtained crude was loaded unto Celite and purified via reverse phase flash column chromatography using acetonitrile/water (5% → 100%) as eluent first followed by a second purification step via reverse phase flash column chromatography using acetonitrile/water (5% → 100%) as eluent to give the title compound (144 mg, 60%). ^1^H NMR (500 MHz, DMSO-*d*_6_): δ = 12.48–11.68 (m, 1H), 9.75–8.55 (m, 2H), 7.79–7.53 (m, 2H), 7.26–7.03 (m, 2H), 6.23–5.16 (m, 2H), 3.92 (s, 2H), 3.60 (t, ^3^J = 6.2 Hz, 2H), 3.58–3.42 (m, 12H), 2.53–2.47 (m, 2H), 2.42 (t, ^3^J = 6.2 Hz, 2H), 1.91–1.75 (m, 1H), 1.40–1.38 (m, 2H), 1.39 (s, 9H), 0.99–0.77 (m, 2H), 0.74–0.57 (m, 2H). ^13^C NMR (126 MHz, DMSO-*d*_6_): δ = 170.4, 163.2, 160.7, 158.7, 149.2, 141.0, 128.1, 122.4, 119.6, 118.9, 92.5, 79.7, 77.1, 70.3, 69.64, 69.62, 69.5, 68.2, 66.2, 57.3, 52.8, 50.0, 43.9, 35.9, 35.8, 32.2, 28.5, 27.8, 21.7, 7.6. LCMS: R_t_ = 6.840 min (method C). MS­(ESI+) [*m*/*z*]: calculated = 632.36 [M + H]^+^; found = 632.40 [M + H]^+^.

#### **2-(4-((4-(4-(2-(2-(3-(4-(4-((2-Aminoethyl)­amino)-2-(1-(4-chlorophenyl)­cyclohexyl)­quinazolin-7-yl)­piperazin-1-yl)-3-oxopropoxy)­ethoxy)­ethyl)­piperazin-1-yl)-6-((5-cyclopropyl-1** *H* **-pyrazol-3-yl)­amino)­pyrimidin-2-yl)­amino)­phenyl)­acetonitrile (D-1a)**

*tert*-Butyl 3-(2-(2-(4-(2-((4-(cyanomethyl)­phenyl)­amino)-6-((5-cyclopropyl-1*H*-pyrazol-3-yl)­amino)­pyrimidin-4-yl)­piperazin-1-yl)­ethoxy)­ethoxy)­propanoate **(S-27)** (20 mg, 0.032 mmol, 1.0 equiv) was dissolved in 1.0 mL of a 25 vol % solution of trifluoroacetic acid/dichloromethane. The resulting mixture was stirred at room temperature for 45 min. All volatiles were removed under reduced pressure and the residue was coevaporated with dichloromethane twice to give the free acid as the according trifluoroacetate salt (quant.). The obtained trifluoroacetate salt and DIPEA (11 μL, 0.063 mmol, 2.0 equiv) were dissolved in 150 μL dry DMF, a solution of PyBOP (20 mg, 0.038 mmol, 1.2 equiv) in 100 μL was added and the resulting reaction mixture was stirred at room temperature for 5 min. A solution of *tert*-butyl (2-((2-(1-(4-chlorophenyl)­cyclohexyl)-7-(piperazin-1-yl)­quinazolin-4-yl)­amino)­ethyl)­carbamate **(S-16)** (20 mg, 0.035 mmol, 1.1 equiv) in 150 μL dry DMF was added and the resulting reaction mixture was then stirred at room temperature for 2 h. All volatiles were removed under reduced pressure. The isolated crude was dissolved in 1.0 mL of a 25 vol % solution of trifluoroacetic acid/dichloromethane. The resulting mixture was stirred at room temperature for 30 min. All volatiles were removed under reduced pressure and the residue was coevaporated with dichloromethane twice to give the according trifluoroacetate salt. The obtained crude was purified via preparative HPLC using acetonitrile/water (5% → 100%) with 0.1% TFA additive as eluent to give the title compound as the according trifluoroacetate salt (1:2) (12 mg, 30%). ^1^H NMR (500 MHz, DMSO-*d*_6_): δ = 12.84 (bs, 1H), 10.23–9.26 (m, 3H), 8.16–8.04 (m, 4H), 7.67 (d, ^3^J = 8.3 Hz, 2H), 7.49 (d, ^3^J = 8.4 Hz, 2H), 7.44–7.39 (m, 3H), 7.25 (d, ^3^J = 8.0 Hz, 2H), 7.18–7.11 (m, 1H), 5.88 (bs, 2H), 4.27 (bs, 2H), 3.95 (s, 2H), 3.96–3.90 (m, 2H), 3.80–3.77 (m, 2H), 3.68 (t, ^3^J = 6.5 Hz, 2H), 3.65–3.56 (m, 10H), 3.54–3.50 (m, 2H), 3.47–3.43 (m, 2H), 3.37–3.26 (m, 4H), 3.19–3.08 (m, 4H), 2.80–2.71 (m, 2H), 2.64 (t, ^3^J = 6.5 Hz, 2H), 2.19–2.08 (m, 2H), 1.91–1.84 (m, 1H), 1.66–1.59 (m, 2H), 1.55–1.35 (m, 4H), 0.95–0.90 (m, 2H), 0.71–0.67 (m, 2H). ^13^C NMR (126 MHz, DMSO-*d*_6_): δ = 169.1, 165.7, 162.3, 159.3, 154.3, 142.9, 139.8, 131.8, 128.6, 128.4, 128.3, 125.4, 123.7, 119.5, 116.3, 102.0, 77.1, 69.6, 69.3, 66.5, 64.1, 54.9, 50.7, 48.9, 46.1, 45.8, 43.9, 37.9, 34.0, 32.8, 24.9, 22.6, 21.8, 16.7, 7.9, 7.3. LC: R_t_ = 13.85 min (method E); purity = 99%. MS­(ESI+) [*m*/*z*]: calculated = 1022.53 [M + H]^+^; found = 1022.61 [M + H]^+^. HRMS: (method 1): calculated = 1022.5391 [M + H]^+^; found = 1022.5386 [M + H]^+^.

#### ***tert*** **-Butyl (2-((2-(1-(4-chlorophenyl)­cyclohexyl)-7-(4-(3-(2-(2-(4-(2-((4-(cyanomethyl)­phenyl)­amino)-6-((5-cyclopropyl-1** *H* **-pyrazol-3-yl)­amino)­pyrimidin-4-yl)­piperazin-1-yl)­ethoxy)­ethoxy)­propanoyl)­piperazin-1-yl)­quinazolin-4-yl)­amino)­ethyl)­carbamate (D-1a** ^**n.c.**^)

A solution of **S-27** (24 mg, 38 μmol, 1.0 equiv) in DCM/TFA (4.0 mL, 1/1) was stirred at r.t. for 3 h and all volatiles were removed under reduced pressure. The residue was dissolved in DCM and all volatiles were removed under reduced pressure (2x). PyBOP (24 mg, 46 μmol, 1.2 equiv) was added to a solution of the crude acid and DIPEA (16 μL, 0.11 mmol, 3.0 equiv) in DMF/THF (2.0 mL, 1/1) and the mixture was stirred at r.t. for 3 min. The DCAF1 ligand **(S-16)** (24 mg, 42 μmol, 1.1 equiv) was added and the reaction mixture was stirred at r.t. overnight (15 h) and all volatiles were removed under reduced pressure. The crude material was purified using flash column chromatography (DCM/MeOH 10%) and preparative HPLC (H_2_O/MeCN/FA). The title compound was isolated as a pale yellow solid (17 mg, 40%). ^1^H NMR (600 MHz, MeOD): 8.43–8.24 (m, 4H), 7.95 (d, ^3^ *J* = 9.3 Hz, 1H), 7.61 (d, ^3^ *J* = 8.3 Hz, 2H), 7.48 (d, ^3^ *J* = 8.4 Hz, 2H), 7.35 (d, ^3^ *J* = 8.4 Hz, 2H), 7.24 (d, ^3^ *J* = 8.3 Hz, 2H), 7.21 (d, ^3^ *J* = 8.8 Hz, 1H), 6.96 (s, 1H), 5.74 (s, 1H), 3.86–3.76 (m, 8H), 3.76–3.70 (m, 10H), 3.65 (s, 4H), 3.50 (s, 2H), 3.42 (t, *J* = 5.3 Hz, 4H), 3.13–3.09 (m, 6H), 2.70 (t, ^3^ *J* = 5.7 Hz, 4H), 2.23 (t, ^3^ *J* = 11.9 Hz, 2H), 1.91–1.85 (m, 3H), 1.69 (s, 2H), 1.66–1.55 (m, 3H), 1.52–1.44 (m, 1H), 1.36 (s, 9H), 0.97–0.93 (m, 2H), 0.72–0.68 (m, 2H). ^13^C NMR (151 MHz, MeOD): δ = 172.3, 168.2, 167.3, 164.6, 161.9, 161.0, 159.9, 156.2, 141.7, 134.1, 129.9, 129.6, 129.4, 126.0, 124.9, 120.9, 120,0 117.4, 103.9, 100.0, 91.4, 80.3, 71.4, 71.3, 68.9, 68.1, 67.1, 57.7, 53.3, 50.6, 47.7, 47.3, 45.9, 43.7, 43.6, 42.2, 40.5, 35.9, 34.4, 28.7, 26.6, 26.5, 24.1, 22.9, 8.3. LCMS: R_t_ = 4.41 min (method D); purity = > 99%. MS­(ESI+) [*m*/*z*]: calculated = 561.8 [M/2+H]^+^; found = 562.0 [M/2+H]^+^. HRMS: (method 2): calculated = 1122.5915 [M + H]^+^; found = 1122.5978 [M + H]^+^.

#### **2-(4-((4-(4-(2-(2-(2-(3-(4-(4-((2-Aminoethyl)­amino)-2-(1-(4-chlorophenyl)­cyclohexyl)­quinazolin-7-yl)­piperazin-1-yl)-3-oxopropoxy)­ethoxy)­ethoxy)­ethyl)­piperazin-1-yl)-6-((5-cyclopropyl-1** *H* **-pyrazol-3-yl)­amino)­pyrimidin-2-yl)­amino)­phenyl)­acetonitrile (D-1b)**

The synthesis followed the general procedure of **D-1a** starting from t*ert*-butyl 3-(2-(2-(2-(4-(2-((4-(cyanomethyl)­phenyl)­amino)-6-((5-cyclopropyl-1H-pyrazol-3-yl)­amino)­pyrimidin-4-yl)­piperazin-1-yl)­ethoxy)­ethoxy)­ethoxy)­propanoic acid **(S-28)** (20 mg, 0.030 mmol). The title compound was isolated as trifluoroacetate salt (1:2) (15 mg, 39%). ^1^H NMR (500 MHz, DMSO-*d*_6_): δ = 12.86 (bs, 1H), 10.31–9.43 (m, 3H), 8.17–8.07 (m, 4H), 7.65 (d, ^3^J = 8.5 Hz, 2H), 7.49 (d, ^3^J = 8.8 Hz, 2H), 7.44–7.38 (m, 3H), 7.27 (d, ^3^J = 8.6 Hz, 2H), 7.18–7.15 (m, 1H), 5.97–5.80 (m, 2H), 4.27 (bs, 2H), 3.97 (s, 2H), 3.97–3.91 (m, 2H), 3.80–3.76 (m, 2H), 3.67–3.55 (m, 12H), 3.53–3.50 (m, 6H), 3.47–3.44 (m, 2H), 3.29–3.27 (m, 4H), 3.23–3.07 (m, 4H), 2.81–2.71 (m, 2H), 2.62 (t, ^3^J = 6.7 Hz, 2H), 2.19–2.08 (m, 2H), 1.92–1.86 (m, 1H), 1.67–1.59 (m, 2H), 1.56–1.36 (m, 4H), 0.96–0.91 (m, 2H), 0.72–0.68 (m, 2H). ^13^C NMR (126 MHz, DMSO-*d*_6_): δ = 169.1, 165.7, 162.2, 159.3, 154.6, 142.8, 139.4, 131.8, 128.6, 128.41, 128.37, 125.4, 124.1, 119.8, 119.5, 116.3, 102.0, 98.2, 91.0, 77.0, 69.70, 69.66, 69.6, 69.4, 66.6, 64.1, 54.9, 50.7, 48.9, 46.1, 45.8, 43.9, 41.1, 37.9, 34.0, 32.8, 24.9, 22.6, 21.8, 8.0, 7.2. LC: R_t_ = 13.75 min (method E); purity = 99%. MS­(ESI+) [*m*/*z*]: calculated = 1066.55 [M + H]^+^; found = 1066.65 [M + H]^+^. HRMS: (method 1): calculated = 1066.5653 [M + H]^+^; found = 1066.5651 [M + H]^+^.

#### ***tert*** **-Butyl 1-(4-(2-((4-(cyanomethyl)­phenyl)­amino)-6-((5-cyclopropyl-1** *H* **-pyrazol-3-yl)­amino)­pyrimidin-4-yl)­piperazin-1-yl)-3,6,9,12,15-pentaoxaoctadecan-18-oate (S-29)**

The synthesis followed the general procedure of **S-27** using *tert*-butyl 1-bromo-3,6,9,12,15-pentaoxaoctadecan-18-oate **(S-26)** (248 mg, 0.578 mmol). The title compound was isolated as a pale-yellow resin (149 mg, 68%). ^1^H NMR (500 MHz, DMSO-*d*_6_): δ = 12.44–11.50 (m, 1H), 9.79–8.46 (m, 2H), 7.81–7.60 (m, 2H), 7.20 (d, ^3^J = 7.9 Hz, 2H), 6.26–5.19 (m, 2H), 3.92 (s, 2H), 3.63–3.39 (m, 26H), 2.54–2.46 (m, 4H), 2.40 (t, ^3^J = 6.2 Hz, 2H), 1.88–1.78 (m, 1H), 1.38 (s, 9H), 0.92–0.82 (m, 2H), 0.70–0.60 (m, 2H). ^13^C NMR (126 MHz, DMSO-*d*_6_): δ = 170.4, 163.2, 160.7, 158.7, 145.2, 140.9, 122.4, 119.6, 118.8, 92.4, 79.7, 77.1, 69.8, 69.78, 69.75, 69.7, 69.6, 68.2, 66.2, 57.2, 52.8, 43.9, 35.8, 27.7, 21.7, 7.6. LCMS: R_t_ = 5.05 min (method C). MS (ESI+) [*m*/*z*]: calculated = 764.44 [M + H]^+^; found = 764.45 [M + H]^+^.

#### **2-(4-((4-(4-(18-(4-(4-((2-Aminoethyl)­amino)-2-(1-(4-chlorophenyl)­cyclohexyl)­quinazolin-7-yl)­piperazin-1-yl)-18-oxo-3,6,9,12,15-pentaoxaoctadecyl)­piperazin-1-yl)-6-((5-cyclopropyl-1** *H* **-pyrazol-3-yl)­amino)­pyrimidin-2-yl)­amino)­phenyl)­acetonitrile (D-1c)**

The synthesis followed the general procedure of **D-1a** starting from *tert*-butyl 1-(4-(2-((4-(cyanomethyl)­phenyl)­amino)-6-((5-cyclopropyl-1*H*-pyrazol-3-yl)­amino)­pyrimidin-4-yl)­piperazin-1-yl)-3,6,9,12,15-pentaoxaoctadecan-18-oate **(S-29)** (25 mg, 0.033 mmol). The title compound was isolated as trifluoroacetate salt (1:2) (13 mg, 29%). ^1^H NMR (500 MHz, DMSO-*d*_6_): δ = 12.82 (bs, 1H), 10.37–9.24 (m, 3H), 8.14 (d, ^3^J = 9.5 Hz, 1H), 8.09 (bs, 3H), 7.66 (d, ^3^J = 8.4 Hz, 2H), 7.49 (d, ^3^J = 8.7 Hz, 2H), 7.44 (d, ^3^J = 9.5 Hz, 1H), 7.40 (d, ^3^J = 8.7 Hz, 2H), 7.26 (d, ^3^J = 8.4 Hz, 2H), 7.19–7.11 (m, 1H), 5.88 (m, 2H), 4.26 (bs, 2H), 3.96 (s, 2H), 3.96–3.91 (m, 2H), 3.80–3.77 (m, 2H), 3.65–3.55 (m, 12H), 3.54–3.43 (m, 18H), 3.38–3.28 (m, 4H), 3.20–3.07 (m, 4H), 2.82–2.69 (m, 2H), 2.61 (t, ^3^J = 6.6 Hz, 2H), 2.16–2.05 (m, 2H), 1.91–1.84 (m, 1H), 1.67–1.59 (m, 2H), 1.56–1.35 (m, 4H), 0.95–0.89 (m, 2H), 0.71–0.67 (m, 2H). ^13^C NMR (126 MHz, DMSO-*d*_6_): δ = 169.1, 165.7, 162.3, 159.3, 154.4, 142.9, 141.0, 139.6, 131.8, 128.6, 128.4, 128.3, 125.4, 123.8, 119.6, 119.5, 116.3, 102.0, 98.1, 91.0, 77.1, 69.77, 69.75, 69.72, 69.70, 69.62, 69.56, 69.4, 66.6, 64.1, 54.9, 50.7, 48.9, 46.1, 45.8, 44.0, 41.0, 37.9, 34.1, 32.8, 24.9, 22.6, 21.8, 7.9, 7.3. LC: R_t_ = 13.94 min (method E); purity = 98%. MS­(ESI+) [*m*/*z*]: calculated = 1154.61 [M + H]^+^; found = 1154.74 [M + H]^+^. HRMS: (method 1): calculated = 1154.6177 [M + H]^+^; found = 1154.6178 [M + H]^+^.

#### **Ethyl 7-(4-(2-((4-(Cyanomethyl)­phenyl)­amino)-6-((5-cyclopropyl-1** *H* **-pyrazol-3-yl)­amino)-pyrimidin-4-yl)­piperazin-1-yl)­heptanoate (S-32)**

*tert*-Butyl 4-(2-((4-(cyanomethyl)­phenyl)­amino)-6-((5-cyclopropyl-1*H*-pyrazol-3-yl)­amino)­pyrimidin-4-yl)­piperazine-1-carboxylate **(1inh)** (50 mg, 0.097 mmol, 1.0 equiv) was dissolved in 1.0 mL of a 25 vol % solution of trifluoroacetic acid/dichloromethane. The resulting mixture was stirred at room temperature for 20 min. All volatiles were removed under reduced pressure and the residue was coevaporated with dichloromethane twice to give the free amine as the according trifluoroacetate salt (quant.). The trifluoroacetate salt and potassium carbonate (40 mg, 0.29 mmol, 3.0 equiv) were then suspended in 0.5 mL dry acetonitrile. A solution of ethyl 7-bromoheptanoate **(S-30)** (46 mg, 0.19 mmol, 2.0 equiv) in 0.5 mL dry acetonitrile was added and the resulting mixture was stirred at 80 °C overnight. After cooling to room temperature, all volatiles were removed under reduced pressure. The obtained crude was loaded unto Celite and purified via flash column chromatography using methanol/dichloromethane (0% → 10%) as eluent to give the title compound as a colorless solid (31 mg, 56%). ^1^H NMR (500 MHz, DMSO-*d*_6_): δ = 12.65–11.55 (m, 1H), 9.83–8.44 (m, 2H), 7.72 (d, ^3^J = 8.1 Hz, 2H), 7.21 (d, ^3^J = 8.5 Hz, 2H), 6.58–5.21 (m, 2H), 4.04 (q, ^3^J = 7.1 Hz, 2H), 3.92 (s, 2H), 3.50–3.43 (m, 4H), 2.47–2.37 (m, 4H), 2.31–2.24 (m, 2H), 2.27 (t, ^3^J = 7.4 Hz, 2H), 1.86–1.80 (m, 1H), 1.55–1.49 (m, 2H), 1.47–1.40 (m, 2H), 1.30–1.25 (m, 4H), 1.17 (t, ^3^J = 7.1 Hz, 3H), 0.91–0.85 (m, 2H), 0.70–0.63 (m, 2H). ^13^C NMR (126 MHz, DMSO-*d*_6_): δ = 172.9, 163.3, 160.5, 158.7, 148.7, 145.7, 140.9, 128.1, 122.4, 119.6, 118.8, 92.2, 77.0, 60.6, 59.6, 57.8, 52.5, 43.9, 33.5, 32.3, 28.4, 28.4, 26.6, 26.0, 25.2, 24.5, 24.4, 21.7, 14.1, 7.67. LCMS: R_t_ = 3.72 min (method C). MS­(ESI+) [*m*/*z*]: calculated = 572.35 [M + H]^+^; found = 572.4 [M + H]^+^.

#### **7-(4-(2-((4-(Cyanomethyl)­phenyl)­amino)-6-((5-cyclopropyl-1** *H* **-pyrazol-3-yl)­amino)­pyrimidin-4-yl)­piperazin-1-yl)­heptanoic Acid (S-34)**

Ethyl 10-(4-(2-((4-(cyanomethyl)­phenyl)­amino)-6-((5-cyclopropyl-1*H*-pyrazol-3-yl)­amino)­pyrimidin-4-yl)­piperazin-1-yl)­decanoate **(S-33)** (25 mg, 0.044 mmol, 1.0 equiv) and lithium hydroxide monohydrate (9 mg, 0.2 mmol, 5 equiv) were dissolved in 1.0 mL methanol/water (4:1). The resulting mixture was stirred at 40 °C overnight. The mixture was then carefully acidified with 1 M HCl_aqu._ to a pH of approximately 1. All volatiles were removed under reduced pressure to give the free acid as the according hydrochloride salt (quant.), which was used without further purification in follow-up reactions. LCMS: R_t_ = 3.79 min (method C). MS­(ESI+) [*m*/*z*]: calculated = 544.31 [M + H]^+^; found = 544.30 [M + H]^+^.

#### **2-(4-((4-(4-(7-(4-(4-((2-Aminoethyl)­amino)-2-(1-(4-chlorophenyl)­cyclohexyl)­quinazolin-7-yl)­piperazin-1-yl)-7-oxoheptyl)­piperazin-1-yl)-6-((5-cyclopropyl-1** *H* **-pyrazol-3-yl)­amino)­pyrimidin-2-yl)­amino)­phenyl)­acetonitrile (D-1d)**

7-(4-(2-((4-(cyanomethyl)­phenyl)­amino)-6-((5-cyclopropyl-1*H*-pyrazol-3-yl)­amino)­pyrimidin-4-yl)­piperazin-1-yl)­heptanoic acid **(S-34)** (23 mg, 0.043 mmol, 1.1 equiv) and DIPEA (8 μL, 0.047 mmol, 1.2 equiv) were dissolved in 300 μL dry DMF, a solution of PyAOP (24 mg, 0.047 mmol, 1.2 equiv) in 100 μL was added and the resulting reaction mixture was stirred at room temperature for 5 min. A solution of *tert*-butyl (2-((2-(1-(4-chlorophenyl)­cyclohexyl)-7-(piperazin-1-yl)­quinazolin-4-yl)­amino)­ethyl)­carbamate **(S-16)** (22 mg, 0.039 mmol, 1.0 equiv) in 300 μL dry DMF was added and the resulting reaction mixture was then stirred at room temperature for 1.5 h. All volatiles were removed under reduced pressure. The isolated crude was loaded unto Celite and purified via reverse phase flash column chromatography using acetonitrile/water (5% → 100%) as eluent. The obtained intermediate was dissolved in 400 μL of a 25 vol % solution of trifluoroacetic acid/dichloromethane. The resulting mixture was stirred at room temperature for 30 min. All volatiles were removed under reduced pressure and the residue was coevaporated with dichloromethane twice to give the according trifluoroacetate salt. The obtained crude was purified via preparative HPLC using acetonitrile/water (5% → 100%) with 0.1% TFA additive as eluent to give the title compound as the according trifluoroacetate salt (1:2) (6 mg, 13%). ^1^H NMR (500 MHz, DMSO-*d*_6_): δ = 12.76 (bs, 1H), 9.88–9.08 (m, 3H), 8.13 (d, ^3^J = 9.4 Hz, 1H), 8.05–7.93 (m, 3H), 7.68 (d, ^3^J = 8.5 Hz, 2H), 7.49–7.43 (m, 3H), 7.40 (d, ^3^J = 8.5 Hz, 2H), 7.24 (d, ^3^J = 8.6 Hz, 2H), 7.17–7.07 (m, 1H), 5.09 (bs, 2H), 4.31–4.23 (m, 2H), 3.95 (s, 2H), 3.96–3.89 (m, 2H), 3.60–3.56 (m, 6H), 3.54–3.51 (m, 2H), 3.48–3.44 (m, 2H), 3.26–3.01 (m, 10H), 2.78–2.72 (m, 2H), 2.39–2.34 (m, 2H), 2.16–2.06 (m, 2H), 1.89–1.83 (m, 1H), 1.71–1.60 (m, 4H), 1.57–1.38 (m, 6H), 1.36–1.30 (m, 4H), 0.94–0.89 (m, 2H), 0.69–0.65 (m, 2H). ^13^C NMR (126 MHz, DMSO-*d*_6_): δ = 170.9, 162.5, 159.3, 154.3, 140.2, 131.8, 128.6, 128.4, 128.2, 125.4, 123.3, 119.5, 119.3, 116.3, 77.3, 55.6, 50.4, 49.0, 46.1, 45.9, 43.9, 41.1, 38.0, 34.1, 32.0, 28.2, 25.9, 24.9, 24.3, 23.1, 22.6, 21.8, 7.8. LC: R_t_ = 14.29 min (method E); purity = 97%. MS­(ESI+) [*m*/*z*]: calculated = 990.54 [M + H]^+^; found = 990.67 [M + H]^+^. HRMS: (method 1): calculated = 990.5493 [M + H]^+^; found = 990.5482 [M + H]^+^.

#### **Ethyl 10-(4-(2-((4-(cyanomethyl)­phenyl)­amino)-6-((5-cyclopropyl-1** *H* **-pyrazol-3-yl)­amino)­pyrimidin-4-yl)­piperazin-1-yl)­decanoate (S-33)**

The synthesis followed the general procedure of **S-32** using ethyl 10-bromodecanoate **(S-31)** (292 mg, 1.05 mmol) to give the title compound (194 mg, 60%). ^1^H NMR (500 MHz, DMSO-*d*_6_): δ = 12.58–11.70 (m, 1H), 9.65–8.66 (m, 2H), 7.79–7.58 (m, 2H), 7.28–7.13 (m, 2H), 6.24–5.25 (m, 2H), 4.04 (q, ^3^J = 7.1 Hz, 2H), 3.92 (m, 2H), 3.50–3.40 (m, 4H), 2.45–2.37 (m, 4H), 2.30–2.24 (m, 4H), 1.92–1.72 (m, 1H), 1.54–1.48 (m, 2H), 1.47–1.41 (m, 2H), 1.28–1.23 (m, 10H), 1.17 (t, ^3^J = 7.1 Hz, 3H), 0.93–0.80 (m, 2H), 0.71–0.60 (m, 2H). ^13^C NMR (126 MHz, DMSO-*d*_6_): δ = 172.9, 163.3, 160.7, 158.8, 149.2, 145.2, 141.0, 128.0, 122.3, 119.6, 118.9, 92.5, 77.2, 59.6, 58.0, 52.5, 43.9, 33.5, 28.9, 28.8, 28.6, 28.4, 26.9, 26.3, 24.5, 21.7, 14.1, 7.6. LCMS: R_t_ = 5.51 min (method C). MS (ESI+) [*m*/*z*]: calculated = 614.39 [M + H]^+^; found = 614.40 [M + H]^+^.

#### **10-(4-(2-((4-(Cyanomethyl)­phenyl)­amino)-6-((5-cyclopropyl-1** *H* **-pyrazol-3-yl)­amino)­pyrimidin-4-yl)­piperazin-1-yl)­decanoic Acid (S-35)**

The synthesis followed the general procedure of **S-34** starting from ethyl 10-(4-(2-((4-(cyanomethyl)­phenyl)­amino)-6-((5-cyclopropyl-1*H*-pyrazol-3-yl)­amino)­pyrimidin-4-yl)­piperazin-1-yl)­decanoate **(S-33)** (25 mg, 0.041 mmol). LCMS: R_t_ = 4.60 min (method C). MS­(ESI+) [*m*/*z*]: calculated = 586.35 [M + H]^+^; found = 586.35 [M + H]^+^.

#### **2-(4-((4-(4-(10-(4-(4-((2-Aminoethyl)­amino)-2-(1-(4-chlorophenyl)­cyclohexyl)­quinazolin-7-yl)­piperazin-1-yl)-10-oxodecyl)­piperazin-1-yl)-6-((5-cyclopropyl-1** *H* **-pyrazol-3-yl)­amino)­pyrimidin-2-yl)­amino)­phenyl)­acetonitrile (D-1e)**

The synthesis followed the general procedure of **D-1d** starting from 10-(4-(2-((4-(cyanomethyl)­phenyl)­amino)-6-((5-cyclopropyl-1*H*-pyrazol-3-yl)­amino)­pyrimidin-4-yl)­piperazin-1-yl)­decanoic acid **(S-35)** (24 mg, 0.040 mmol). The title compound was isolated as trifluoroacetate salt (1:2) (10 mg, 24%). ^1^H NMR (500 MHz, DMSO-*d*_6_): δ = 12.81 (bs, 1H), 10.06–9.15 (m, 3H), 8.14 (d, ^3^J = 9.5 Hz, 1H), 8.10–7.99 (m, 3H), 7.68 (d, ^3^J = 8.5 Hz, 2H), 7.51–7.47 (m, 2H), 7.46–7.43 (m, 1H), 7.42–7.38 (m, 2H), 7.25 (d, ^3^J = 8.6 Hz, 2H), 7.16–7.12 (m, 1H), 5.89 (bs, 2H), 4.30–4.24 (m, 2H), 3.95 (s, 2H), 3.96–3.91 (m, 2H), 3.63–3.58 (m, 6H), 3.54–3.51 (2H), 3.48–3.44 (m, 2H), 3.28–2.99 (m, 10H), 2.79–2.72 (m, 2H), 2.34 (t, ^3^J = 7.6 Hz, 2H), 2.17–2.07 (m, 2H), 1.90–1.84 (m, 1H), 1.69–1.59 (m, 2H), 1.56–1.37 (m, 6H), 1.32–1.27 (m, 10H), 0.95–0.89 (m, 2H), 0.72–0.65 (m, 2H). ^13^C NMR (126 MHz, DMSO-*d*_6_): δ = 171.0, 165.7, 162.4, 159.3, 154.4, 142.9, 139.9, 131.8, 128.6, 128.4, 128.3, 125.4, 123.5, 119.5, 119.4, 116.3, 102.0, 77.2, 55.6, 50.4, 48.9, 46.2, 45.9, 45.7, 43.9, 41.2, 37.9, 34.1, 32.2, 28.9, 28.8, 28.7, 28.5, 26.0, 24.9, 24.6, 23.2, 22.6, 21.8, 7.8, 7.3. LC: R_t_ = 15.85 min (method E); purity = 98%. MS­(ESI+) [*m*/*z*]: calculated = 1032.59 [M + H]^+^; found = 1032.67 [M + H]^+^. HRMS: (method 1): calculated = 1032.5962 [M + H]^+^; found = 1032.5956 [M + H]^+^.

#### ***tert*** **-Butyl 4-(4-(2-((4-(cyanomethyl)­phenyl)­amino)-6-((5-cyclopropyl-1** *H* **-pyrazol-3-yl)­amino)­pyrimidin-4-yl)­piperazin-1-yl)-4-oxobutanoate (S-37)**

*tert*-Butyl 4-(2-((4-(cyanomethyl)­phenyl)­amino)-6-((5-cyclopropyl-1*H*-pyrazol-3-yl)­amino)­pyrimidin-4-yl)­piperazine-1-carboxylate **(1inh)** (300 mg, 0.582 mmol, 1.00 equiv) was dissolved in 4.0 mL of a 25 vol % solution of trifluoroacetic acid/dichloromethane. The resulting mixture was stirred at room temperature for 30 min. All volatiles were removed under reduced pressure and the residue was coevaporated with dichloromethane twice to give the free amine as the according trifluoroacetate salt (quant.). 4-(*tert*-butoxy)-4-oxobutanoic acid **(S-36)** (122 mg, 0.98 mmol, 1.20 equiv) and DIPEA (122 μL, 0.698 mmol, 1.20 equiv) were dissolved in 0.5 mL dry DMF, a solution of PyAOP (364 mg, 0.698 mmol, 1.20 equiv) in 1.0 mL was added and the resulting mixture was stirred at room temperature for 5 min. A solution of the previously obtained free amine and DIPEA (122 μL, 0.698 mmol, 1.20 equiv) in 1.0 mL dry DMF was added and the resulting reaction mixture was stirred for 1.5 h at room temperature. All volatiles were removed under reduced pressure. The obtained crude was loaded unto Celite and purified via reverse phase flash column chromatography using acetonitrile/water (5% → 100%) as eluent first, followed by a second purification step via flash column chromatography using methanol/dichloromethane (0% → 10%) as eluent to give the title compound (226 mg, 68%). ^1^H NMR (500 MHz, DMSO-*d*_6_): δ = 12.94–11.74 (m, 1H), 10.71–9.23 (m, 2H), 7.63 (d, ^3^J = 8.4 Hz, 2H), 7.33 (d, ^3^J = 8.3 Hz, 2H), 6.12–5.38 (m, 2H), 4.00 (s, 2H), 3.67–3.55 (m, 8H), 2.59–2.55 (m, 2H), 2.45–2.42 (m, 2H), 1.93–1.88 (m, 1H), 1.39 (s, 9H), 0.99–0.93 (m, 2H), 0.74–0.70 (m, 2H). ^13^C NMR (126 MHz, DMSO-*d*_6_): δ = 171.7, 169.8, 161.7, 128.6, 120.5, 119.4, 90.6, 75.8, 43.9, 40.5, 29.9, 27.8, 27.4, 21.8, 8.1, 7.0. LCMS: R_t_ = 4.58 min (method C). MS­(ESI+) [*m*/*z*]: calculated = 572.30 [M + H]^+^; found = 572.30 [M + H]^+^.

#### **2-(4-((4-(4-(4-(4-(4-((2-Aminoethyl)­amino)-2-(1-(4-chlorophenyl)­cyclohexyl)­quinazolin-7-yl)­piperazin-1-yl)-4-oxobutanoyl)­piperazin-1-yl)-6-((5-cyclopropyl-1** *H* **-pyrazol-3-yl)­amino)­pyrimidin-2-yl)­amino)­phenyl)­acetonitrile (D-1f)**

*tert*-Butyl 4-(4-(2-((4-(cyanomethyl)­phenyl)­amino)-6-((5-cyclopropyl-1*H*-pyrazol-3-yl)­amino)­pyrimidin-4-yl)­piperazin-1-yl)-4-oxobutanoate **(S-37)** (20 mg, 0.035 mmol, 1.0 equiv) was dissolved in 1.0 mL of a 25 vol % solution of trifluoroacetic acid/dichloromethane. The resulting mixture was stirred at room temperature for 30 min. All volatiles were removed under reduced pressure and the residue was coevaporated with dichloromethane twice to give the free acid as the according trifluoroacetate salt (quant.). The obtained trifluoroacetate salt and DIPEA (12 μL, 0.070 mmol, 2.0 equiv) were dissolved in 150 μL dry DMF, a solution of PyBOP (22 mg, 0.042 mmol, 1.2 equiv) in 150 μL dry DMF was added and the resulting reaction mixture was stirred at room temperature for 5 min. A solution of *tert*-butyl (2-((2-(1-(4-chlorophenyl)­cyclohexyl)-7-(piperazin-1-yl)­quinazolin-4-yl)­amino)­ethyl)­carbamate **(S-16)** (22 mg, 0.039 mmol, 1.1 equiv) in 150 μL dry DMF was added and the resulting reaction mixture was then stirred at room temperature for 1 h. All volatiles were removed under reduced pressure. The isolated crude was dissolved in 1.0 mL of a 25 vol % solution of trifluoroacetic acid/dichloromethane. The resulting mixture was stirred at room temperature for 45 min. All volatiles were removed under reduced pressure and the residue was coevaporated with dichloromethane twice to give the according trifluoroacetate salt. The obtained crude was purified via preparative HPLC using acetonitrile/water (5% → 100%) with 0.1% TFA additive as eluent to give the title compound as the according trifluoroacetate salt. It was then loaded unto Celite and immobilized on propylsulfonic acid functionalized silica using a 50:50 mixture of acetonitrile/water and subsequently flushed down from the silica using an ammonia/methanol solution. All volatiles were then removed under reduced pressure to give the title compound as an off-white solid (6 mg, 16%). (The desalted compound exhibited particularly poor solubility across various deuterated solvents leading to poor spectral resolution. Thus, only the 1H-NMR spectrum of the corresponding trifluoroacetate salt is provided.) ^1^H NMR (600 MHz, DMSO-*d*_6_): δ = 12.83 (bs, 1H), 10.64–9.75 (m, 3H), 8.15 (d, ^3^J = 9.5 Hz, 1H), 8.12–8.06 (m, 3H), 7.62 (d, ^3^J = 8.5 Hz, 2H), 7.50–7.48 (m, 2H), 7.46 (dd, ^3^J = 9.4 Hz, ^4^J = 2.0 Hz, 1H), 7.42–7.39 (m, 2H), 7.35 (d, ^3^J = 8.6 Hz, 2H), 7.18–7.13 (m, 1H), 5.86–5.75 (m, 2H), 4.01 (s, 2H), 3.96–3.93 (m, 2H), 3.71–3.65 (m, 6H), 3.63–3.55 (m, 8H), 3.50–3.46 (m, 2H), 3.18–3.14 (m, 2H), 2.79–2.73 (m, 2H), 2.66–2.60 (m, 4H), 2.17–2.09 (m, 2H), 1.97–1.90 (m, 1H), 1.66–1.61 (m, 2H), 1.57–1.37 (m, 4H), 0.99–0.96 (m, 2H), 0.75–0.72 (m, 2H). LC: Disclaimer: The purity of this compound could not be determined via LC due to poor solubility. Purity assessment for this compound was therefore conducted via ^1^H NMR spectroscopy and estimated to be >95%. MS­(ESI+) [*m*/*z*]: calculated = 962.47 [M + H]^+^; found = 962.60 [M + H]^+^. HRMS: (method 1): calculated = 962.4816 [M + H]^+^; found = 962.4814 [M + H]^+^.

#### ***tert*** **-Butyl 3-(2-(2-((2-(2,6-dioxopiperidin-3-yl)-1,3-dioxoisoindolin-4-yl)­oxy)­ethoxy)­ethoxy)­propanoate (S-38)**

2-(2,6-Dioxopiperidin-3-yl)-4-hydroxyisoindoline-1,3-dione **(4-hydroxy thalidomide)** (50 mg, 0.18 mmol, 1.00 equiv), sodium hydrogen carbonate (31 mg, 0.37 mmol, 2.0 equiv) and sodium iodide (27 mg, 0.182 mmol, 1.00 equiv) were suspended in 1.0 mL dry DMF. A solution of *tert*-butyl 3-(2-(2-bromoethoxy)­ethoxy)­propanoate **(S-24)** (65 mg, 0.22 mmol, 1.2 equiv) in 0.5 mL dry DMF was added and the reaction mixture was stirred at 80 °C overnight. After cooling to room temperature, the reaction mixture was diluted with dichloromethane and washed with water twice, followed by brine. The organic layer was dried over MgSO_4_, filtered and all volatiles were removed under reduced pressure. The obtained crude was loaded unto Celite and purified via reverse phase flash column chromatography using acetonitrile/water (5% → 100%) as eluent to give the title compound (67 mg, 75%). ^1^H NMR (500 MHz, DMSO-*d*_6_): δ = 11.10 (s, 1H), 7.82–7.77 (m, 1H), 7.52 (d, ^3^J = 8.5 Hz, 1H), 7.45 (d, ^3^J = 7.2 Hz, 1H), 5.11–5.05 (m, 1H), 4.35–4.30 (m, 2H), 3.82–3.77 (m, 2H), 3.65–3.61 (m, 2H), 3.58 (t, ^3^J = 6.2 Hz, 2H), 3.52–3.49 (m, 2H), 2.94–2.85 (m, 1H), 2.64–2.51 (m, 2H), 2.39 (t, ^3^J = 6.2 Hz, 2H), 2.07–2.00 (m, 1H), 1.37 (s, 9H). ^13^C NMR (126 MHz, DMSO-*d*_6_): δ = 172.8, 170.4, 169.9, 166.8, 165.2, 155.8, 136.9, 133.2, 120.0, 116.3, 115.4, 79.7, 70.1, 69.7, 68.9, 68.7, 66.2, 48.8, 35.8, 31.0, 27.7, 22.0. LCMS: R_t_ = 4.72 min (method C). MS­(ESI+) [*m*/*z*]: calculated = 491.20 [M + H]^+^; found = 513.20 [M + Na]^+^.

#### **2-(4-((4-((5-Cyclopropyl-1** *H* **-pyrazol-3-yl)­amino)-6-(4-(3-(2-(2-((2-(2,6-dioxopiperidin-3-yl)-1,3-dioxoisoindolin-4-yl)­oxy)­ethoxy)­ethoxy)­propanoyl)­piperazin-1-yl)­pyrimidin-2-yl)­amino)­phenyl)­acetonitrile (C-1a)**

*tert*-Butyl 4-(2-((4-(cyanomethyl)­phenyl)­amino)-6-((5-cyclopropyl-1*H*-pyrazol-3-yl)­amino)­pyrimidin-4-yl)­piperazine-1-carboxylate **(1inh)** (50 mg, 0.097 μmol, 1.0 equiv) and *tert*-butyl 3-(2-(2-((2-(2,6-dioxopiperidin-3-yl)-1,3-dioxoisoindolin-4-yl)­oxy)­ethoxy)­ethoxy)­propanoate **(S-38)** (62 mg, 0.13 mmol, 1.3 equiv) were dissolved in 1.0 mL of a 25 vol % solution trifluoroacetic acid/dichloromethane, respectively. The resulting mixtures were stirred at room temperature for 20 min. All volatiles were then removed under reduced pressure and the residues were coevaporated with dichloromethane twice to give the free amine and the free acid as the corresponding trifluoroacetate salts, respectively (quant.). The free acid and DIPEA (24 μL, 0.14 mmol, 1.4 equiv) were then then dissolved in 0.5 dry DMF and HATU (52 mg, 0.14 μmol, 1.4 equiv) was added. The resulting reaction mixture was stirred for 15 min at room temperature, upon which a solution of the free amine and DIPEA (24 μL, 0.14 μmol, 1.4 equiv) in 1.0 mL dry DMF was added. The reaction mixture was stirred at room temperature for 1 h. All volatiles were then removed under reduced pressure. The obtained crude was loaded unto Celite and purified via flash column chromatography using methanol/dichloromethane (0% → 10%) as eluent first, followed by a second purification step via flash column chromatography using methanol/dichloromethane (0% → 5%) as eluent to give the title compound (53 mg, 65%). ^1^H NMR (500 MHz, DMSO-*d*_6_): δ = 13.00–11.51 (m, 1H), 11.10 (s, 1H), 10.40–10.44 (m, 2H), 7.78–7.74 (m, 1H), 7.62 (d, ^3^J = 8.4 Hz, 2H), 7.48 (d, ^3^J = 8.5 Hz, 1H), 7.42 (d, ^3^J = 7.2 Hz, 1H), 7.33 (d, ^3^J = 8.4 Hz, 2H), 6.06–5.47 (m, 2H), 5.07 (dd, ^3^J = 5.4 Hz, 12.8 Hz, 1H), 4.32–4.29 (m, 2H), 3.99 (s, 2H), 3.80–3.78 (m, 2H), 3.66- −3.64 (m, 4H), 3.62–3.52 (m, 6H), 3.55–3.51 (m, 4H), 2.93–2.83 (m, 1H), 2.65–2.57 (m, 1H), 2.61 (t, ^3^J = 6.5 Hz, 2H), 2.57–2.51 (m, 1H), 2.03 1.98 (m, 1H), 1.94–1.88 (m, 1H), 1.25 (t, ^3^J = 6.8 Hz, 2H), 0.98–0.94 (m, 2H), 0.74–0.70 (m, 2H). ^13^C NMR (126 MHz, DMSO-*d*_6_): δ = 172.8, 170.0, 169.2, 166.8, 165.3, 161.6, 155.8, 148.2, 147.2, 138.3, 137.0, 133.2, 128.6, 125.3, 120.5, 119.9, 119.4, 116.3, 115.4, 90.7, 75.7, 70.2, 69.8, 68.9, 68.7, 66.8, 53.6, 48.8, 44.3, 44.1, 43.9, 40.5, 32.9, 31.0, 22.0, 21.9, 18.1, 16.7, 12.5, 8.1, 7.0. LCMS: R_t_ = 4.11 min (method C); purity = 96%. MS­(ESI+) [*m*/*z*]: calculated = 832.35 [M + H]^+^; found = 832.35 [M + H]^+^. HRMS: (method 1): calculated = 832.35312 [M + H]^+^; found = 832.35166 [M + H]^+^.

#### ***tert*** **-Butyl 3-(2-(2-(2-((2-(2,6-dioxopiperidin-3-yl)-1,3-dioxoisoindolin-4-yl)­oxy)­acetamido)­ethoxy)­ethoxy)­propanoate (S-40)**

A solution of *tert*-butyl 2-((2-(2,6-dioxopiperidin-3-yl)-1,3-dioxoisoindolin-4-yl)­oxy)­acetate **(S-23)** (250 mg, 0.752 mmol, 1.00 equiv) was dissolved in 1.0 mL of a 25 vol % solution trifluoroacetic acid/dichloromethane. The resulting mixture was stirred at room temperature for 30 min. All volatiles were then removed under reduced pressure and the residue was coevaporated with dichloromethane twice to give the free acid as the corresponding trifluoroacetate salt (quant.). The trifluoroacetate salt was then dissolved in 2.0 mL dry DMF and DIPEA was added (184 μL, 1.05 mmol, 1.40 equiv) followed by HATU (401 mg, 1.05 mmol, 1.40 equiv) and the resulting mixture was stirred at room temperature for 30 min. A solution of *tert*-butyl 3-(2-(2-aminoethoxy)­ethoxy)­propanoate **(S-39)** (193 mg, 0.828 mmol, 1.10 equiv) in 1.0 mL dry DMF was added to the reaction mixture and the resulting mixture was stirred at room temperature overnight. The reaction mixture was quenched with water and then washed with 10 vol % acetic acid, followed by saturated aqueous NaHCO_3_ solution and water. The combined organic layers were dried over MgSO_4_, filtered and all volatiles were removed under reduced pressure. The obtained crude was loaded unto Celite and purified via reverse phase flash column chromatography using acetonitrile/water (5% → 100%) as eluent to give the title compound (230 mg, 56%). ^1^H NMR (500 MHz, DMSO-*d*_6_): δ = 11.11 (s, 1H), 8.24 (t, ^3^J = 5.7 Hz, 1H), 7.86 (d, ^3^J = 8.3 Hz, 2H), 7.43 (d, ^4^J = 2.2 Hz, 1H), 7.38 (dd, ^3^J = 8.3 Hz, ^4^J = 2.3 Hz), 5.12 (dd, ^3^J = 5.4 Hz, 12.8 Hz, 1H), 4.73 (s, 2H), 3.58 (t, ^3^J = 6.2 Hz, 2H), 3.50–3.47 (m, 4H), 3.45 (t, ^3^J = 5.9 Hz, 2H), 3.31–3.27 (m, 2H), 2.93–2.84 (m, 1H), 2.64–2.51 (m, 2H), 2.40 (t, ^3^J = 6.2 Hz, 2H), 2.08–2.02 (m, 1H), 1.38 (s, 9H). ^13^C NMR (126 MHz, DMSO-*d*_6_): δ = 172.8, 170.4, 169.92, 166.90, 166.81, 166.75, 163.1, 133.7, 125.3, 123.6, 121.0, 109.4, 79.8, 69.6, 69.5, 68.8, 67.3, 66.2, 49.0, 38.4, 35.8, 31.0, 27.8, 22.1. LCMS: R_t_ = 4.23 min (method C). MS­(ESI+) [*m*/*z*]: calculated = 548.22 [M + H]^+^; found = 570.20 [M + Na]^+^.

#### ***N*** **-(2-(2-(3-(4-(2-((4-(Cyanomethyl)­phenyl)­amino)-6-((5-cyclopropyl-1** *H* **-pyrazol-3-yl)­amino)­pyrimidin-4-yl)­piperazin-1-yl)-3-oxopropoxy)­ethoxy)­ethyl)-2-((2-(2,6-dioxopiperidin-3-yl)-1,3-dioxoisoindolin-4-yl)­oxy)­acetamide (C-1b)**

*tert*-Butyl 3-(2-(2-(2-((2-(2,6-dioxopiperidin-3-yl)-1,3-dioxoisoindolin-4-yl)­oxy)­acetamido)­ethoxy)­ethoxy)­propanoate **(S-40)** (30 mg, 0.055 mmol, 1.0 equiv) and t*ert*-butyl 4-(2-((4-(cyanomethyl)­phenyl)­amino)-6-((5-cyclopropyl-1*H*-pyrazol-3-yl)­amino)­pyrimidin-4-yl)­piperazine-1-carboxylate **(1inh)** (31 mg, 0.060 mmol, 1.1 equiv) were dissolved in 2.0 mL of a 25 vol % solution trifluoroacetic acid/dichloromethane, respectively. The resulting mixtures were stirred at room temperature for 45 min. All volatiles were then removed under reduced pressure and the residues were coevaporated with dichloromethane twice to give the free amine and the free acid as the corresponding trifluoroacetate salts, respectively (quant.). The free acid and DIPEA (13 μL, 0.077 mmol, 1.4 equiv) were dissolved in 1.0 dry DMF and HATU (29 mg, 0.077 mmol, 1.4 equiv) was added. The resulting reaction mixture was stirred for 25 min at room temperature, upon which a solution of the free amine and DIPEA (13 μL, 0.077 mmol, 1.4 equiv) in 1.5 mL dry DMF was added. The reaction mixture was then stirred at room temperature overnight. The reaction mixture was diluted with ethyl acetate and washed with water. The combined organic layers were dried over MgSO_4_, filtered and all volatiles were removed under reduced pressure. The obtained crude was loaded unto Celite and purified via reverse phase flash column chromatography using acetonitrile/water (5% → 100%) as eluent to give the title compound (11 mg, 23%). ^1^H NMR (500 MHz, DMSO-*d*_6_): δ = 12.48–11.73 (m, 1H), 11.10 (s, 1H), 9.74–8.58 (m, 2H), 8.24 (t, ^3^J = 5.7 Hz, 1H), 7.85 (d, ^3^J = 8.3 Hz, 1H), 7.79–7.54 (m, 2H), 7.43 (d, ^4^J = 2.2 Hz, 1H), 7.37 (dd, ^3^J = 8.3 Hz, ^4^J = 2.3 Hz, 1H), 7.31–7.15 (m, 2H), 6.30–5.80 (m, 1H), 5.14–5.09 (m, 1H), 4.73 (s, 2H), 3.93 (s, 2H), 3.65 (t, ^3^J = 6.6 Hz, 2H), 3.60–3.51 (m, 6H), 3.50 (bs, 4H), 3.49–3.42 (m, 4H), 3.31–3.26 (m, 2H), 2.93–2.83 (m, 1H), 2.64–2.56 (m, 3H), 2.53–2.51 (s, 1H), 2.07–2.01 (m, 1H), 1.89–1.73 (m, 1H), 0.93–0.82 (m, 2H), 0.69–0.61 (m, 2H). ^13^C NMR (126 MHz, DMSO-*d*_6_): δ = 172.7, 169.9, 169.0, 166.9, 166.8, 166.7, 163.1, 161.0, 158.6, 140.8, 133.7, 128.7, 128.1, 125.3, 123.5, 122.5, 120.9, 120.5, 119.6, 118.9, 109.4, 77.1, 69.60, 69.56, 68.8, 67.3, 66.7, 49.0, 44.4, 43.9, 43.6, 38.4, 32.9, 30.9, 22.0, 21.7, 7.7. LCMS: R_t_ = 3.62 min (method C); purity = 97%. MS­(ESI+) [*m*/*z*]: calculated = 889.37 [M + H]^+^; found = 889.45 [M + H]^+^.HRMS: (method 1): calculated = 889.37400 [M + H]^+^; found = 889.37470 [M + H]^+^.

#### ***tert*** **-Butyl (2-(2-(2-(2-(4-(2-((4-(cyanomethyl)­phenyl)­amino)-6-((5-cyclopropyl-1** *H* **-pyrazol-3-yl)­amino)­pyrimidin-4-yl)­piperazin-1-yl)­ethoxy)­ethoxy)­ethoxy)­ethyl)­carbamate (S-42)**

The synthesis followed the general procedure of **S-32** using *tert*-butyl (2-(2-(2-(2-bromoethoxy)­ethoxy)­ethoxy)­ethyl)­carbamate **(S-41)** (69 mg, 0.19 mmol) to give the title compound (53 mg, 79%). ^1^H NMR (500 MHz, DMSO-*d*_6_): δ = 12.57–11.68 (m, 1H), 9.96–8.44 (m, 2H), 7.72 (t, ^3^J = 7.5 Hz, 2H), 7.21 (d, ^3^J = 8.4 Hz, 2H), 6.73 (t, ^3^J = 5.4 Hz, 1H), 6.26–5.18 (m, 2H), 3.92 (s, 2H), 3.55 (t, ^3^J = 5.9 Hz, 2H), 3.52–3.49 (m, 8H), 3.48–3.44 (m, 4H), 3.38 (t, ^3^J = 6.2 Hz, 2H), 3.09–3.04 (m, 2H), 1.86–1.81 (m, 1H), 1.37 (s, 9H), 0.92–0.85 (m, 2H), 0.68–0.64 (m, 2H). ^13^C NMR (126 MHz, DMSO-*d*_6_): δ = 163.3, 160.5, 158.7, 155.6, 140.9, 128.1, 122.5, 119.6, 118.8, 77.6, 77.0, 69.78, 69.76, 69.7, 69.5, 69.2, 68.3, 57.3, 52.8, 44.0, 28.5, 28.2, 21.8, 7.7. LCMS: R_t_ = 3.93 min (method C). MS­(ESI+) [*m*/*z*]: calculated = 691.40 [M + H]^+^; found = 691.40 [M + H]^+^.

#### ***N*** **-(2-(2-(2-(2-(4-(2-((4-(Cyanomethyl)­phenyl)­amino)-6-((5-cyclopropyl-1** *H* **-pyrazol-3-yl)­amino)­pyrimidin-4-yl)­piperazin-1-yl)­ethoxy)­ethoxy)­ethoxy)­ethyl)-2-((2-(2,6-dioxo-piperidin-3-yl)-1,3-dioxoisoindolin-4-yl)­oxy)­acetamide (C-1c)**

*tert*-Butyl (2-(2-(2-(2-(4-(2-((4-(cyanomethyl)­phenyl)­amino)-6-((5-cyclopropyl-1*H*-pyrazol-3-yl)­amino)­pyrimidin-4-yl)­piperazin-1-yl)­ethoxy)­ethoxy)­ethoxy)­ethyl)­carbamate **(S-42)** (60 mg, 0.087 mmol, 1.0 equiv) and *tert*-butyl 2-((2-(2,6-dioxopiperidin-3-yl)-1,3-dioxoisoindolin-4-yl)­oxy)­acetate **(S-23)** (44 mg, 0.11 mmol, 1.3 equiv) were dissolved in 1.0 mL of a 25 vol % solution trifluoroacetic acid/dichloromethane, respectively. The resulting mixtures were stirred at room temperature for 40 min. All volatiles were then removed under reduced pressure and the residues were coevaporated with dichloromethane twice to give the free amine and the free acid as the corresponding trifluoroacetate salts, respectively (quant.). The free acid and DIPEA (21 μL, 0.12 mmol, 1.4 equiv) were then dissolved in 0.5 dry DMF and HATU (46 mg, 0.12 mmol, 1.4 equiv) was added. The resulting reaction mixture was stirred for 10 min at room temperature, upon which a solution of the free amine and DIPEA (21 μL, 0.12 mmol, 1.4 equiv) in 1.0 mL dry DMF was added. The reaction mixture was then stirred at room temperature for 1.5 h. All volatiles were removed under reduced pressure. The obtained crude was loaded unto Celite and purified via reverse phase flash column chromatography using acetonitrile/water (5% → 100%) as eluent to give the title compound (46 mg, 59%). ^1^H NMR (500 MHz, DMSO-*d*_6_): δ = 12.71–11.66 (m, 1H), 11.12 (s, 1H), 10.13–8.63 (m, 2H), 7.98 (t, ^3^J = 5.4 Hz, 1H), 7.80 (t, ^3^J = 5.0 Hz, 1H), 7.73–7.56 (m, 2H), 7.50 (d, ^3^J = 7.2 Hz, 1H), 7.39 (d, ^3^J = 8.6 Hz, 1H), 7.25–7.10 (m, 2H), 6.31–5.45 (m, 2H), 5.14–5.09 (m, 1H), 4.78 (s, 2H), 3.93 (s, 2H), 3.75–3.52 (m, 12H), 3.49–3.46 (m, 2H), 3.42–3.28 (m, 8H), 2.93–2.86 (m, 1H), 2.64–2.52 (m, 2H), 2.08–2.01 (m, 1H), 1.87–1.81 (m, 1H), 0.93–0.85 (m, 2H), 0.69–0.64 (m, 2H). ^13^C NMR (126 MHz, DMSO-*d*_6_): δ = 172.8, 169.9, 167.0, 166.7, 165.5, 162.8, 160.8, 158.7, 154.9, 140.6, 137.0, 133.0, 128.2, 122.8, 120.4, 119.6, 119.0, 116.8, 116.1, 77.3, 69.72, 69.66, 69.6, 68.8, 67.6, 55.5, 51.2, 50.0, 48.9, 42.4, 41.6, 31.0, 28.6, 22.0, 21.8, 7.7. LCMS: R_t_ = 3.77 min (method C); purity = 96%. MS­(ESI+) [*m*/*z*]: calculated = 905.40 [M + H]^+^; found = 905.35 [M + H]^+^. HRMS: (method 1): calculated = 905.40585 [M + H]^+^; found = 905.40548 [M + H]^+^.

#### ***tert*** **-Butyl (7-(4-(2-((4-(cyanomethyl)­phenyl)­amino)-6-((5-cyclopropyl-1** *H* **-pyrazol-3-yl)­amino)­pyrimidin-4-yl)­piperazin-1-yl)-7-oxoheptyl)­carbamate (S-44)**

The synthesis followed the general procedure of **S-37** using 7-((t*ert*-butoxycarbonyl)­amino)­heptanoic acid **(S-43)** (186 mg, 0.756 mmol) and HATU (310 mg, 0.815 mmol, 1.40 equiv) to give the title compound (211 mg, 56%). ^1^H NMR (500 MHz, DMSO-*d*_6_): δ = 12.46–11.80 (m, 1H), 9.75–8.69 (m, 2H), 7.82–7.57 (m, 2H), 7.40–7.09 (m, 2H), 6.74 (t, ^3^J = 5.3 Hz, 1H), 6.24–5.17 (m, 2H), 3.93 (s, 2H), 3.58–3.44 (m, 8H), 2.92–2.87 (m, 2H), 2.34 (t, ^3^J = 7.5 Hz, 2H), 1.90–1.76 (m, 1H), 1.54–1.46 (m, 2H), 1.38–1.34 (m, 2H), 1.36 (s, 9H), 1.29–1.22 (m, 4H), 0.98–0.80 (m, 2H), 0.72–0.61 (m, 2H). ^13^C NMR (126 MHz, DMSO-*d*_6_): δ = 170.9, 170.0, 163.1, 160.7, 158.7, 155.6, 149.1, 145.3, 140.8, 128.1, 122.5, 119.6, 119.0, 92.5, 77.3, 50.0, 44.4, 44.0, 43.7, 40.6, 32.3, 29.4, 28.54, 28.49, 28.3, 26.1, 24.7, 21.8, 7.7. LCMS: R_t_ = 4.70 min (method C). MS­(ESI+) [*m*/*z*]: calculated = 643.28 [M + H]^+^; found = 643.40 [M + H]^+^.

#### ***N*** **-(7-(4-(2-((4-(Cyanomethyl)­phenyl)­amino)-6-((5-cyclopropyl-1** *H* **-pyrazol-3-yl)­amino)-pyrimidin-4-yl)­piperazin-1-yl)-7-oxoheptyl)-2-((2-(2,6-dioxopiperidin-3-yl)-1,3-dioxo-isoindolin-4-yl)­oxy)­acetamide (C-1d)**

*tert*-Butyl (7-(4-(2-((4-(cyanomethyl)­phenyl)­amino)-6-((5-cyclopropyl-1*H*-pyrazol-3-yl)­amino)­pyrimidin-4-yl)­piperazin-1-yl)-7-oxoheptyl)­carbamate **(S-44)** (35 mg, 0.055 mmol, 1.0 equiv) and *tert*-butyl 2-((2-(2,6-dioxopiperidin-3-yl)-1,3-dioxoisoindolin-4-yl)­oxy)­acetate **(S-23)** (28 mg, 0.071 mmol, 1.3 equiv) were dissolved in 1.0 mL of a 25 vol % solution trifluoroacetic acid/dichloromethane, respectively. The resulting mixtures were stirred at room temperature for 25 min. All volatiles were then removed under reduced pressure and the residues were coevaporated with dichloromethane twice to give the free amine and the free acid as the corresponding trifluoroacetate salts, respectively (quant.). The free acid and DIPEA (13 μL, 0.076 mmol, 1.4 equiv) were then dissolved in 0.5 dry DMF and HATU (29 mg, 0.076 mmol, 1.4 equiv) was added. The resulting reaction mixture was stirred for 10 min at room temperature, upon which a solution of the free amine and DIPEA (13 μL, 0.076 mmol, 1.4 equiv) in 1.0 mL dry DMF was added. The reaction mixture was then stirred at room temperature for 1.5 h. All volatiles were removed under reduced pressure. The obtained crude was loaded unto Celite and purified via reverse phase flash column chromatography using acetonitrile/water (5% → 100%) as eluent to give the title compound (20 mg, 43%). ^1^H NMR (500 MHz, DMSO-*d*_6_): δ = 12.76–11.68 (m, 1H), 11.11 (s, 1H), 10.07–8.57 (m, 2H), 7.92 (t, ^3^J = 5.5 Hz, 1H), 7.83–7.79 (m, 1H), 7.76–7.56 (m, 2H), 7.49 (d, ^3^J = 7.3 Hz, 1H), 7.40 (d, ^3^J = 8.5 Hz, 1H), 7.30–7.09 (m, 2H), 7.57–5.24 (m, 2H), 5.14–5.09 (m, 1H), 4.77 (s, 2H), 3.93 (s, 2H), 3.58–3.50 (m, 6H), 3.49–3.44 (m, 2H), 3.18–3.12 (m, 2H), 2.93–2.85 (m, 1H), 2.64–2.52 (m, 2H), 2.34 (t, ^3^J = 7.4 Hz, 2H), 2.07–2.01 (m, 1H), 1.86–1.79 (m, 1H), 1.53–1.41 (m, 4H), 1.32–1.27 (m, 4H), 0.92–0.84 (m, 2H), 0.69–0.63 (m, 2H). ^13^C NMR (126 MHz, DMSO-*d*_6_): δ = 172.8, 170.9, 169.9, 166.7, 166.6, 165.5, 163.1, 160.6, 158.7, 155.0, 140.8, 136.9, 133.0, 128.1, 122.5, 120.4, 119.6, 118.9, 116.8, 116.1, 77.1, 67.7, 50.0, 48.8, 44.4, 44.0, 43.7, 40.6, 38.3, 32.3, 31.0, 28.9, 28.54, 28.48, 26.2, 24.7, 22.0, 21.8, 7.7. LCMS: R_t_ = 4.20 min (method C); purity = 91%. MS­(ESI+) [*m*/*z*]: calculated = 857.38 [M + H]^+^; found = 857.40 [M + H]^+^. HRMS: (method 1): calculated = 857.38472 [M + H]^+^; found = 857.38359 [M + H]^+^.

#### 2-((2-((4-(4-(2-(2-(3-(4-(4-((2-Aminoethyl)­amino)-2-(1-(4-chlorophenyl)­cyclohexyl)-quinazolin-7-yl)­piperazin-1-yl)-3-oxopropoxy)­ethoxy)­ethyl)­piperazin-1-yl)­phenyl)-amino)-5-chloropyrimidin-4-yl)­amino)­benzamide (D-2a)

A solution of *tert*-butyl 3-(2-(2-(4-(4-((4-((2-carbamoylphenyl)­amino)-5-chloropyrimidin-2-yl)­amino)­phenyl)­piperazin-1-yl)­ethoxy)­ethoxy)­propanoate **(S-45)** (18 mg, 31 μmol, 1.0 equiv) in DCM/TFA (1/1, 4 mL) was stirred 2 h. All volatiles were removed under reduced pressure. The residue was dissolved with DCM and the solvent was removed under reduced pressure (2x). *Tert*-butyl (2-((2-(1-(4-chlorophenyl)­cyclohexyl)-7-(piperazin-1-yl)­quinazolin-4-yl)­amino)­ethyl)­carbamate **(S-16)** (19 mg, 33 μmol, 1.1 equiv) was added to a solution of the crude acid, DIPEA (21 μL, 119 μmol, 4.0 equiv) and PyBOP (19 mg, 36 μmol, 1.2 equiv) in DMF/THF (2 mL, 1/1). The reaction mixture was stirred overnight at 50 °C. All volatiles were removed under reduced pressure and the residue was stirred with DCM/TFA (1/1, 4 mL) for 2 h. All volatiles were removed under reduced pressure and the crude product was purified using preparative HPLC (ACN/H_2_O/TFA). TFA was removed by loading the sample on propylsulfonic acid-modified silica, purging with water, acetonitrile and methanol and mobilizing with ammonia in methanol. The title compound was isolated as a yellow solid (16 mg, 52%). ^1^H NMR (500 MHz, MeOD*-d*_4_): δ = 8.73 (d, ^3^ *J* = 8.4 Hz, 1H), 7.99 (s, 1H), 7.83 (d, ^3^ *J* = 9.1 Hz, 1H), 7.76 (dd, ^3^ *J* = 7.9 Hz, ^4^ *J* = 1.4 Hz, 1H), 7.45–7.42 (m, 2H), 7.42–7.37 (m, 3H), 7.24–7.18 (m, 2H), 7.15 (dd, ^3^ *J =* 9.2 Hz, ^4^ *J* = 2.4 Hz, 1H), 7.12–7.07 (m, 2H), 6.89–6.85 (m, 2H), 3.80 (t, ^3^ *J* = 6.1 Hz, 2H), 3.78–3.74 (m, 4H), 3.66–3.60 (m, 8H), 3.41–3.38 (m, 2H), 3.33–3.30 (m, 2H), 3.06–3.02 (m, 4H), 2.89 (t, ^3^ *J* = 6.2 Hz, 2H), 2.85 (d, ^3^ *J* = 12.9 Hz, 2H), 2.73 (t, ^3^ *J* = 6.1 Hz, 2H), 2.58–2.52 (m, 6H), 2.02 (t, ^3^ *J* = 10.4 Hz, 2H), 1.69–1.50 (m, 6H). Contains traces of MeOH. ^13^C NMR (126 MHz, MeOD*-d*_4_): δ = 173.8, 172.4, 171.1, 161.2, 159.9, 157.0, 155.2, 153.1, 148.7, 148.5, 141.3, 134.0, 133.2, 132.4, 129.5, 129.4, 128.9, 124.1, 123.8, 123.09, 123.06, 121.4, 117.9, 117.2, 109.7, 107.4, 106.4, 71.6, 71.5, 69.5, 68.7, 58.6, 54.4, 51.2, 50.8, 46.9, 44.0, 42.7, 42.1, 37.1, 34.5, 27.2, 24.7. LC:R_t_ = 11.70 min (method F); purity = > 99%. MS: MS­(ESI+) [*m*/*z*]: calculated = 1030.48 [M + H]^+^; found = 1030.55 [M + H]^+^. HRMS: (method 1): calculated = 1030.4732 [M + H]^+^; found = 1030.4731 [M + H]^+^.

#### ***tert*** **-Butyl 3-(2-(2-(2-(4-(4-((4-((2-carbamoylphenyl)­amino)-5-chloropyrimidin-2-yl)-amino)­phenyl)­piperazin-1-yl)­ethoxy)­ethoxy)­ethoxy)­propanoate (S-46)**

A solution of *tert*-butyl 4-(4-((4-((2-carbamoylphenyl)­amino)-5-chloropyrimidin-2-yl)­amino)­phenyl)­piperazine-1-carboxylate **(S-12)** (150 mg, 286 μmol, 1.0 equiv) in DCM/TFA (6 mL, 1/1) was stirred for 3.5 h and evaporated under reduced pressure. The residue was suspended in DCM and evaporated two times. A mixture of the amine, *tert*-butyl 3-(2-(2-(2-bromoethoxy)­ethoxy)­ethoxy)­propanoate (147 mg, 429 μmol, 1.5 equiv) and Cs_2_CO_3_ (280 mg, 859 μmol, 3.0 equiv) in DMF (2.5 mL) was stirred at 80 °C overnight. All volatiles were removed under reduced pressure and the crude material was purified by reversed phase flash column chromatography (H_2_O/ACN). The title compound was isolated as a brown solid (151 mg, 77%). ^1^H NMR (500 MHz, DMSO*-d*_6_): δ = 11.84 (s, 1H), 9.19 (s, 1H), 8.80 (s, 1H), 8.28 (s, 1H), 8.15 (s, 1H), 7.81 (dd, ^3^ *J* = 7.9 Hz, ^3^ *J* = 1.5 Hz, 1H), 7.72 (s, 1H), 7.46 (t, ^3^ *J* = 6.3 Hz, 3H), 7.13–7.08 (m, 1H), 6.88 (d, ^3^ *J* = 9.1 Hz, 2H), 3.59 (t, ^3^ *J* = 6.2 Hz, 2H), 3.54 (t, ^3^ *J* = 5.9 Hz, 2H), 3.53–3.47 (m, 8H), 3.08–3.04 (m, 4H), 2.59–2.55 (m, 4H), 2.52 (t, ^3^ *J* = 5.9 Hz, 2H), 2.41 (t, ^3^ *J* = 6.2 Hz, 2H), 1.39 (s, 9H). ^13^C NMR (126 MHz, DMSO*-d*_6_): δ = 171.03, 170.39, 158.06, 154.94, 154.61, 146.63, 140.02, 132.10, 131.79, 128.55, 121.56, 121.38, 121.12, 119.70, 115.67, 104.29, 79.71, 69.77, 69.72, 69.69, 68.41, 66.24, 57.26, 53.19, 48.99, 35.86, 27.76. LCMS: R_t_ = 3.42 min (method A). MS­(ESI+) [*m*/*z*]: calculated = 684.4 [M + H]^+^; found = 684.3 [M + H]^+^.

#### 2-((2-((4-(4-(2-(2-(2-(3-(4-(4-((2-Aminoethyl)­amino)-2-(1-(4-chlorophenyl)­cyclohexyl)-quinazolin-7-yl)­piperazin-1-yl)-3-oxopropoxy)­ethoxy)­ethoxy)­ethyl)­piperazin-1-yl)­phenyl)­amino)-5-chloropyrimidin-4-yl)­amino)­benzamide (D-2b)

The synthesis followed the general procedure of **D-2a** starting from *tert*-butyl 3-(2-(2-(2-(4-(4-((4-((2-carbamoylphenyl)­amino)-5-chloropyrimidin-2-yl)­amino)­phenyl)­piperazin-1-yl)­ethoxy)­ethoxy)­ethoxy)­propanoate **(S-46)** (19 mg, 28 μmol). The title compound was isolated as a yellow solid (12 mg, 40%). ^1^H NMR (500 MHz, MeOD*-d*_4_): δ = 8.73 (d, ^3^ *J* = 8.5 Hz, 1H), 7.96 (s, 1H), 7.82 (d, ^3^ *J* = 9.1 Hz, 1H), 7.77 (dd, ^3^ *J* = 7.9 Hz, ^4^ *J* = 1.4 Hz, 1H), 7.47–7.38 (m, 5H), 7.24–7.20 (m, 2H), 7.14 (dd, ^3^ *J* = 9.1 Hz, ^4^ *J* = 2.5 Hz, 1H), 7.12–7.07 (m, 2H), 6.93–6.89 (m, 2H), 3.80 (t, ^3^ *J* = 6.2 Hz, 2H), 3.74–3.66 (m, 6H), 3.65–3.56 (m, 10H), 3.37–3.35 (m, 2H), 3.32–3.29 (m, 2H), 3.13–3.08 (m, 4H), 2.94 (t, ^3^ *J* = 6.1 Hz, 2H), 2.88–2.82 (m, 2H), 2.71 (t, ^3^ *J* = 6.2 Hz, 2H), 2.67–2.62 (m, 4H), 2.58 (t, ^3^ *J* = 5.5 Hz, 2H), 2.03 (t, ^3^ *J* = 10.3 Hz, 2H), 1.69–1.52 (m, 6H). Contains traces of MeOH. ^13^C NMR (126 MHz, MeOD*-d*_4_): δ = 173.8, 172.3, 171.1, 161.2, 159.9, 157.0, 155.1, 153.0, 148.7, 148.5, 141.3, 134.1, 133.2, 132.4, 129.4, 128.9, 124.0, 123.8, 123.1, 121.5, 117.9, 117.2, 109.8, 107.4, 106.4, 71.63, 71.60, 71.5, 71.4, 69.5, 68.6, 58.7, 54.5, 51.2, 50.8, 46.7, 43.6, 42.7, 42.0, 37.1, 34.5, 27.2, 24.7. LC:R_t_ = 11.70 min (method F); purity = > 99%. MS: MS­(ESI+) [*m*/*z*]: calculated = 1074.50 [M + H]^+^; found = 1074.57 [M + H]^+^. HRMS: (method 2): calculated = 1074.4994 [M + H]^+^; found = 1074.5022 [M + H]^+^.

#### 2-((2-((4-(4-(15-(4-(4-((2-Aminoethyl)­amino)-2-(1-(4-chlorophenyl)­cyclohexyl)­quinazolin-7-yl)­piperazin-1-yl)-15-oxo-3,6,9,12-tetraoxapentadecyl)­piperazin-1-yl)­phenyl)­amino)-5-chloropyrimidin-4-yl)­amino)­benzamide (D-2c)

The synthesis followed the general procedure of **D-2a** starting from *tert*-butyl 1-(4-(4-((4-((2-carbamoylphenyl)­amino)-5-chloropyrimidin-2-yl)­amino)­phenyl)­piperazin-1-yl)-3,6,9,12-tetraoxapentadecan-15-oate **(S-47)** (20 mg, 27 μmol). The title compound was isolated as a yellow solid (9 mg, 32%). ^1^H NMR (500 MHz, MeOD*-d*_4_): δ = 8.62 (d, ^3^ *J* = 8.5 Hz, 1H), 7.86 (s, 1H), 7.72 (d, ^3^ *J* = 9.1 Hz, 1H), 7.64 (dd, ^3^ *J* = 7.9 Hz, ^4^ *J* = 1.4 Hz, 1H), 7.34–7.26 (m, 5H), 7.12–7.08 (m, 2H), 7.04 (dd, ^3^ *J* = 9.2 Hz, ^4^ *J* = 2.5 Hz, 1H), 6.99–6.95 (m, 2H), 6.84–6.80 (m, 2H), 3.67–3.56 (m, 8H), 3.54–3.42 (m, 16H), 3.27–3.24 (m, 2H), 3.05–3.00 (m, 4H), 2.84 (t, ^3^ *J* = 6.1 Hz, 2H), 2.77–2.70 (m, 2H), 2.59–2.54 (m, 6H), 2.50 (t, ^3^ *J* = 5.5 Hz, 2H), 1.90 (t, ^3^ *J* = 10.5 Hz, 2H), 1.57–1.40 (m, 6H). Contains traces of MeOH. ^13^C NMR (126 MHz, MeOD*-d*_4_): δ = 173.8, 172.3, 171.0, 161.2, 159.9, 157.0, 155.2, 153.0, 148.7, 141.3, 134.1, 133.2, 132.4, 129.5, 129.4, 129.0, 124.0, 123.8, 123.1, 121.5, 117.9, 117.3, 109.8, 107.4, 106.4, 71.62, 71.60, 71.59, 71.57, 71.4, 69.7, 68.6, 58.7, 54.6, 51.2, 50.8, 46.7, 43.3, 42.6, 42.0, 37.1, 34.5, 27.2, 24.7. LC: R_t_ = 11.46 min (method F); purity = > 99%. MS: MS­(ESI+) [*m*/*z*]: calculated = 1118.53 [M + H]^+^; found = 1119.92 [M + H]^+^. HRMS: (method 1): calculated = 1118.5266 [M + H]^+^; found = 1118.5256 [M + H]^+^.

#### Ethyl 5-(4-(4-((4-((2-carbamoylphenyl)­amino)-5-chloropyrimidin-2-yl)­amino)­phenyl)-piperazin-1-yl)­pentanoate (S-48)

The synthesis followed the general procedure of **S-46** using ethyl 5-bromopentanoate (91 mg, 435 μmol). The title compound was isolated as a beige solid (127 mg, 79%). ^1^H NMR (500 MHz, DMSO-*d*_6_): δ = 11.84 (s, 1H), 9.19 (s, 1H), 8.80 (s, 1H), 8.28 (s, 1H), 8.15 (s, 1H), 7.81 (dd, ^3^ *J* = 7.9 Hz, ^4^ *J* = 1.4 Hz, 1H), 7.72 (s, 1H), 7.46 (d, ^3^ *J* = 8.9 Hz, 3H), 7.14–7.07 (m, 1H), 6.88 (d, ^3^ *J* = 9.1 Hz, 2H), 4.05 (q, ^3^ *J* = 7.1 Hz, 2H), 3.09–3.04 (m, 4H), 2.49–2.46 (m, 4H), 2.33–2.29 (m, 4H), 1.60–1.52 (m, 2H), 1.50–1.43 (m, 2H), 1.18 (t, ^3^ *J* = 7.1 Hz, 3H). ^13^C NMR (126 MHz, DMSO-*d*_6_): δ = 172.89, 171.03, 158.06, 154.95, 154.60, 146.64, 140.01, 132.10, 131.80, 128.55, 121.57, 121.37, 121.12, 119.71, 115.67, 104.28, 59.65, 57.38, 52.78, 49.00, 33.37, 25.61, 22.47, 14.15. LCMS: R_t_ = 3.27 min (method A). MS­(ESI+) [*m*/*z*]: calculated = 552.3 [M + H]^+^; found = 552.2 [M + H]^+^.

#### 2-((2-((4-(4-(5-(4-(4-((2-Aminoethyl)­amino)-2-(1-(4-chlorophenyl)­cyclohexyl)­quinazolin-7-yl)­piperazin-1-yl)-5-oxopentyl)­piperazin-1-yl)­phenyl)­amino)-5-chloropyrimidin-4-yl)-amino)­benzamide (D-2d)

A solution of ethyl 5-(4-(4-((4-((2-carbamoylphenyl)­amino)-5-chloropyrimidin-2-yl)­amino)­phenyl)­piperazin-1-yl)­pentanoate **(S-48)** (21 mg, 38 μmol, 1.0 equiv) and LiOH monohydrate (16 mg, 380 μmol, 10.0 equiv) in THF/MeOH/water (1/1/1, 6 mL) was stirred at 50 °C for 5.5 h. The mixture was acidified with HCl (1 M) and all volatiles were removed under reduced pressure. Tert-butyl (2-((2-(1-(4-chlorophenyl)­cyclohexyl)-7-(piperazin-1-yl)­quinazolin-4-yl)­amino)­ethyl)­carbamate **(S-16)** (24 mg, 42 μmol, 1.1 equiv) was added to a solution of the crude acid, DIPEA (15 μL, 114 μmol, 3.0 equiv) and PyBOP (24 mg, 46 μmol, 1.2 equiv) in DMF/THF (2 mL, 1/1). The reaction mixture was stirred overnight at 50 °C. All volatiles were removed under reduced pressure and the residue was stirred with DCM/TFA (1/1, 4 mL) for 2 h. All volatiles were removed under reduced pressure and the crude product was purified using preparative HPLC (ACN/H_2_O/TFA). TFA was removed by loading the sample on propylsulfonic acid-modified silica, purging with water, acetonitrile and methanol and mobilizing with ammonia in methanol. The title compound was isolated as a yellow solid (9 mg, 24%). ^1^H NMR (500 MHz, MeOD-*d*_4_): δ = 8.64 (d, *J* = 7.7 Hz, 1H), 8.11 (d, *J* = 9.4 Hz, 1H), 8.07 (s, 1H), 7.83 (dd, *J* = 7.9, 1.3 Hz, 1H), 7.49 (d, *J* = 8.8 Hz, 2H), 7.47–7.36 (m, 6H), 7.22 (t, *J* = 7.6 Hz, 1H), 7.09 (dd, *J* = 7.6, 5.7 Hz, 3H), 4.09 (t, *J* = 6.1 Hz, 2H), 3.86 (s, 2H), 3.82–3.67 (m, 6H), 3.66–3.61 (m, 2H), 3.61–3.54 (m, 2H), 3.34 (t, *J* = 6.1 Hz, 2H), 3.29–3.23 (m, 2H), 3.13 (s, 2H), 2.74 (dd, *J* = 12.6, 4.0 Hz, 2H), 2.58 (t, *J* = 6.9 Hz, 2H), 2.30 (t, *J* = 10.2 Hz, 2H), 1.92–1.83 (m, 2H), 1.79–1.70 (m, 4H), 1.68–1.48 (m, 4H). ^13^C NMR (126 MHz, MeOD-*d*_4_): δ = 173.5, 173.3, 168.3, 161.5, 158.0, 156.6, 155.9, 148.9, 146.6, 143.5, 142.6, 139.8, 134.4, 133.3, 132.1, 130.0, 129.7, 129.5, 126.4, 125.7, 125.0, 123.7, 122.6, 118.6, 117.9, 107.5, 103.7, 99.1, 57.6, 53.1, 50.7, 47.4, 47.1, 45.6, 42.1, 40.6, 39.7, 35.7, 32.9, 26.5, 24.6, 24.1, 22.8. LC: R_t_ = 11.69 min (method F); purity = > 98%. MS: MS­(ESI+) [*m*/*z*]: calculated = 970.45 [M + H]^+^; found = 970.58 [M + H]^+^. HRMS: (method 1): calculated = 970.4521 [M + H]^+^; found = 970.4519 [M + H]^+^.

#### 2-((2-((4-(4-(7-(4-(4-((2-Aminoethyl)­amino)-2-(1-(4-chlorophenyl)­cyclohexyl)­quinazolin-7-yl)­piperazin-1-yl)-7-oxoheptyl)­piperazin-1-yl)­phenyl)­amino)-5-chloropyrimidin-4-yl)-amino)­benzamide (D-2e)

The synthesis followed the general procedure of **D-2d** starting from ethyl 7-(4-(4-((4-((2-carbamoylphenyl)­amino)-5-chloropyrimidin-2-yl)­amino)­phenyl)­piperazin-1-yl)­heptanoate **(S49)** (18 mg, 31 μmol). The title compound was isolated as a yellow solid (10 mg, 32%). ^1^H NMR (500 MHz, MeOD*-d*_4_): δ = 8.66 (d, *J* = 7.9 Hz, 1H), 8.10 (d, *J* = 9.4 Hz, 1H), 8.05 (s, 1H), 7.82 (dd, *J* = 7.9, 1.1 Hz, 1H), 7.49 (d, *J* = 8.7 Hz, 2H), 7.47–7.36 (m, 6H), 7.20 (t, *J* = 7.6 Hz, 1H), 7.09 (d, *J* = 2.2 Hz, 1H), 7.07 (d, *J* = 8.9 Hz, 2H), 4.09 (t, *J* = 6.1 Hz, 2H), 3.84 (s, 2H), 3.80–3.74 (m, 4H), 3.70 (s, 2H), 3.64–3.59 (m, 2H), 3.58–3.53 (m, 2H), 3.37–3.33 (m, 2H), 3.26–3.20 (m, 2H), 3.12 (s, 2H), 2.80–2.70 (m, 2H), 2.49 (t, *J* = 7.4 Hz, 2H), 2.30 (t, *J* = 10.2 Hz, 2H), 1.88–1.79 (m, 2H), 1.79–1.45 (m, 12H). ^13^C NMR (126 MHz, MeOD*-d*_4_): δ = 174.2, 173.4, 168.3, 161.5, 157.8, 156.6, 156.4, 148.6, 147.8, 143.5, 142.6, 140.0, 134.4, 133.3, 132.6, 130.0, 129.7, 129.5, 126.4, 125.4, 124.7, 123.6, 122.4, 118.6, 117.9, 107.4, 103.7, 99.0, 57.9, 53.1, 50.7, 47.5, 47.2, 45.7, 42.1, 40.6, 39.7, 35.7, 33.7, 29.7, 27.3, 26.5, 25.9, 24.8, 24.1. LC: R_t_ = 11.25 min (method F); purity = > 97%. MS: MS­(ESI+) [*m*/*z*]: calculated = 998.49 [M + H]^+^; found = 998.58 [M + H]^+^. HRMS: (method 1): calculated = 998.4834 [M + H]^+^; found = 998.4830 [M + H]^+^.

#### Ethyl 10-(4-(4-((4-((2-carbamoylphenyl)­amino)-5-chloropyrimidin-2-yl)­amino)­phenyl)-piperazin-1-yl)­decanoate (S-50)

The synthesis followed the general procedure of **S-46** using ethyl 10-bromodecanoate (121 mg, 432 μmol). The title compound was isolated as a beige solid (71 mg, 40%). ^1^H NMR (500 MHz, DMSO*-d*_6_): δ = 11.84 (s, 1H), 9.19 (s, 1H), 8.80 (s, 1H), 8.28 (s, 1H), 8.15 (s, 1H), 7.81 (dd, ^3^ *J* = 7.9 Hz, ^4^ *J* = 1.4 Hz, 1H), 7.72 (s, 1H), 7.46 (t, ^3^ *J* = 6.3 Hz, 3H), 7.13–7.07 (m, 1H), 6.87 (d, ^3^ *J* = 9.1 Hz, 2H), 4.04 (q, ^3^ *J* = 7.1 Hz, 2H), 3.09–3.04 (m, 4H), 2.48 (d, ^3^ *J* = 5.1 Hz, 4H), 2.30 (t, ^3^ *J* = 7.3 Hz, 2H), 2.26 (t, ^3^ *J* = 7.4 Hz, 2H), 1.54–1.47 (m, 2H), 1.48–1.40 (m, 2H), 1.25 (s, 10H), 1.17 (t, ^3^ *J* = 7.1 Hz, 3H). ^13^C NMR (126 MHz, DMSO*-d*_6_): δ = 172.88, 171.02, 158.06, 154.94, 154.60, 146.64, 140.02, 132.09, 131.80, 128.55, 121.56, 121.36, 121.11, 119.70, 115.66, 104.29, 59.61, 57.93, 52.86, 49.00, 33.51, 28.90, 28.85, 28.63, 28.41, 26.95, 26.30, 24.45, 14.13. LCMS: R_t_ = 3.60 min (method A). MS­(ESI+) [*m*/*z*]: calculated = 622.3 [M + H]^+^; found = 622.4 [M + H]^+^.

#### 2-((2-((4-(4-(10-(4-(4-((2-Aminoethyl)­amino)-2-(1-(4-chlorophenyl)­cyclohexyl)­quinazolin-7-yl)­piperazin-1-yl)-10-oxodecyl)­piperazin-1-yl)­phenyl)­amino)-5-chloropyrimidin-4-yl)­amino)­benzamide (D-2f)

The synthesis followed the general procedure of **D-2d** starting from ethyl 10-(4-(4-((4-((2-carbamoylphenyl)­amino)-5-chloropyrimidin-2-yl)­amino)­phenyl)­piperazin-1-yl)­decanoate **(S-50)** (21 mg, 31 μmol). The title compound was isolated as a yellow solid (10 mg, 31%). ^1^H NMR (500 MHz, MeOD*-d*_4_): δ = 8.63 (d, *J* = 7.6 Hz, 1H), 8.09 (d, *J* = 9.4 Hz, 1H), 8.06 (s, 1H), 7.83 (dd, *J* = 7.9, 1.3 Hz, 1H), 7.53–7.47 (m, 2H), 7.47–7.40 (m, 3H), 7.40–7.34 (m, 3H), 7.22 (t, *J* = 7.6 Hz, 1H), 7.11–7.05 (m, 3H), 4.09 (t, *J* = 6.1 Hz, 2H), 3.86 (d, *J* = 7.7 Hz, 2H), 3.80–3.74 (m, 4H), 3.74–3.65 (m, 2H), 3.64–3.58 (m, 2H), 3.58–3.52 (m, 2H), 3.34 (t, *J* = 6.3 Hz, 2H), 3.29–3.19 (m, 4H), 3.17–3.08 (m, 2H), 2.80–2.69 (m, 2H), 2.46 (t, *J* = 7.6 Hz, 2H), 2.35–2.26 (m, 2H), 1.85–1.70 (m, 4H), 1.68–1.49 (m, 6H), 1.48–1.35 (m, 10H). ^13^C NMR (126 MHz, MeOD*-d*_4_): δ = 174.5, 173.3, 168.3, 161.5, 158.0, 156.6, 155.7, 149.0, 146.3, 143.4, 142.6, 139.7, 134.4, 133.3, 131.9, 130.0, 129.7, 129.5, 126.4, 125.8, 125.1, 123.7, 122.6, 118.5, 117.9, 107.5, 99.0, 58.0, 53.1, 50.7, 48.2, 47.6, 47.2, 45.8, 42.1, 40.6, 39.7, 35.7, 34.0, 30.4, 30.3, 30.1, 27.6, 26.5, 26.3, 25.0, 24.1. LC: R_t_ = 11.45 min (method F); purity = > 99%. MS: MS­(ESI+) [*m*/*z*]: calculated = 1041.53 [M+2H]^2+^; found = 1041.46 [M+2H]^2+^. HRMS: (method 1): calculated = 1040.5303 [M + H]^+^; found = 1040.5309 [M + H]^+^.

#### ***tert*** **-Butyl (2-((7-(4-(1-(4-(4-((4-((2-carbamoylphenyl)­amino)-5-chloropyrimidin-2-yl)-amino)­phenyl)­piperazin-1-yl)-3,6,9,12-tetraoxapentadecan-15-oyl)­piperazin-1-yl)-2-(1-(4-chlorophenyl)­cyclohexyl)­quinazolin-4-yl)­amino)­ethyl)­carbamate (D-2c** ^**n.c.**^).

A solution of the intermediate **(S-47)** (30 mg, 41 μmol, 1.0 equiv) in DCM/TFA (4.0 mL, 1/1) was stirred at r.t. for 3 h and all volatiles were removed under reduced pressure. The residue was dissolved in DCM and all volatiles were removed under reduced pressure (2x). PyBOP (26 mg, 49 μmol, 1.2 equiv) was added to a solution of the crude acid and DIPEA (22 μL, 0.12 mmol, 3.0 equiv) in DMF/THF (2.0 mL, 1/1) and the mixture was stirred at r.t. for 3 min. The DCAF1 ligand **(S-16)** (26 mg, 45 μmol, 1.1 equiv) was added and the reaction mixture was stirred at 50 °C overnight (15 h) and all volatiles were removed under reduced pressure. The crude material was purified using flash column chromatography (DCM/MeOH 10%) and preparative HPLC (H_2_O/MeCN/FA). The title compound was isolated as a beige solid (28 mg, 56%). ^1^H NMR (500 MHz, MeOD-*d*_4_): δ = 8.75 (d, ^3^ *J* = 7.5 Hz, 1H), 8.43 (s, 3H), 7.90 (d, ^3^ *J* = 8.8 Hz, 1H), 7.84 (s, 1H), 7.79 (d, ^3^ *J* = 8.1 Hz, 1H), 7.55–7.48 (m, ^3^ *J* = 13.5, 8.6 Hz, 4H), 7.45 (t, ^3^ *J* = 7.5 Hz, 1H), 7.37 (d, ^3^ *J* = 8.0 Hz, 2H), 7.15–7.09 (m, ^3^ *J* = 6.7 Hz, 2H), 7.01 (d, ^3^ *J* = 7.9 Hz, 2H), 6.88 (s, 1H), 3.89–3.83 (m, 4H), 3.75 (t, ^3^ *J* = 5.8 Hz, 2H), 3.73–3.69 (m, 2H), 3.69–3.62 (m, 12H), 3.58–3.54 (m, 2H), 3.45 (t, ^3^ *J* = 5.4 Hz, 2H), 3.41–3.29 (m, 10H), 3.25–3.21 (m, 2H), 2.74 (d, ^3^ *J* = 10.6 Hz, 2H), 2.60 (t, ^3^ *J* = 5.9 Hz, 2H), 2.26 (t, ^3^ *J* = 10.8 Hz, 2H), 1.76–1.68 (m, 2H), 1.68–1.60 (m, 3H), 1.55–1.48 (m, 1H), 1.44–1.25 (m, 11H). ^13^C NMR (126 MHz, MeOD-*d*_4_): δ = 173.7, 172.1, 168.4, 161.0, 159.5, 158.7, 156.8, 155.9, 155.1, 147.4, 144.2, 141.3, 134.9, 134.0, 133.2, 129.9, 129.6, 129.6, 125.8, 123.4, 123.1, 122.9, 121.3, 118.3, 117.1, 106.6, 104.1, 100.4, 80.3, 71.6, 71.6, 71.53, 71.51, 71.2, 68.2, 66.6, 57.5, 53.7, 50.6, 47.5, 47.2, 45.8, 43.6, 42.0, 40.6, 36.0, 34.3, 28.7, 26.6, 24.2. LCMS: R_t_ = 3.39 min (method A); purity = > 99%. MS­(ESI+) [*m*/*z*]: calculated = 609.8 [M/2+H]^+^; found = 610.6 [M/2+H]^+^. HRMS: (method 2): calculated = 1218.5781 [M + H]^+^; found = 1218.5824 [M + H]^+^.

#### 2-((5-Chloro-2-((4-(4-(3-(2-(2-((2-(2,6-dioxopiperidin-3-yl)-1,3-dioxoisoindolin-4-yl)­oxy)-ethoxy)­ethoxy)­propanoyl)­piperazin-1-yl)­phenyl)­amino)­pyrimidin-4-yl)­amino)­benzamide (C-2a)

A solution of *tert*-butyl 4-(4-((4-((2-carbamoylphenyl)­amino)-5-chloropyrimidin-2-yl)­amino)­phenyl)­piperazine-1-carboxylate **(S-12)** (22 mg, 43 μmol, 1.1 equiv) in DCM/TFA (4 mL, 1/1) was stirred for 4.5 h and evaporated under reduced pressure. The residue was suspended in DCM and evaporated. A solution of *tert*-butyl 3-(2-(2-((2-(2,6-dioxopiperidin-3-yl)-1,3-dioxoisoindolin-4-yl)­oxy)­ethoxy)­ethoxy)­propanoate **(S-38)** (19 mg, 39 μmol, 1.0 equiv) in DCM/TFA (4 mL, 1/1) was stirred for 4.5 h and evaporated under reduced pressure. The residue was suspended in DCM and evaporated. A solution of the amine, acid, HATU (18 mg, 46 μmol, 1.2 equiv) and DIPEA (20 μL, 116 μmol, 3.0 equiv) in DMF (2 mL) were stirred at r.t. overnight. DCM and a sat. aqueous solution of NH_4_Cl was added. The layers were separated and the aqueous layer was extracted with DCM (3x). The combined organic layers were dried with MgSO_4_ and the solvent was removed under reduced pressure. The crude material was purified using reversed phase flash column chromatography (H_2_O/ACN). The title compound was isolated as a beige solid (18 mg, 55%). ^1^H NMR (500 MHz, DCM-*d2*): δ = 11.23 (s, 1H), 8.94 (s, 1H), 8.71 (d, ^3^ *J* = 7.9 Hz, 1H), 8.06 (s, 1H), 7.67 (dd, ^3^ *J* = 8.4 Hz, ^3^ *J* = 7.3 Hz, 1H), 7.59 (dd, ^3^ *J* = 7.9 Hz, ^4^ *J* = 1.5 Hz, 1H), 7.47–7.40 (m, 4H), 7.25 (d, ^3^ *J* = 8.4 Hz, 1H), 7.22 (s, 1H), 7.11–7.06 (m, 1H), 6.90–6.86 (m, 2H), 6.30 (bs, 1H), 5.75 (bs, 1H), 4.93 (dd, ^3^ *J* = 12.2 Hz, ^3^ *J* = 5.5 Hz, 1H), 4.33–4.25 (m, 2H), 3.90–3.82 (m, 2H), 3.78–3.73 (m, 4H), 3.73–3.70 (m, 2H), 3.67–3.64 (m, 2H), 3.63–3.60 (m, 2H), 3.14–3.10 (m, 2H), 3.10–3.06 (m, 2H), 2.85–2.66 (m, 3H), 2.64 (td, ^3^ *J* = 6.5 Hz, ^4^ *J* = 2.4 Hz, 2H), 2.14–2.09 (m, 1H). ^13^C NMR (126 MHz, DCM-*d2*): δ = 171.8, 171.7, 170.0, 169.4, 167.5, 166.2, 158.7, 156.9, 156.3, 155.0, 147.8, 141.0, 137.1, 134.4, 133.2, 132.9, 128.2, 122.8, 122.5, 122.4, 120.2, 120.0, 117.8, 117.6, 116.5, 106.7, 71.6, 71.0, 69.8, 69.7, 68.1, 51.0, 50.52, 50.0, 46.3, 42.0, 34.1, 32.0, 23.2. LCMS: R_t_ = 3.77 min (method A); purity = 95%. MS­(ESI+) [*m*/*z*]: calculated = 840.3 [M + H]^+^; found = 840.3 [M + H]^+^. HRMS: (method 1): calculated = 840.2867 [M + H]^+^; found = 840.2863 [M + H]^+^.

#### ***tert*** **-Butyl 1-((2-(2,6-dioxopiperidin-3-yl)-1,3-dioxoisoindolin-4-yl)­oxy)-3,6,9,12-tetraoxapentadecan-15-oate (S-51)**

The synthesis followed the general procedure of **S-38** using *tert*-butyl 1-bromo-3,6,9,12-tetraoxapentadecan-15-oate (59 mg, 153 μmol). The title compound was isolated as a colorless oil (62 mg, 70%). ^1^H NMR (500 MHz, DCM*-d*_2_): δ = 8.47 (s, 1H), 7.73–7.66 (m, 1H), 7.44 (d, ^3^ *J* = 7.3 Hz, 1H), 7.30 (d, ^3^ *J* = 8.5 Hz, 1H), 4.95 (dd, ^3^ *J* = 12.0 Hz, ^4^ *J* = 5.5 Hz, 1H), 4.37–4.31 (m, 2H), 3.94–3.88 (m, 2H), 3.73 (dd, ^3^ *J* = 5.6 Hz, ^4^ *J* = 3.7 Hz, 2H), 3.68–3.64 (m, 2H), 3.63–3.55 (m, 10H), 2.87–2.69 (m, 3H), 2.45 (t, ^3^ *J* = 6.5 Hz, 2H), 2.16–2.10 (m, 1H), 1.42 (s, 9H). ^13^C NMR (126 MHz, DCM*-d*_2_): δ = 171.8, 171.3, 169.0, 167.5, 166.1, 157.0, 137.0, 134.4, 120.0, 117.8, 116.4, 80.8, 71.5, 71.1, 71.0, 70.92, 70.86, 69.86, 69.80, 67.4, 49.7, 36.8, 32.0, 28.4, 23.1. LCMS: R_t_ = 4.19 min (method A). MS­(ESI+) [*m*/*z*]: calculated = 601.2 [M + Na]^+^; found = 601.3 [M + Na]^+^.

#### 2-((5-Chloro-2-((4-(4-(1-((2-(2,6-dioxopiperidin-3-yl)-1,3-dioxoisoindolin-4-yl)­oxy)-3,6,9,12-tetraoxapentadecan-15-oyl)­piperazin-1-yl)­phenyl)­amino)­pyrimidin-4-yl)­amino)-benzamide (C-2b)

The synthesis followed the general procedure of **C-2a** starting from *tert*-butyl 1-((2-(2,6-dioxopiperidin-3-yl)-1,3-dioxoisoindolin-4-yl)­oxy)-3,6,9,12-tetraoxapentadecan-15-oate **(S-51)** (20 mg, 35 μmol). The title compound was isolated as a yellow oil (25 mg, 78%). ^1^H NMR (500 MHz, DCM-*d2*): δ = 11.25 (s, 1H), 9.31 (s, 1H), 8.70 (d, ^3^ *J* = 8.4 Hz, 1H), 8.06 (s, 1H), 7.67 (dd, ^3^ *J* = 8.4 Hz, ^3^ *J* = 7.4 Hz, 1H), 7.60 (dd, ^3^ *J* = 7.9 Hz, ^4^ *J* = 1.5 Hz, 1H), 7.45–7.40 (m, 4H), 7.35 (s, 1H), 7.26 (d, ^3^ *J* = 8.5 Hz, 1H), 7.09–7.06 (m, 1H), 6.90–6.86 (m, 2H), 6.40 (bs, 1H), 5.89 (bs, 1H), 4.94 (dd, ^3^ *J* = 12.3 Hz, ^3^ *J* = 5.4 Hz, 1H), 4.33–4.30 (m, 2H), 3.89–3.86 (m, 2H), 3.77–3.70 (m, 6H), 3.66–3.62 (m, 2H), 3.61–3.54 (m, 10H), 3.13–3.04 (m, 4H), 2.86–2.70 (m, 3H), 2.64 (t, ^3^ *J* = 6.5 Hz, 2H), 2.14–2.08 (m, 1H). ^13^C NMR (126 MHz, DCM-*d2*): δ = 172.0, 171.7, 170.0, 169.5, 167.6, 166.2, 158.7, 156.9, 156.3, 155.0, 147.8, 140.9, 137.0, 134.4, 133.2, 132.9, 128.3, 122.8, 122.5, 120.2, 120.0, 117.8, 117.6, 116.4, 106.6, 71.6, 71.1, 71.0, 70.99, 69.9, 69.8, 68.0, 50.96, 50.5, 49.7, 46.3, 42.0, 34.1, 32.0, 23.2. LCMS: R_t_ = 3.72 min (method A); purity = 98%. MS­(ESI+) [*m*/*z*]: calculated = 928.3 [M + H]^+^; found = 928.3 [M + H]^+^. HRMS: (method 2): calculated = 928.3391 [M + H]^+^; found = 928.3420 [M + H]^+^.

#### ***tert*** **-Butyl (2-(2-(2-(2-(4-(4-((4-((2-carbamoylphenyl)­amino)-5-chloropyrimidin-2-yl)­amino)­phenyl)­piperazin-1-yl)­ethoxy)­ethoxy)­ethoxy)­ethyl)­carbamate (S-52)**

A solution of *tert*-butyl 4-(4-((4-((2-carbamoylphenyl)­amino)-5-chloropyrimidin-2-yl)­amino)­phenyl)­piperazine-1-carboxylate **(S-12)** (180 mg, 344 μmol, 1.0 equiv) in HCl/1,4-dioxane (4 mL, 2M) was stirred for 2 h and evaporated under reduced pressure. The residue was suspended in DCM and evaporated 2 times. A mixture of the amine, *tert*-butyl (2-(2-(2-(2-bromoethoxy)­ethoxy)­ethoxy)­ethyl)­carbamate (128 mg, 361 μmol, 1.1 equiv) and K_2_CO_3_ (71 mg, 515 μmol, 1.5 equiv) in DMF (4 mL) was stirred at room temperature for 5 h, then at 40 °C for 18 h. All volatiles were removed under reduced pressure. The crude material was purified by flash column chromatography (DCM/MeOH 1%–5%). The title crude title compound was used without further purification (yellow wax, 152 mg, 63%). LCMS: R_t_ = 4.81 min (method A). MS­(ESI+) [*m*/*z*]: calculated = 699.3 [M + H]^+^; found = 699.3 [M + H]^+^.

#### 2-((5-Chloro-2-((4-(4-(1-((2-(2,6-dioxopiperidin-3-yl)-1,3-dioxoisoindolin-4-yl)­oxy)-2-oxo-6,9,12-trioxa-3-azatetradecan-14-yl)­piperazin-1-yl)­phenyl)­amino)­pyrimidin-4-yl)­amino)­benzamide (C-2c)

A solution of HCl in 1,4-dioxane (1.0 mL, 4M) was added to a solution of *tert*-butyl (2-(2-(2-(2-(4-(4-((4-((2-carbamoylphenyl)­amino)-5-chloropyrimidin-2-yl)­amino)­phenyl)­piperazin-1-yl)­ethoxy)­ethoxy)­ethoxy)­ethyl)­carbamate **(S-52)** (28 mg, 40 μmol, 1.0 equiv) in 1,4-dioxane/water (4.0 mL/0.4 mL) and was stirred at r.t. for 3.5 h. All volatiles were removed under reduced pressure and the residue was dissolved in DMF (6.0 mL) and TEA (0.6 mL). All volatiles were removed under reduced pressure. A solution of *tert*-butyl 2-((2-(2,6-dioxopiperidin-3-yl)-1,3-dioxoisoindolin-4-yl)­oxy)­acetate **(S-23)** (16 mg, 40 μmol, 1.0 equiv) in DCM/TFA (1 mL, 3/2) was stirred at r.t. for 2 h. All volatiles were removed under reduced pressure and the residue was dissolved in DCM/MeOH (3.0 mL, 2/1) and TEA (150 μL). All volatiles were removed under reduced pressure. To a solution of the crude amine DMF (2.0 mL) were added HOBt (0.5 mg, 4 μmol, 0.1 equiv), the crude acid, 4-methylmorpholine (22 μL, 22 μmol, 5.0 equiv) and EDC hydrochloride (9 mg, 48 μmol, 1.2 equiv). The reaction mixture was stirred at r.t. for 12 h and all volatiles were removed under reduced pressure. The crude material was purified using reversed phase flash column chromatography (H_2_O/ACN). The title compound was isolated as yellow wax (15 mg, 41%). ^1^H NMR (500 MHz, MeOD): δ = 8.65 (d, ^3^ *J* = 8.3 Hz, 1H), 8.02 (s, 1H), 7.78 (dd, ^3^ *J* = 7.9 Hz, ^4^ *J* = 1.4 Hz, 1H), 7.70 (dd, ^3^ *J* = 8.3 Hz, ^3^ *J* = 7.4 Hz, 1H), 7.45 (d, ^3^ *J* = 7.3 Hz, 1H), 7.42–7.38 (m, 3H), 7.28 (d, ^3^ *J* = 8.4 Hz, 1H), 7.16–7.10 (m, 1H), 6.96 (d, ^3^ *J* = 8.9 Hz, 2H), 5.09 (dd, ^3^ *J* = 12.7, ^3^ *J* = 5.5 Hz, 1H), 4.61 (s, 2H), 3.91–3.87 (m, 2H), 3.73–3.58 (m, 16H), 3.51–3.42 (m, 6H), 2.90–2.81 (m, 1H), 2.78–2.62 (m, 2H), 2.15–2.07 (m, 1H). ^13^C NMR (126 MHz, MeOD): δ = 173.1, 172.1, 170.1, 168.4, 166.8, 166.3, 156.4, 155.9, 154.5, 150.1, 146.2, 139.2, 136.8, 133.4, 132.6, 131.8, 128.2, 122.7, 122.6, 121.9, 120.5, 119.9, 117.6, 117.1, 116.51, 105.6, 70.1, 69.96, 69.9, 68.9, 67.5, 63.8, 55.8, 52.0, 49.1, 46.9, 38.7, 30.8, 22.2. LCMS: R_t_ = 6.87 min (method D); purity = 97%. MS­(ESI+) [*m*/*z*]: calculated = 913.3 [M + H]^+^; found = 913.3 [M + H]^+^. HRMS: (method 1): calculated = 913.3395 [M + H]^+^; found = 913,3387 [M + H]^+^.

#### ***tert*** **-Butyl (7-(4-(4-((4-((2-carbamoylphenyl)­amino)-5-chloropyrimidin-2-yl)­amino)-phenyl)­piperazin-1-yl)­heptyl)­carbamate (S-53)**

The synthesis followed the general procedure of **D-2d** using *tert*-butyl 7-bromoheptyl)­carbamate (73 mg, 249 μmol). The crude off-white title compound was used without further purification (75 mg, 71%). LCMS: R_t_ = 4.81 min (method A). MS­(ESI+) [*m*/*z*]: calculated = 637.3 [M + H]^+^; found = 637.3 [M+2Na–H]^+^.

#### 2-((5-Chloro-2-((4-(4-(7-(2-((2-(2,6-dioxopiperidin-3-yl)-1,3-dioxoisoindolin-4-yl)­oxy)-acetamido)­heptyl)­piperazin-1-yl)­phenyl)­amino)­pyrimidin-4-yl)­amino)­benzamide (C-2d)

A solution of *tert*-butyl *tert*-butyl (7-(4-(4-((4-((2-carbamoylphenyl)­amino)-5-chloropyrimidin-2-yl)­amino)­phenyl)­piperazin-1-yl)­heptyl)­carbamate **(S-53)** (20 mg, 31 μmol, 1.1 equiv) in DCM/TFA (4 mL, 1/1) was stirred for 3.5 h and evaporated under reduced pressure. The residue was suspended in DCM and evaporated two times. A solution of *tert*-butyl 2-((2-(2,6-dioxopiperidin-3-yl)-1,3-dioxoisoindolin-4-yl)­oxy)­acetate **(S-23)** (11 mg, 29 μmol, 1.0 equiv) in DCM/TFA (4 mL, 1/1) was stirred for 3.5 h and evaporated under reduced pressure. The residue was suspended in DCM and evaporated two times.

A solution of the amine, acid, HATU (13 mg, 34 μmol, 1.2 equiv) and DIPEA (20 μL, 114 μmol, 4.0 equiv) in DMF (2 mL) were stirred at r.t. overnight. DCM and a sat. aqueous solution of NH_4_Cl was added. The layers were separated and the aqueous layer was extracted with DCM (3x). The combined organic layers were dried with MgSO_4_ and the solvent was removed under reduced pressure. The crude material was purified using reversed phase flash column chromatography (H_2_O/ACN). Preparative HPLC yielded the title compound as a beige solid (8 mg, 33%). ^1^H NMR (500 MHz, MeOD*-d4*): δ = 8.72 (d, ^3^ *J* = 8.3 Hz, 1H), 8.09 (s, 1H), 7.86–7.82 (m, 2H), 7.57 (d, ^3^ *J* = 7.3 Hz, 1H), 7.50–7.44 (m, 4H), 7.20 (t, ^3^ *J* = 7.3 Hz, 1H), 7.07 (d, ^3^ *J* = 9.0 Hz, 2H), 5.17 (dd, ^3^ *J* = 12.7 Hz, ^3^ *J* = 5.5 Hz, 1H), 4.79 (s, 2H), 3.84 (s, 2H), 3.70 (s, 2H), 3.39–3.35 (m, 2H), 3.29 (s, 2H), 3.25–3.20 (m, 2H), 3.10 (s, 2H), 2.91 (ddd, ^3^ *J* = 17.6 Hz, ^4^ *J* = 14.0, ^3^ *J* = 5.3 Hz, 1H), 2.84–2.67 (m, 2H), 2.22–2.14 (m, 1H), 1.85–1.77 (m, 2H), 1.68–1.60 (m, 2H), 1.50–1.42 (m, 6H). ^13^C NMR (126 MHz, MeOD*-d4*): δ = 174.6, 173.5, 171.4, 169.9, 168.3, 167.9, 157.6, 156.3, 150.1, 148.1, 140.4, 138.3, 134.9, 133.4, 133.2, 129.6, 124.9, 124.3, 123.4, 122.2, 121.8, 119.3, 118.6, 118.0, 69.4, 58.0, 53.1, 50.6, 49.0, 40.0, 32.2, 30.0, 29.6, 27.4, 27.4, 24.9, 23.7. LCMS: R_t_ = 5.34 min (method B); purity = > 99%. MS­(ESI+) [*m*/*z*]: calculated = 851.3 [M + H]^+^; found = 851.3 [M + H]^+^. HRMS: (method 1): calculated = 851.3390 [M + H]^+^; found = 851.3382 [M + H]^+^.

## Supplementary Material







## Notes

Processed proteomics and ubiquitinomics data (XLSX)
Molecular formula strings and associated biochemical data (CSV)
Supplementary elements Figure S1–S180 and Tables S1–S7, including additional biochemical and biological assay data as well as small molecule synthesis schemes and characterization data (PDF)

## Abbreviations

cmpd: compound
conc.: concentration
CRBN: cereblon
DCAF1: DDB1 and CUL4 Associated Factor 1
DIPEA: Diisopropylethylamine
EtOAc: ethyl acetate
EtOH: ethanol
FA: formic acid
HATU: O-(7-azabenzotriazol-1-yl)-N,N,N’,N’-tetramethyluronium hexafluorophosphate
HBA: H-bond acceptor
HBD: H-bond donor
MeOH: methanol
MW: molecular weight
n.c.: negative control
nLuc: nano Luciferase
POI: protein of interest
PROTAC: proteolysis targeting chimera
PyAOP: (7-azabenzotriazol-1-yloxy)­tripyrrolidinophosphonium hexafluorophosphate
PyBOP: (benzotriazol-1-yloxy)­tripyrrolidinophosphonium hexafluorophosphate
RB: rotatable bonds
TEA: triethylamine
TPSA: total polar surface area
UPS: ubiquitin proteasome system
VHL: von Hippel-Lindau.
