# Supplementary material for: On the Scope of DCAF1-Recruiting PROTACs Degrading Protein Kinases
Source: J Med Chem. 2026 Jun 17;69(13):15531–57. doi: 10.1021/acs.jmedchem.6c00383 (PMC13370871; doi:10.1021/acs.jmedchem.6c00383)
Supplement: Supplementary file 3 [file jm6c00383_si_003.pdf]

Supporting Information

Supplementary file 1

On the Scope of DCAF1-Recruiting PROTACs Degrading Protein Kinases

Janik Weckesser[1,2]†, Nebojša Miletić[1,2]†, Saran Aswathaman Sivashanmugam[1,2]†, Uli Ohmayer[5], Martin Steger[5], Bachuki Shashikadze[5], Paul Gehrtz[6], Andrea Unzue Lopez[6], Ansgar Wegener[6], Timo Yoshua Dietz[6], Tobias Hammann[6], Ingo V. Hartung[6], Lewis Elson[1,2], Václav Němec[1,2], Martin Peter Schwalm[1,2,3], Bikash Adhikari[7], Elmar Wolf[7], Andreas Krämer[1,2], Susanne Müller[1,2,4], Henrik Daub[5], Stefan Knapp[1,2,3,4]*

[1]Institute for Pharmaceutical Chemistry, Department of Biochemistry, Chemistry and Pharmacy, Goethe University Frankfurt, Max-von-Laue-Straße 9, 60438 Frankfurt, Germany

[2]Structural Genomics Consortium, Buchmann Institute for Molecular Life Sciences, Goethe University Frankfurt, Max-von-Laue-Straße 15, 60438 Frankfurt, Germany

[3]German Cancer Consortium (DKTK), partner site 60438 Frankfurt/Mainz, Germany

[4]FCI Frankfurt Cancer Center (FCI), Georg-Speyerhaus, 60438 Frankfurt am Main, Germany

[5]NEOsphere Biotechnologies GmbH, Fraunhofer Str. 1, 82152 Martinsried, Germany

[6]Merck Healthcare KGaA, 64293 Darmstadt, Germany

[7]Institute of Biochemistry, University of Kiel, 24118 Kiel, Germany

† these authors contributed equally

* correspondence: knapp@pharmchem.uni-frankfurt.de

Table of contents

1. Target Engagement Data – NanoBRET Assay 4
   1.1. Promiscuous Kinase PROTACs 4
   1.2. Positive Controls JB300 and BI-0319 6
   1.3. Negative Controls D-1a$^{n.c.}$ and D-2c$^{n.c.}$ 9
2. Cell Viability Data – CellTiterGLO Assay 12
   2.1. Promiscuous Kinase PROTACs 12
   2.2. Negative Controls D-1a$^{n.c.}$ and D-2c$^{n.c.}$ 14
3. MS-Proteomic Data 16
   3.1. DCAF1-recruiting Promiscuous Kinase PROTACs 16
   3.2. Negative Controls 24
   3.3. CRBN-recruiting Promiscuous Kinase PROTACs 30
4. Hit Validation 39
   4.1. Luciferase Assay 39
   4.2. Western Blotting 41
   4.3. HiBiT Split Luciferase Assay 42
5. Physicochemical Properties of Utilized E3 Ligase Ligands 45
6. Chemical Synthesis Schemes 46
   6.1. DCAF1 Parent Ligand and tracer 46
   6.2. Promiscuous Kinase PROTACs 47
      6.2.1. DCAF1-recruiting Promiscuous Kinase PROTACs based on Kinase Parent Inhibitor 1inh 47
      6.2.2. CRBN-recruiting Promiscuous Kinase PROTACs based on Kinase Parent Inhibitor 1inh 48
      6.2.3. DCAF1-recruiting Promiscuous Kinase PROTACs based on Kinase Parent Inhibitor 2inh 49
      6.2.4. CRBN-recruiting Promiscuous Kinase PROTACs based on Kinase Parent Inhibitor 2inh 50
7. Compound Characterization Data 51
   7.1. DCAF1 Parent Ligand and Corresponding Negative Controls 51
   7.2. Promiscuous Kinase PROTACs 66
      7.2.1. DCAF1-recruiting Promiscuous Kinase PROTACs based on Kinase Parent Inhibitor 1inh 66
      7.2.2. CRBN-recruiting Promiscuous Kinase PROTACs based on Kinase Parent Inhibitor 1inh 97
      7.2.3. DCAF1-recruiting Promiscuous Kinase PROTACs based on Kinase Parent Inhibitor 2inh 121
      7.2.4. CRBN-recruiting Promiscuous Kinase PROTACs based on Kinase Parent Inhibitor 2inh 150

1. Target Engagement Data – NanoBRET Assay

1.1. Promiscuous Kinase PROTACs

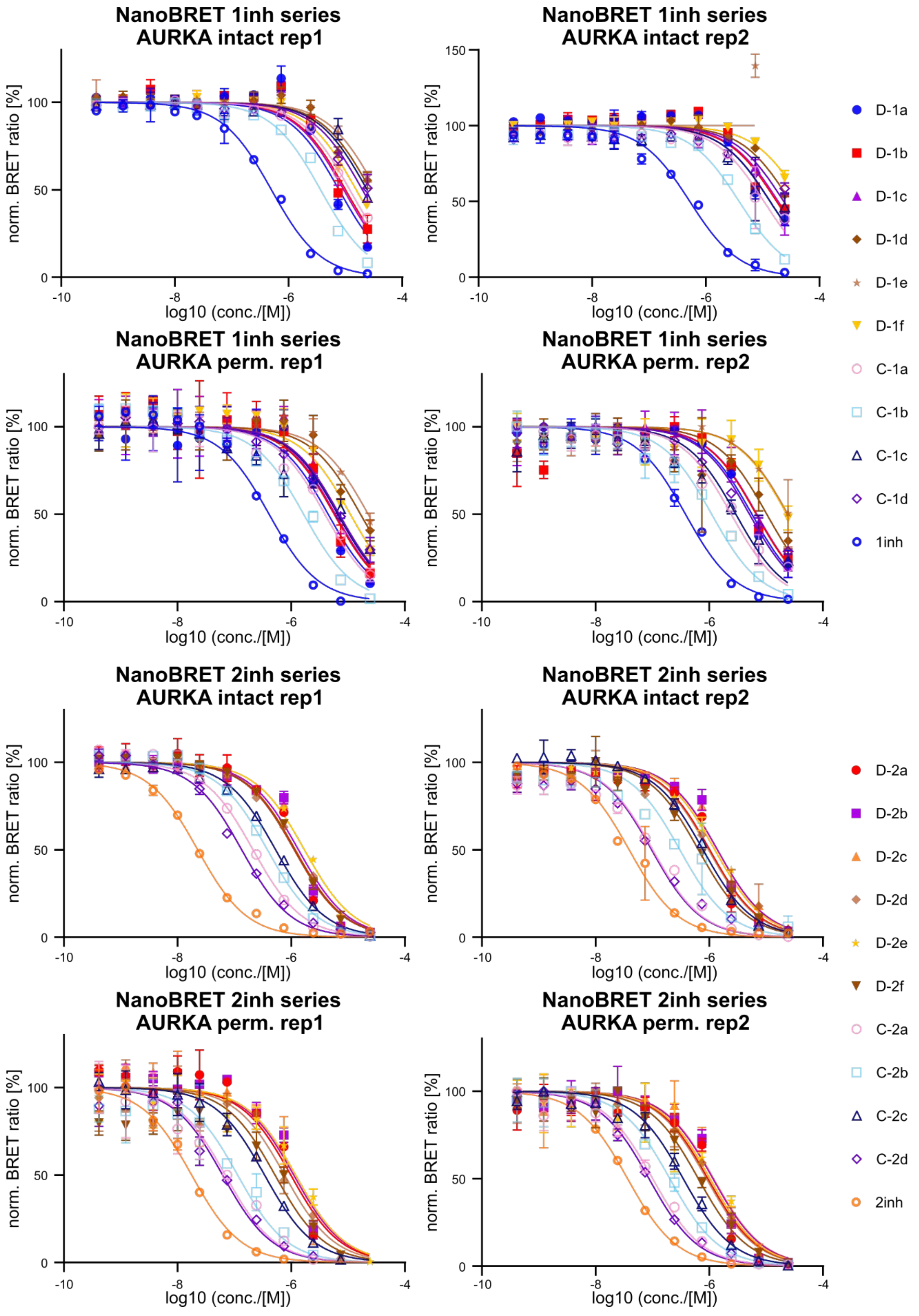

Figure S1: *Cellular target engagement data (NanoBRET) of all promiscuous kinase PROTACs and respective kinase parent ligands tested against AURKA in intact and digitonin-treated (permeabilized, perm.) cells. Each curve represents the mean of technical replicates (n=2). Error bars indicate the standard deviation.*

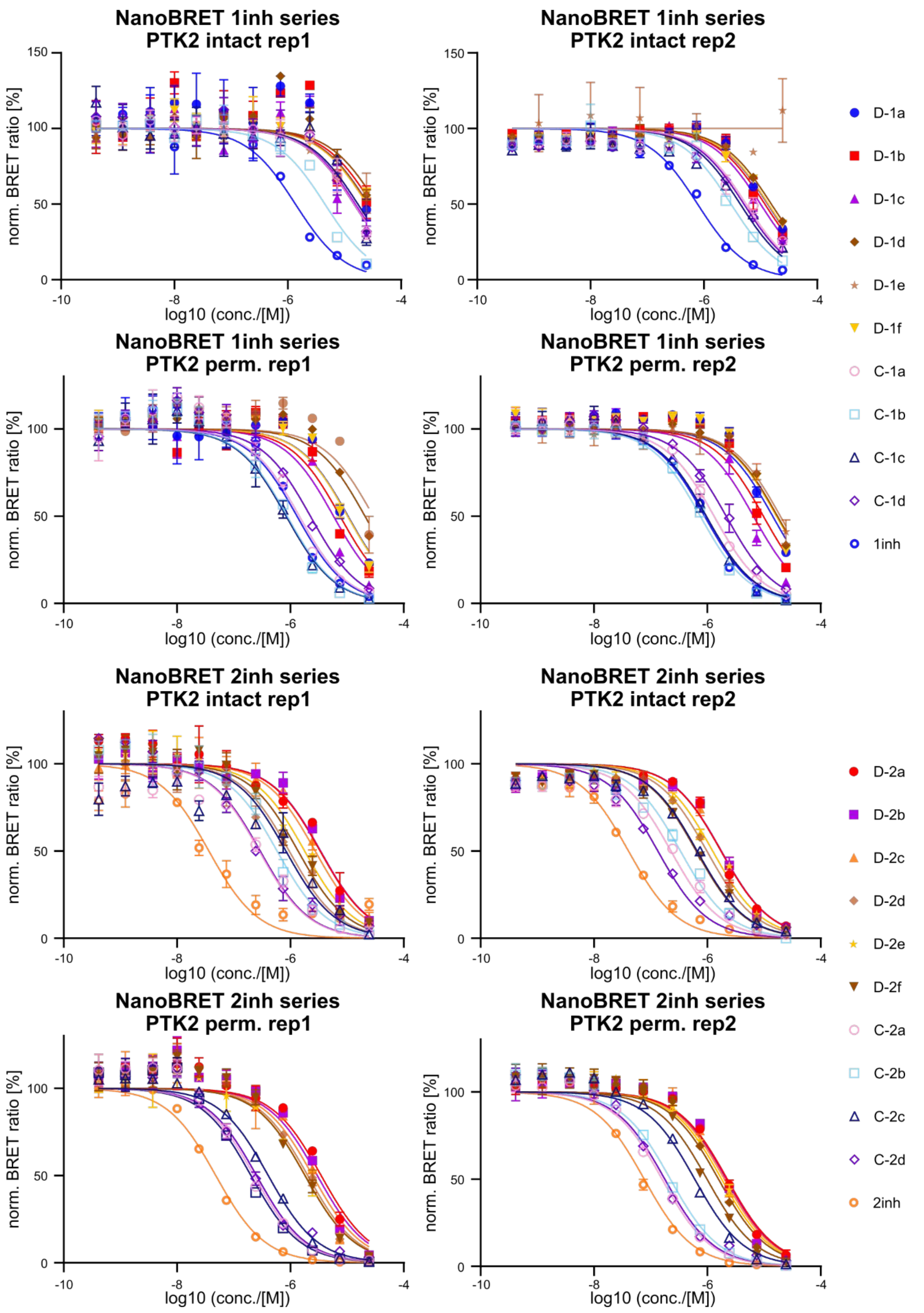

Figure S2: *Cellular target engagement data (NanoBRET) of all promiscuous kinase PROTACs and respective kinase parent ligands tested against PTK2 in intact and digitonin-treated (permeabilized, perm.) cells. Each curve represents the mean of technical replicates (n=2). Error bars indicate the standard deviation.*

1.2. Positive Controls JB300 and BI-0319

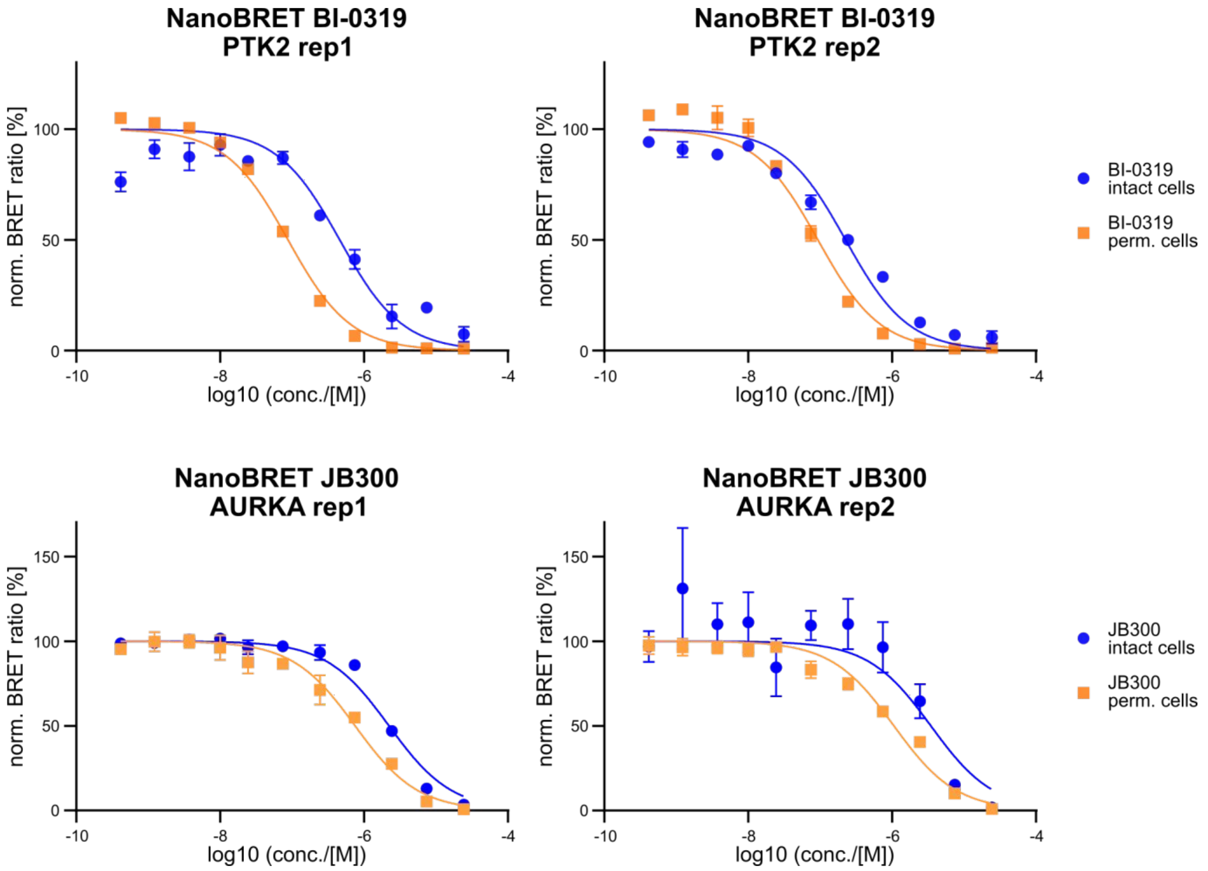

Figure S3: *Cellular target engagement data (NanoBRET) of the positive controls **JB300** and **BI-0319** tested against AURKA and PTK2 in intact and digitonin-treated (permeabilized, perm.) cells. Each curve represents the mean of technical replicates (n=2). Error bars indicate the standard deviation.*

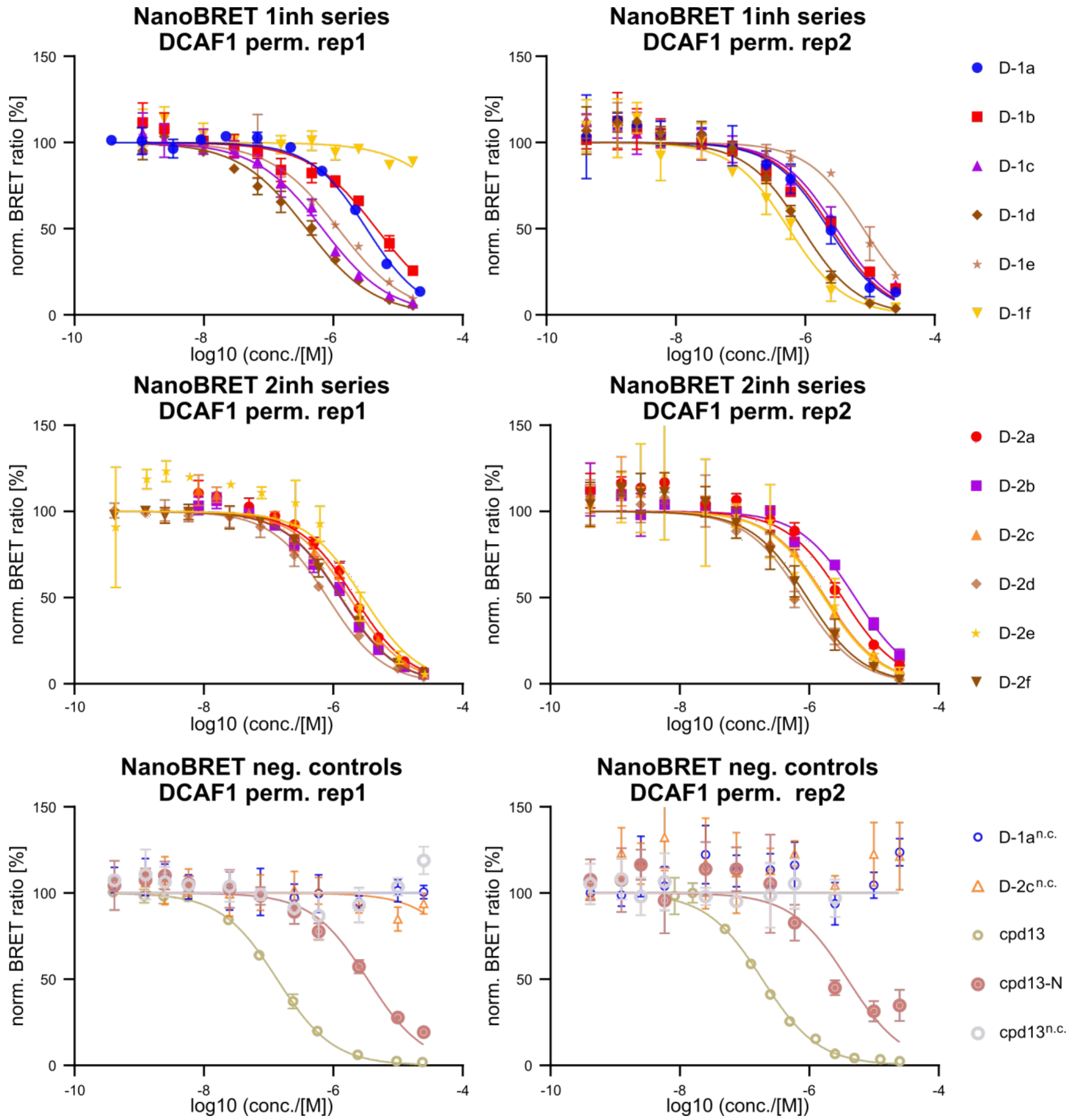

Figure S4: *Target engagement data (NanoBRET) of all DCAF1-recruiting promiscuous kinase PROTACs, the parent ligand* ***cpd13*** *and negative controls tested against DCAF1 in digitonin-treated (permeabilized, perm.) cells. Each curve represents the mean of technical replicates (n=2). Error bars indicate the standard deviation.*

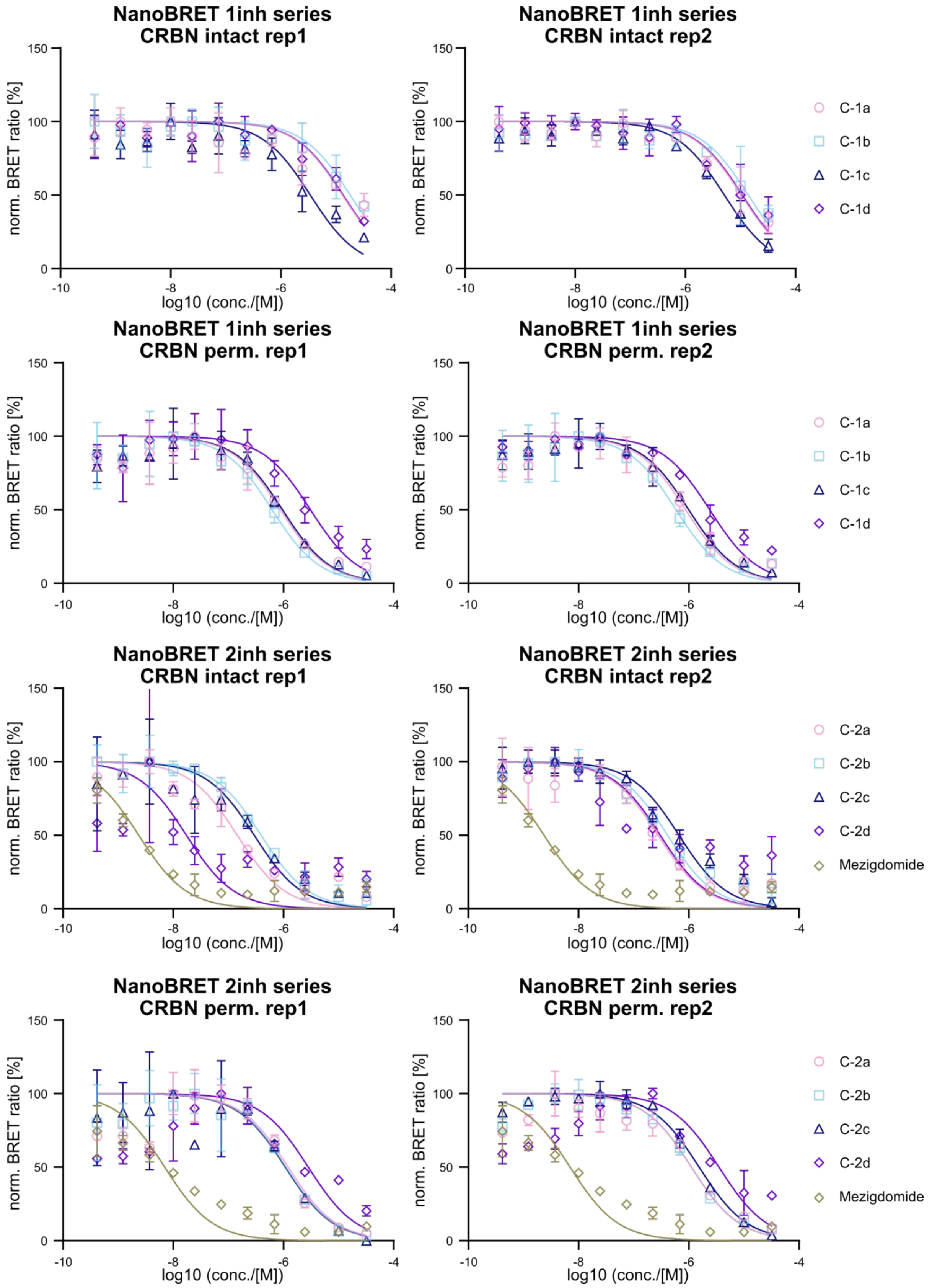

Figure S5: *Target engagement data (NanoBRET) of all CRBN-recruiting promiscuous kinase PROTACs and positive control* ***Mezigdomide*** *tested against CRBN in intact and digitonin-treated (permeabilized, perm.) cells. Each curve represents the mean of technical replicates (n=2). Error bars indicate the standard deviation.*

1.3. Negative Controls D-1a$^{n.c.}$ and D-2c$^{n.c.}$

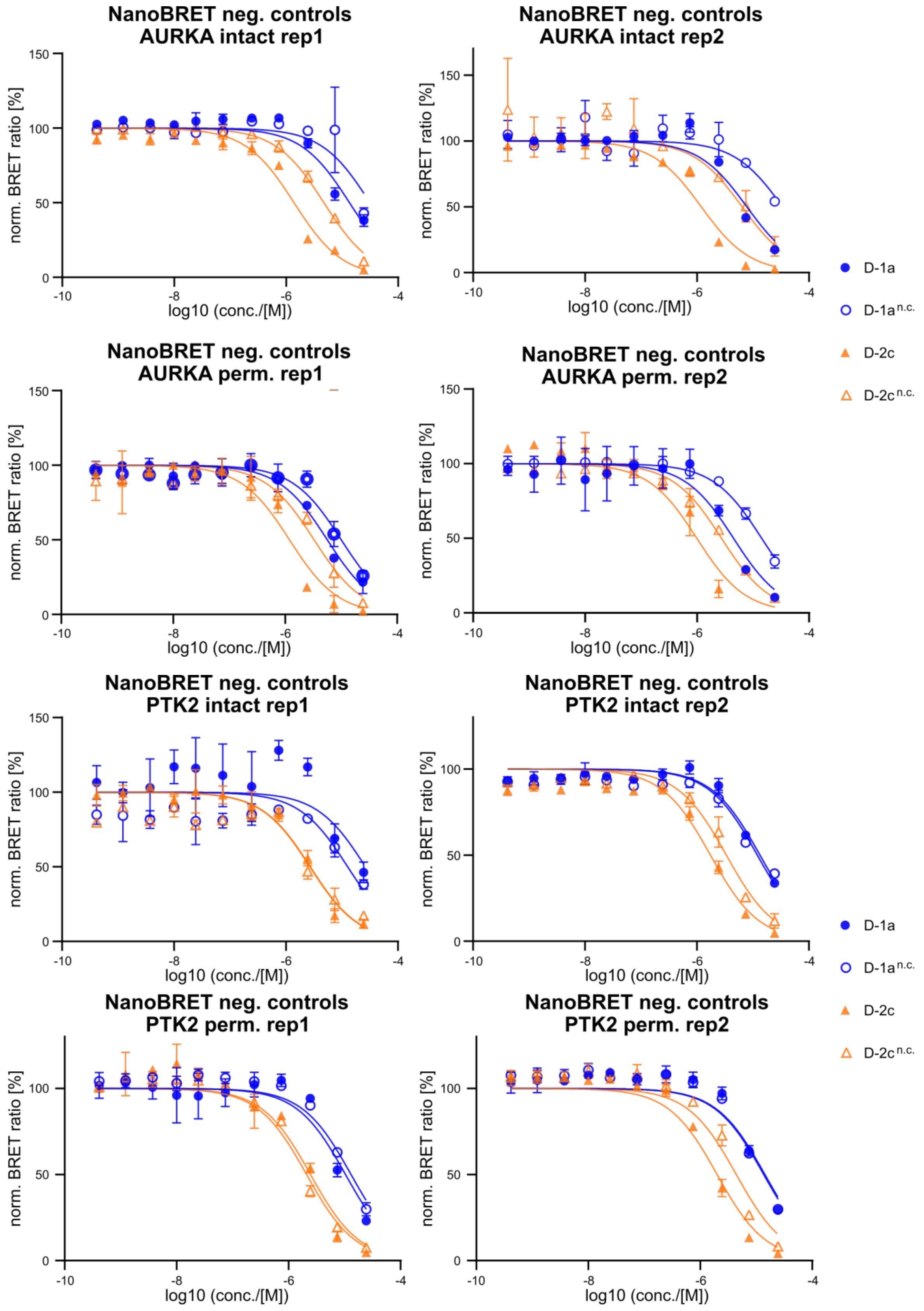

Figure S6: *Cellular target engagement data (NanoBRET) of the negative controls **D-1a$^{n.c.}$** and **D-2c$^{n.c.}$** tested against AURKA and PTK2 in intact and digitonin-treated (permeabilized, perm.) cells. Results for the active degraders **D-1a** and **D-2a** are included for a direct comparison. Each curve represents the mean of technical replicates (n=2). Error bars indicate the standard deviation.*

Table S1: *Target engagement data (NanoBRET) of all promiscuous kinase PROTACs, respective kinase parent ligands, the positive controls* ***JB300*** *and* ***BI-0319*** *as well as negative controls* ***D-1a***[n.c.] *and* ***D-2c***[n.c.] *tested against AURKA and PTK2 in intact and digitonin-treated (permeabilized, perm.) cells.* EC_{50} *values were calculated from biological replicates (n=4). Errors were calculated using the standard deviation.* [a]*Estimated* EC_{50} *values based on extrapolation.* [b]***D-1f*** *precipitated during the assay.*

cmpd ID	EC_{50}(AURKA) [µM] intact	EC_{50}(AURKA) [µM] perm.	EC_{50}(PTK2) [µM] intact	EC_{50}(PTK2) [µM] perm.
1inh	0.51±0.02	0.40±0.02	1.07±0.42	1.10±0.29
D-1a	10.64±4.12[a]	5.07±0.84	19.90±9.17[a]	12.34±2.72
D-1a[n.c.]	>20	12.16±3.00	12.36±0.47	13.34±0.36
D-1b	13.87±5.35[a]	6.21±0.73	>20	8.66±1.58
D-1c	14.11±6.71[a]	6.44±0.65	12.79±3.93[a]	6.16±0.53
D-1d	>20	13.46±4.42	>20	19.03±2.42
D-1e	>20	>20	>20	>20
D-1f	-[b]	-[b]	-[b]	-[b]
C-1a	11.27±0.95	3.18±1.04	10.17±6.50	1.38±0.09
C-1b	3.77±0.15	1.38±0.39	3.77±1.07	0.82±0.09
C-1c	18.86±7.46[a]	4.77±2.57	10.84±9.11	0.90±0.08
C-1d	>20	6.07±1.35	9.91±6.80	2.21±0.07
2inh	0.03±0.01	0.03±0.01	0.04±0.01	0.06±0.02
D-2a	1.08±0.08	0.97±0.02	2.54±1.13	2.87±0.87
D-2b	1.37±0.04	1.14±0.04	2.59±1.16	2.57±0.59
D-2c	1.29±0.14	1.09±0.15	2.24±0.70	2.24±0.33
D-2c[n.c.]	5.56±1.17	3.01±0.40	3.02±0.48	3.25±1.63
D-2d	1.05±0.11	0.83±0.09	0.97±0.02	1.80±0.32
D-2e	1.48±0.32	1.12±0.11	1.51±0.38	1.81±0.02
D-2f	0.91±0.37	0.58±0.09	1.00±0.52	1.40±0.58
C-2a	0.16±0.08	0.09±0.02	0.26±0.04	0.19±0.05
C-2b	0.34±0.05	0.16±0.06	0.46±0.17	0.21±0.02
C-2c	0.66±0.17	0.34±0.01	0.75±0.12	0.50±0.14
C-2d	0.12±0.03	0.07±0.02	0.21±0.11	0.22±0.05
JB300	2.84±0.88	0.91±0.22	n.d.	n.d.
BI-0349	n.d.	n.d.	0.34±0.17	0.09±0.01

Table S2: *Target engagement data (NanoBRET) of all promiscuous kinase PROTACs, respective kinase parent ligands, positive controls **cpd13** and **Mezigdomide** as well as negative controls **D-1a**$^{n.c.}$ and **D-2c**$^{n.c.}$ tested against DCAF1 and CRBN in intact (only CRBN) and digitonin-treated (permeabilized, perm.) cells. EC_{50} values were calculated from biological replicates (n=4). Errors were calculated using the standard deviation.*

cmpd ID	EC_{50}(DCAF1) [μM]	EC_{50}(CRBN) [μM]	
	perm.	intact	perm.
D-1a	2.97±0.95	n.d.	n.d.
D-1a$^{n.c.}$	>20	n.d.	n.d.
D-1b	5.51±4.20	n.d.	n.d.
D-1c	2.37±0.93	n.d.	n.d.
D-1d	0.98±0.12	n.d.	n.d.
D-1e	5.32±3.66	n.d.	n.d.
C-1a	n.d.	11.76±1.90	0.88±0.01
C-1b	n.d.	15.97±2.94	0.61±0.03
C-1c	n.d.	4.27±1.20	0.98±0.03
C-1d	n.d.	12.14±1.90	2.75±0.49
D-2a	2.70±0.80	n.d.	n.d.
D-2b	3.23±2.73	n.d.	n.d.
D-2c	1.80±0.01	n.d.	n.d.
D-2c$^{n.c.}$	>20	n.d.	n.d.
D-2d	0.80±0.03	n.d.	n.d.
D-2e	2.20±0.72	n.d.	n.d.
D-2f	1.13±0.27	n.d.	n.d.
C-2a	n.d.	0.21±0.09	1.17±0.07
C-2b	n.d.	0.40±0.01	1.11±0.01
C-2c	n.d.	0.48±0.26	1.31±0.33
C-2d	n.d.	0.16±0.20	3.18±0.50
cpd13	0.17±0.03	n.d.	n.d.
cpd13-N	3.65±0.35	n.d.	n.d.
cpd13$^{n.c.}$	-	n.d.	n.d.
Mezigd.	n.d.	0.002±0.01	0.007±0.01

2. Cell Viability Data – CellTiterGLO Assay

2.1. Promiscuous Kinase PROTACs

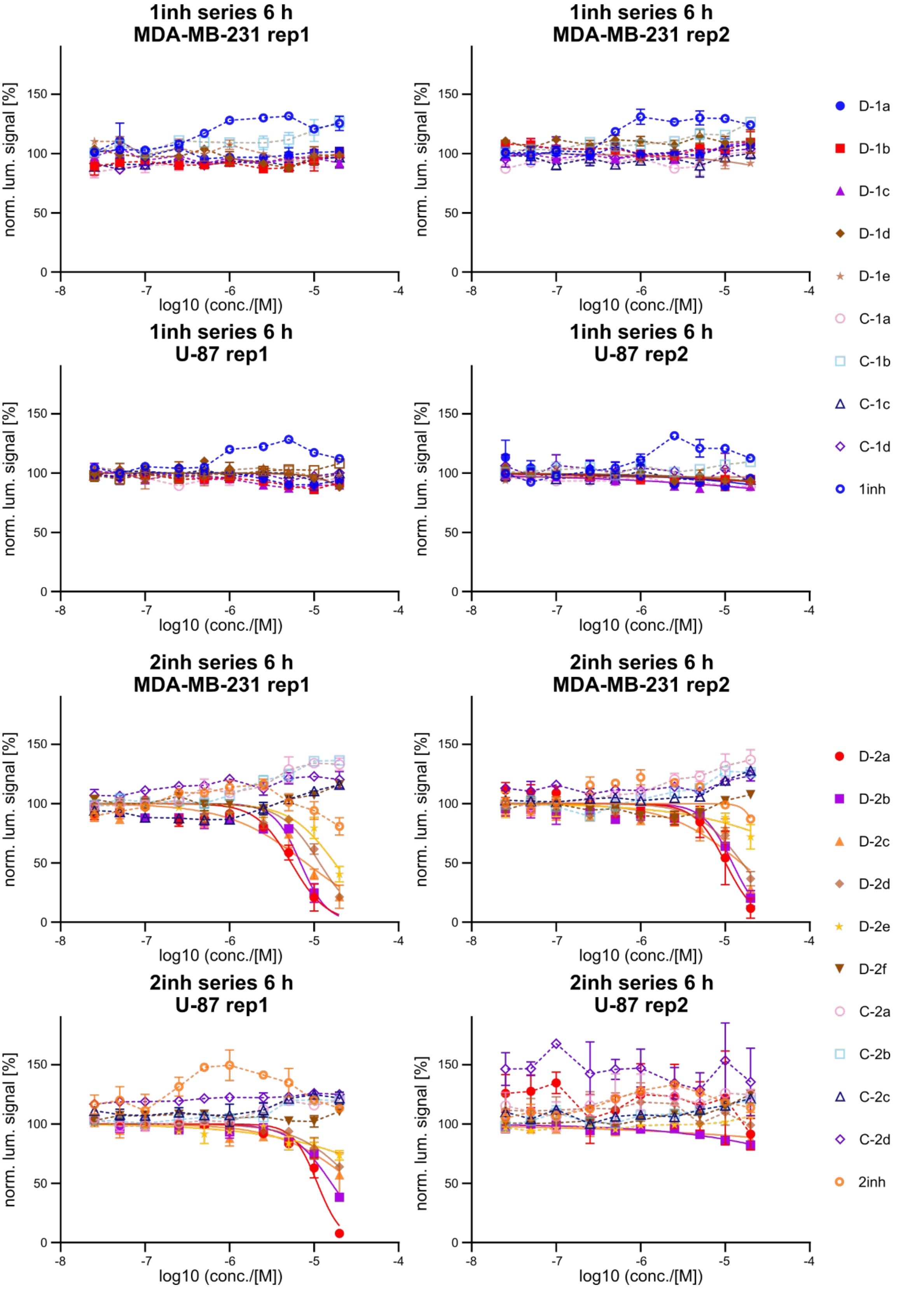

Figure S7: *Cell viability data (CellTiter Glo) of all promiscuous kinase PROTACs and respective kinase parent ligands. MDA-MB-231 and U-87 cells were treated in a 10-point dose series of the indicated compounds for 6 h.*

Each curve represents the mean of technical replicates (n=2). Error bars indicate the standard deviation. Dotted lines connect data points for which no sigmoidal curve could be fitted.

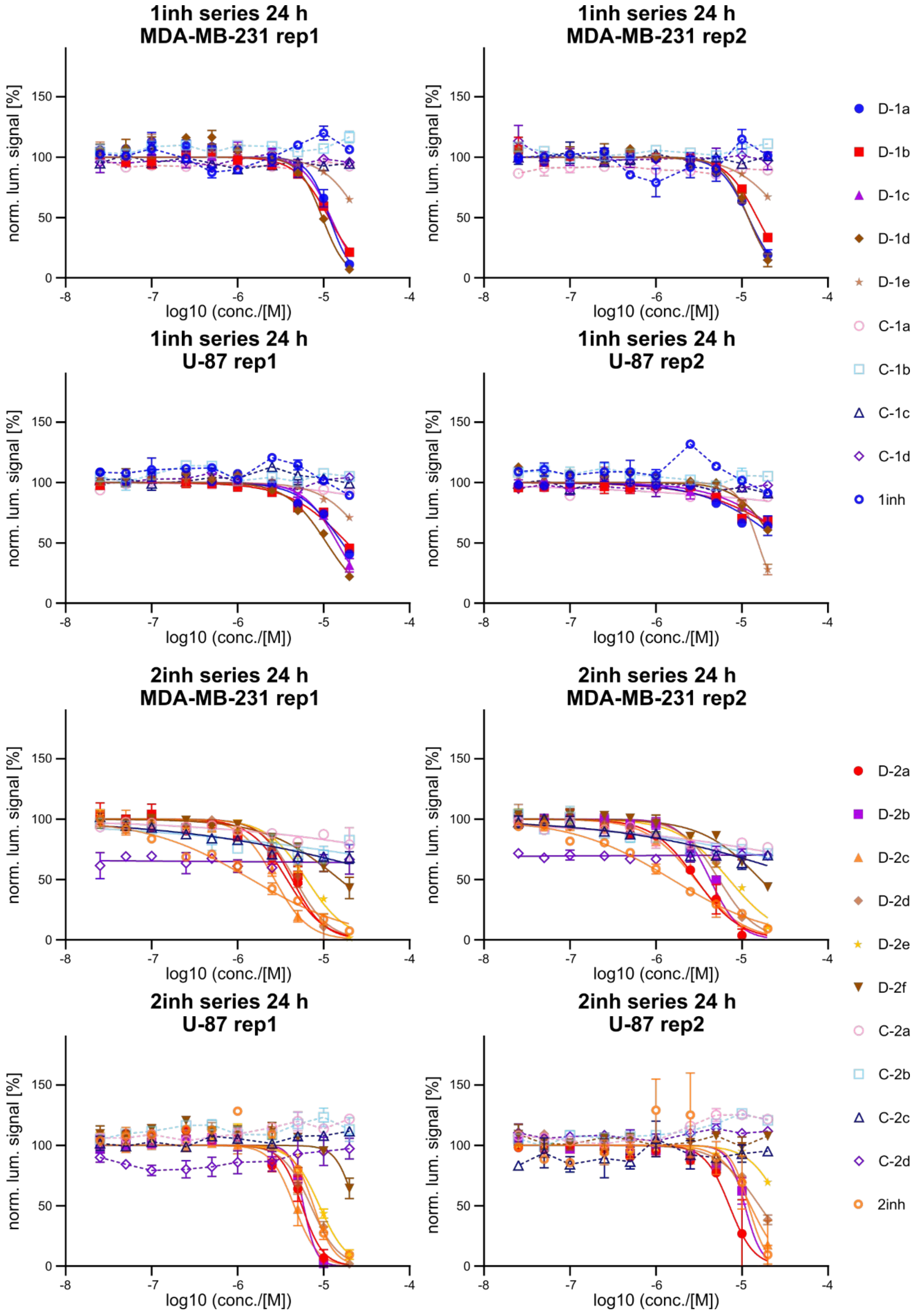

Figure S8: *Cell viability data (CellTiter Glo) of all promiscuous kinase PROTACs and respective kinase parent ligands. MDA-MB-231 and U-87 cells were treated in a 10-point dose series of the indicated compounds for 24 h. Each curve represents the mean of technical replicates (n=2). Error bars indicate the standard deviation. Dotted lines connect datapoints for which no sigmoidal curve could be fitted.*

2.2. Negative Controls D-1a$^{n.c.}$ and D-2c$^{n.c.}$

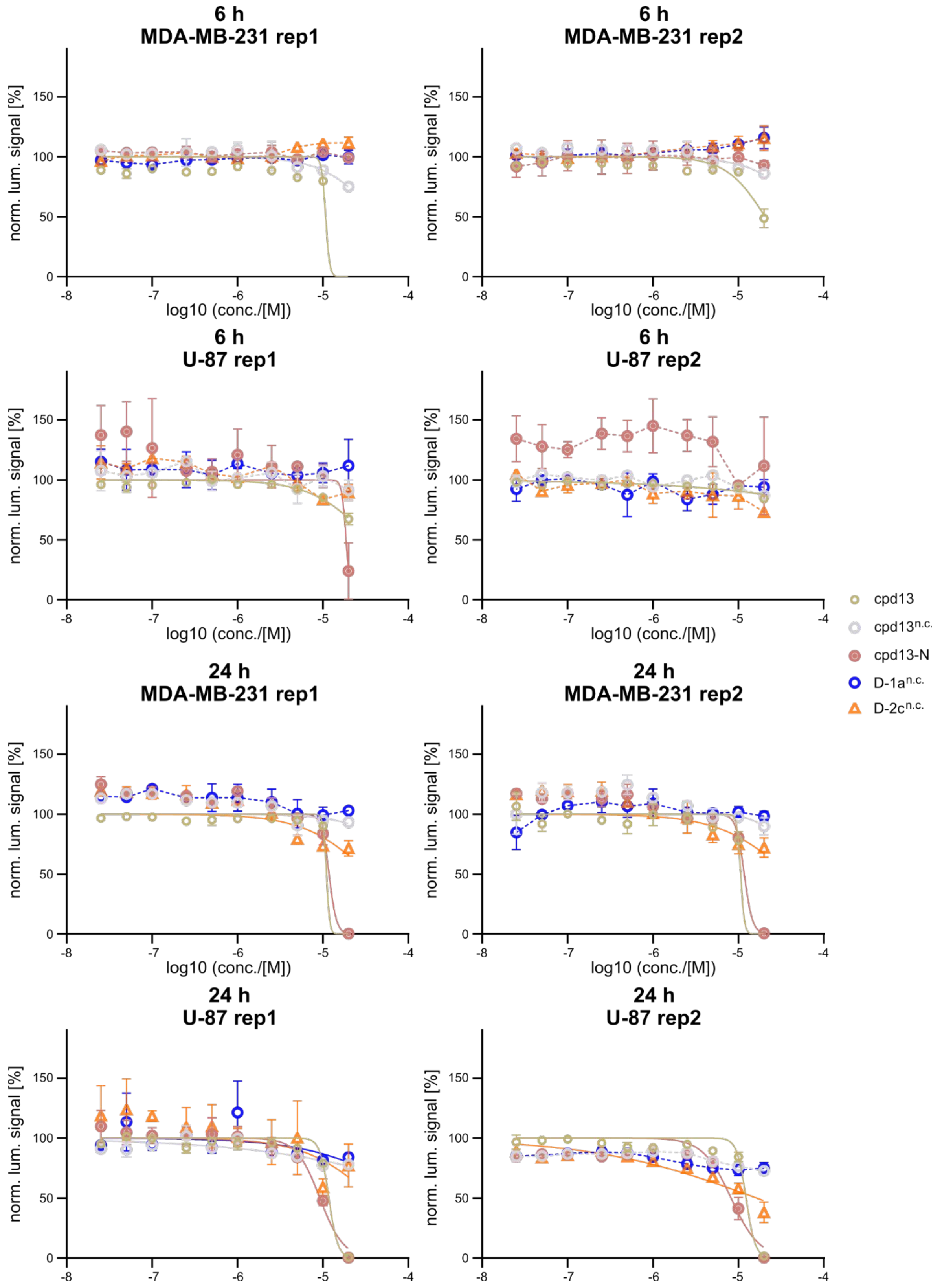

Figure S9: *Cell viability data (CellTiter Glo) of negative controls **D-1a$^{n.c.}$** and **D-2c$^{n.c.}$** and DCAF1-inactive ligands **cpd13$^{n.c.}$** and **cpd13-N** and active ligand **cpd13**. MDA-MB-231 and U-87 cells were treated in a 10-point dose series of the indicated compounds for 6 and 24 h, respectively. Each curve represents the mean of technical replicates*

(n=2). Error bars indicate the standard deviation. Dotted lines connect datapoints for which no sigmoidal curve could be fitted.

Table S3: *Cell viability data of all promiscuous kinase PROTACs, the respective kinase parent ligands, negative controls* ***D-1a***$^{n.c.}$ *and* ***D-2***$^{n.c.}$*, DCAF1-ligand* ***cpd13*** *and DCAF1-inactive ligands* ***cpd13***$^{n.c.}$ *and* ***cpd13-N.*** *MDA-MB-231 and U-87 cells were treated in 10-point doses of the indicated compound for 6 h and 24 h, respectively.* IC_{50} *values were calculated from biological replicates (n=4). Errors were calculated using the standard deviation.* [a]*Values were out of the assay window (*IC_{50} *> 25 µM).*

cmpd ID	IC_{50} ±sd µM] MDA-MB-231		IC_{50} ±sd µM] U-87	
	6 h	24 h	6 h	24 h
1inh	-	-	-	-[a]
D-1a	-	-	-	-
D-1a$^{n.c.}$	-	-	-	-
D-1b	-	13.3±2.6	-	-[a]
D-1c	-	13.9±2.5	-	-[a]
D-1d	-	10.8±1.6	-[a]	17.6±9.6
D-1e	-	-[a]	-	24.3±13
C-1a	-	-	-	-
C-1b	-	-	-	-
C-1c	-	-	-	-
C-1d	-	-	-	-
2inh	-	1.5±0.1	-	9.8±3.1
D-2a	7.7±3.4	3.4±0.6	-[a]	6.4±1.1
D-2b	9.5±3.7	4.3±2.6	-[a]	8.3±3.5
D-2c	11.7±5.3	2.8±0.2	-[a]	8.6±5.7
D-2c$^{n.c.}$	-	-[a]	-[a]	-[a]
D-2d	13.8±3.1	5.0±0.6	-[a]	11.6±6.4
D-2e	-[a]	6.7±0.6	-[a]	19.0±15
D-2f	-	16.0±0.8	-	-[a]
C-2a	-	-	-	-
C-2b	-	-	-	-
C-2c	-	-[a]	-	-
C-2d	-	-	-	-
cpd13	15.7±7.0	15.8±6.8	-[a]	12.0±0.4
cpd13-N	-[a]	11.8±0.1	-[a]	8.7±0.7
cpd13$^{n.c.}$	-[a]	-[a]	-[a]	-

3. MS-Proteomic Data

3.1. DCAF1-recruiting Promiscuous Kinase PROTACs

Table S4: *Number of kinases degraded by each PROTAC in respect to the indicated cell line with a moderated adjusted p-value < 0.01 and fold change threshold of* log_2*(fold change) ≤ -0.6.*

cmpd	no. of kinases		cmpd	no. of kinases	
ID	MDA-MB-231	U-87	ID	MDA-MB-231	U-87
**D-1a**	3	2	**C-1a**	4	5
**D-1b**	4	3	**C-1b**	0	0
**D-1c**	34	0	**C-1c**	4	1
**D-1d**	4	1	**C-1d**	5	0
**D-1e**	1	0	**C-2a**	16	31
**D-2a**	5	2	**C-2b**	15	37
**D-2b**	5	2	**C-2c**	26	11
**D-2c**	7	5	**C-2d**	42	64
**D-2d**	4	0			
**D-2e**	10	0			
**D-2f**	0	0			

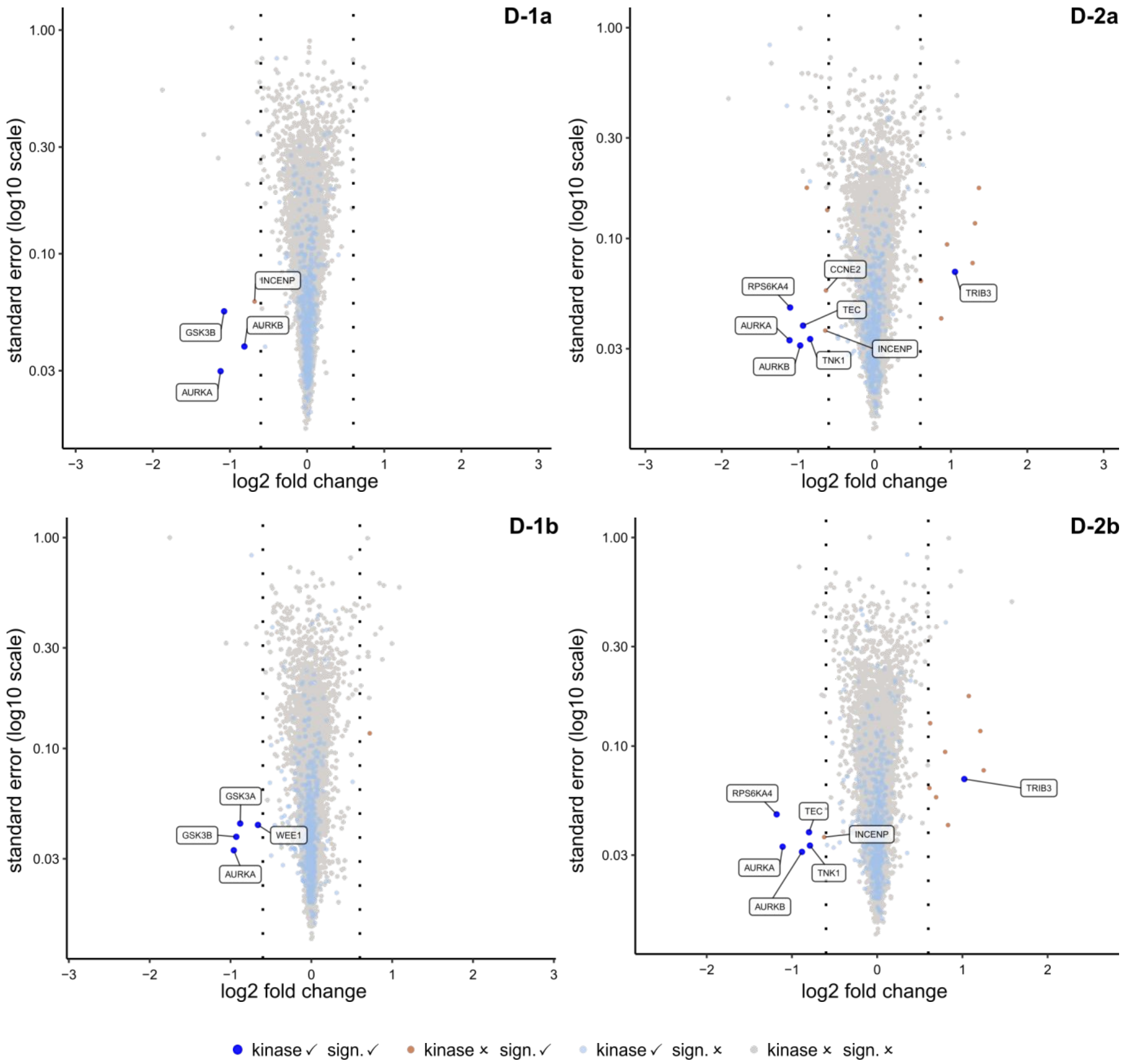

Figure S10: *Proteomics results. MDA-MB-231 cells were treated with 1 µM of the indicated PROTAC for 6 h. Significantly up- or downregulated kinase proteins are highlighted with blue dots and labelled accordingly, whereas non-kinase proteins are represented by orange dots (moderated adjusted p-value < 0.01; log_2 (fold change) ≥ 0.6 or ≤ -0.6). Proteins without significantly altered expression levels are highlighted in light blue (kinases) and grey (non-kinase proteins). Shown results are the mean of biological replicates (n=3). Data shown for* ***D-1a*** *is the same as in* ***Figure 4b****. The complete dataset can be found in supplementary file 2.*

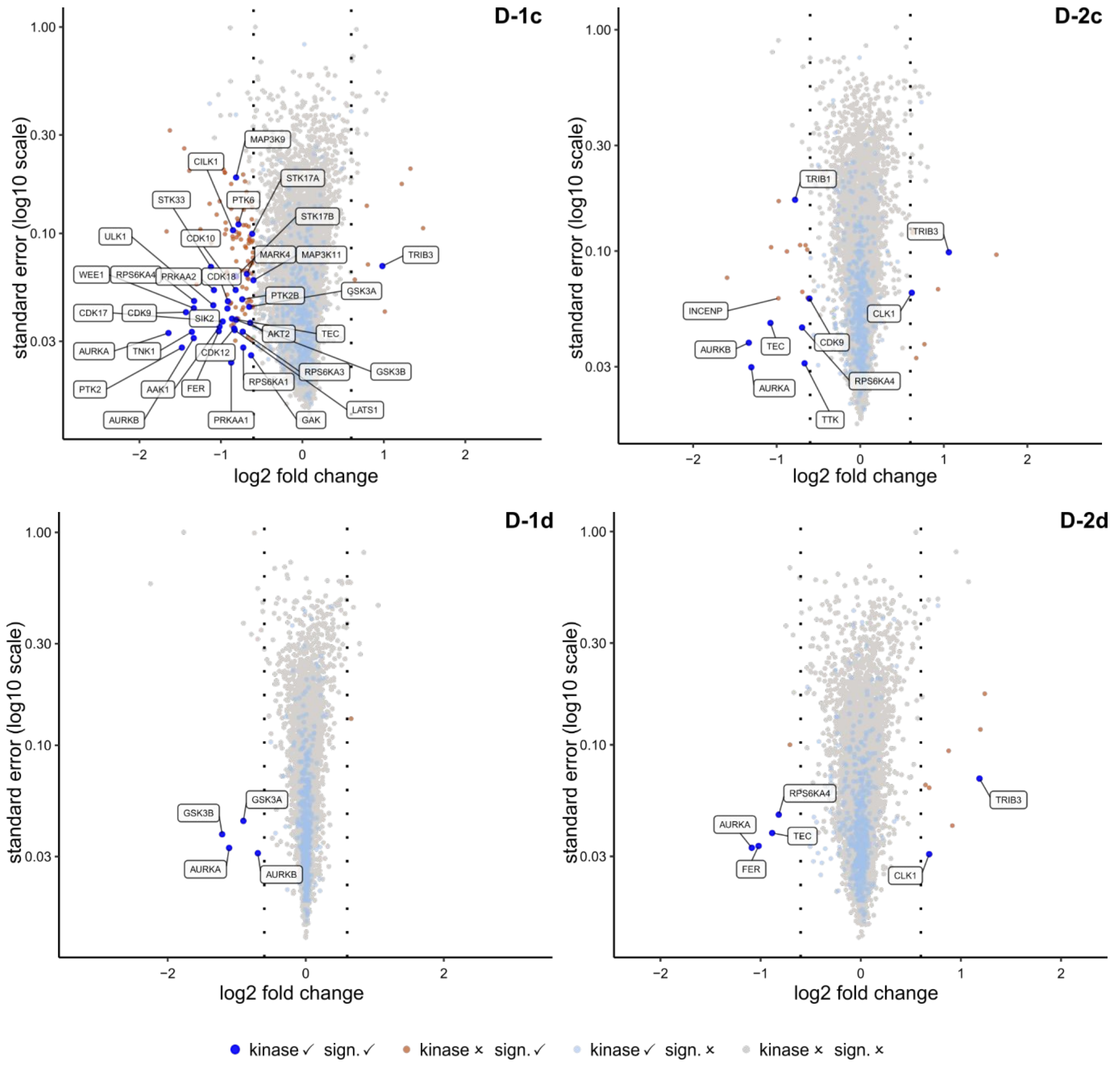

Figure S11: *Proteomics results. MDA-MB-231 cells were treated with 1 µM of the indicated PROTAC for 6 h. Significantly up- or downregulated kinase proteins are highlighted with blue dots and labelled accordingly, whereas non-kinase proteins are represented by orange dots (moderated adjusted p-value < 0.01; log_2 (fold change) ≥ 0.6 or ≤ -0.6). Proteins without significantly altered expression levels are highlighted in light blue (kinases) and grey (non-kinase proteins). Shown results are the mean of biological replicates (n=3). Data shown for* ***D-2c*** *is the same as in* ***Figure 4b****. The complete dataset can be found in supplementary file 2.*

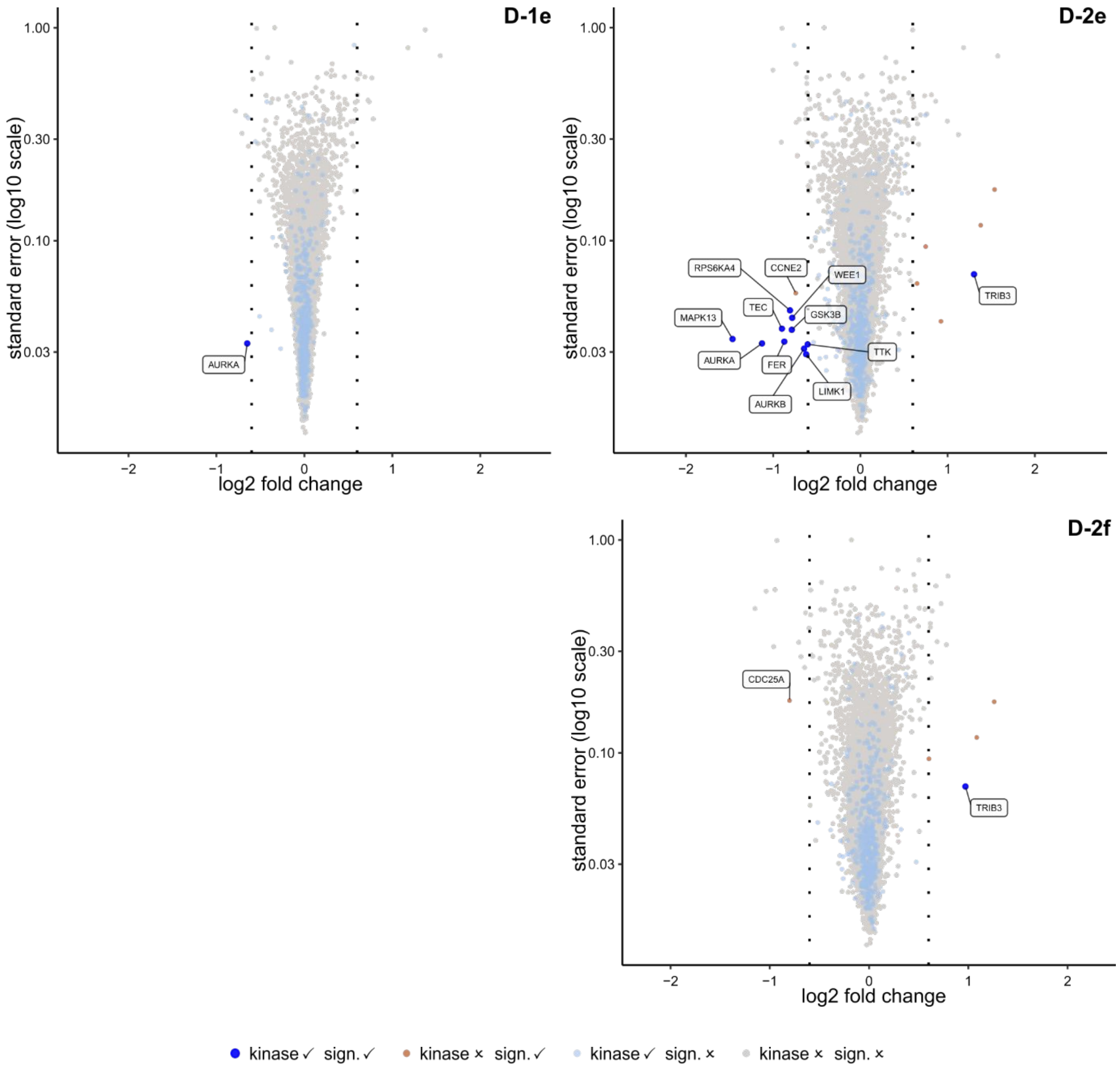

Figure S12: *Proteomics results. MDA-MB-231 cells were treated with 1 µM of the indicated PROTAC for 6 h. Significantly up- or downregulated kinase proteins are highlighted with blue dots and labelled accordingly, whereas non-kinase proteins are represented by orange dots (moderated adjusted p-value < 0.01;* log_2 *(fold change) ≥ 0.6 or ≤ -0.6). Proteins without significantly altered expression levels are highlighted in light blue (kinases) and grey (non-kinase proteins). Shown results are the mean of biological replicates (n=3). The complete dataset can be found in supplementary file 2.*

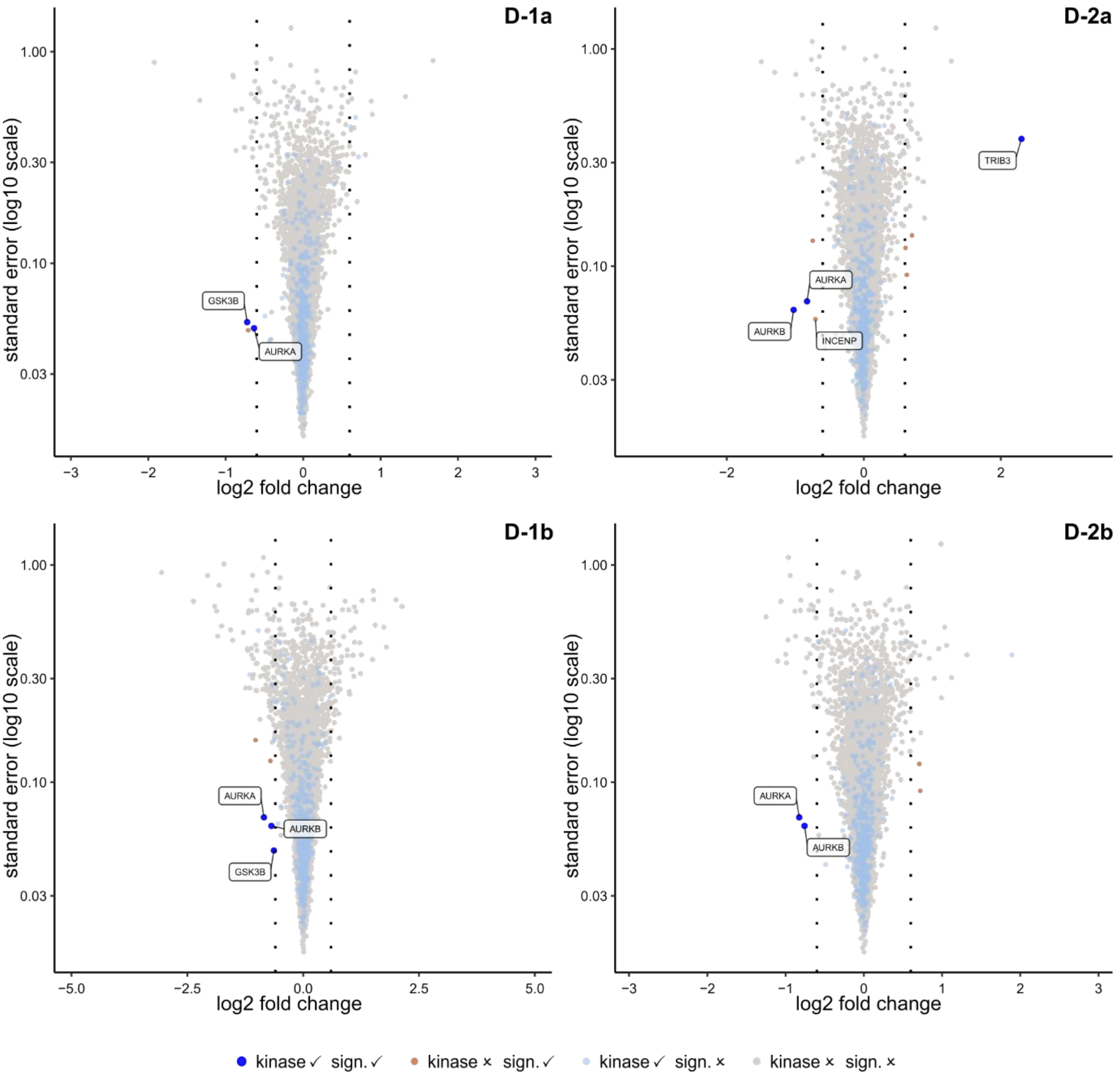

Figure S13: *Proteomics results. U-87 cells were treated with 1 µM of the indicated PROTAC for 6 h. Significantly up- or downregulated kinase proteins are highlighted with blue dots and labelled accordingly, whereas non-kinase proteins are represented by orange dots (moderated adjusted p-value < 0.01; $\log_2$ (fold change) ≥ 0.6 or ≤ -0.6). Proteins without significantly altered expression levels are highlighted in light blue (kinases) and grey (non-kinase proteins). Shown results are the mean of biological replicates (n=3). The complete dataset can be found in supplementary file 2.*

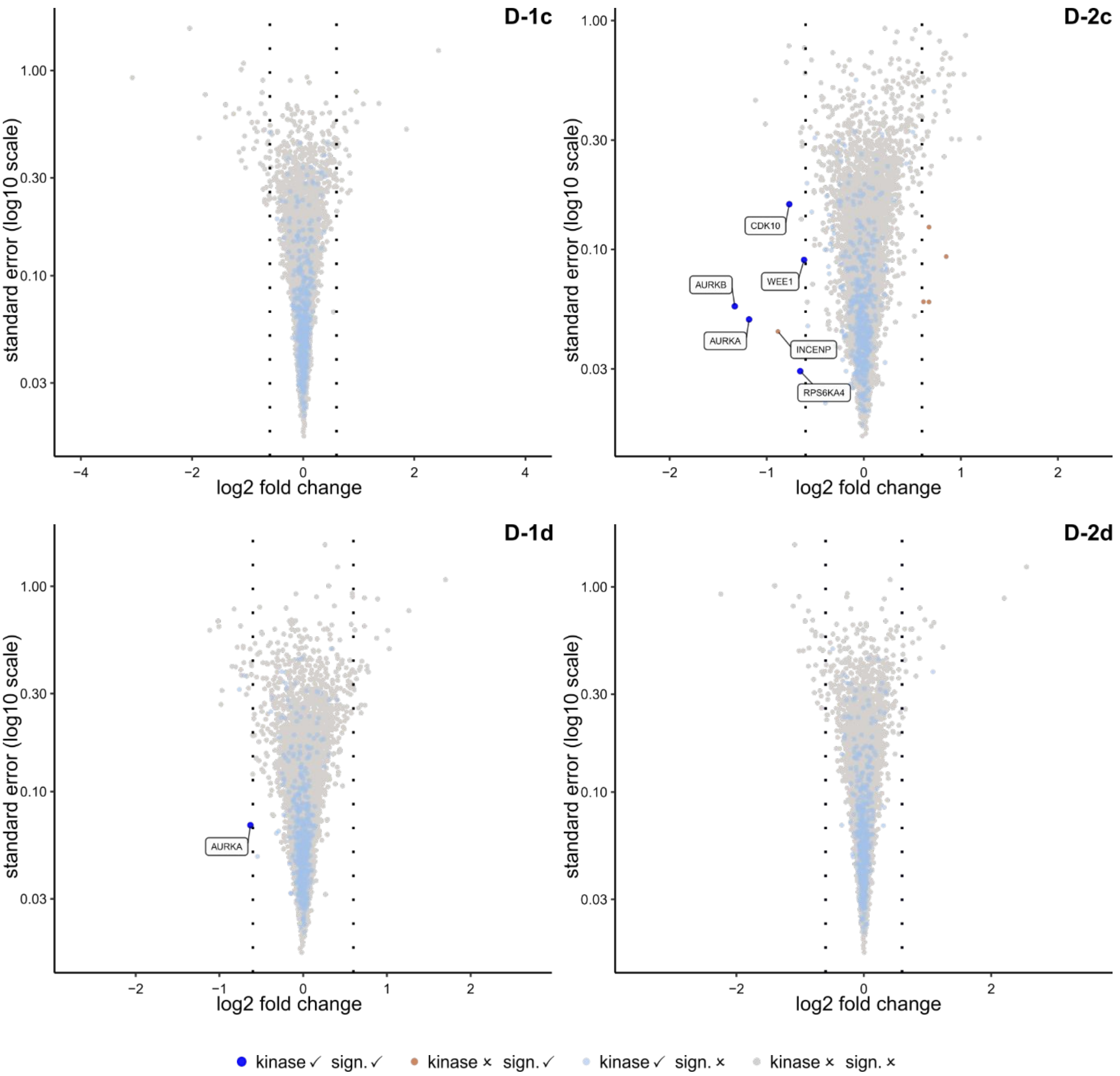

Figure S14: *Proteomics results. U-87 cells were treated with 1 µM of the indicated PROTAC for 6 h. Significantly up- or downregulated kinase proteins are highlighted with blue dots and labelled accordingly, whereas non-kinase proteins are represented by orange dots (moderated adjusted p-value < 0.01; log_2 (fold change) ≥ 0.6 or ≤ -0.6). Proteins without significantly altered expression levels are highlighted in light blue (kinases) and grey (non-kinase proteins). Shown results are the mean of biological replicates (n=3). The complete dataset can be found in supplementary file 2.*

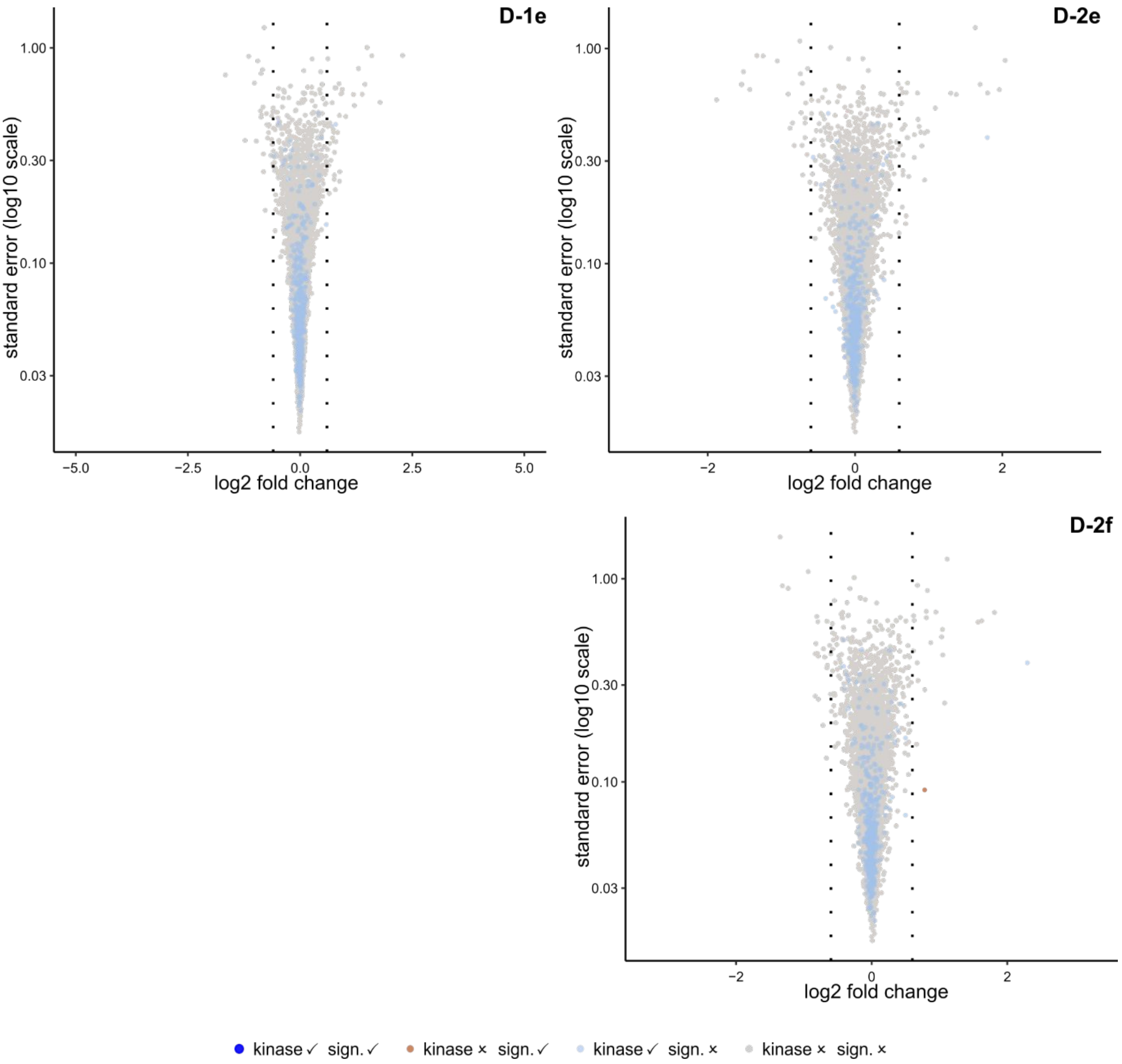

Figure S15: *Proteomics results. U-87 cells were treated with 1 µM of the indicated PROTAC for 6 h. Significantly up- or downregulated kinase proteins are highlighted with blue dots and labelled accordingly, whereas non-kinase proteins are represented by orange dots (moderated adjusted p-value < 0.01;* log_2 *(fold change) ≥ 0.6 or ≤ -0.6). Proteins without significantly altered expression levels are highlighted in light blue (kinases) and grey (non-kinase proteins). Shown results are the mean of biological replicates (n=3). The complete dataset can be found in supplementary file 2.*

Table S5: *Log$_2$ Fold changes of selected kinases degraded by PROTAC **D-1a** and/or **D-2c** in U-87 and MDA-MB-231 cells, respectively. Cell line-restricted kinase expression levels were compared by analysis of according DMSO control samples (log$_2$ fold changes U-87/MDA-MB-231).*

kinase	D-1a MDA-MB-231	D-1a U-87	U-87/ MDA-MB-231	D-2c MDA-MB-231	D-2c U-87
**AURKA**	-1.12	-0.63	-	-1.30	-1.18
**AURKB**	-0.81	-	-0.38	-1.33	-1.33
**CDK9**	-	-	-0.78	-0.61	-
**CDK10**	-	-	-0.81	-	-0.77
**GSK3B**	-1.08	-0.72	0.42	-	-
**RPS6KA4**	-	-	0.40	-0.70	-0.66
**TEC**	-	-	-1.34	-1.07	-
**TRIB1**	-	-	-2.86	-0.78	-
**TTK**	-	-	-0.63	-0.67	-
**WEE1**	-	-	-1.23	-	-0.62

3.2. Negative Controls

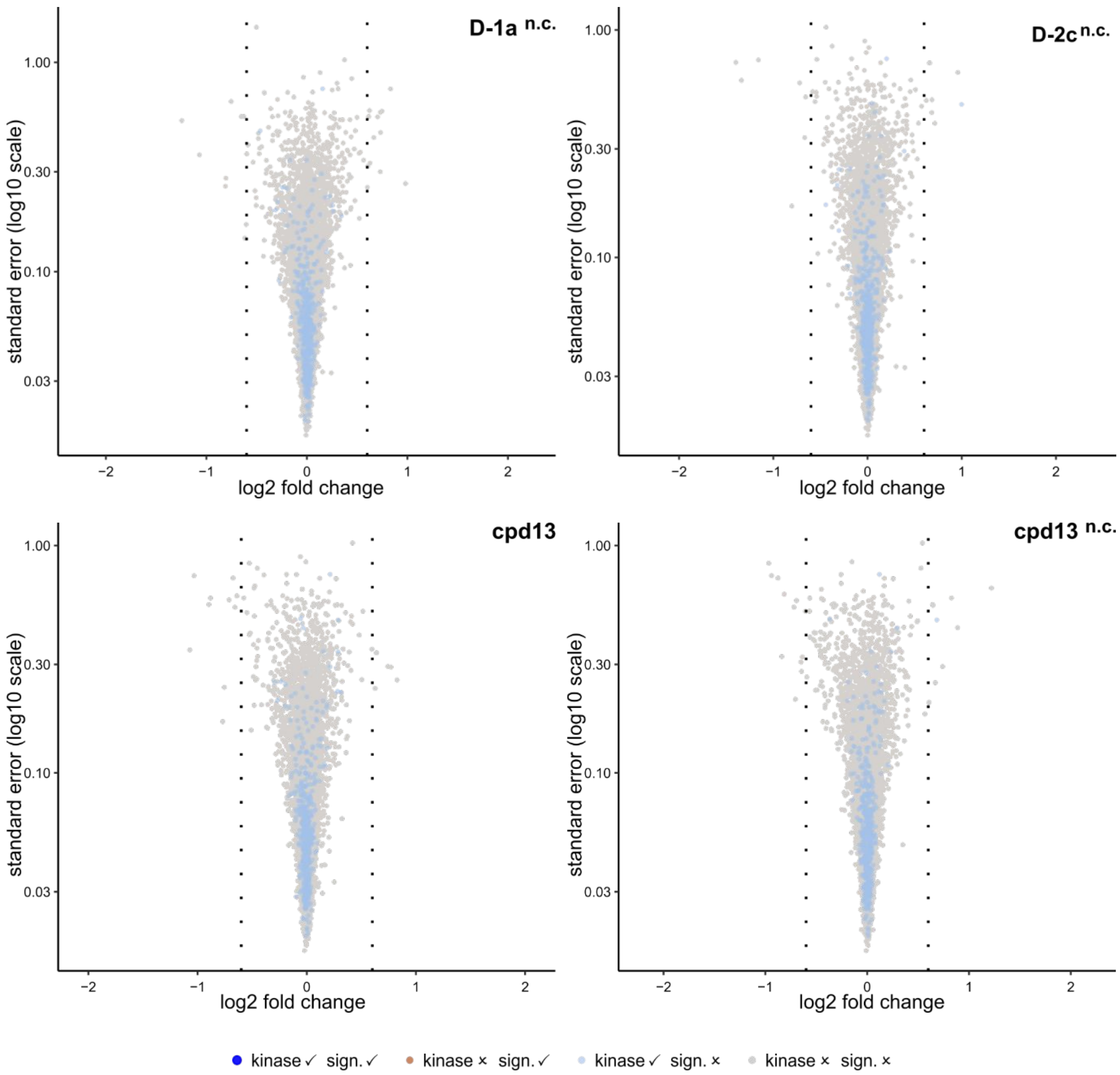

Figure S16: *Proteomics results. MDA-MB-231 cells were treated with 1 µM of the indicated compound for 6 h. Significantly up- or downregulated kinase proteins are highlighted with blue dots and labelled accordingly, whereas non-kinase proteins are represented by orange dots (moderated adjusted p-value < 0.01; $\log_2$ (fold change) ≥ 0.6 or ≤ -0.6). Proteins without significantly altered expression levels are highlighted in light blue (kinases) and grey (non-kinase proteins). Shown results are the mean of biological replicates (n=3). Data shown for* ***D-1a****$^{n.c.}$ and* ***D-2c****$^{n.c.}$ is the same as in* ***Figure 4c****. The complete dataset can be found in supplementary file 2.*

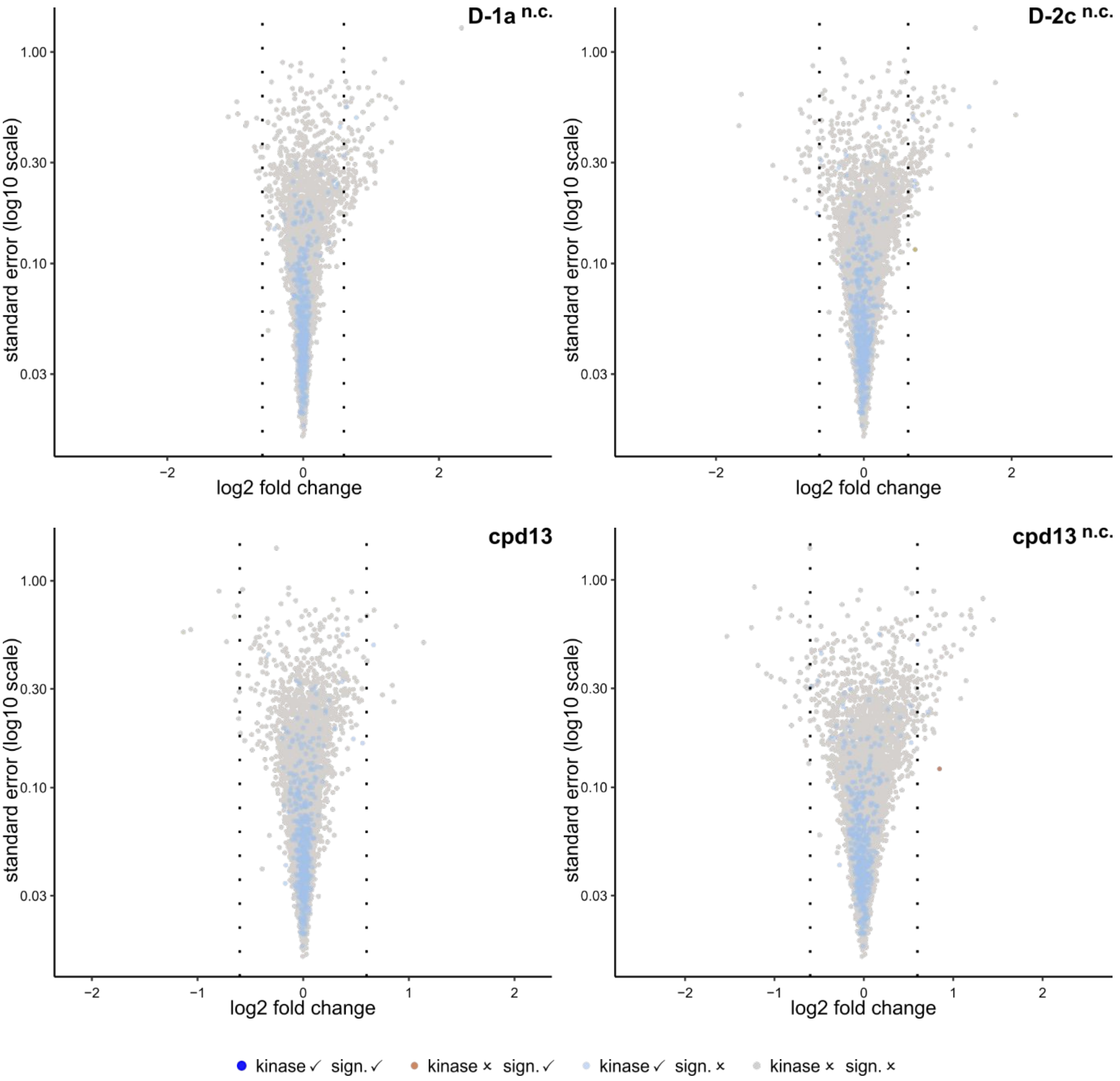

Figure S17: *Proteomics results. U-87 cells were treated with 1 µM of the indicated compound for 6 h. Significantly up- or downregulated kinase proteins are highlighted with blue dots and labelled accordingly, whereas non-kinase proteins are represented by orange dots (moderated adjusted p-value < 0.01; log_2 (fold change) ≥ 0.6 or ≤ -0.6). Proteins without significantly altered expression levels are highlighted in light blue (kinases) and grey (non-kinase proteins). Shown results are the mean of biological replicates (n=3). The complete dataset can be found in supplementary file 2.*

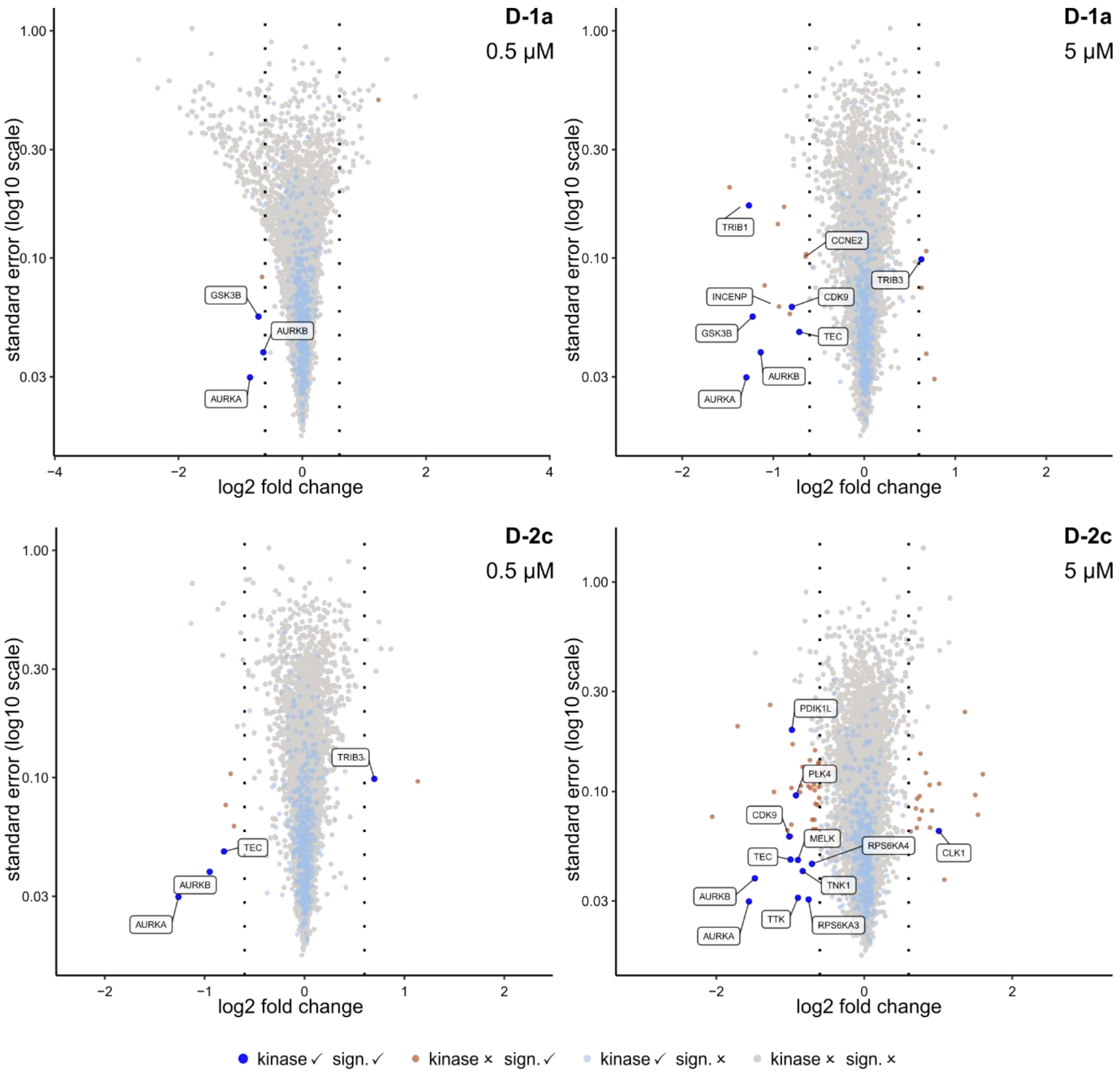

Figure S18: *Proteomics results. MDA-MB-231 cells were treated with 0.5 µM or 5.0 µM of the indicated PROTAC for 6 h. Significantly up- or downregulated kinase proteins are highlighted with blue dots and labelled accordingly, whereas non-kinase proteins are represented by orange dots (moderated adjusted p-value < 0.01; log_2 (fold change) ≥ 0.6 or ≤ -0.6). Proteins without significantly altered expression levels are highlighted in light blue (kinases) and grey (non-kinase proteins). Shown results are the mean of biological replicates (n=3). The complete dataset can be found in supplementary file 2.*

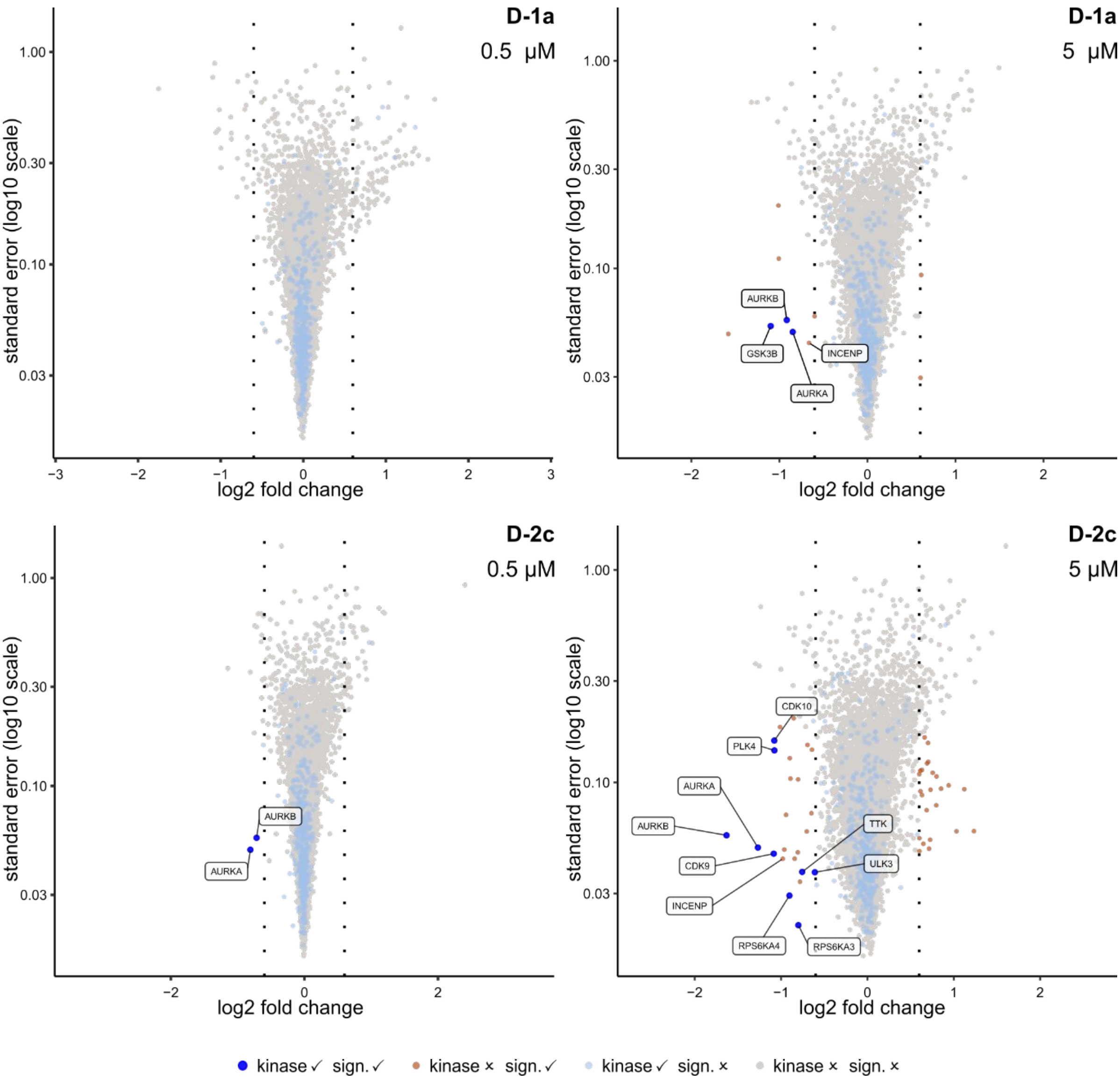

Figure S19: *Proteomics results. U-87 cells were treated with 0.5 µM or 5.0 µM of the indicated PROTAC for 6 h. Significantly up- or downregulated kinase proteins are highlighted with blue dots and labelled accordingly, whereas non-kinase proteins are represented by orange dots (moderated adjusted p-value < 0.01; log_2 (fold change) ≥ 0.6 or ≤ -0.6). Proteins without significantly altered expression levels are highlighted in light blue (kinases) and grey (non-kinase proteins). Shown results are the mean of biological replicates (n=3). The complete dataset can be found in supplementary file 2.*

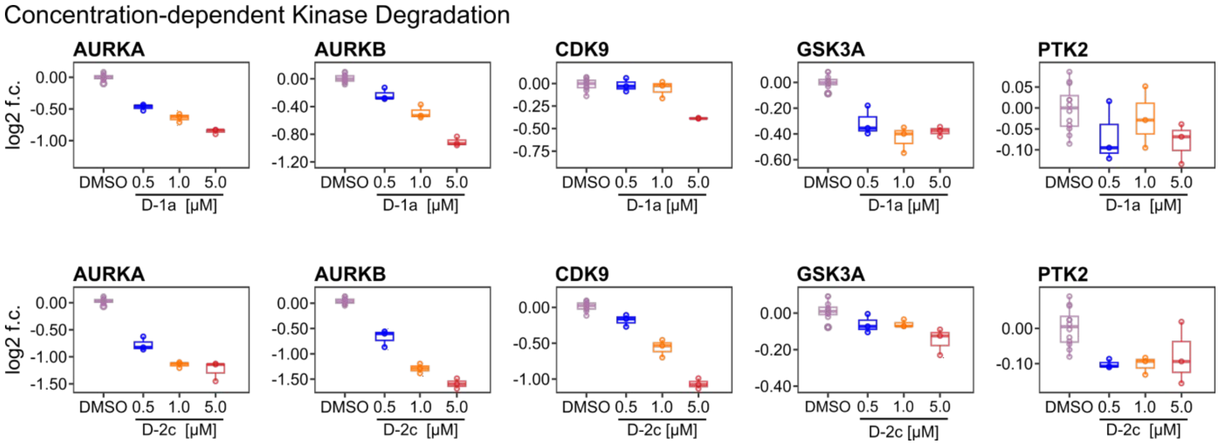

Figure S20: *DCAF1-based PROTACs **D-1a** and **D-2c** induced dose-dependent kinase degradation. Dose-dependent log_2 fold changes (proteomics) for selected kinases induced by PROTACs **D-1a** and **D-2c** in U-87 cells.*

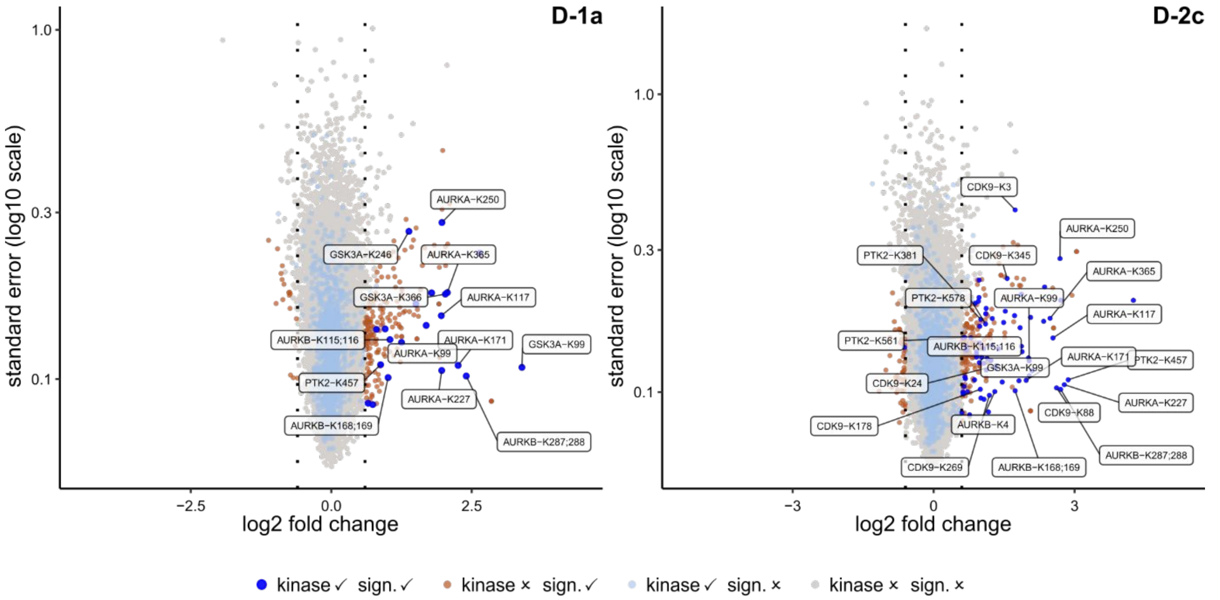

Figure S21: *Ubiquitination events correlate with degradation events. MDA-MB-231 cells were treated with 1 μM of either PROTAC **D-1a** or **D-2c** for 30 min. Significantly up- or downregulated ubiquitinated peptides of non-kinase proteins are highlighted with orange dots whereas peptides of protein kinases are shown with blue dots and are labelled (moderated adjusted p-value < 0.01; log_2 fold change ≤ -0.6 or ≥ +0.6). Levels of peptides of proteins that were not significantly changed are shown in light blue (kinases) and grey (non-kinase proteins). Shown results are the mean of biological replicates (n=4). The complete dataset can be found in supplementary file 2.*

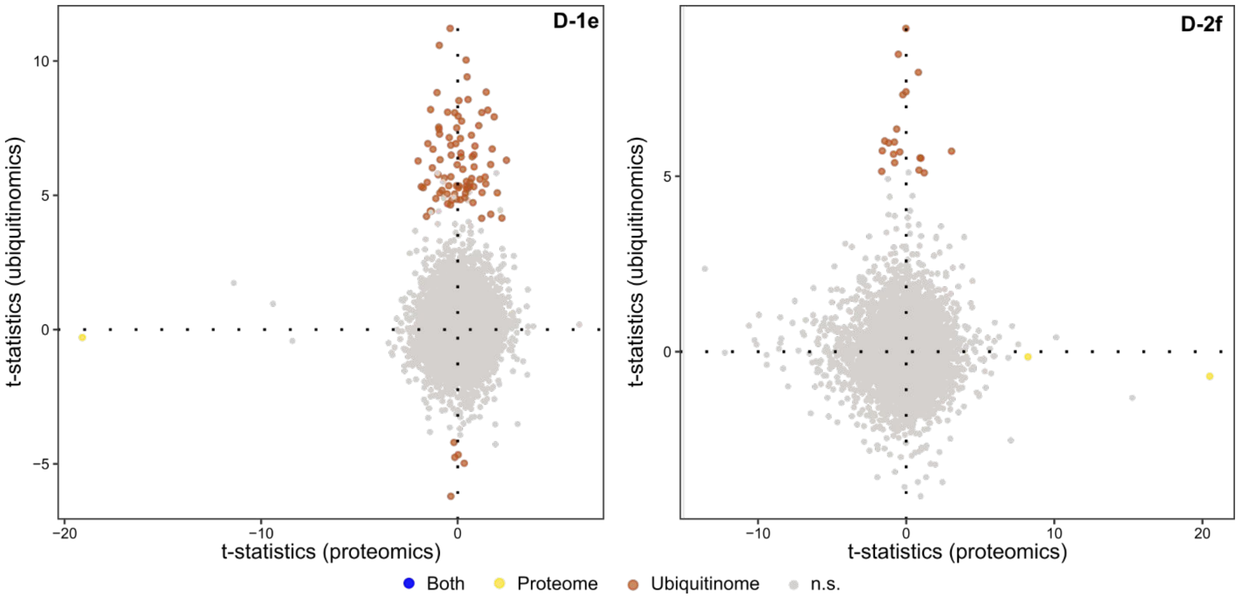

Figure S22: *T-statistic comparison of the changes of the ubiquitinome and proteome for* ***D-1e*** *and* ***D-2f*** *in MDA-MB-231 cells (1 µM, proteomics: 6 h, ubiquitinomics: 30 min).*

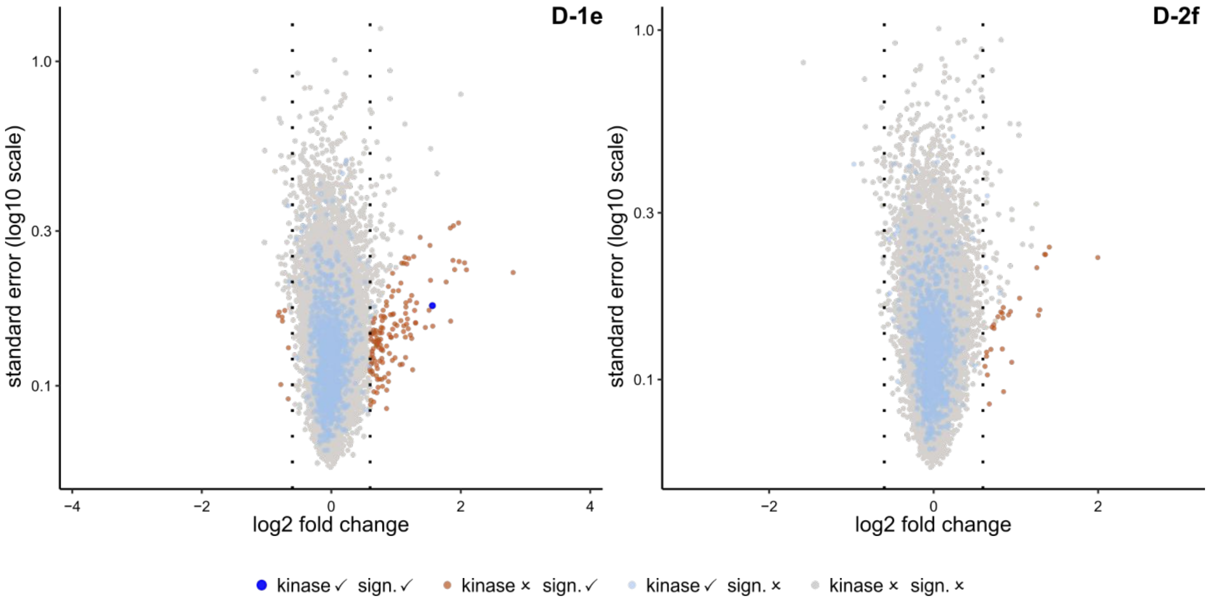

Figure S23: *Inactive PROTACs do not significantly alter kinase ubiquitination levels. MDA-MB-231 cells have been treated 1 µM of either* ***D-1e*** *or* ***D-2f*** *for 30 min. Significantly up- or downregulated ubiquitinated peptides of non-kinase proteins are highlighted with orange dots whereas peptides of protein kinases are shown with blue dots and are labelled (moderated adjusted p-value < 0.01; log_2 fold change ≤ -0.6 or ≥ +0.6). Levels of peptides of proteins that were not significantly changed are shown in light blue (kinases) and grey (non-kinase proteins). Shown results are the mean of biological replicates (n=4). The complete dataset can be found in supplementary file 2.*

3.3. CRBN-recruiting Promiscuous Kinase PROTACs

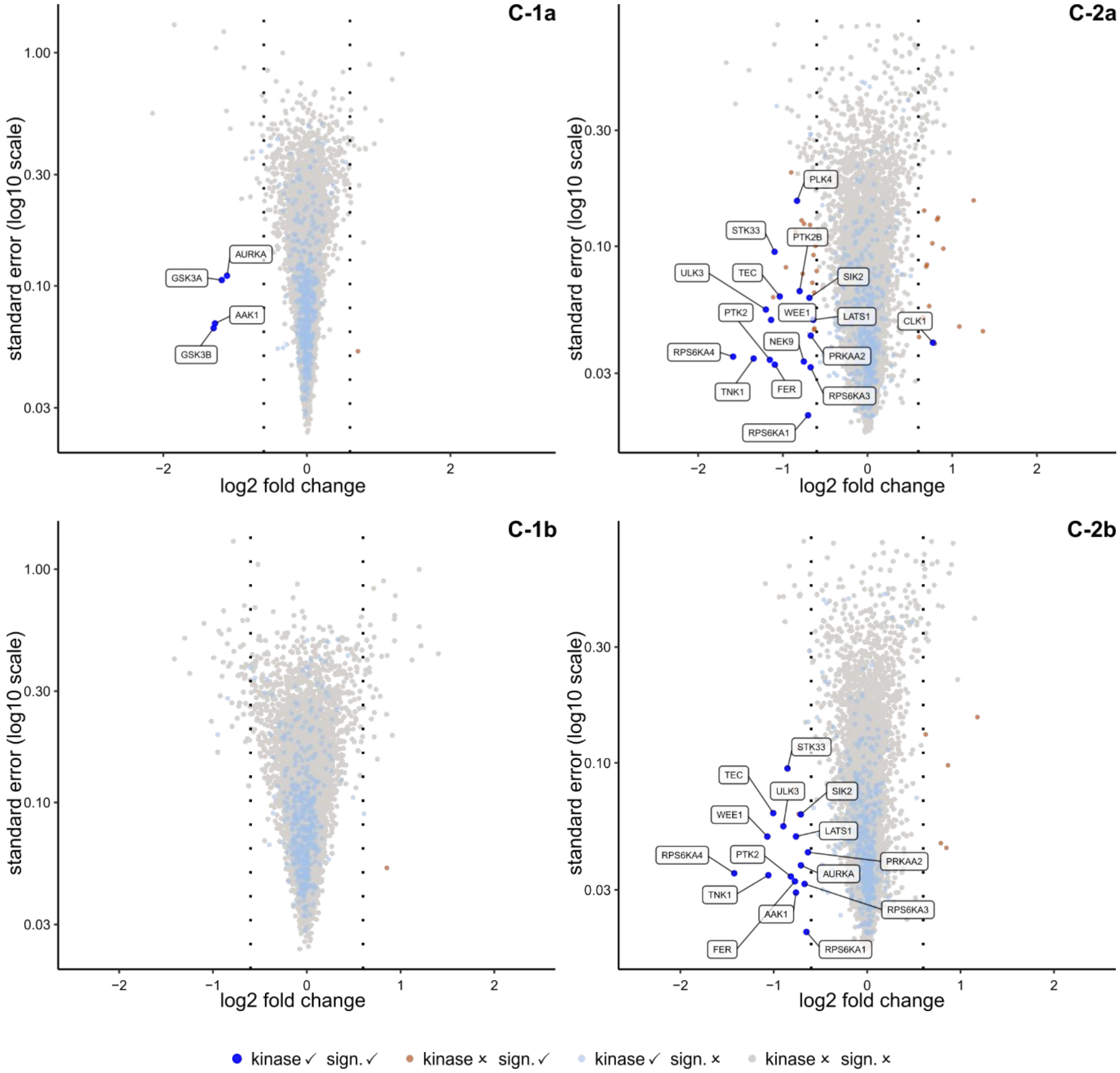

Figure S24: *Proteomics results. MDA-MB-231 cells have been treated 1 µM of either* ***C-1a****,* ***C-1b****,* ***C-2a*** *or* ***C-2b*** *for 6 h. Significantly up- or downregulated non-kinase proteins are highlighted with orange dots whereas protein kinases are shown with blue dots and are labelled (moderated adjusted p-value < 0.01; log_2 fold change ≤ -0.6 or ≥ +0.6). Levels of proteins that were not significantly changed are shown in light blue (kinases) and grey (non-kinase proteins). Shown results are the mean of biological replicates (n=3). Data shown for* ***C-1a*** *and* ***C-2a*** *is the same as in* ***Figure 6a****. The complete dataset can be found in supplementary file 2.*

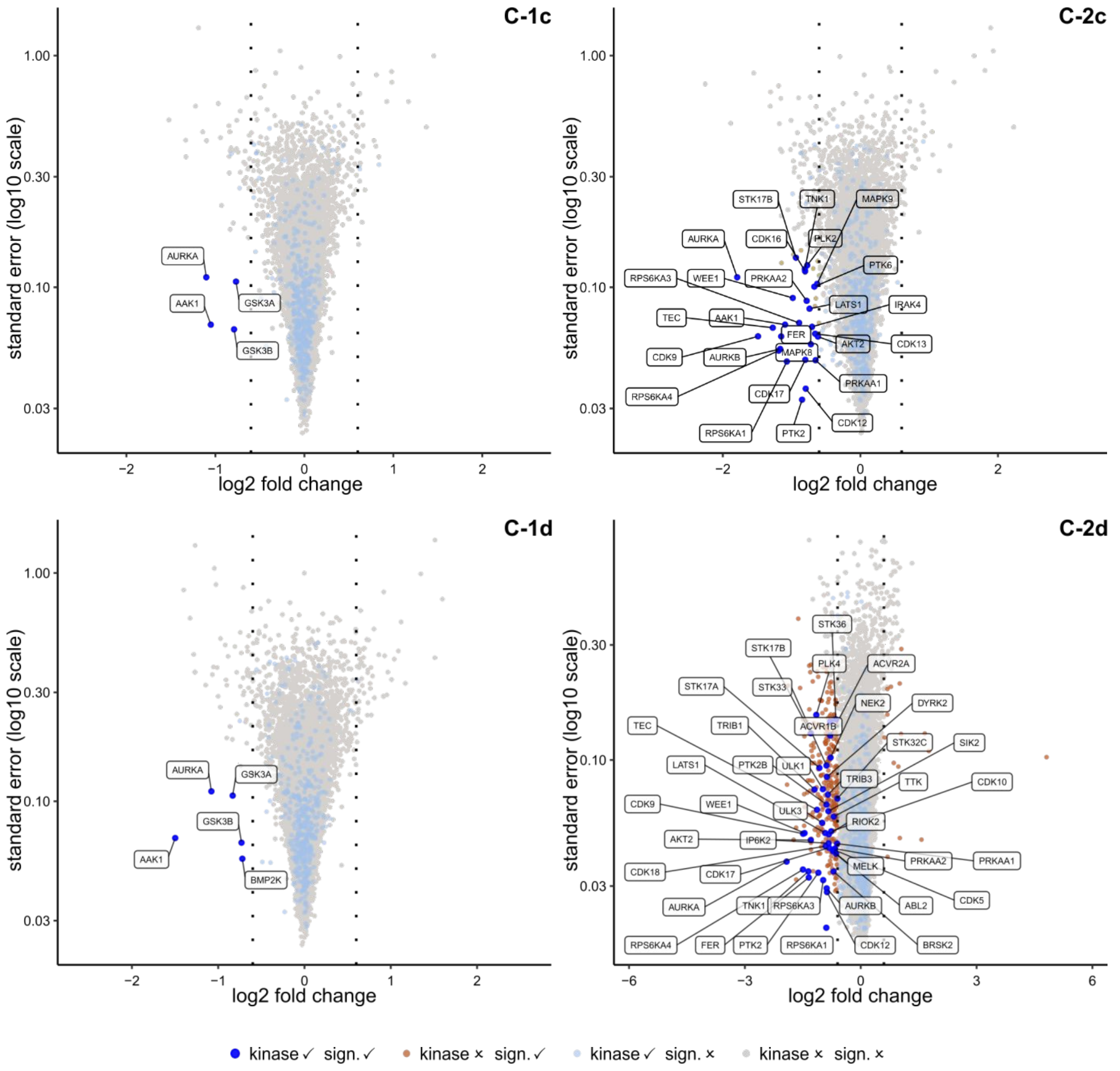

Figure S25: *Proteomics results. MDA-MB-231 cells have been treated 1 µM of either* ***C-1c****,* ***C-1d****,* ***C-2c****, or* ***C-2d*** *for 6 h. Significantly up- or downregulated non-kinase proteins are highlighted with orange dots whereas protein kinases are shown with blue dots and are labelled (moderated adjusted p-value < 0.01; log_2 fold change ≤ -0.6 or ≥ +0.6). Levels of proteins that were not significantly changed are shown in light blue (kinases) and grey (non-kinase proteins). Shown results are the mean of biological replicates (n=3). The complete dataset can be found in supplementary file 2.*

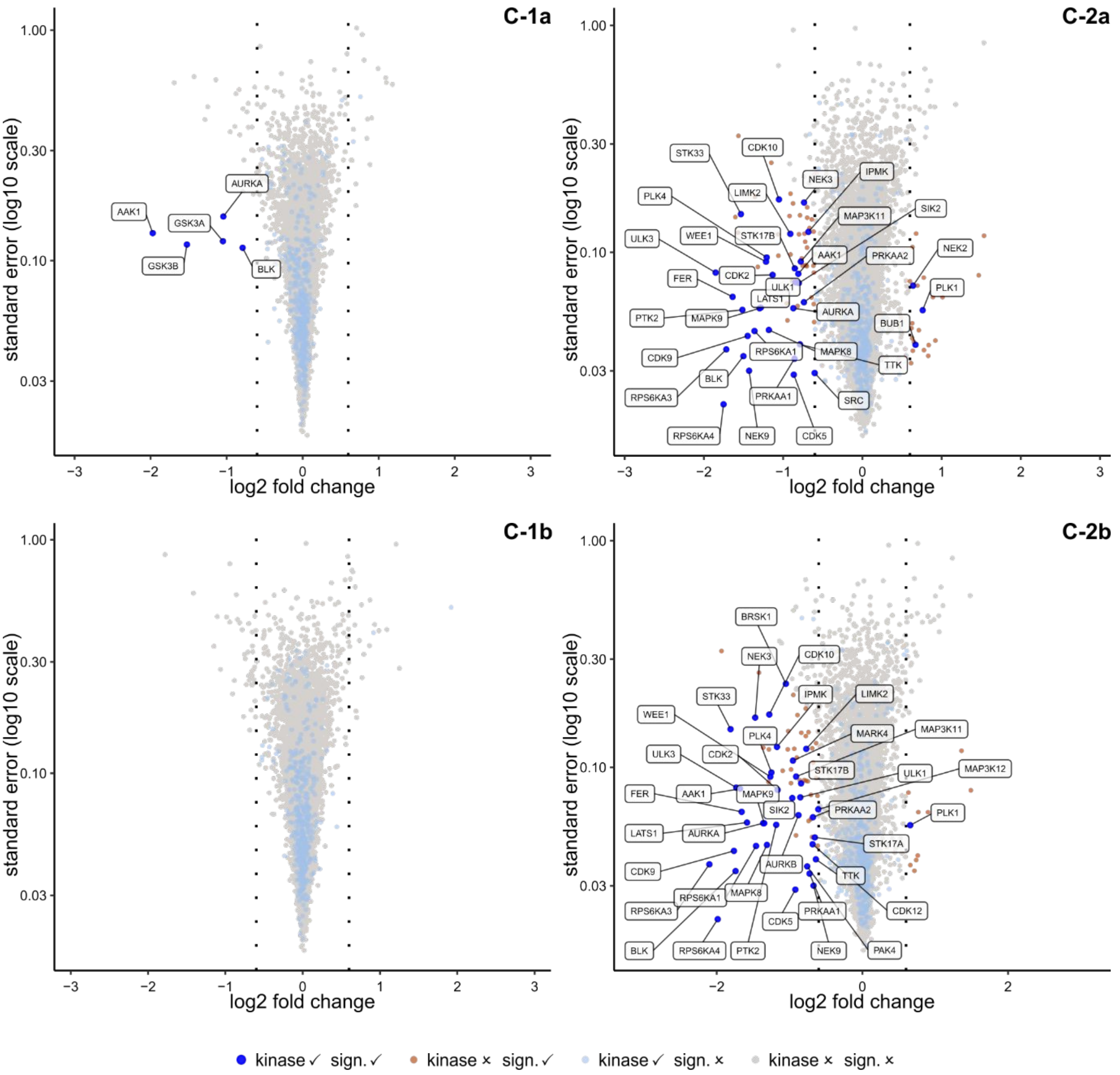

Figure S26: *Proteomics results. U-87 cells have been treated with 1 µM of either **C-1a**, **C-1b**, **C-2a or C-2b** for 6 h. Significantly up- or downregulated non-kinase proteins are highlighted with orange dots whereas protein kinases are shown with blue dots and are labelled (moderated adjusted p-value < 0.01; log_2 fold change ≤ -0.6 or ≥ +0.6). Levels of proteins that were not significantly changed are shown in light blue (kinases) and grey (non-kinase proteins). Shown results are the mean of biological replicates (n=3). The complete dataset can be found in supplementary file 2.*

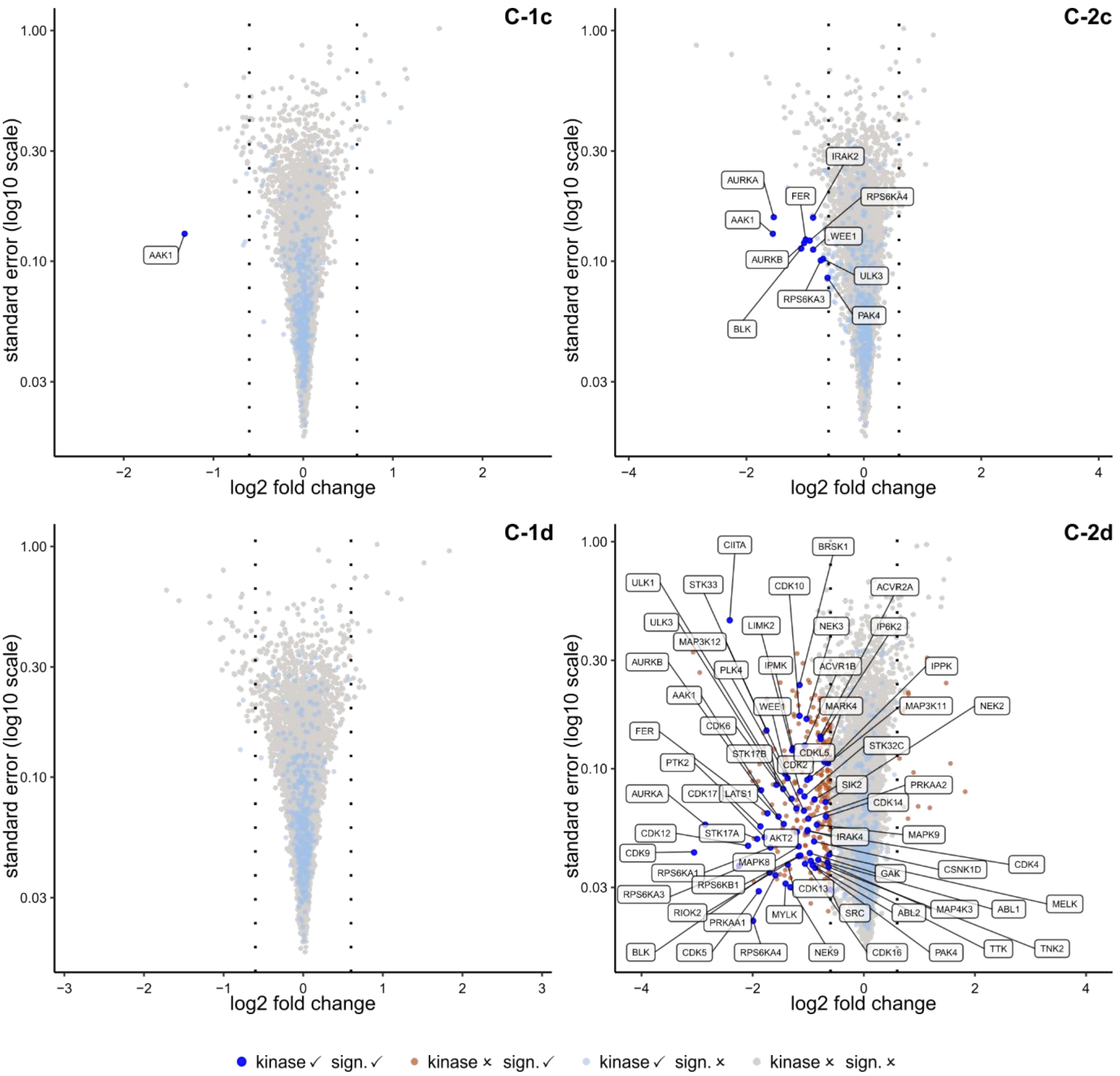

Figure S27: *Proteomics results. U-87 cells have been treated with 1 µM of either* ***C-1c****,* ***C-1d****,* ***C-2c*** *or* ***C-2d*** *for 6 h. Significantly up- or downregulated non-kinase proteins are highlighted with orange dots whereas protein kinases are shown with blue dots and are labelled (moderated adjusted p-value < 0.01; log_2 fold change ≤ -0.6 or ≥ +0.6). Levels of proteins that were not significantly changed are shown in light blue (kinases) and grey (non-kinase proteins). Shown results are the mean of biological replicates (n=3). The complete dataset can be found in supplementary file 2.*

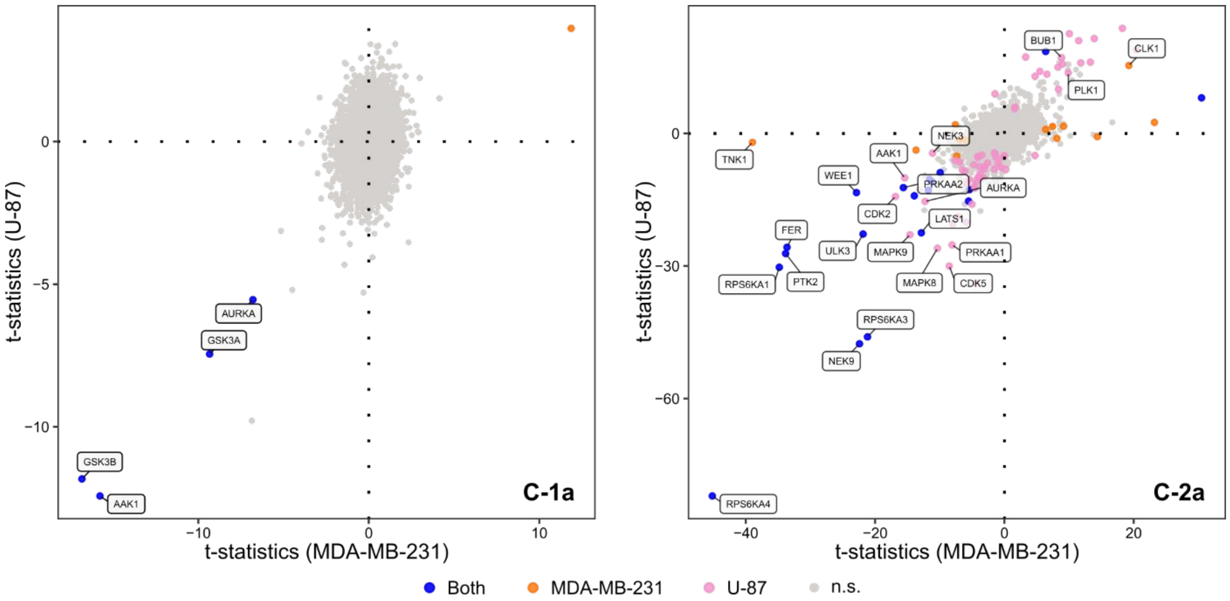

Figure S28: *T-statistic comparison of the degradation in U-87 and MDA-MB-231 cells for* ***C-1a*** *and* ***C-2a*** *(proteomics 1 µM, 6 h).*

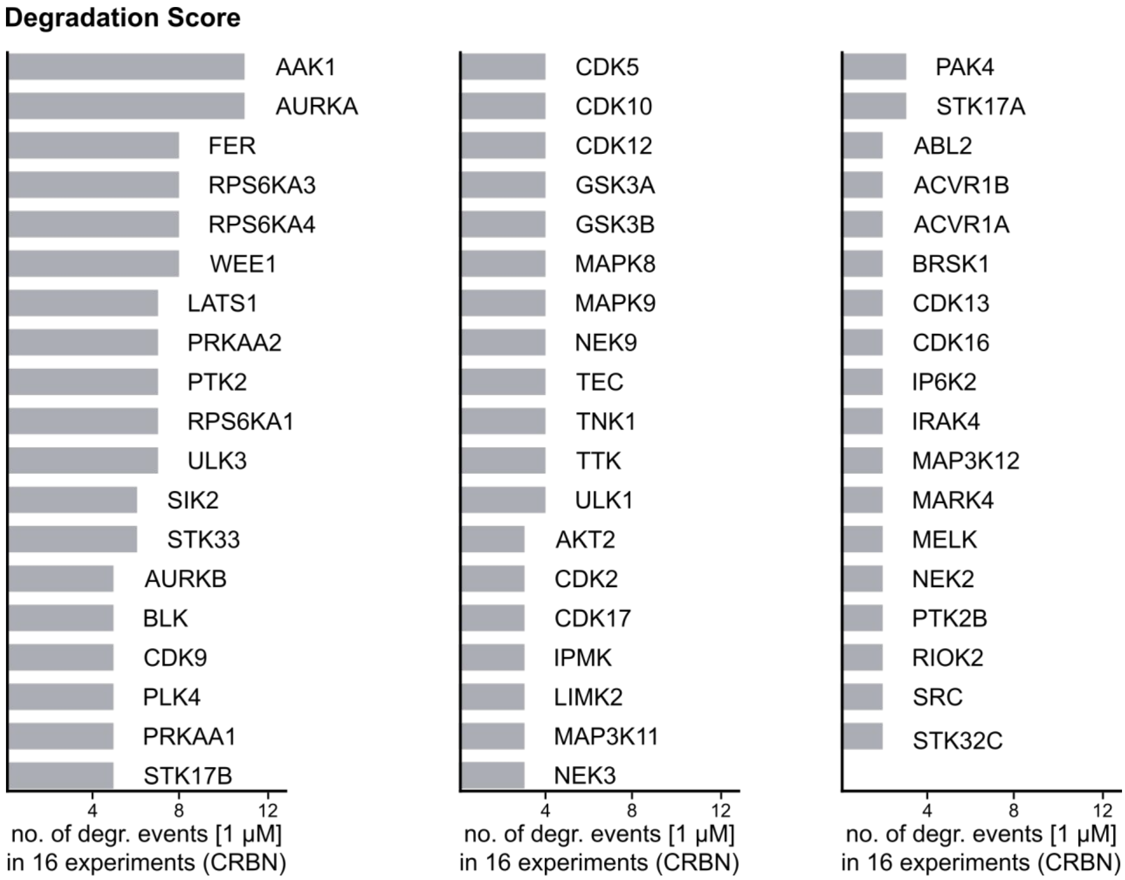

Figure S29: *Number of observed degradation events for each kinase in a total of 16 proteomics experiments (moderated adjusted p-value < 0.01; log_2 fold change ≤ -0.6) with CRBN-recruiting PROTACs. All kinases that were degraded in at least two experiments are listed.*

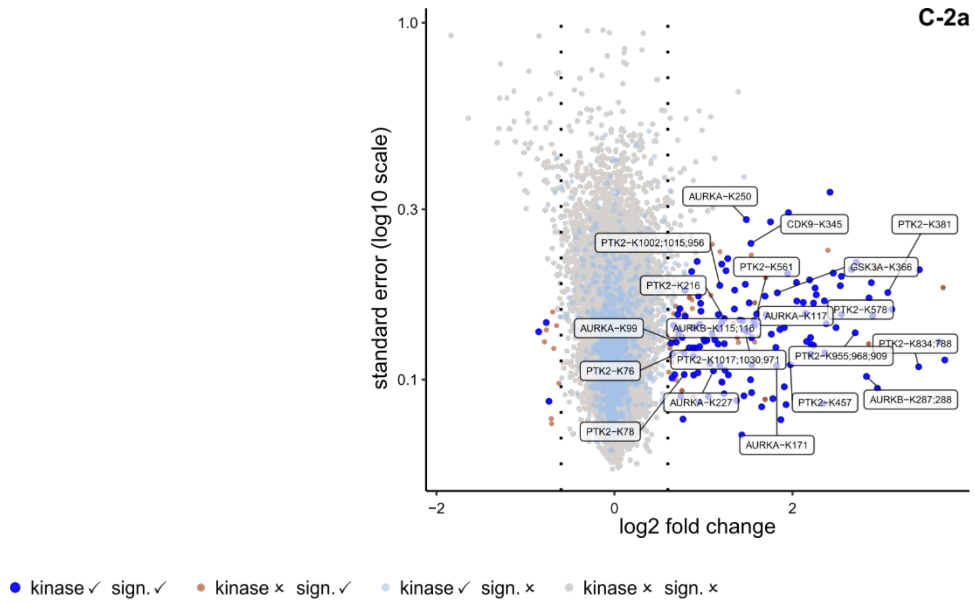

Figure S30: *Ubiquitinomics results. MDA-MB-231 cells have been treated 1 µM of* ***C-2a*** *for 30 min. Significantly up- or downregulated ubiquitinated peptides of non-kinase proteins are highlighted with orange dots whereas peptides of protein kinases are shown with blue dots and are labelled (moderated adjusted p-value < 0.01; log_2 fold change ≤ -0.6 or ≥ 0.6). Levels of peptides of proteins that were not significantly changed are shown in light blue (kinases) and grey (non-kinase proteins). Shown results are the mean of biological replicates (n=4). The complete dataset can be found in supplementary file 2.*

	AURKA			AURKB		
	D-2c	D-1a	C-2a	D-2c	D-1a	C-2a
f.c. prot.	-1.30	-1.12	-0.47	-1.33	-0.81	-0.36
modified lysines (f.c.)	K99 (2.03)	K99 (1.25)	K99 (0.70)	K4 (1.30)		
	K117 (2.54)	K117 (1.96)	K117 (1.71)	K115;116 (1.14)	K115;116 (1.04)	K115;116 (1.00)
	K143 (2.06) AURKA;B;C	K143 (2.85) AURKA;B;C		K87 (2.06) AURKA;B;C	K87 (2.85) AURKA;B;C	
	K171 (1.97)	K171 (2.26)	K171 (1.82)	K168;169 (1.74)	K168;169 (1.01)	K168;169 (0.46)
	K227 (2.78)	K227 (1.97)	K227 (1.11)	K231;232 (2.55) AURKB;C	K231;232 (1.92) AURKB;C	K231;232 (1.58) AURKB;C
	K250 (2.69)	K250 (1.97)	K250 (1.48)	K287;288 (2.70)	K287;288 (2.40)	K287;288 (2.83)
	K365 (2.48)	K365 (2.07)				

	AKT2			CDK10		
	D-2c	D-1a	C-2a	D-2c	D-1a	C-2a
f.c. prot.	-0.18	-	-0.34	-0.59	-	-0.39
modified lysines (f.c.)			K111 (1.28)	K356;285;279 (4.25)		K356;285;279 (3.43)
			K146 (1.96)			
	K165 (1.43) AKT2;AKT3					
			K191 (0.89)			

	CDK9			FER		
	D-2c	D-1a	C-2a	D-2c	D-1a	C-2a
f.c. prot.	-0.61	-	-0.21	-0.31	0.00	-1.10
modified lysines (f.c.)	K3 (1.74)			K420;245 (0.91)		K420;245 (2.20)
	K24 (1.33)					K475;300 (0.90)
	K88 (2.62)					K488;313 (2.19)
	K178 (0.99)					K541;366 (1.18)
	K269 (1.19)			K585;410 (2.34)		K585;410 (2.27)
	K345 (1.56)		K345 (1.53)	K594;419 (0.32)		K594;419 (0.77)
				K720;545 (1.52)		K720;545 (2.89)
				K791;616 (0.89)		K791;616 (2.46)

Figure S31: *Detailed analysis of lysine ubiquitination. The log_2 fold changes detected in the proteomics experiments (f.c. prot.) and lysine residue ubiquitination mediated by PROTACs **D-2c**, **D-1a** and **C-2a** are shown for selected kinases. Numbers in brackets indicate log_2 fold changes detected in the ubiquitinomics experiments.*

	MAPK8 D-2c	MAPK8 D-1a	MAPK8 C-2a	PTK2 D-2c	PTK2 D-1a	PTK2 C-2a
f.c. prot.	-0.17	-	-0.38	-0.12	-	-1.15
modified lysines (f.c.)	K24 (1.84)		K24 (2.12)			K76 (0.84)
	K250 (0.85) MAPK10;MAPK8		K250 (1.38) MAPK10;MAPK8			K78 (0.78)
						K216 (1.24)
			K308 (0.53)	K381 (1.00)		K381 (3.07)
				K457 (2.86)	K457 (0.88)	K457 (1.98)
				K561 (0.66)		K561 (1.60)
				K578 (1.11)		K578 (2.86)
				K834;788 (0.54)		K834;788 (3.42)
						K955;968;909 (2.71)
						K1002;1015;956 (1.18)
						K1017;1030;971 (1.46)

	RPS6KA1 D-2c	RPS6KA1 D-1a	RPS6KA1 C-2a	RPS6KA3 D-2c	RPS6KA3 D-1a	RPS6KA3 C-2a
**f.c. prot.**	-0.30	-	-0.70	-0.50	-	-0.67
modified lysines (f.c.)	K51;60;35 (0.62)		K51;60;35 (2.55)	K57 (1.17)		K57 (2.35)
			K56;65;40 (0.94)	K81 (1.12)		
	K75;84;59 (0.70) RPS6KA1;RPS6KA6		K75;84;59 (0.84) RPS6KA1;RPS6KA6	K322 (0.45)		K322 (0.74)
						K504 (0.70) RPS6KA1;RPS6KA3
	K276;285;260 (0.96)		K276;285;260 (1.25)			
			K505;514;489 (0.70) RPS6KA1;RPS6KA3			

	RPS6KA4 D-2c	RPS6KA4 D-1a	RPS6KA4 C-2a	TEC D-2c	TEC D-1a	TEC C-2a
**f.c. prot.**	-0.70	-	-1.59	-1.07	-0.34	-1.04
modified lysines (f.c.)	K46 (3.04) RPS6KA4;RPS6KA5			K145 (1.38)		K145 (1.91)
			K60 (0.66)	K350 (1.72)		K350 (2.25)
			K134 (1.78)	K394 (1.49)		K394 (1.69)
	K191 (0.97)			K398 (2.10)		K398 (2.20)
	K440;434 (0.91)		K440;434 (1.50)			K504 (1.47)
			K664;658 (0.91)			K526 (3.12)
			K661;655 (0.87)			
	K720;714 (0.65)		K720;714 (0.94)			
	K729;723 (0.55)		K729;723 (1.37)			

Figure S32: *Detailed analysis of lysine ubiquitination. The log_2 fold changes detected in the proteomics experiments (f.c. prot.) and lysine residue ubiquitination mediated by PROTACs **D-2c**, **D-1a** and **C-2a** are shown for selected kinases. Numbers in brackets indicate log_2 fold changes detected in the ubiquitinomics experiments.*

	TNK1			TTK		
	D-2c	D-1a	C-2a	D-2c	D-1a	C-2a
f.c. prot.	-0.56	-	-1.34	-0.67	-	-0.35
modified lysines (f.c.)	K64 (1.53)		K64 (0.63)	K102 (0.35)		
	K73 (1.65)		K73 (0.77)	K546;545 (1.82)		K546;545 (1.18)
	K141 (2.72)		K141 (1.87)	K577;576 (0.72)		
	K148 (2.95)		K148 (1.07)			
	K260 (2.49)		K260 (1.07)			
	K475;470 (1.76)		K475;470 (0.91)			
			K577;572 (0.71)			
	K629;624 (2.54)		K629;624 (1.11)			

	ULK3			WEE1		
	D-2c	D-1a	C-2a	D-2c	D-1a	C-2a
f.c. prot.	-0.30	-	-1.20	-0.39	-	-1.14
modified lysines (f.c.)			K53 (2.05)	K197 (0.68)		K197 (1.07)
	K112 (2.70)		K112 (2.66)	K207 (1.01)		K207 (1.91)
	K293 (0.97)		K293 (2.21)	K219 (0.70)		K219 (1.86)
	K333 (1.24)		K333 (2.38)	K292 (2.03)		K292 (3.71)
	K441 (0.63)		K441 (1.21)			K596 (0.96)

Figure S33: *Detailed analysis of lysine ubiquitination. The log_2 fold changes detected in the proteomics experiments (f.c. prot.) and lysine residue ubiquitination mediated by PROTACs **D-2c**, **D-1a** and **C-2a** are shown for selected kinases. Numbers in brackets indicate the log_2 fold changes detected in the ubiquitinomics experiments.*

4. Hit Validation

4.1. Luciferase Assay

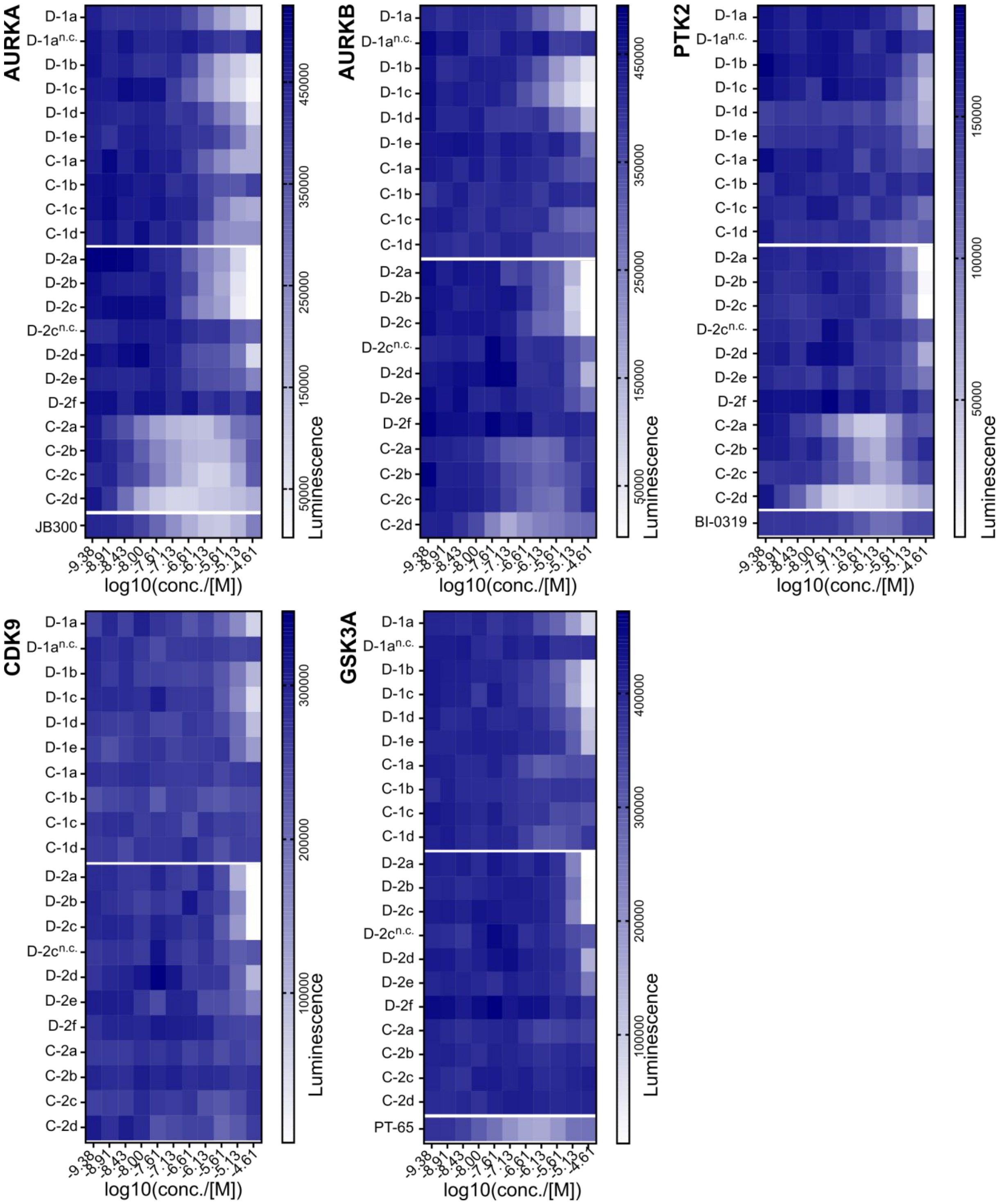

Figure S34: *Concentration-dependent luciferase signal of K562 reporter cell lines treated with the indicated PROTACs. Displayed results are the mean of biological replicates (n=4).*

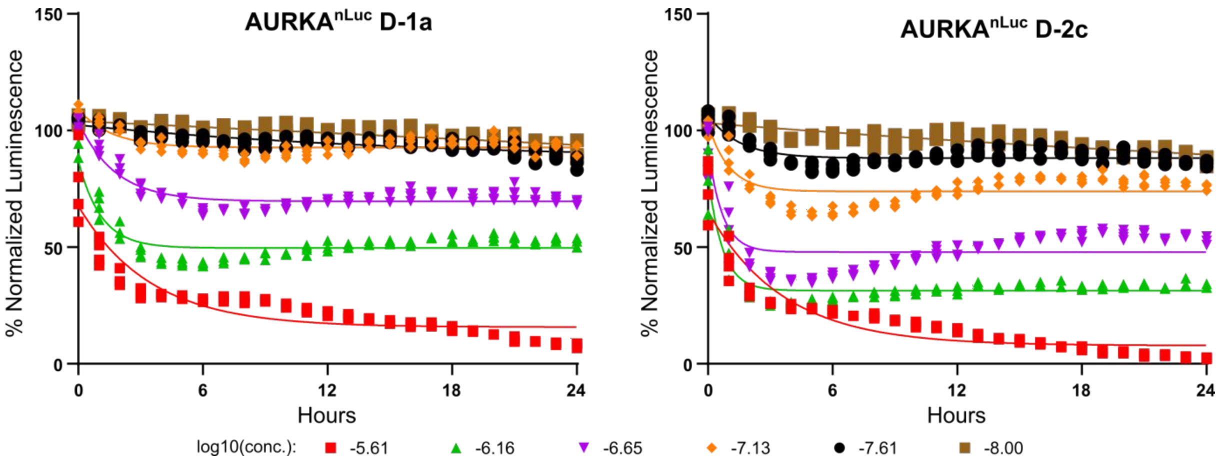

Figure S35: *Time-dependent luciferase signal of AURKA^{nLuc} K562 reporter cell lines treated with the indicated PROTACs concentrations. Displayed results are the mean of technical replicates (n=4).*

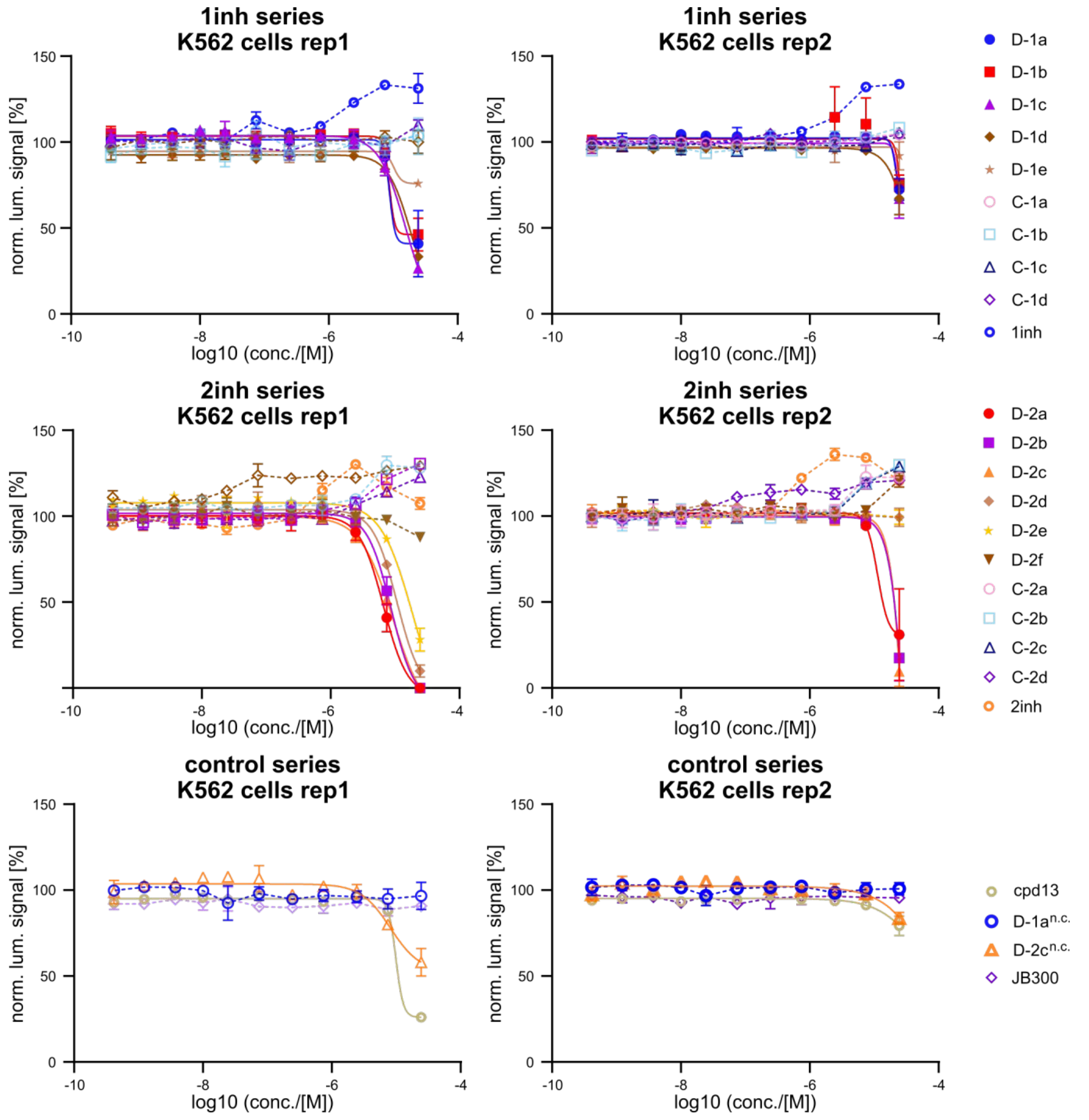

Figure S36: *Cell viability data (CellTiter Glo) of all promiscuous kinase PROTACs, their respective kinase parent ligands and the control compounds. K562 cells, expressing nLuc-tagged AURKA, were treated in a 11-point dose series of the indicated compounds for 6 h. Each curve represents the mean of technical replicates (n=2). Error bars indicate the standard deviation. Dotted lines connect datapoints, for which no sigmoidal curve could be fitted.*

4.2. Western Blotting

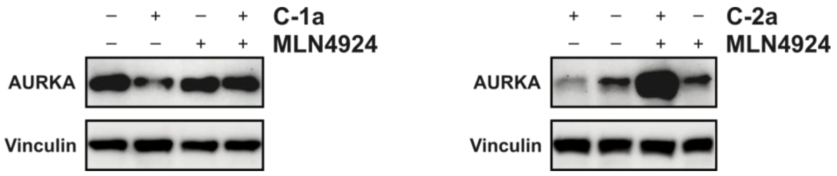

Figure S37: *Western Blotting control experiments for selected CRBN-based PROTACs. MDA-MB-231 cells were treated with **C-1a** (0.5 µM), **C-2a** (0.1 µM) and/or **MLN4924** (1.0 µM) for 6 h.*

4.3. HiBiT Split Luciferase Assay

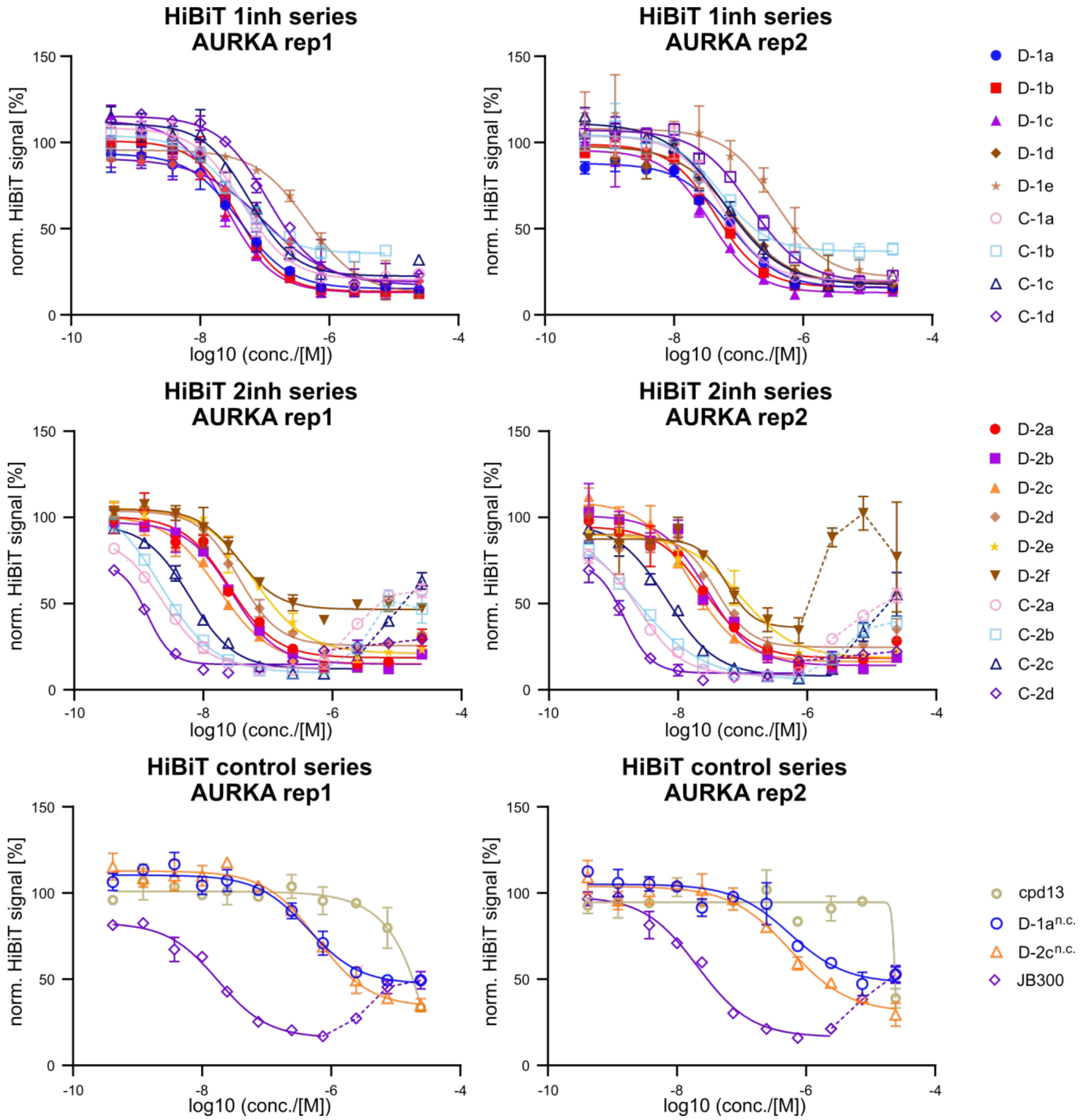

Figure S38: *AURKA protein levels based on luciferase measurements. MV4-11 cells, expressing tagged AURKA^{HiBiT} protein were treated with different concentrations of the specified compounds for 6 hours. Following cell lysis, the resulting lysates were complemented with the largeBiT fragment, and luciferase activity was measured.*

Table S6: *Degradation potency (DC_{50} and D_{max}) of all PROTACs tested against $AURKA^{HiBiT}$. Values were calculated from biological replicates (n=4). Errors represent the standard deviation. [a]Values were calculated excluding datapoints at higher concentrations to correct for the hook effect.*

cmpd ID	DC_{50} ±sd [nM] $AURKA^{HiBiT}$	D_{max} ±sd [%] $AURKA^{HiBiT}$
D-1a	57±23	84±1
D-1a n.c.	501±81	
D-1b	41±5	85±2
D-1c	30±9	87±1
D-1d	92±12	83±7
D-1e	415±66	82±2
C-1a	50±7	84±3
C-1b	44±13	85±2
C-1c	59±2	64±1
C-1d	132±32	82±3
D-2a	26±1	87±3
D-2b	28±1	88±1
D-2c	17±1	88±5
D-2c n.c.	581±36	
D-2d	37±3	80±2
D-2e	77±22	85±7
D-2f	46±16[a]	63±2
C-2a	2±1[a]	92±2
C-2b	2±1[a]	93±2
C-2c	6±1[a]	93±3
C-2d	1±1[a]	91±3
JB300	19±3[a]	86±4

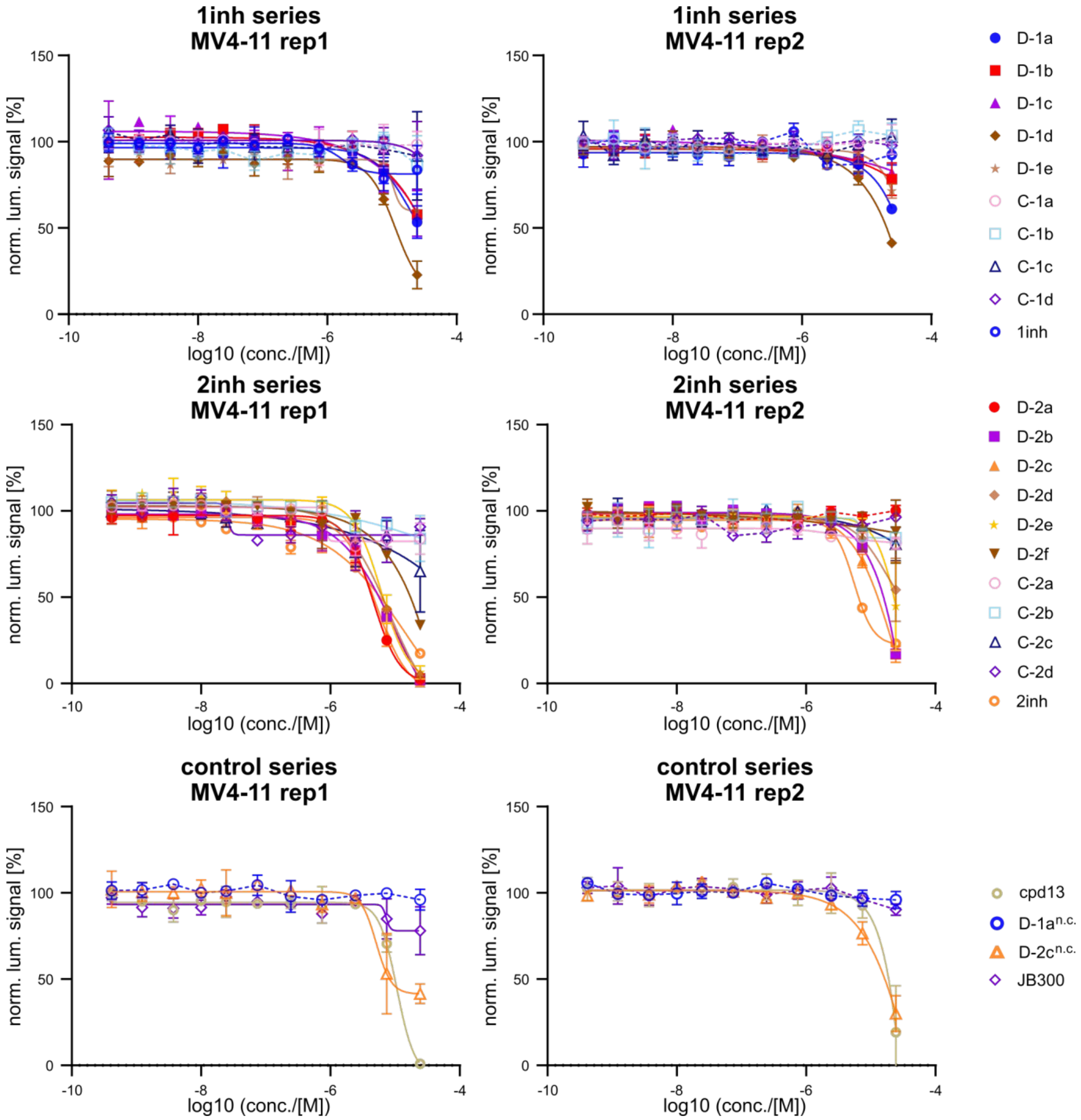

Figure S39: *Cell viability data (CellTiter Glo) of all promiscuous kinase PROTACs, their respective kinase parent ligands and the control compounds. MV4-11 cells, expressing tagged AURKA^{HiBiT}, were treated in a 11-point dose series of the indicated compounds for 6 h. Each curve represents the mean of technical replicates (n=2). Error bars indicate the standard deviation. Dotted lines connect datapoints, for which no sigmoidal curve could be fitted.*

5. Physicochemical Properties of Utilized E3 Ligase Ligands

*Table S7: Comparison of physicochemical properties of the CRBN ligand thalidomide and the DCAF1 ligand cpd13. Criteria and upper limits for oral administration of PROTACs according to K.R. Hornberger and E.M.V. Araujo, Physicochemical Property Determinants of Oral Absorption for PROTAC Degraders, Journal of medicinal chemistry, **2023**, 66, 82181-8287. For the calculation of tPSA values, SWISSADME was used: SwissADME: a free web tool to evaluate pharmacokinetics, drug-likeness and medicinal chemistry friendliness of small molecules. A. Daina, O. Michielin, V. Zoete, Sci. Rep. **2017**, 7, 42717.*

Property	PROTAC Limit	CRBN Ligand	DCAF1 Ligand	Average Linker	POI Binder Budget		
					CRBN	DCAF1	Δ Budget
MW	950	259	507	218	473	225	-248
TPSA (Å2)	200	84	87	14	102	99	-3
RB	14	1	6	3	10	5	-5
HBA	15	4	6	3	7	6	-1
HBD (unsat.)	2	1	2	0	1	0	-1

6. Chemical Synthesis Schemes

6.1. DCAF1 Parent Ligand and tracer

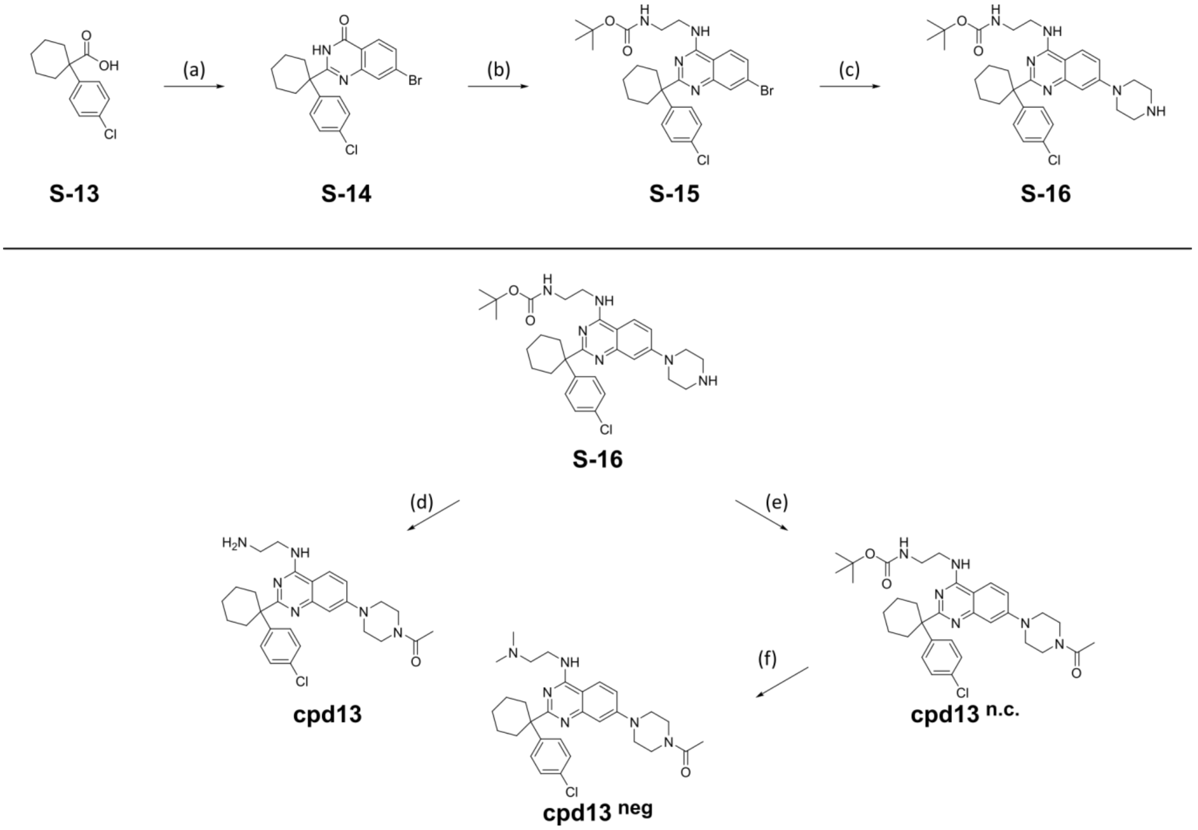

Figure S40: (a) 1-chloro-N,N,2-trimethyl-1-propenylamine, THF, 1 h; 2. 2-amino-4-bromo-benzamide, TEA, THF, 5 h; 3. NaOET, EtOH, 50 °C, 16 h; (b) 1. PyBOP, DIPEA, DMF, 3 h; 2. N-Boc-ethylenediamine, 3 h; (c) piperazine, sodium tert-butoxide, RuPhos Pd G4, THF, 70 °C, 4 h; (d) 1. Acetic anhydride, TEA, 4-DMAP, DCM, 0 °C - r.t., 20 min; 2. 25vol% TFA/DCM, r.t., 30 min; (e) Acetic anhydride, TEA, 4-DMAP, DCM, 0 °C, then r.t., 1 h, (f)1. 33vol% TFA/DCM, r.t., 1 h; 2. Formaldehyde, AcOH, MeOH, r.t. 2 h; 3. Sodium borohydride, r.t., 2 h.

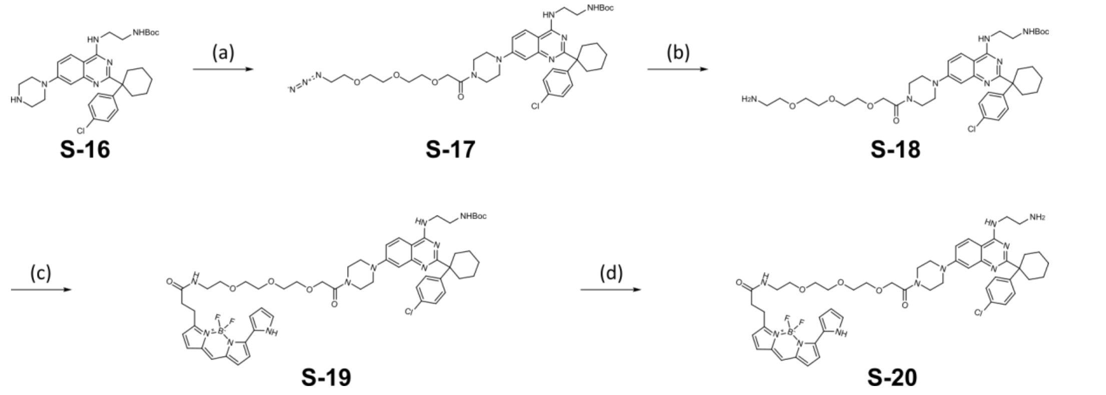

Figure S41: (a) 11-Azido-3,6,9-trioxaundecanoic acid, pyridine, 2,4,6-tripropyl-1,3,5,2,4,6-trioxatriphosphinane 2,4,6-trioxide, DMF, r.t. 1.5 h; (b) nickel(II) chloride, sodium borohydride, MeoH, r.t. 5 h; (c) 12-(2-carboxyethyl)-2,2-difluoro-4-(1H-pyrrol-2-yl)-1lambda5,3-diaza-2-boratricyclo[7.3.0.0^{3,7}]dodeca-1(12),4,6,8,10-pentaen-1-ylium-2-uide (preactivated: HATU, N-methylmorpholine, DMF, r.t., 15 min), N-methylmorpholine, DMF, r.t., 1 h; (d) formic acid, 40 °C, 1 h.

6.2. Promiscuous Kinase PROTACs

6.2.1. DCAF1-recruiting Promiscuous Kinase PROTACs based on Kinase Parent Inhibitor 1inh

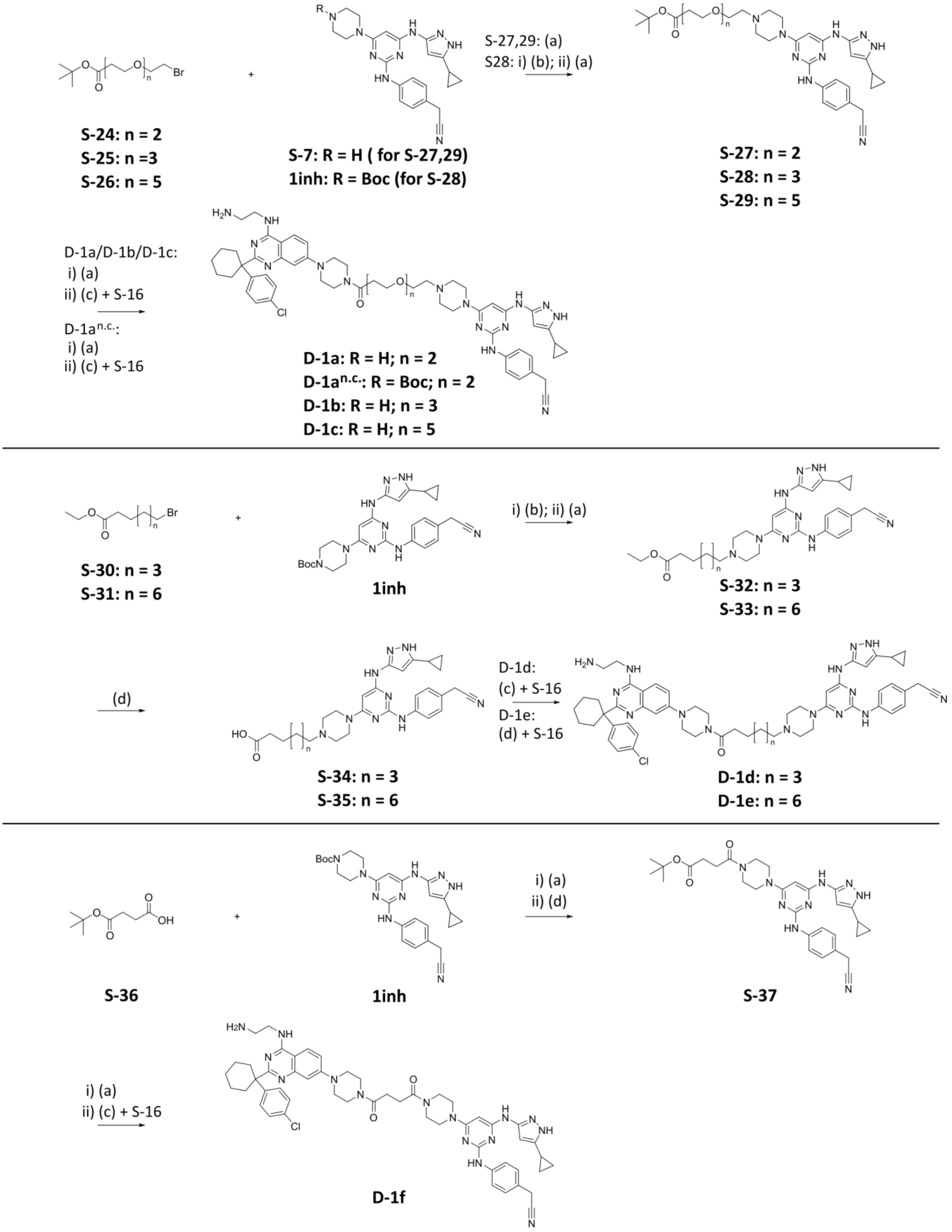

Figure S42: (a) K_2CO_3, MeCN, 80 – 85 °C, overnight; (b) 25vol% TFA/DCM r.t., 30 min – 3 h; (c) PyBOP or PyAOP, DIPEA, DMF, r.t. 1 h – overnight, (d) LiOH·H_2O, MeOH/H_2O (4:1), 40 °C, overnight.

6.2.2. CRBN-recruiting Promiscuous Kinase PROTACs based on Kinase Parent Inhibitor 1inh

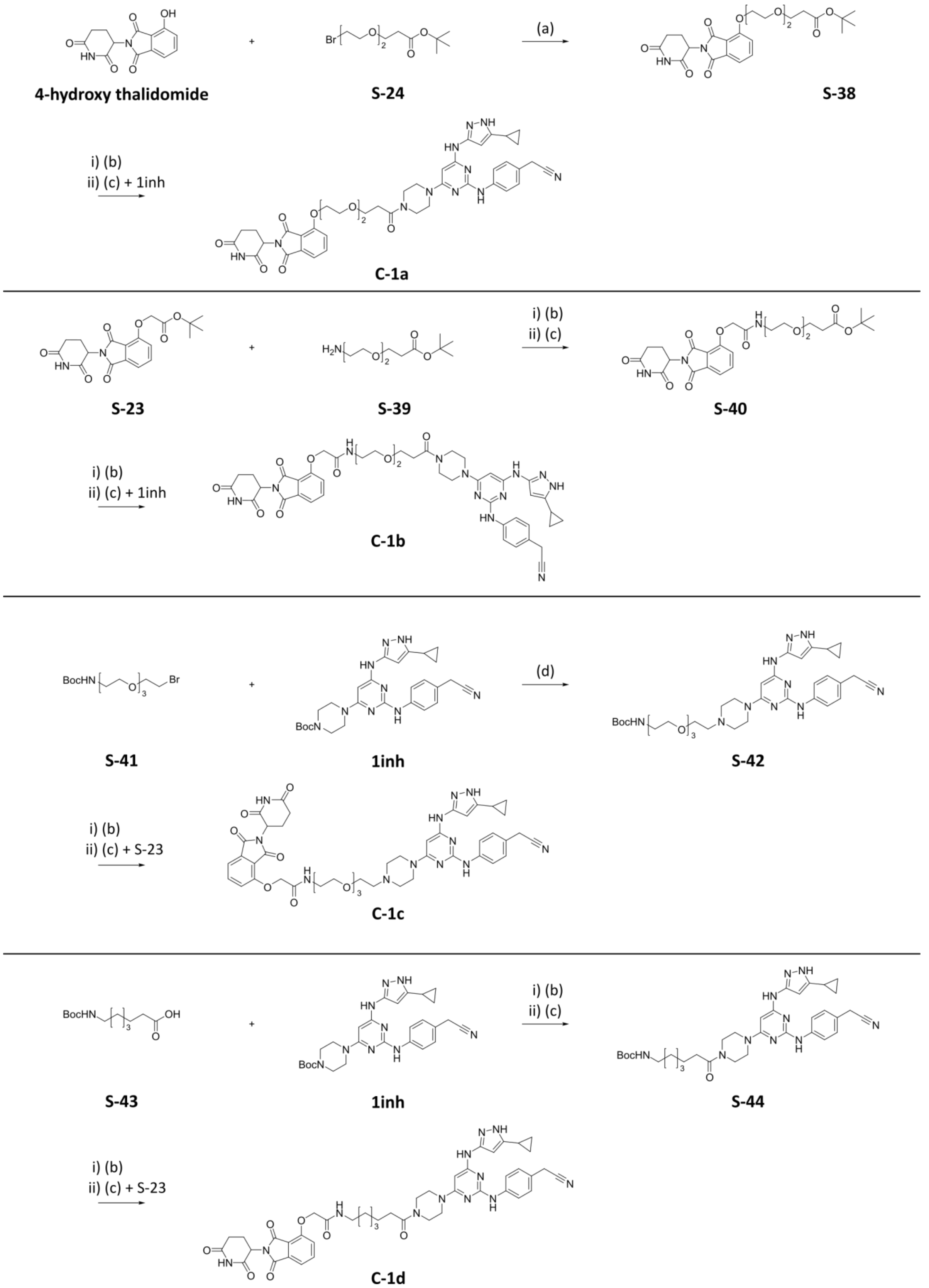

Figure S43: (a) $NaHCO_3$, KI, DMF, 80 °C, overnight; (b) 25vol% TFA/DCM r.t., 30 – 45 min; (c) HATU, DIPEA, DMF, r.t. 1 h– overnight; (d) K_2CO_3, MeCN, 75 °C, overnight.

6.2.3. DCAF1-recruiting Promiscuous Kinase PROTACs based on Kinase Parent Inhibitor 2inh

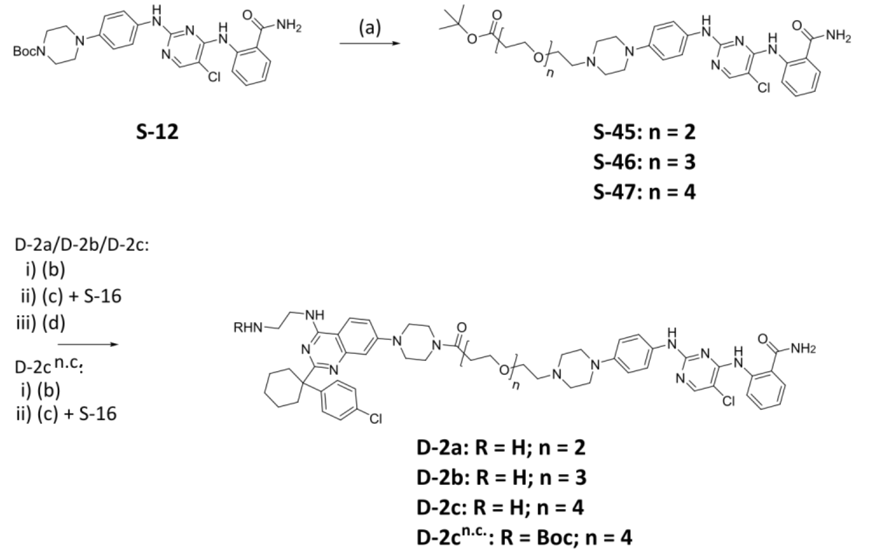

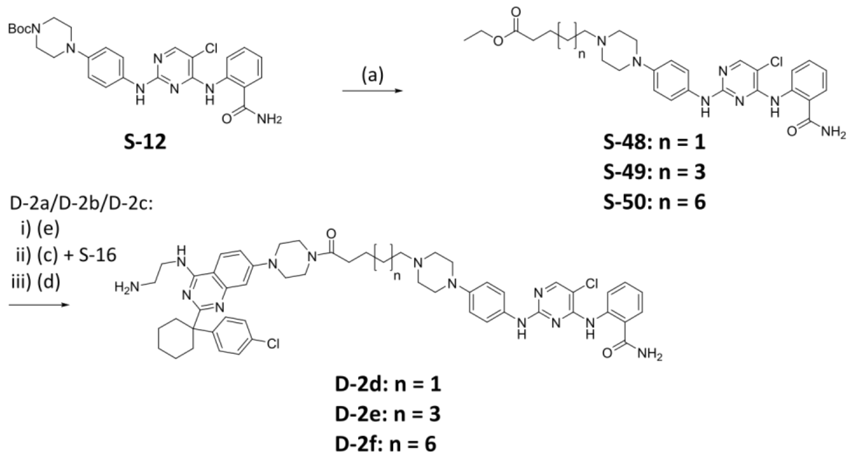

Figure S44: (a) 1. 50vol% TFA/DCM, r.t., 3-6 h or 1,4-dioxane/HCl (4 M), r.t., 5 h; 2. Appropriate linker, K_2CO_3 or Cs_2CO_3, DMF, 70-80 °C, overnight; (b) 50vol% TFA/DCM, r.t., 2-3 h; (c) DIPEA, PyBOP, DMF/THF (1:1), 50 °C, overnight; (d) 50vol% TFA/DCM, r.t., 2 h; (e) LiOH monohydrate, THF/MeOH/H_2O (1:1:1), 50 °C, 4-6 h.

6.2.4. CRBN-recruiting Promiscuous Kinase PROTACs based on Kinase Parent Inhibitor 2inh

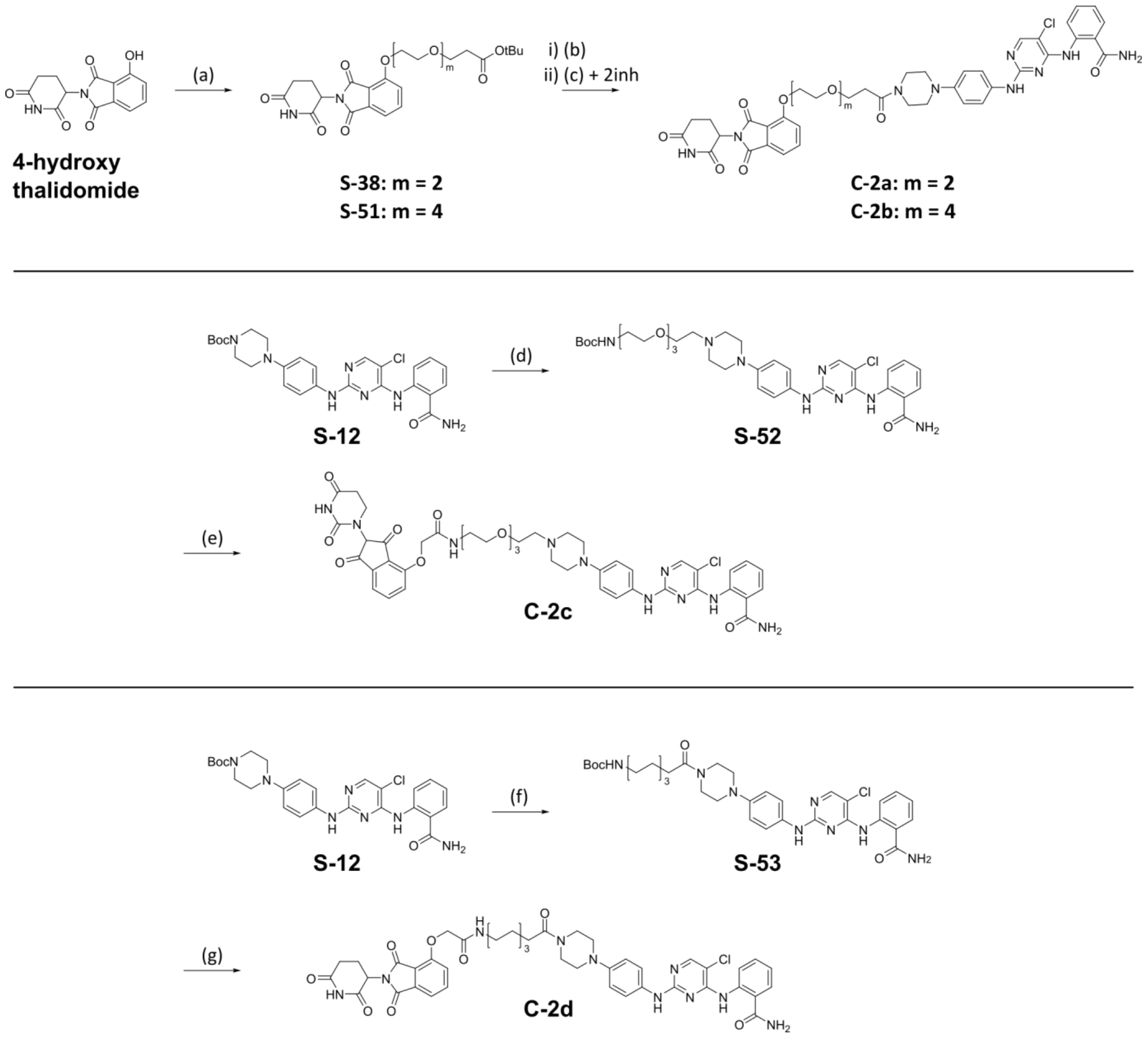

Figure S45: (a) $NaHCO_3$, appropriate linker, KI, DMF, r.t., overnight; (b) TFA/DCM (1:1), r.t., 4-5 h; (c) DIPEA, HATU, DMF, r.t., overnight; (d) 1. 1,4-dioxane/HCl (2 M), r.t., 2 h; 2. K_2CO_3, (2-(2-(2-(2-bromoethoxy)ethoxy)ethoxy)ethyl)carbamate, DMF, r.t., 5 h, then 50 °C, 18 h; (e) 1. 1,4-dioxane/HCl (4 M), r.t., 2 h; 2. rac-2-((2-(2,6-dioxopiperidin-3-yl)-1,3-dioxoisoindolin-4-yl)oxy)acetic acid (prepared from **S-23** and TFA/DCM, r.t., 2 h), HOBt, EDC hydrochloride, 4-methylmorpholine, DMF, r.t., 12 h; (f) 1. 50vol% TFA/DCM, r.t., 2 h; 2. tert-butyl 7-bromoheptyl)carbamate, Cs_2CO_3, DMF, 70 °C, overnight; (g) 1. 50vol% TFA/DCM, r.t., 3.5 h; 2. rac-2-((2-(2,6-dioxopiperidin-3-yl)-1,3-dioxoisoindolin-4-yl)oxy)acetic acid (prepared from **S-23** and TFA/DCM, r.t., 2 h), DIPEA, HATU, DMF, r.t., overnight.

7. Compound Characterization Data

7.1. DCAF1 Parent Ligand and Corresponding Negative Controls

7-Bromo-2-[1- (4-chlorophenyl)cyclohexyl]-3,4-dihydroquinazolin-4-one (S-14)

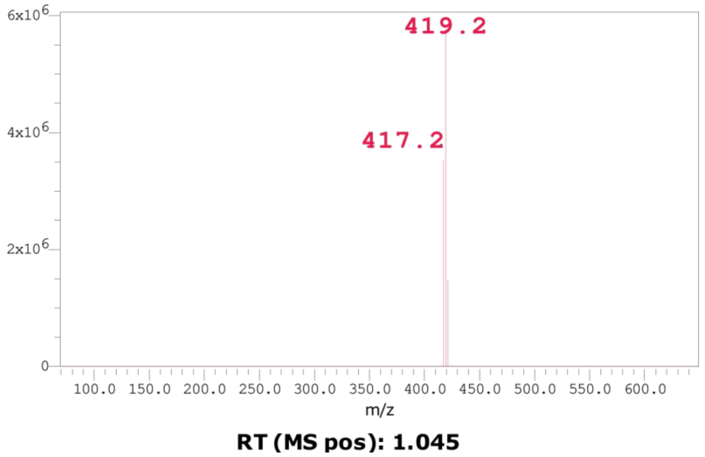

Figure 46: ESI-MS spectrum of compound **S-14** with $[M+3H]^{3+}_{calc.}$ = 419.1 m/z.

Tert-butyl *N*-[2-(7- bromo-2-[1-(4-chlorophenyl)cyclohexyl]quinazolin-4-ylamino)ethyl]carbamate (S-15)

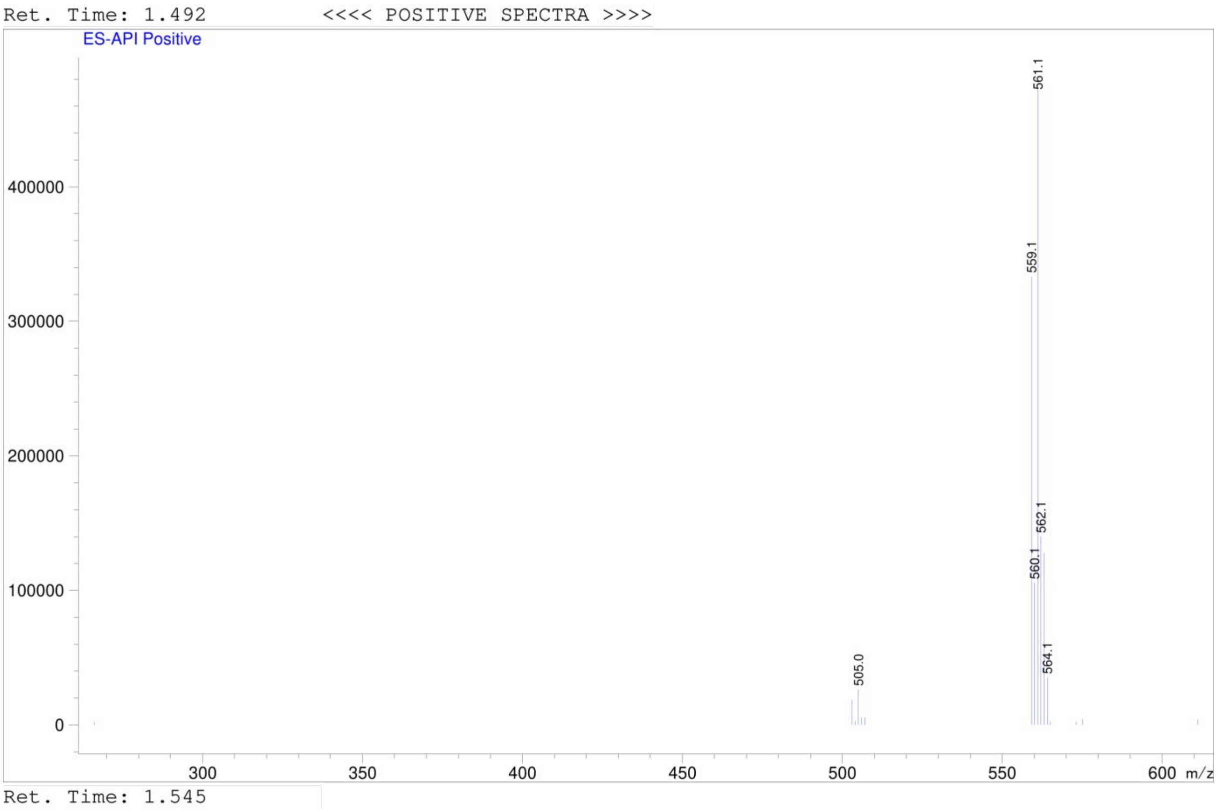

Figure 47: ESI-MS spectrum of compound **S-15** with $[M+3H]^{3+}_{calc.}$ = 561.2 m/z.

Tert-butyl N-[2-({2-[1-(4-chlorophenyl)cyclohexyl]-7-(piperazin-1-yl)quinazolin-4-yl}amino)ethyl]carbamate (S-16)

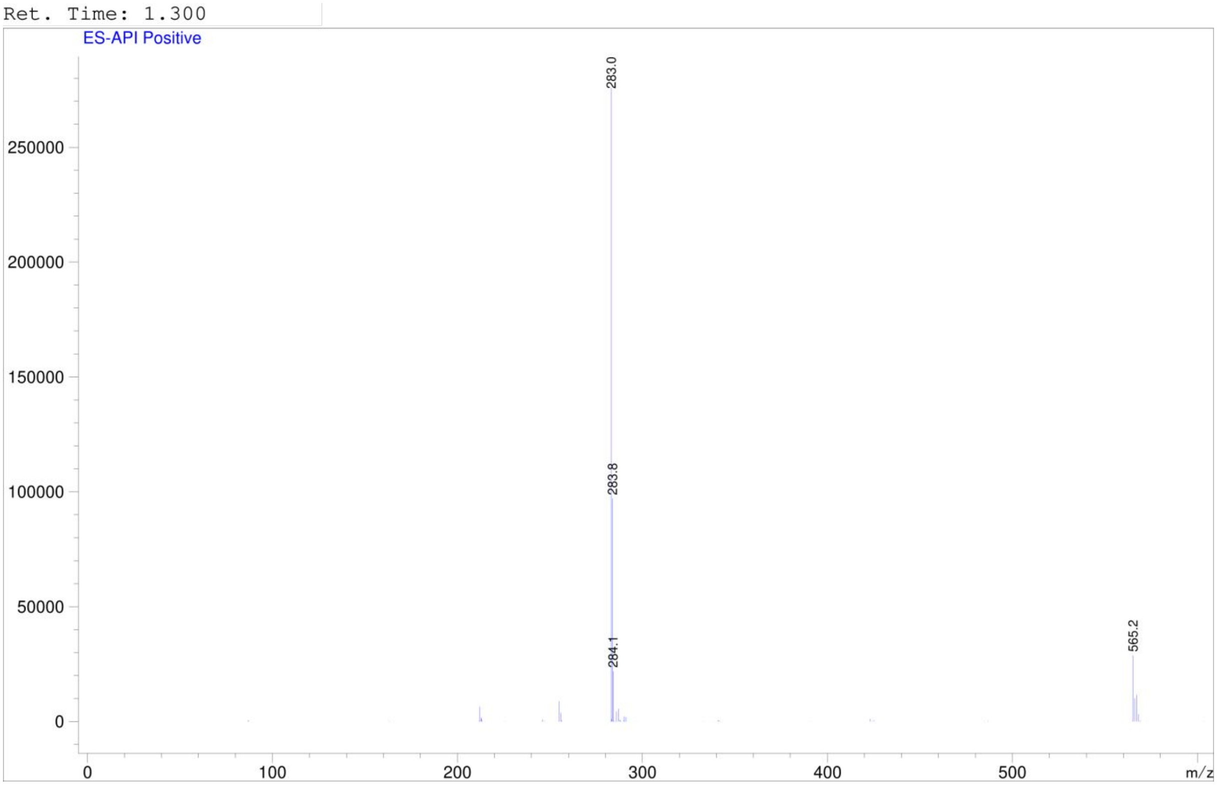

Figure 48: ESI-MS spectrum of compound **S-16** with $[M+H]^+_{calc.}$ = 565.3 m/z.

1-(4-(4-((2-Aminoethyl)amino)-2-(1-(4-chlorophenyl)cyclohexyl)quinazolin-7-yl)piperazin-1-yl)ethan-1-one (cpd13)

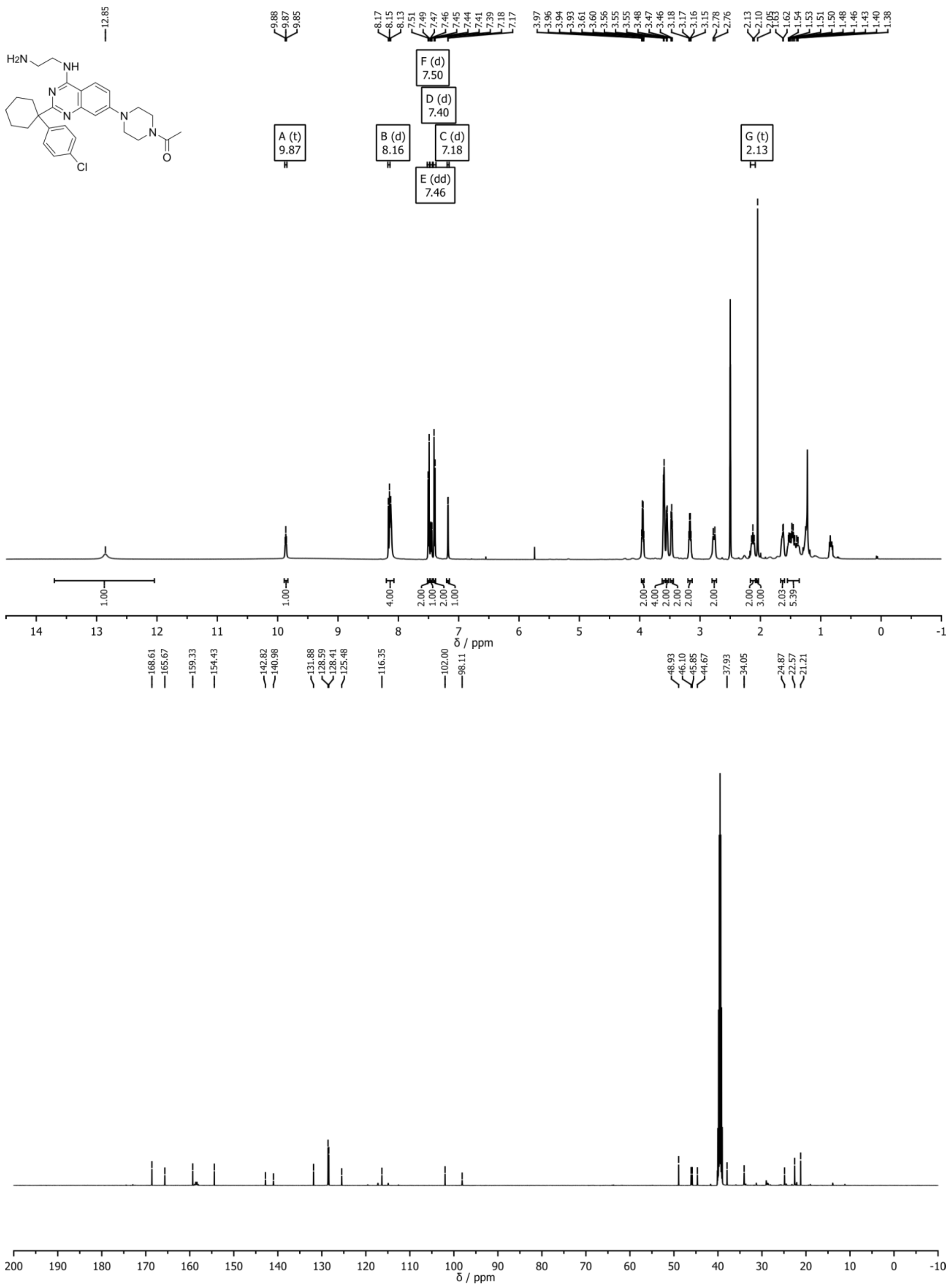

Figure S49: ^{1}H- (top) and ^{13}C-NMR (bottom) spectra (500 MHz and 126 MHz, 298 K, DMSO-d_6) and chemical structure of compound **cpd13**.

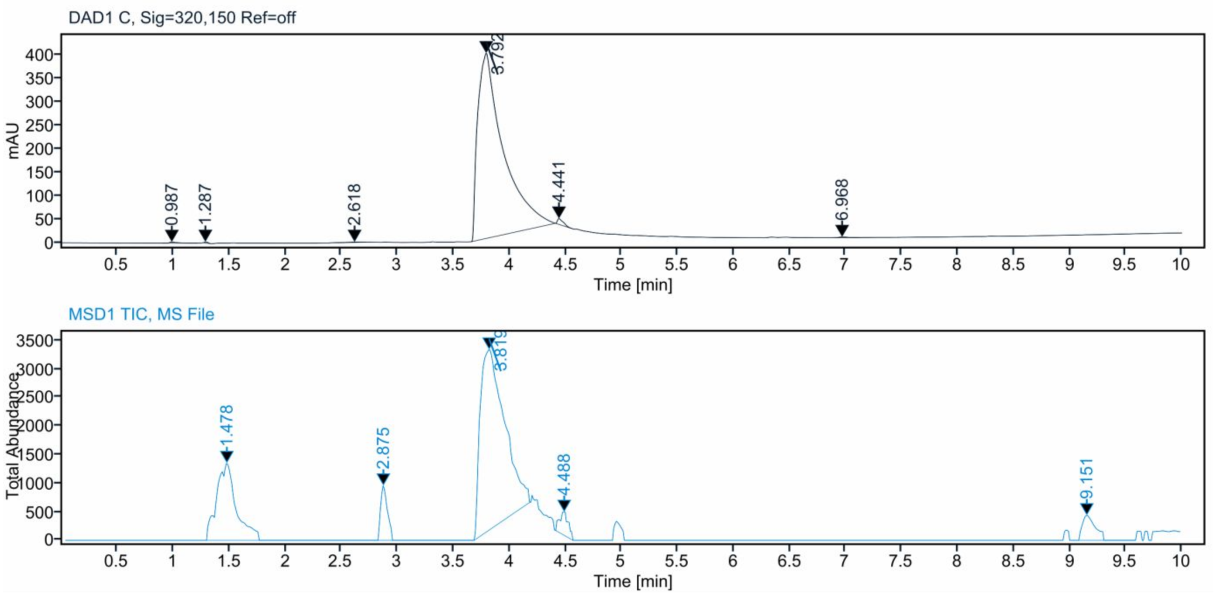

Sample Purity

Signal Description DAD1 C, Sig=320,150 Ref=off

Sample Name	Name	RT	Width	Area	Area%	Height
NM623_pur		0.987	0.056	10.3306	0.16	2.3285
NM623_pur		1.287	0.032	5.6097	0.09	2.5421
NM623_pur		2.618	0.093	8.6243	0.14	1.1494
NM623_pur		3.792	0.233	6271.8921	98.75	394.8911
NM623_pur		4.441	0.049	46.8676	0.74	12.4865
NM623_pur		6.968	0.083	8.0970	0.13	1.5726

Max Area% 98.748

UV Signal Purity>95% Pass

Figure S50: LC/MS spectra of purified compound **cpd13** at 320 nm wavelength.

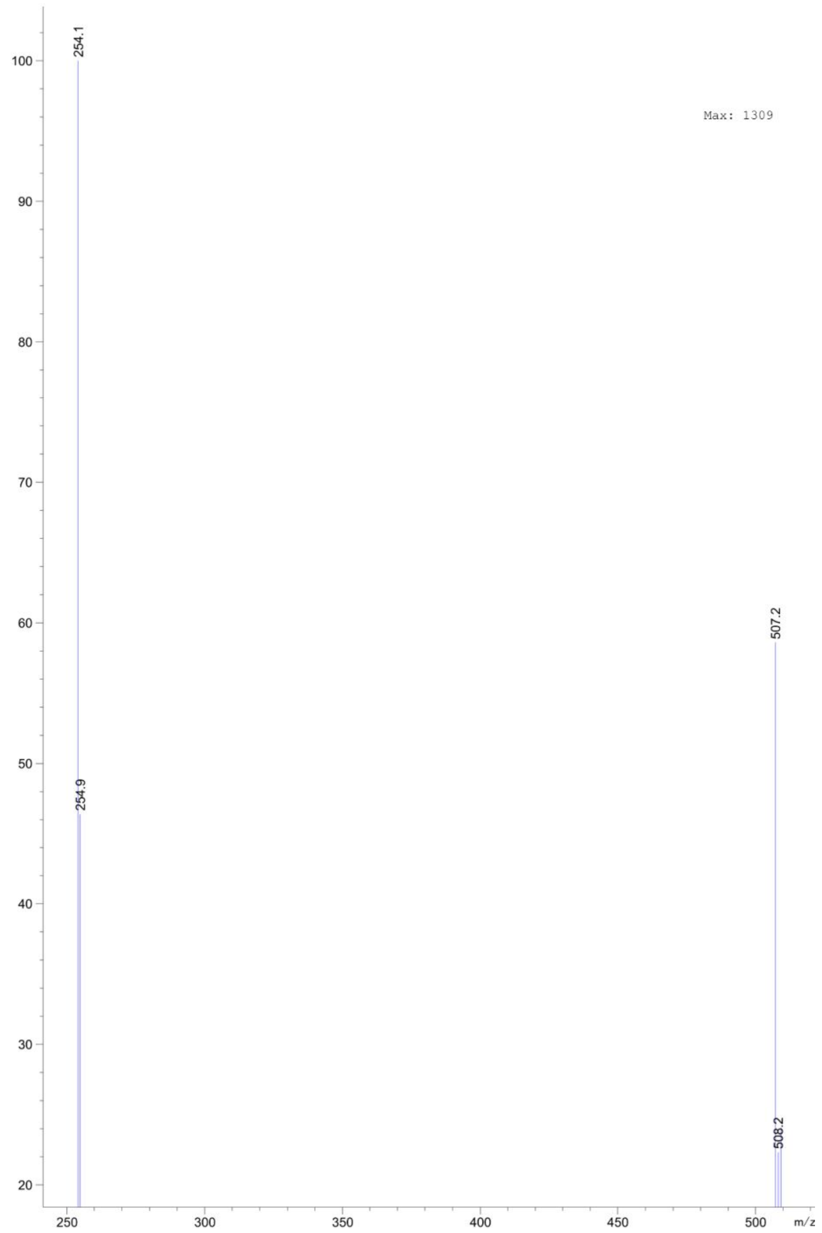

Figure S51: ESI-MS spectrum of **cpd13** with $[M+H]^+_{calc.}$ = 507.26 m/z

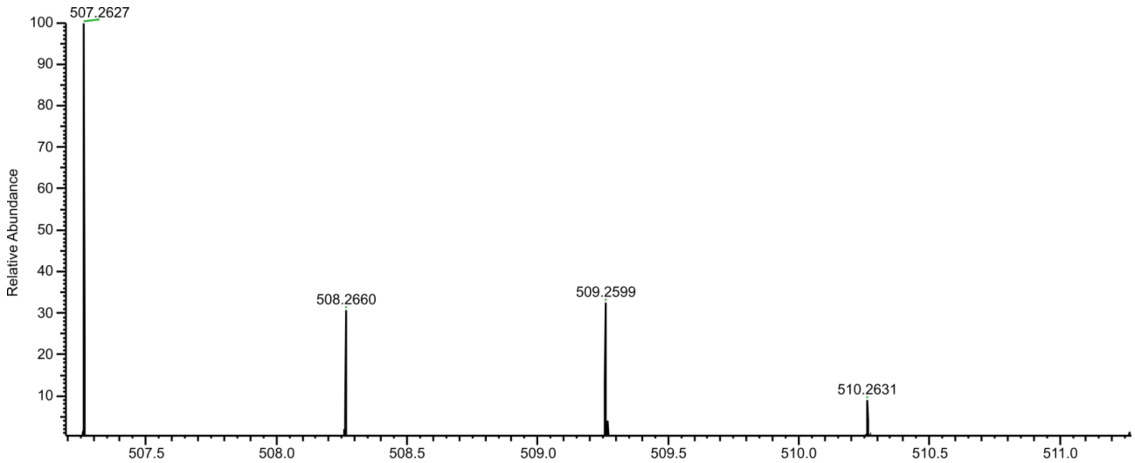

Figure S52: High-resolution mass spectrum of compound **cpd13** with $[M+H]^+_{calc.}$ = 507.2634 m/z.

***Tert*-butyl (2-((7-(4-acetylpiperazin-1-yl)-2-(1-(4-chlorophenyl)cyclohexyl)quinazolin-4-yl)amino)ethyl)carbamate (cpd13[n.c.])**

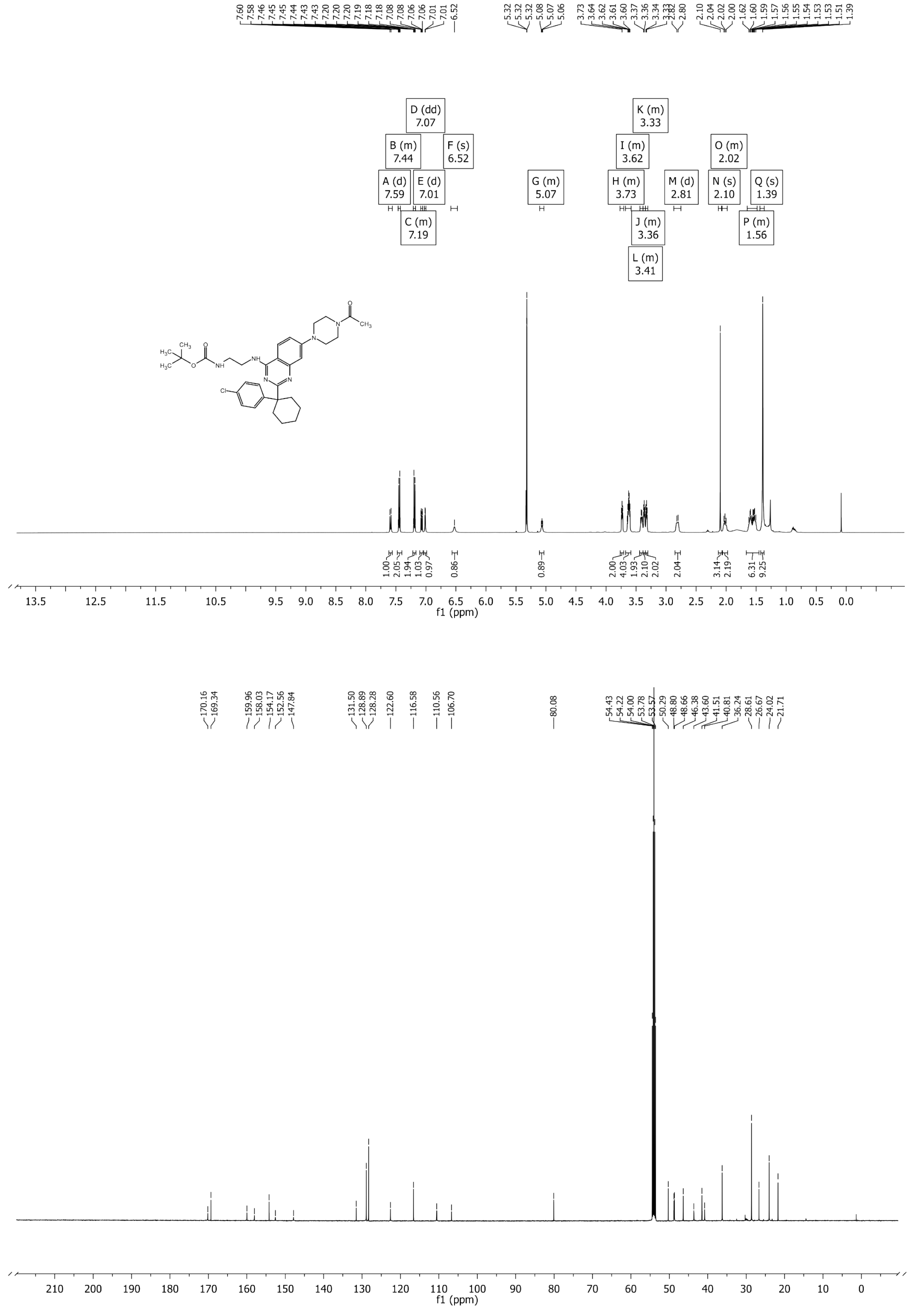

Figure S53: ^{1}H- (top) and ^{13}C-NMR (bottom) spectra (500 MHz and 126 MHz, 298 K, DCM-d_2) and chemical structure of compound **cpd13[n.c.]**.

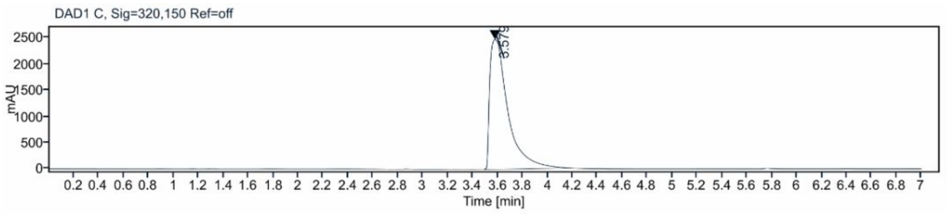

Signal Description	DAD1 C, Sig=320,150 Ref=off					
**Sample Name**	**Name**	**RT**	**Width**	**Area**	**Area%**	**Height**
jw-609		3.579	0.142	24983.58 40	100.00	2476.6445

Max Area%	100.000
UV Signal Purity>95%	Pass

Figure S54: LC/MS spectra of purified compound **cpd13$^{n.c.}$** at 320 nm wavelength.

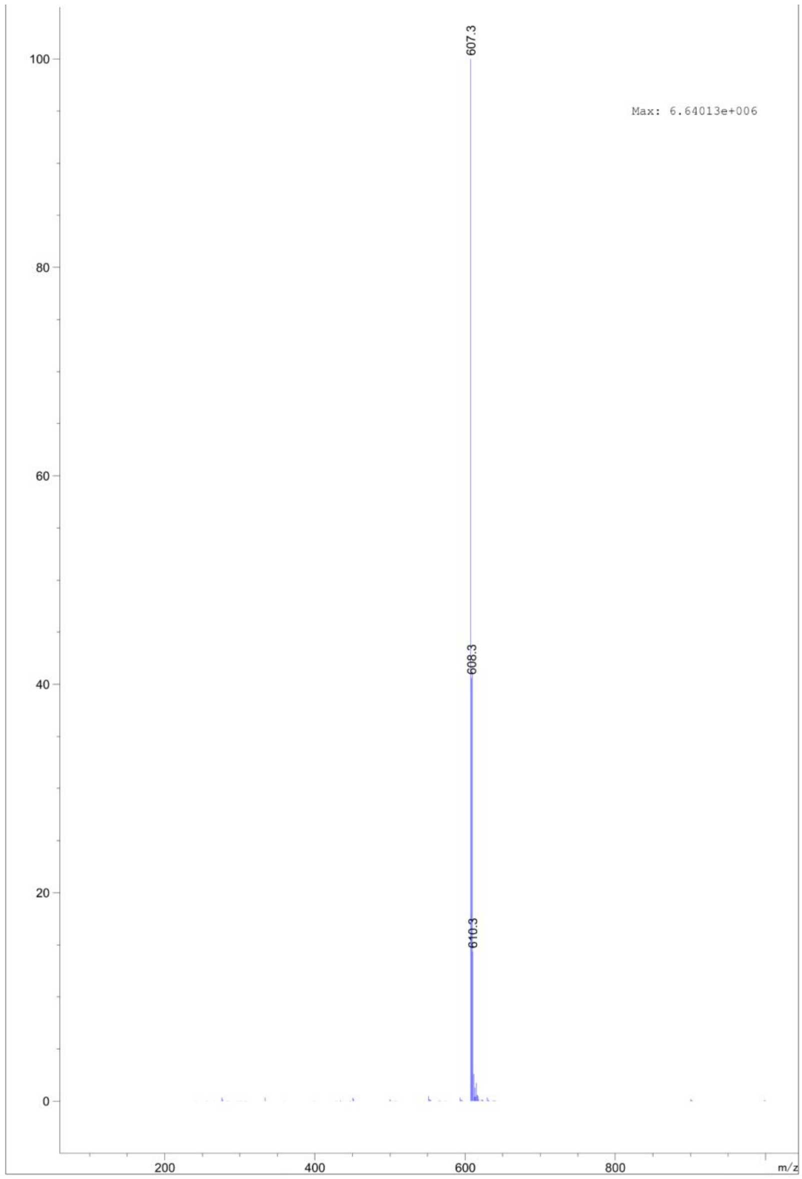

Figure S55: ESI-MS spectrum of **cpd13$^{n.c.}$** with $[M+H]^+_{calc.}$ = 607.3 m/z

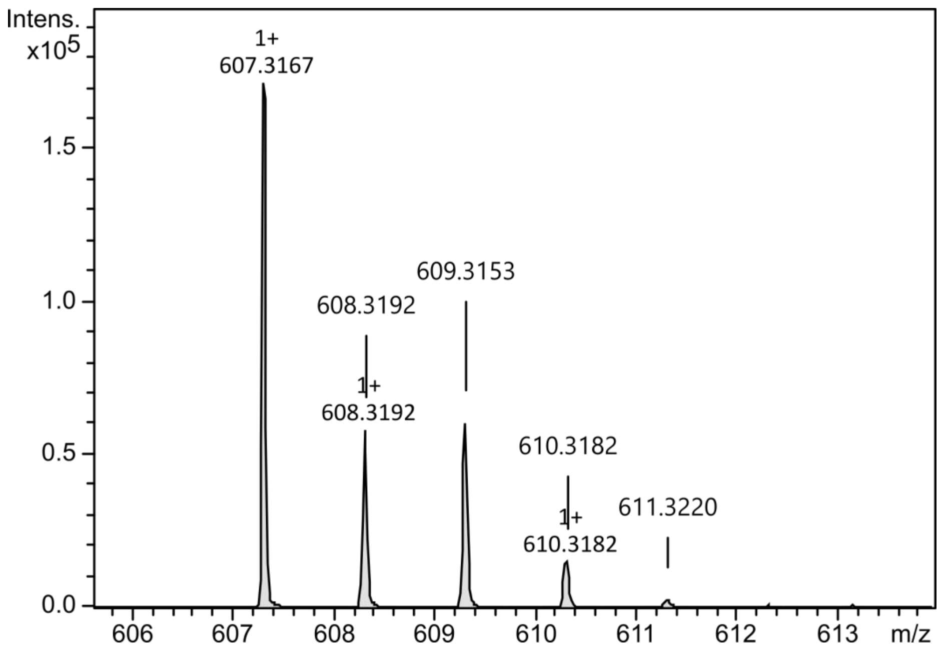

Figure S56: High-resolution mass spectrum of compound **cpd13**$^{n.c.}$ with $[M+H]^{+}_{calc.}$ = 607.3157 m/z.

1-(4-(2-(1-(4-Chlorophenyl)cyclohexyl)-4-((2-(dimethylamino)ethyl)amino)quinazolin-7-yl)piperazin-1-yl)ethan-1-one (cpd13-N)

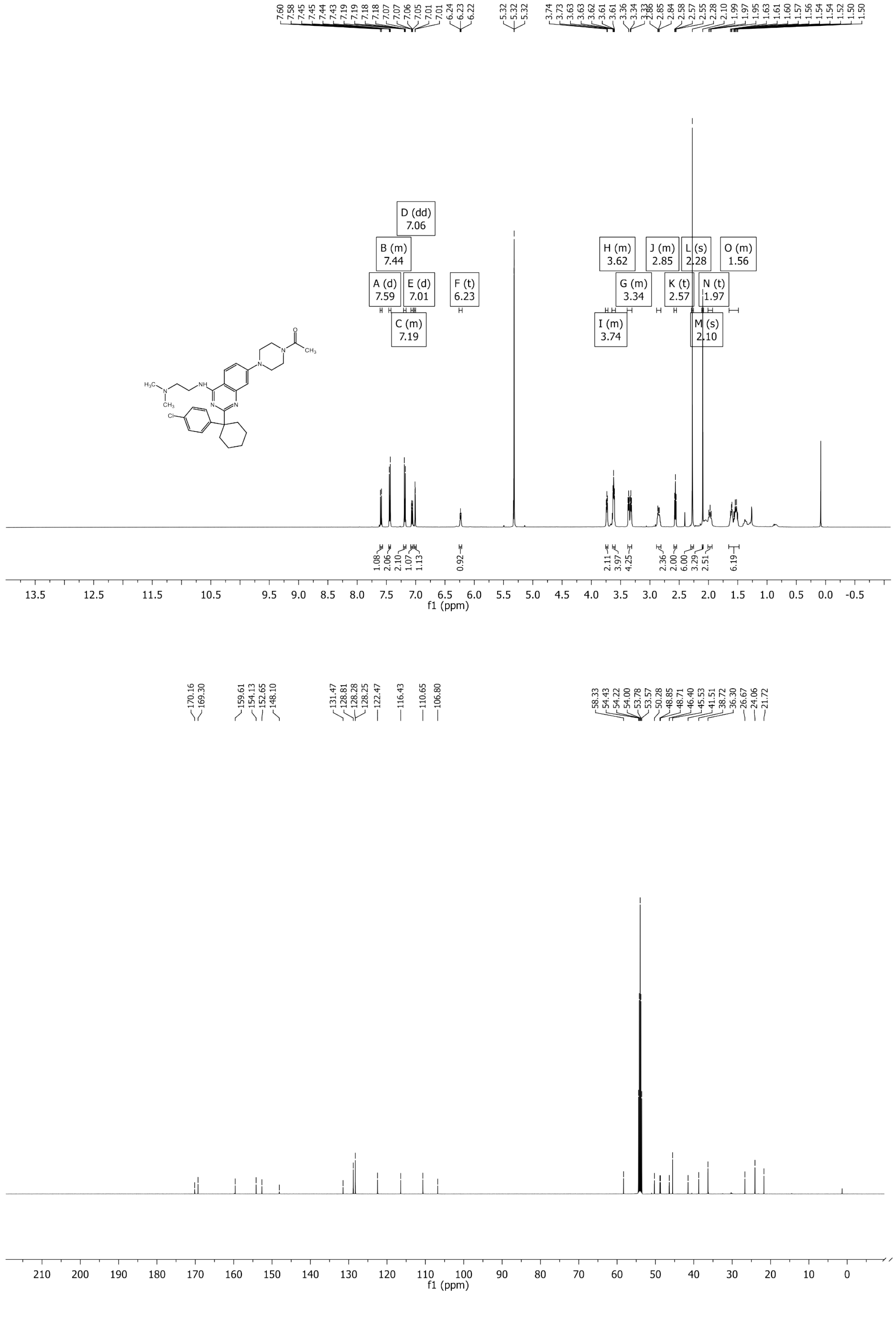

Figure S57: ^{1}H- (top) and ^{13}C-NMR (bottom) spectra (500 MHz and 126 MHz, 298 K, DCM-d_2) and chemical structure of compound **cpd13-N**.

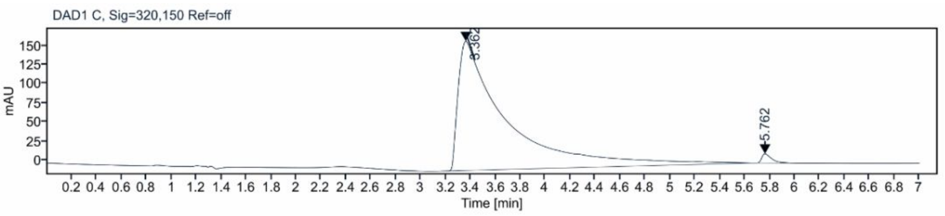

Signal Description	DAD1 C, Sig=320,150 Ref=off					
**Sample Name**	**Name**	**RT**	**Width**	**Area**	**Area%**	**Height**
jw-610 34_x		3.362	0.307	4548.8813	98.58	168.8454
jw-610 34_x		5.762	0.078	65.6750	1.42	11.9589

Max Area% 98.577

UV Signal Purity>95% Pass

Figure S58: LC/MS spectra of purified compound **cpd13-N** at 320 nm wavelength.

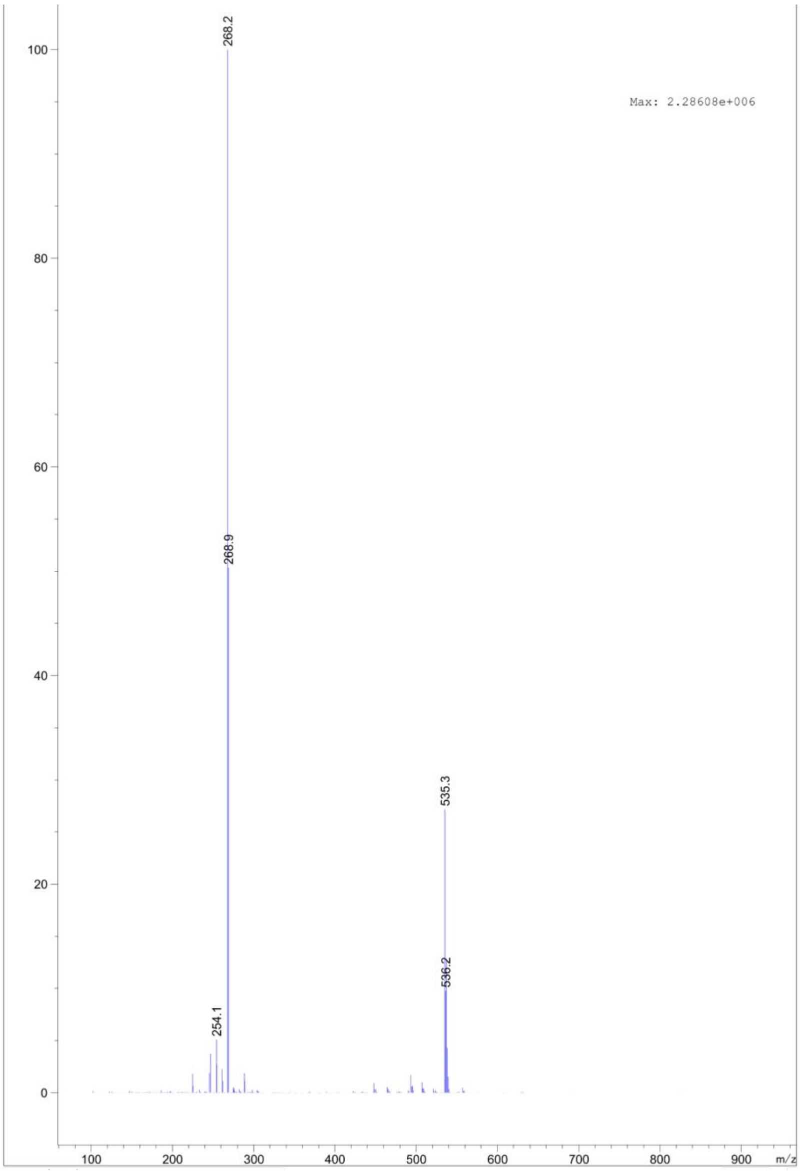

Figure S59: ESI-MS spectrum of **cpd13-N** with $[M+H]^{+}_{calc.}$ = 535.3 m/z

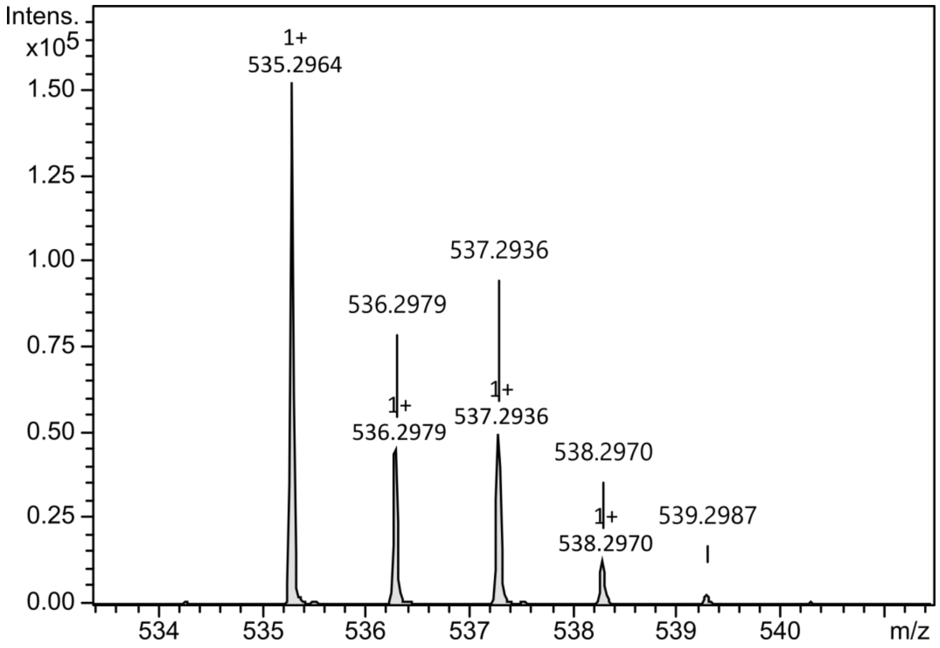

Figure S60: High-resolution mass spectrum of compound **cpd13-N** with $[M+H]^+_{calc.}$ = 535.2947 m/z.

Tert-butyl (2-((7-(4-(2-(2-(2-(2-azidoethoxy)ethoxy)ethoxy)acetyl)piperazin-1-yl)-2-(1-(4-chlorophenyl)cyclohexyl)quinazolin-4-yl)amino)ethyl)carbamate (S-17)

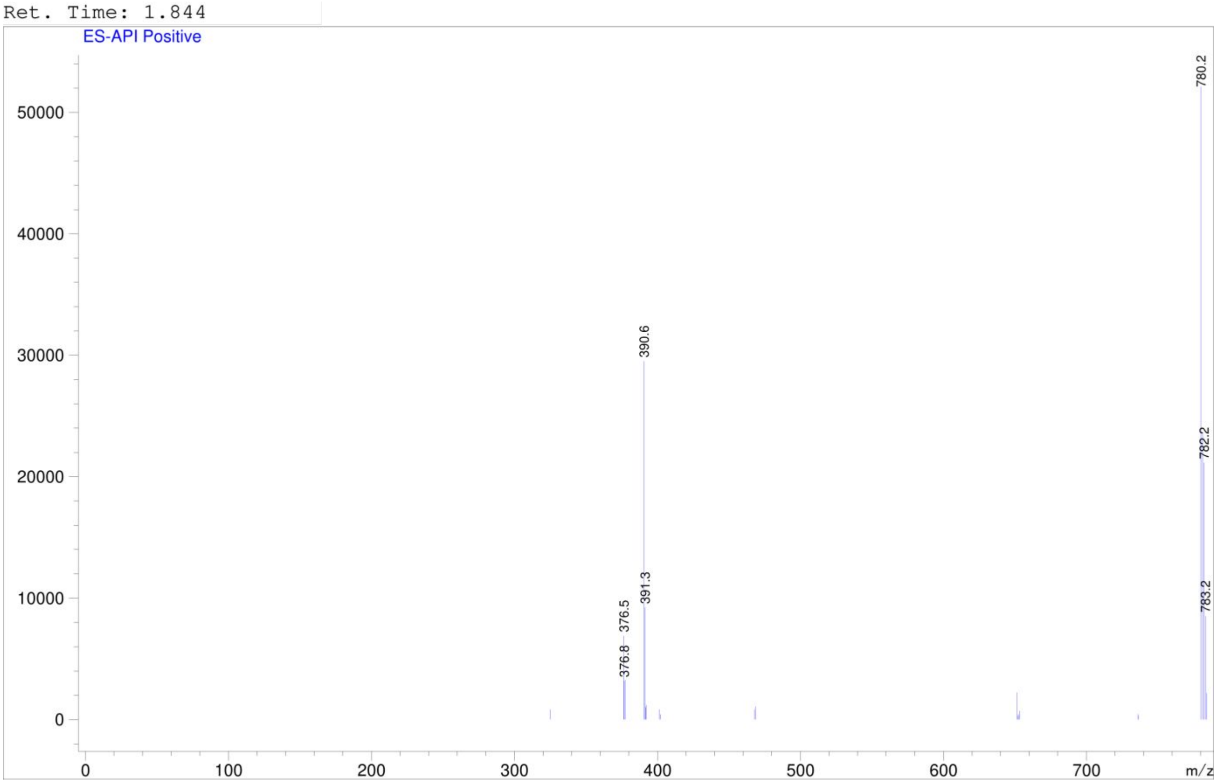

Figure S61: ESI-MS spectrum of compound **S-17** with $[M+H]^{+}_{calc.}$ = 780.4 m/z.

Tert-butyl N-[2-({7-[4-(2-{2-[2-(2-aminoethoxy)ethoxy]ethoxy}acetyl)piperazin-1-yl]-2-[1-(4-chlorophenyl)cyclohexyl]quinazolin-4-yl}amino)ethyl]carbamate (S-18)

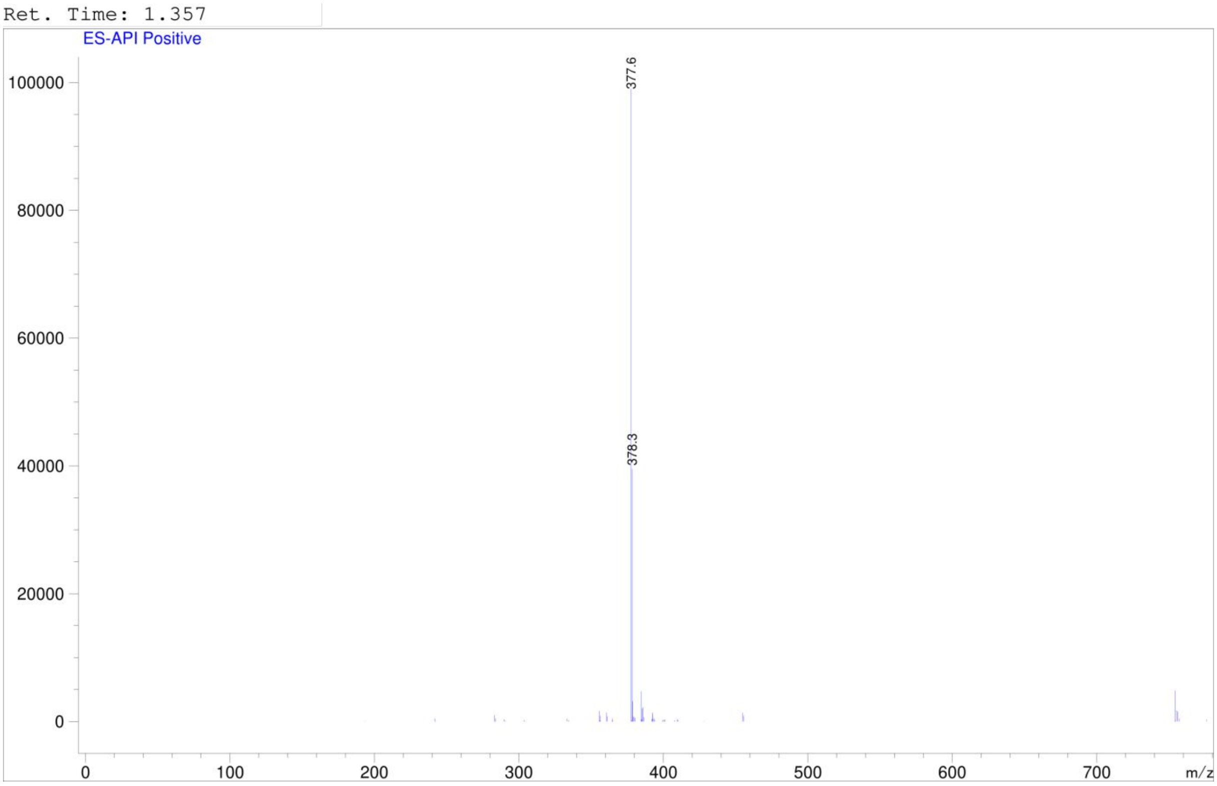

Figure S62: ESI-MS spectrum of compound **S-18** with $[M/2+H]^{+}_{calc.}$ = 377.7 m/z.

***Tert*-butyl (2-((2-(1-(4-chlorophenyl)cyclohexyl)-7-(4-(15-(5,5-difluoro-7-(1H-pyrrol-2-yl)-5H-4l4,5l4-dipyrrolo[1,2-c:2',1'-f][1,3,2]diazaborinin-3-yl)-13-oxo-3,6,9-trioxa-12-azapentadecanoyl)piperazin-1-yl)quinazolin-4-yl)amino)ethyl)carbamate (S-19)**

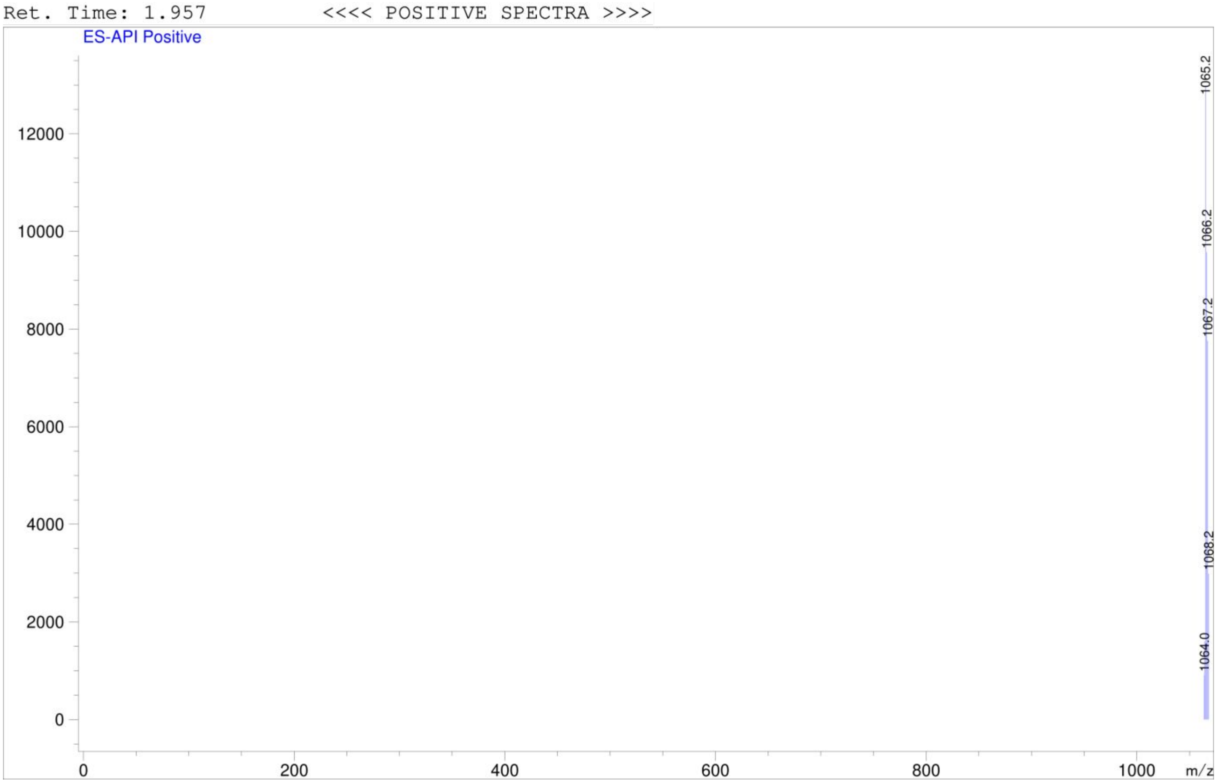

Figure S63: ESI-MS spectrum of compound **S-19** with $[M+H]^{+}_{calc.}$ = 1065.5 m/z.

N-(2-(2-(2-(2-(4-(4-((2-aminoethyl)amino)-2-(1-(4-chlorophenyl)cyclohexyl)quinazolin-7-yl)piperazin-1-yl)-2-oxoethoxy)ethoxy)ethoxy)ethyl)-3-(5,5-difluoro-7-(1H-pyrrol-2-yl)-5H-4l4,5l4-dipyrrolo[1,2-c:2',1'-f][1,3,2]diazaborinin-3-yl)propenamide (S-20)

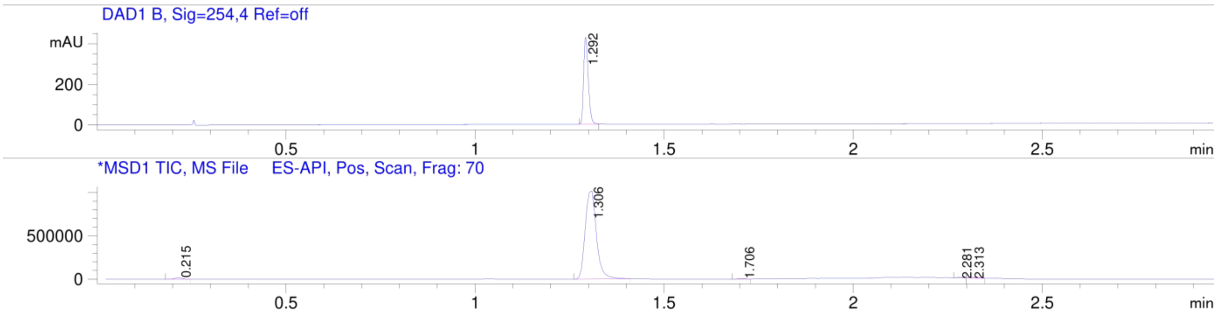

Integration Results for DAD1 B, Sig=254,4 Ref=off

RetTime	Width	Area	Height	Area%	MSBase(+)
1.292	0.013	365.164	429.252	100.000	473.2

Figure S64: LC/MS spectra of purified compound **S-20** at 254 nm wavelength.

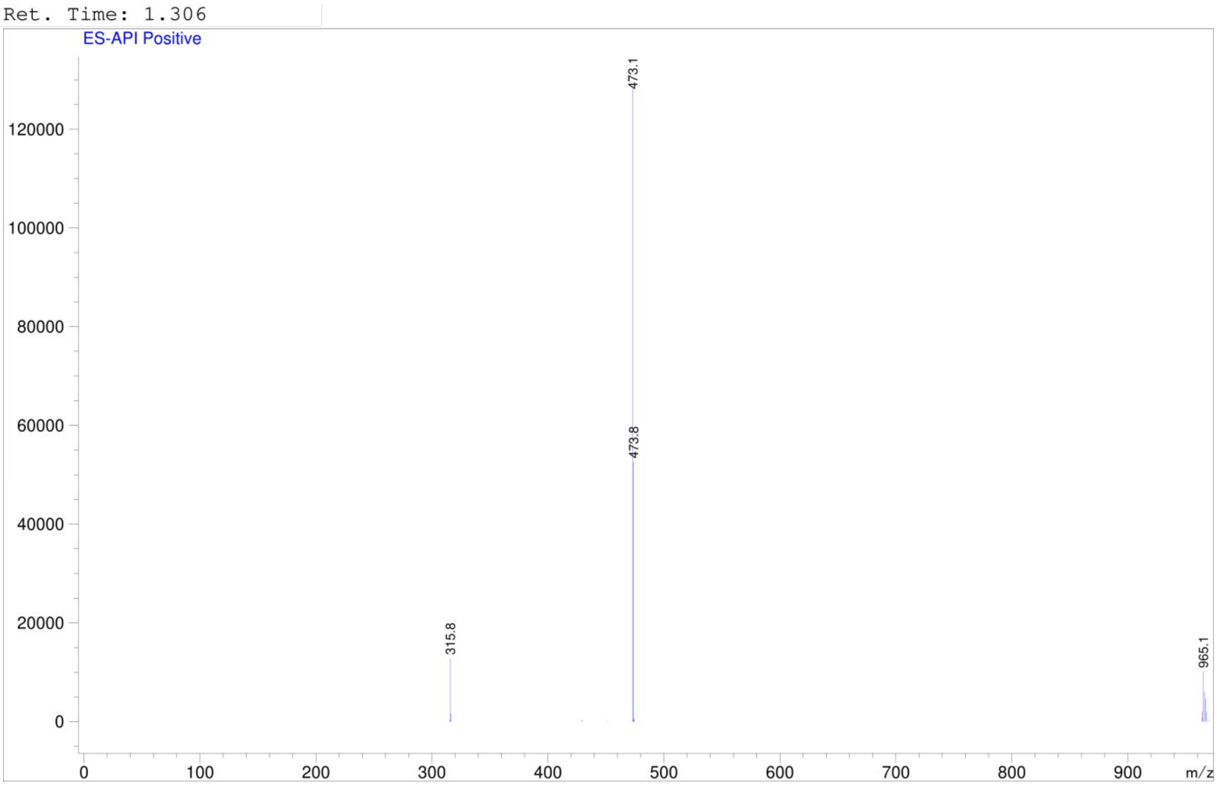

Figure S65: ESI-MS spectrum of compound **S-20** with $[M+H]^{+}_{calc.}$ = 965.5 m/z.

7.2. Promiscuous Kinase PROTACs

7.2.1. DCAF1-recruiting Promiscuous Kinase PROTACs based on Kinase Parent Inhibitor 1inh

***Tert*-butyl 3-(2-(2-(4-(2-((4-(cyanomethyl)phenyl)amino)-6-((5-cyclopropyl-1*H*-pyrazol-3-yl)amino)pyrimidin-4-yl)piperazin-1-yl)ethoxy)ethoxy)propanoate (S-27)**

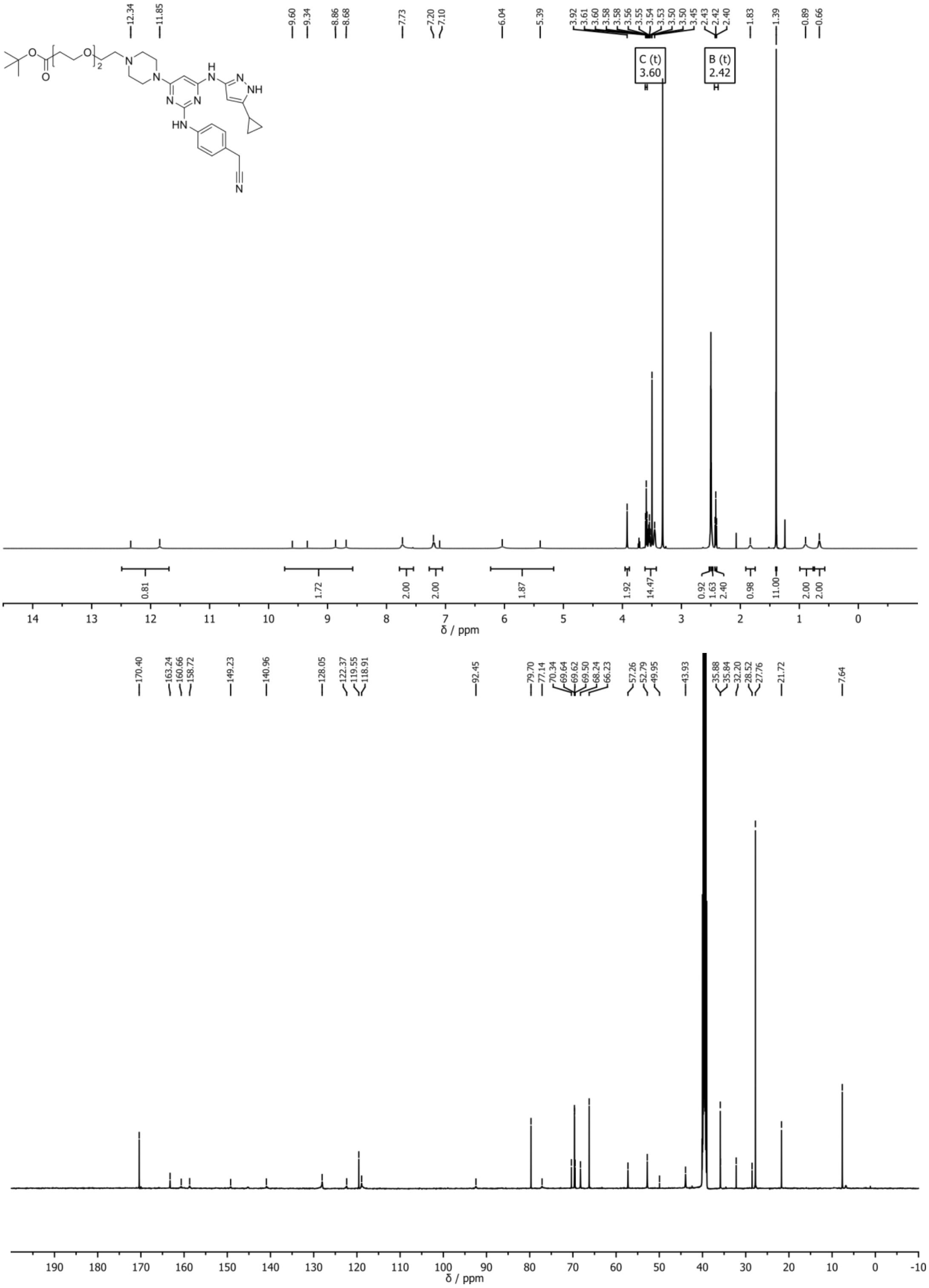

Figure S66: ^{1}H- (top) and ^{13}C-NMR (bottom) spectra (500 MHz and 126 MHz, 298 K, DMSO-d_6) and chemical structure of compound **S-27**.

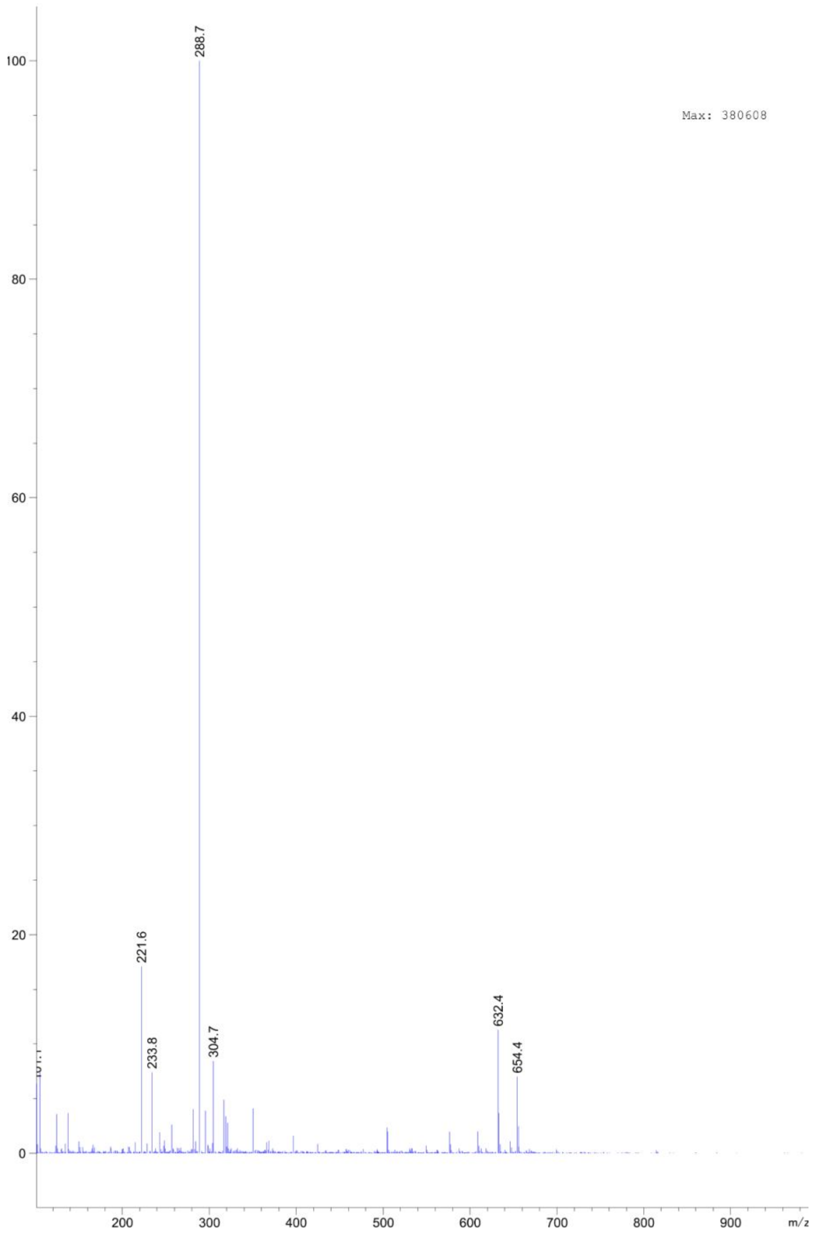

Figure S67: ESI-MS spectrum of compound **S-27** with $[M+H]^{+}_{calc.}$ = 632.36 m/z.

2-(4-((4-(4-(2-(2-(3-(4-(4-((2-aminoethyl)amino)-2-(1-(4-chlorophenyl)cyclohexyl)quinazolin-7-yl)piperazin-1-yl)-3-oxopropoxy)ethoxy)ethyl)piperazin-1-yl)-6-((5-cyclopropyl-1*H*-pyrazol-3-yl)amino)pyrimidin-2-yl)amino)phenyl)acetonitrile (TFA salt, 1:2) (D-1a)

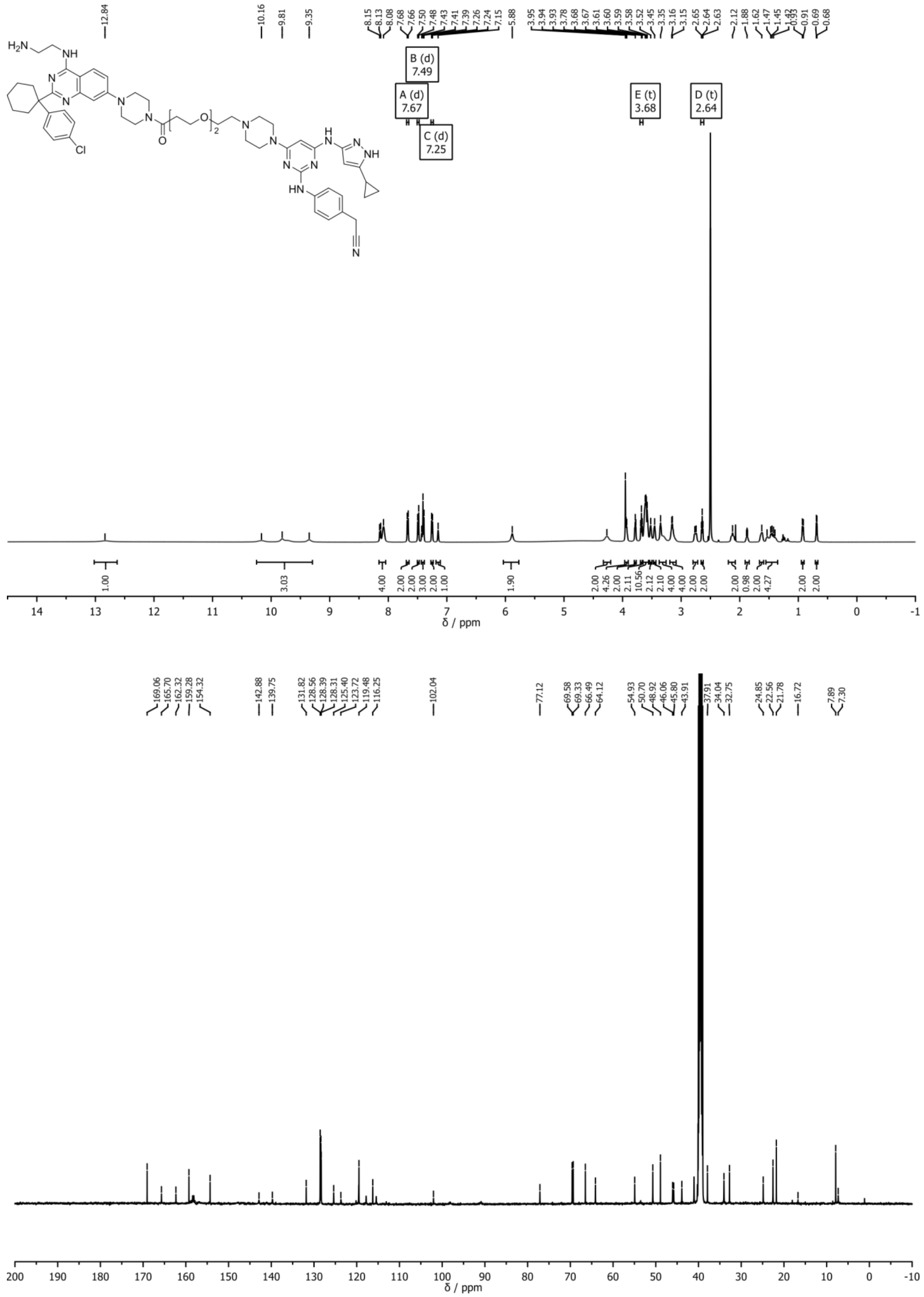

Figure S68: ^{1}H- (top) and ^{13}C-NMR (bottom) spectra (500 MHz and 126 MHz, 298 K, DMSO-d_6) and chemical structure of compound **D-1a**.

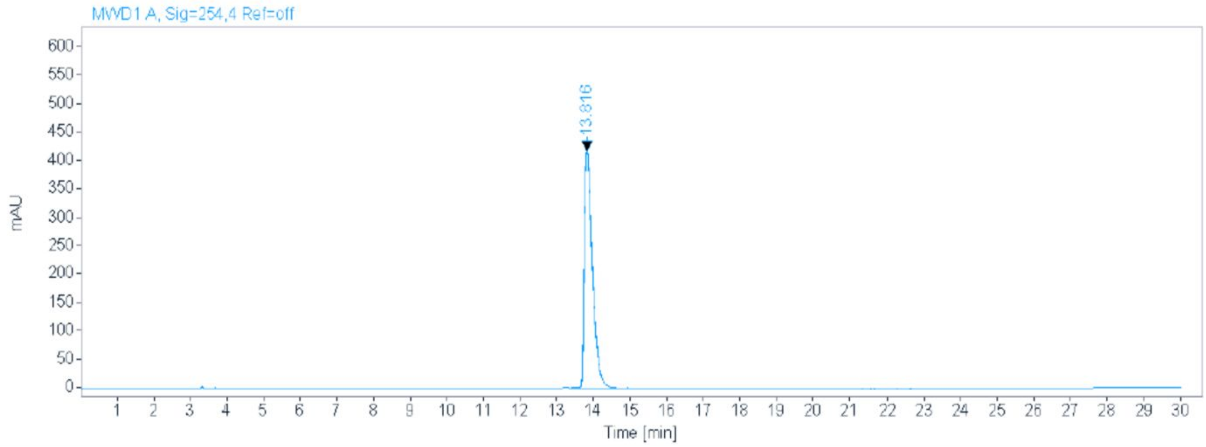

Signal:	MWD1 A, Sig=254,4 Ref=off					
RT [min]	Type	Width [min]	Area	Height	Area%	Name
13.816	VB	0.2377	6625.2021	418.5668	100.0000	
		Sum	6625.2021			

Figure S69: LC/MS spectra of purified compound **D-1a** at 254 nm wavelength.

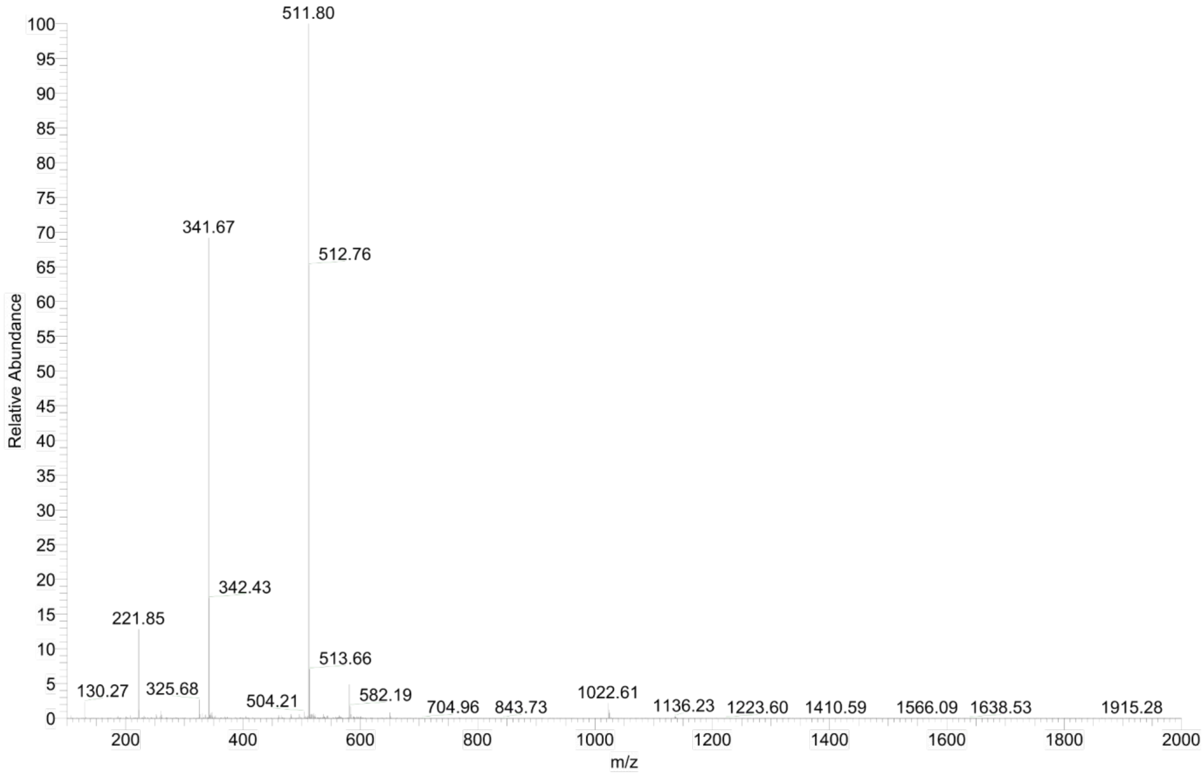

Figure S70: ESI-MS spectrum of compound **D-1a** with $[M+H]^+_{calc.}$ = 1022.36 m/z.

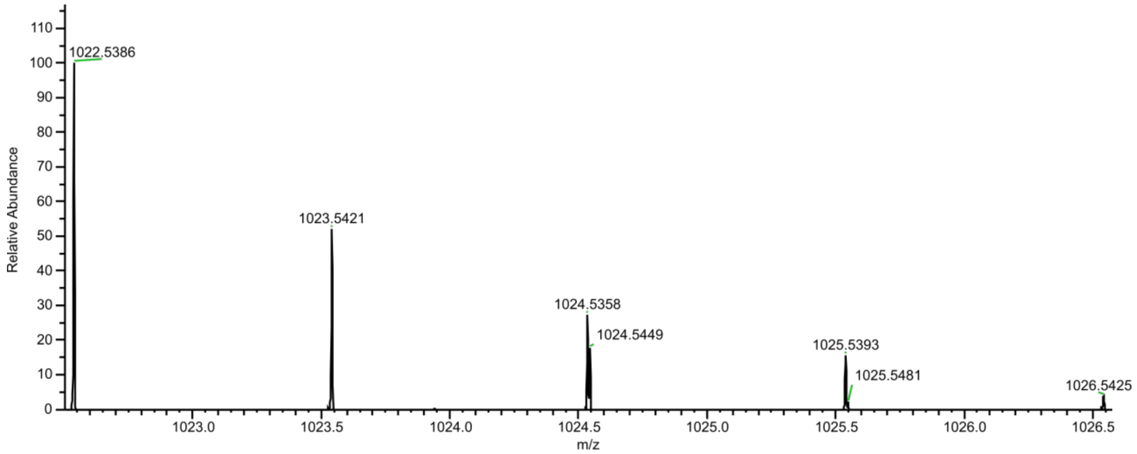

Figure S71: High-resolution mass spectrum of compound **D-1a** with $[M+H]^{+}_{calc.}$ = 1022.5391 m/z.

Tert-butyl (2-((2-(1-(4-chlorophenyl)cyclohexyl)-7-(4-(3-(2-(2-(4-(2-((4-(cyanomethyl)phenyl)amino)-6-((5-cyclopropyl-1_H_-pyrazol-3-yl)amino)pyrimidin-4-yl)piperazin-1-yl)ethoxy)ethoxy)propanoyl)piperazin-1-yl)quinazolin-4-yl)amino)ethyl)carbamate _(D-1a[n.c.])_

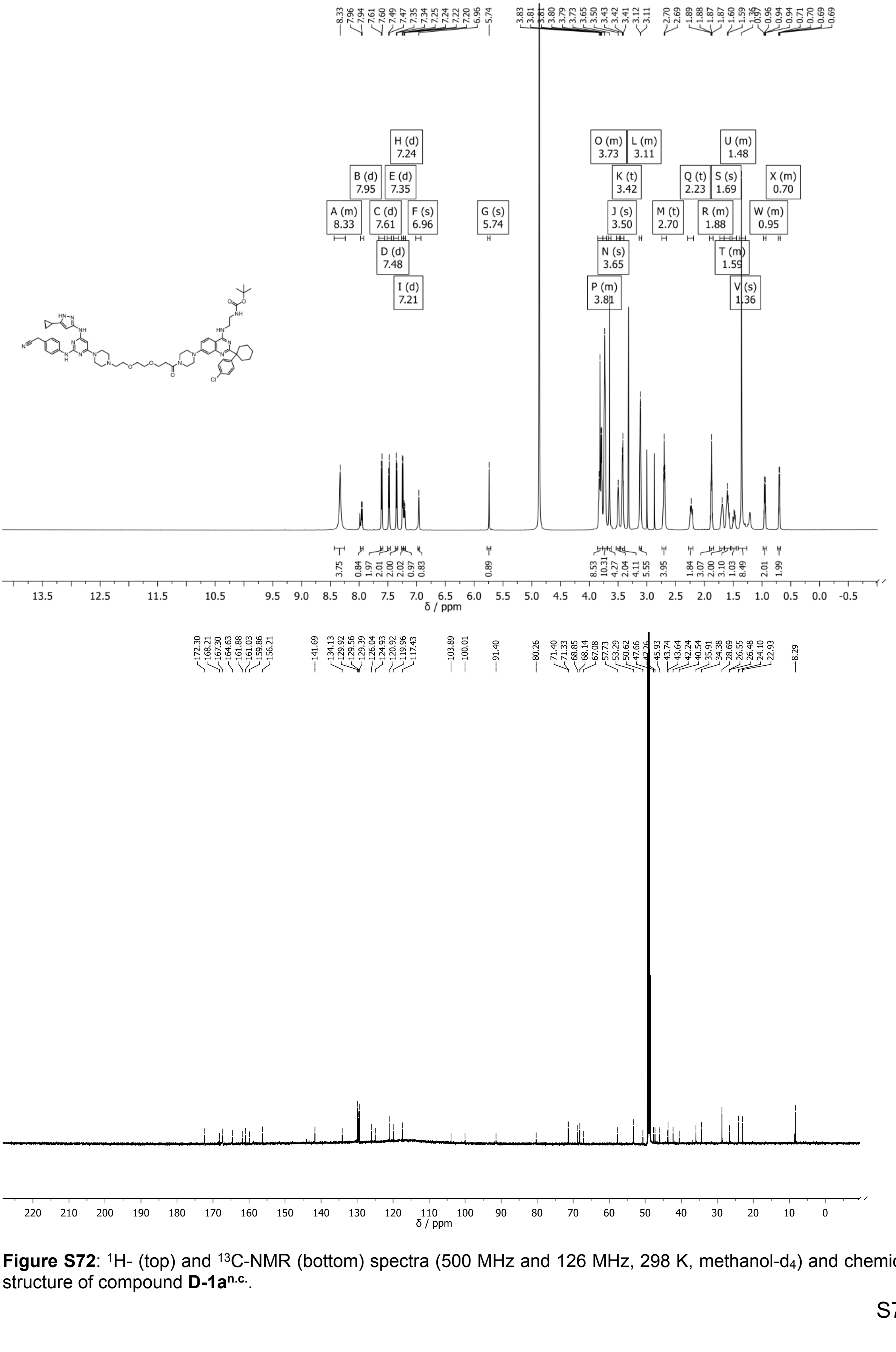

Figure S72: ^{1}H- (top) and ^{13}C-NMR (bottom) spectra (500 MHz and 126 MHz, 298 K, methanol-d_4) and chemical structure of compound **D-1a[n.c.]**.

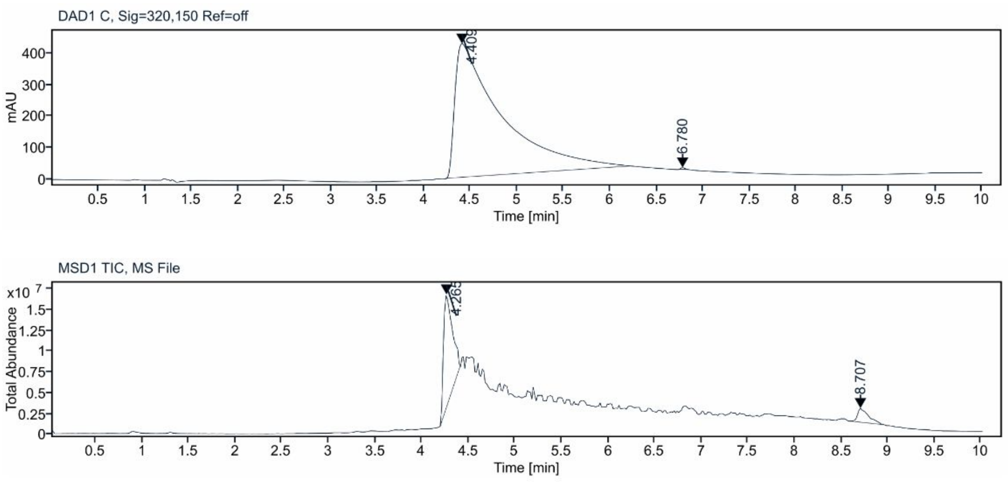

Signal Description DAD1 C, Sig=320,150 Ref=off

Sample Name	Name	RT	Width	Area	Area%	Height
JW-619 38		4.409	0.468	14875.21 97	99.89	424.6808
JW-619 38		6.780	0.050	16.5691	0.11	5.4281

Max Area% 99.889

UV Signal Purity>95% Pass

Figure S73: LC/MS spectra of purified compound **D-1a**$^{n.c.}$ at 320 nm wavelength.

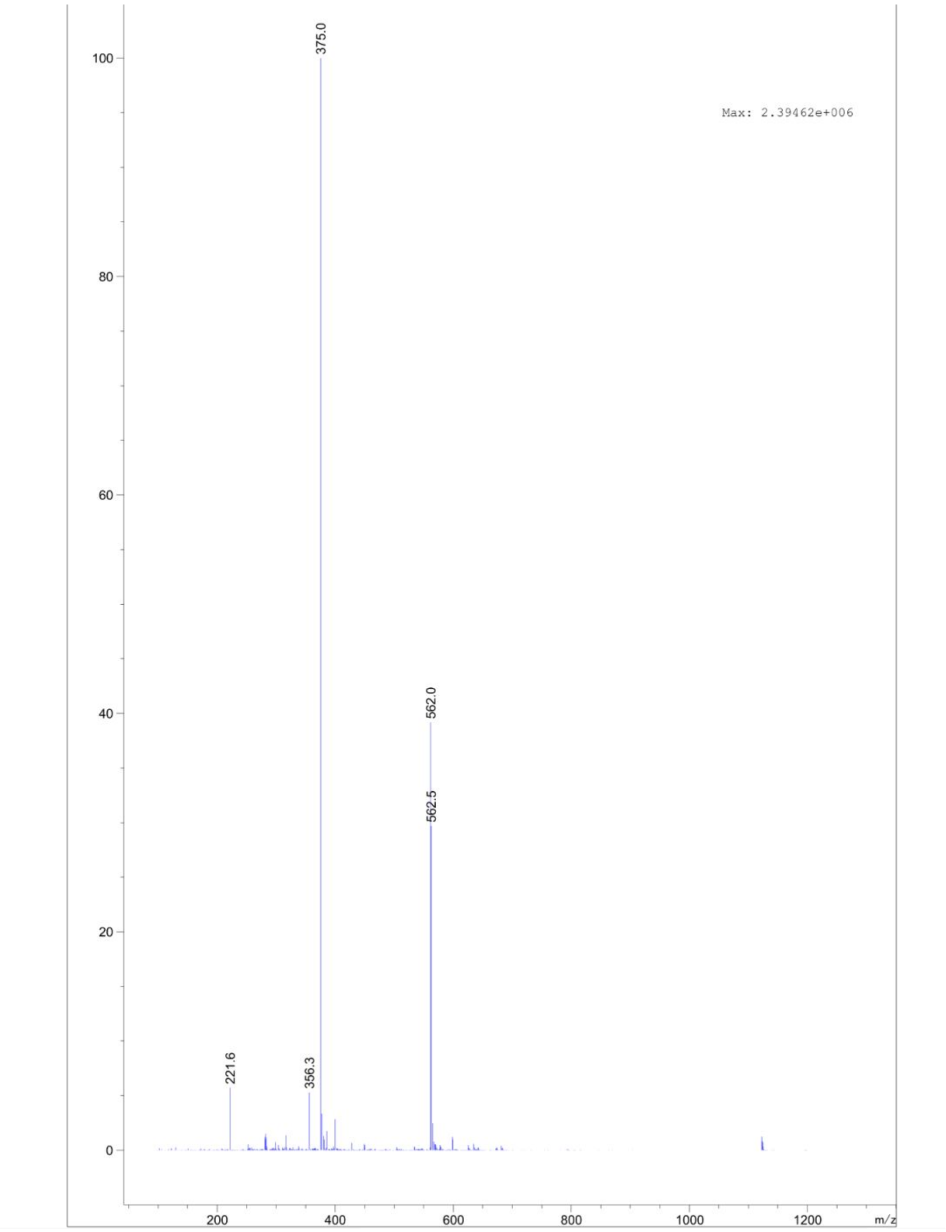

Figure 74: ESI-MS spectrum of compound **D-1a**[n.c.] with $[M/2+H]^+_{calc.}$ = 561.8 m/z.

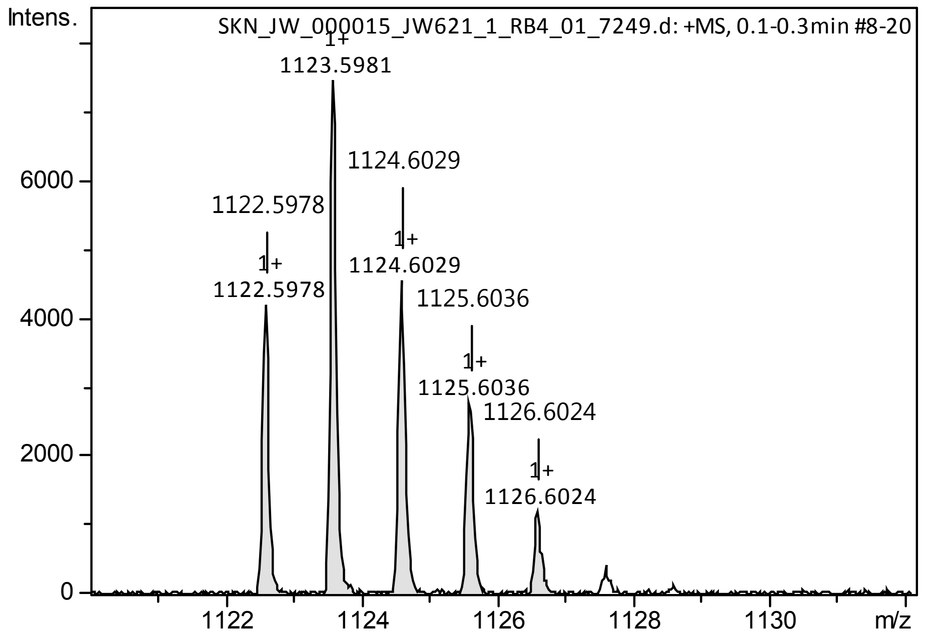

Figure 75: High-resolution mass spectrum of compound **D-1a**$^{n.c.}$ with $[M+H]^{+}_{calc.}$ = 1122.5915 m/z

2-(4-((4-(4-(2-(2-(2-(3-(4-(4-((2-aminoethyl)amino)-2-(1-(4-chlorophenyl)cyclohexyl)quinazolin-7-yl)piperazin-1-yl)-3-oxopropoxy)ethoxy)ethoxy)ethyl)piperazin-1-yl)-6-((5-cyclopropyl-1H-pyrazol-3-yl)amino)pyrimidin-2-yl)amino)phenyl)acetonitrile (TFA salt , 1:2) (D-1b)

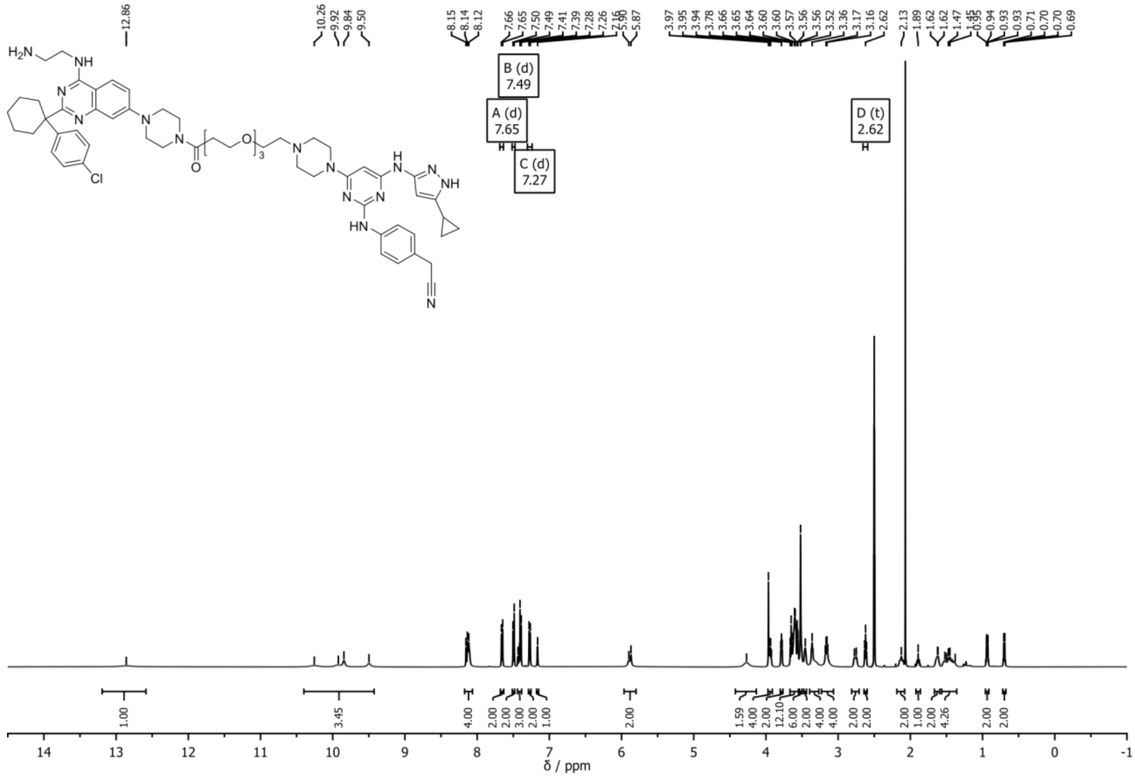

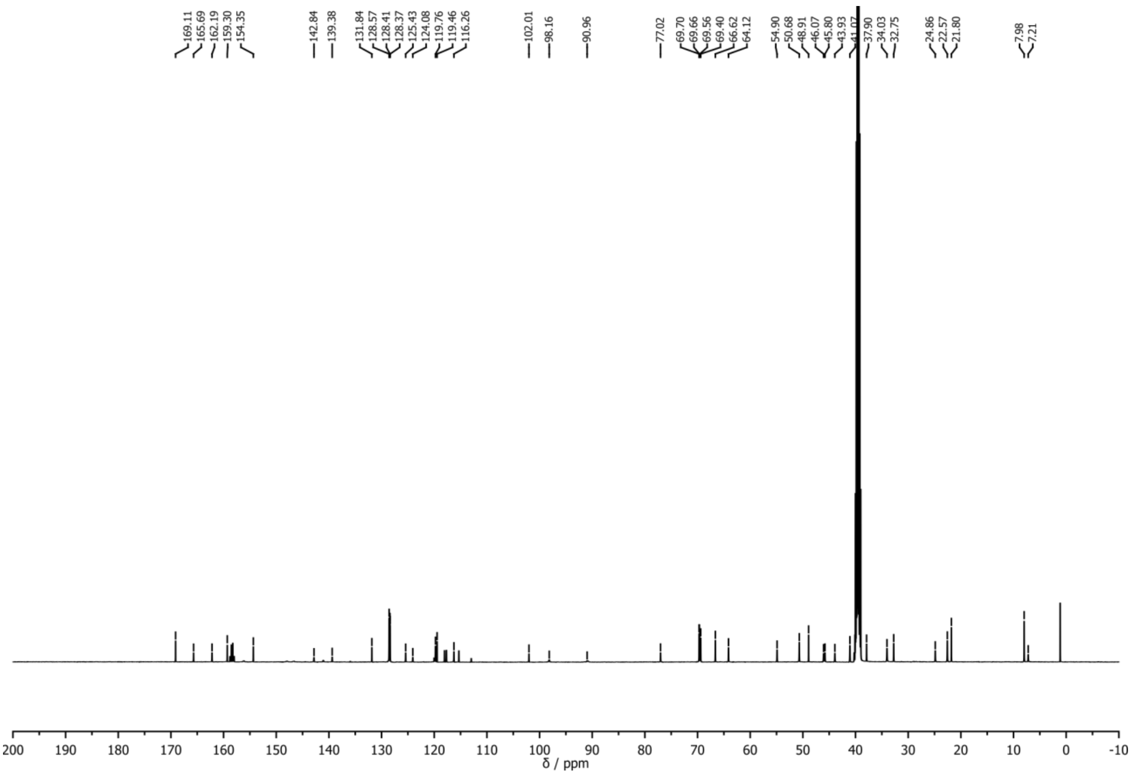

Figure S76: ^{1}H- (top) and ^{13}C-NMR (bottom) spectra (500 MHz and 126 MHz, 298 K, DMSO-d_6) and chemical structure of compound **D-1b**.

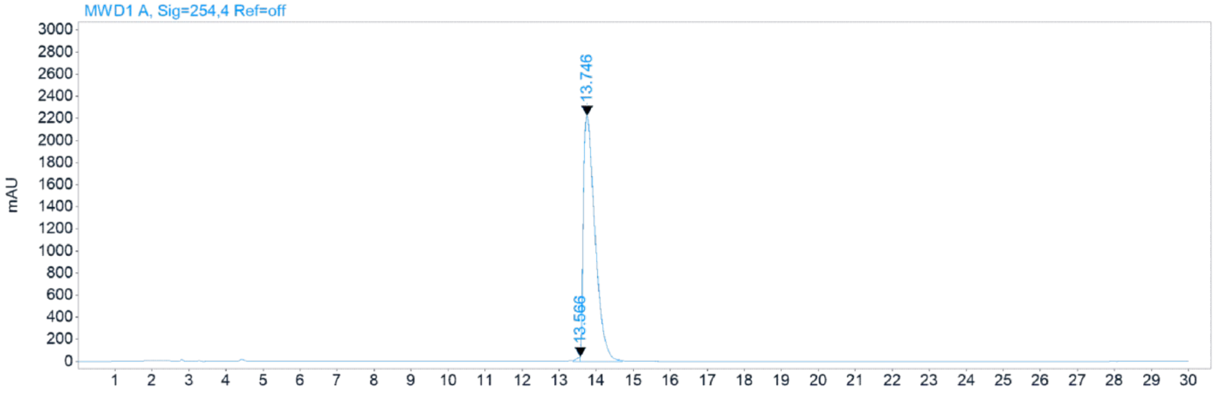

Signal: MWD1 A, Sig=254,4 Ref=off

RT [min]	Type	Width [min]	Area	Height	Area%	Name
13.566	MF	0.1141	312.3837	45.6470	0.6384	
13.746	FM	0.3636	48618.9023	2228.6003	99.3616	
		Sum	48931.2860			

Figure S77: LC/MS spectra of purified compound **D-1a**$^{n.c.}$ at 254 nm wavelength.

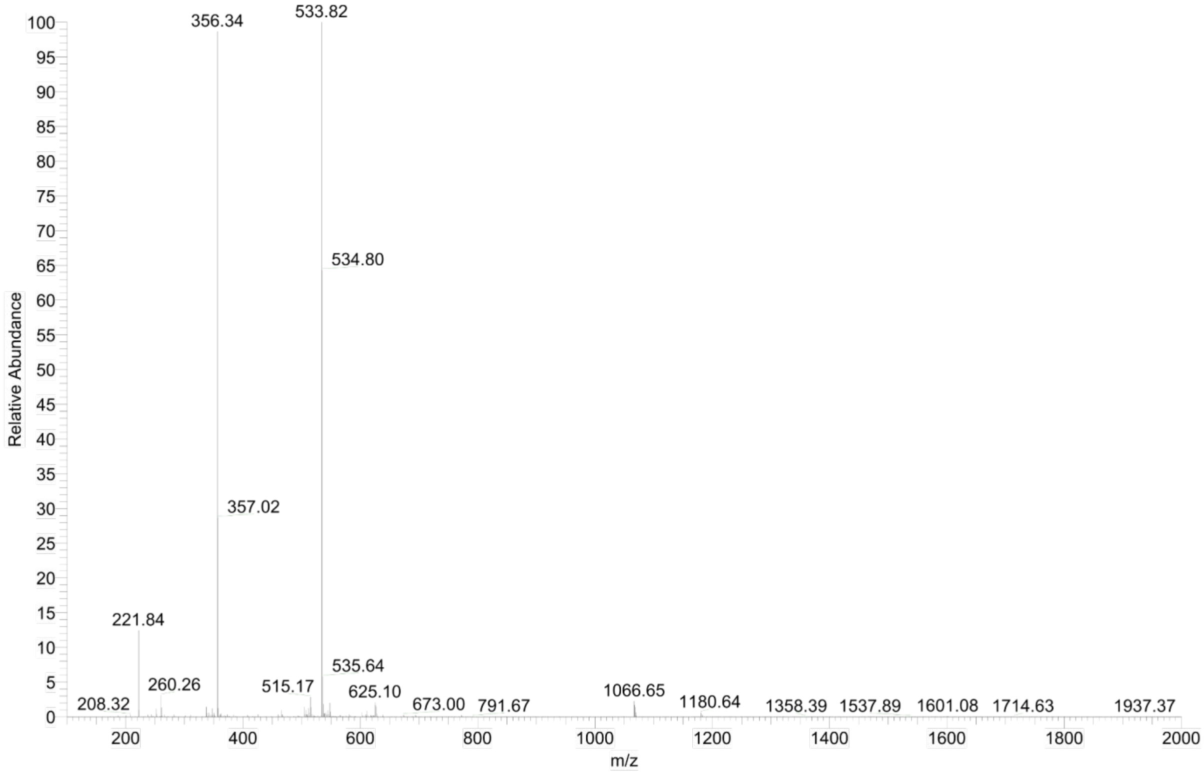

Figure S78: ESI-MS spectrum of compound **D-1b** with $[M+H]^{+}_{calc.}$ = 1066.56 m/z.

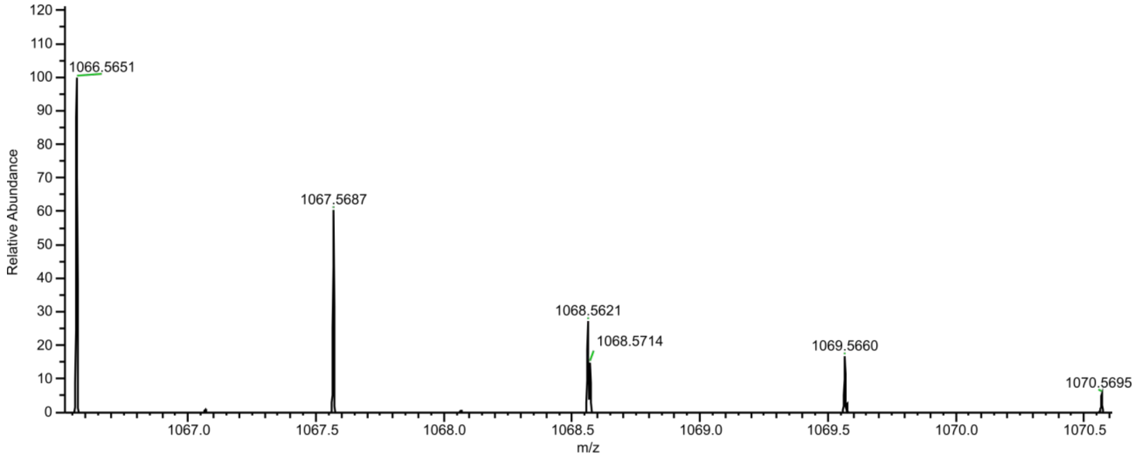

Figure S79: High-resolution mass spectrum of compound **D-1b** with $[M+H]^{+}_{calc.}$ = 1066.5653 m/z.

***Tert*-butyl 1-(4-(2-((4-(cyanomethyl)phenyl)amino)-6-((5-cyclopropyl-1*H*-pyrazol-3-yl)amino)pyrimidin-4-yl)piperazin-1-yl)-3,6,9,12,15-pentaoxaoctadecan-18-oate (S-29)**

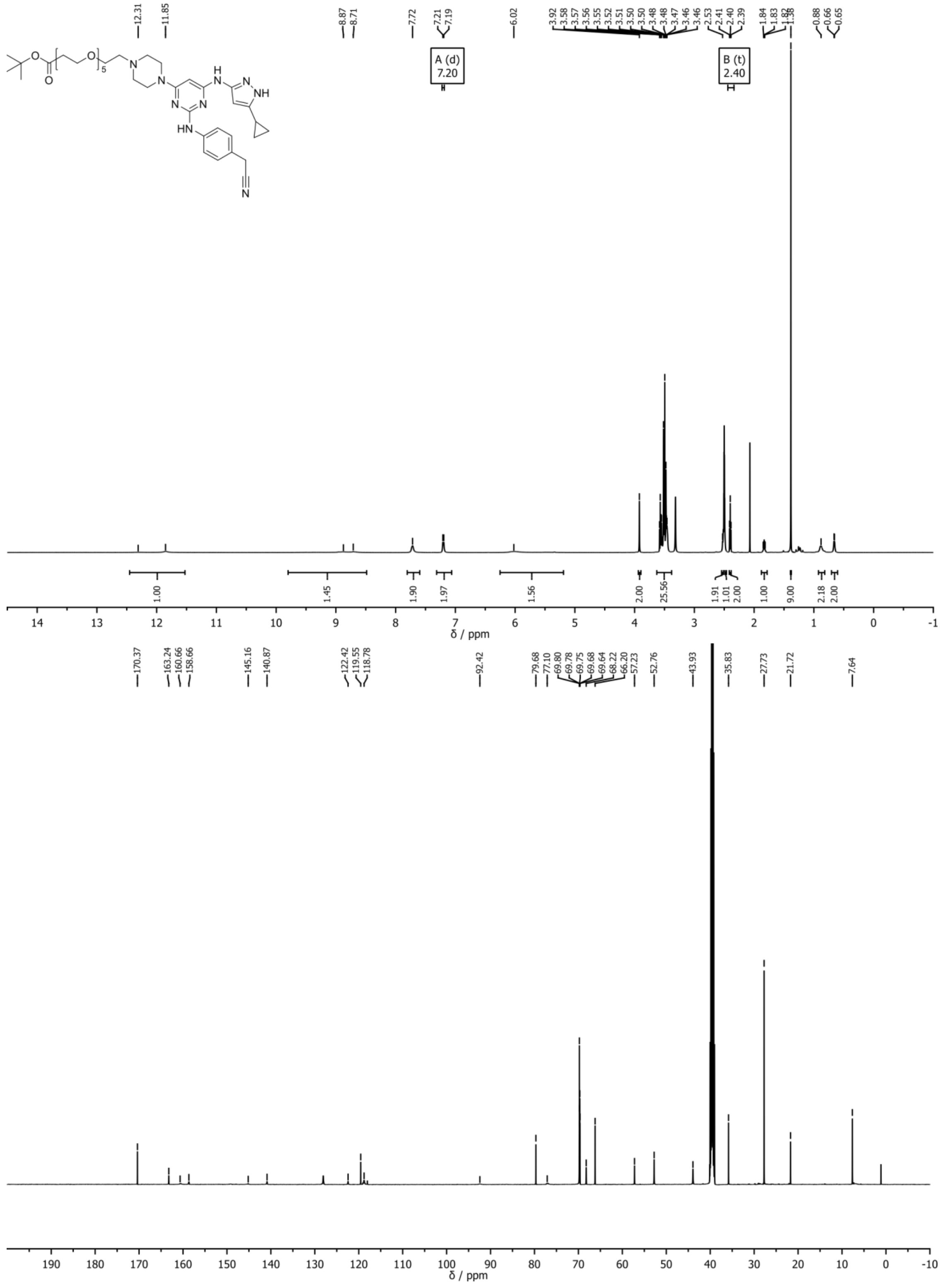

Figure S80: ^{1}H- (top) and ^{13}C-NMR (bottom) spectra (500 MHz and 126 MHz, 298 K, DMSO-d_6) and chemical structure of compound **S-29**.

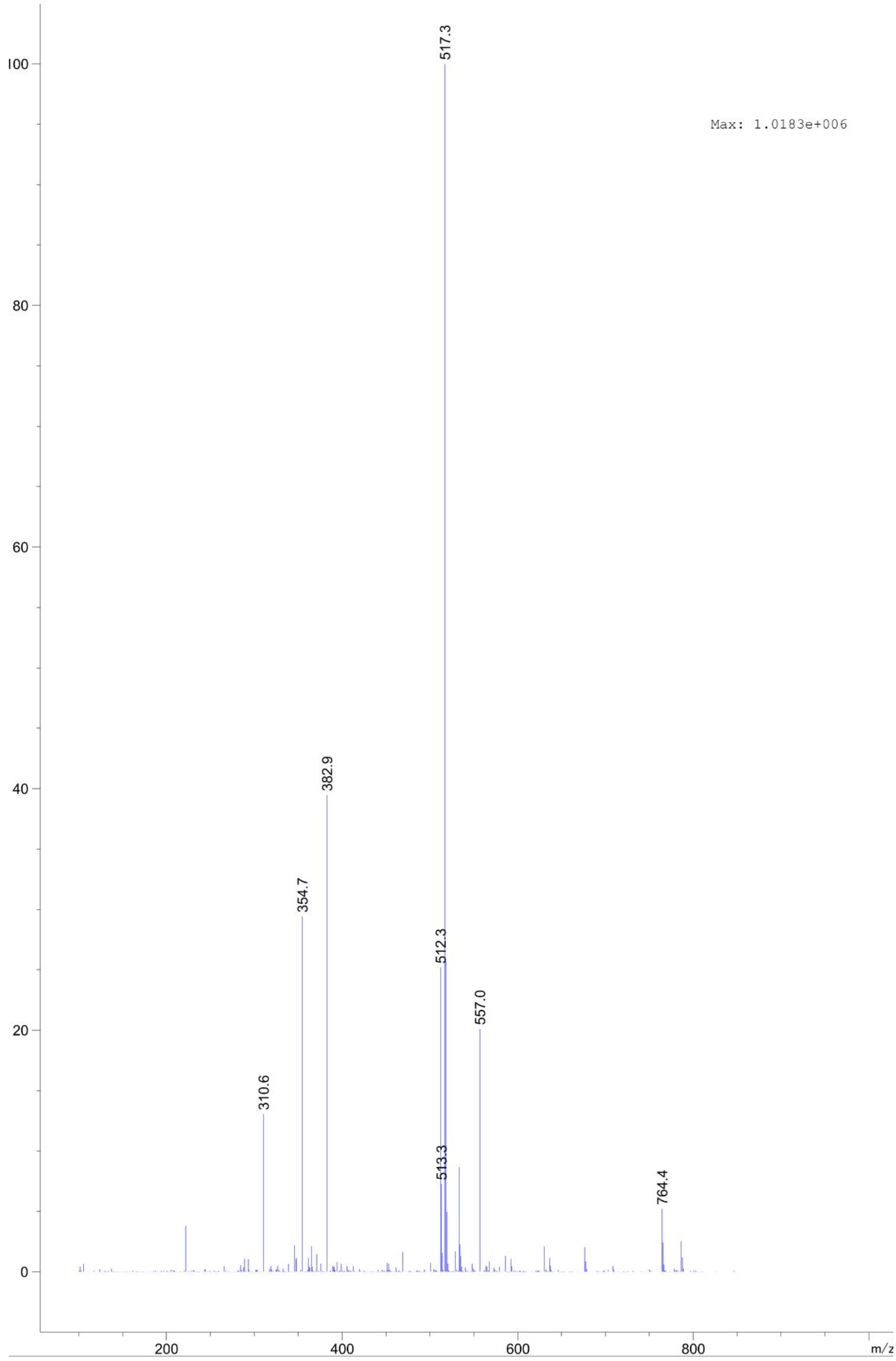

Figure S81: ESI-MS spectrum of compound **S-29** with $[M+H]^+_{calc.}$ = 764.44 m/z.

2-(4-((4-(4-(18-(4-(4-((2-aminoethyl)amino)-2-(1-(4-chlorophenyl)cyclohexyl)quinazolin-7-yl)piperazin-1-yl)-18-oxo-3,6,9,12,15-pentaoxaoctadecyl)piperazin-1-yl)-6-((5-cyclopropyl-1*H*-pyrazol-3-yl)amino)pyrimidin-2-yl)amino)phenyl)acetonitrile (TFA salt, 1:2) (D-1c)

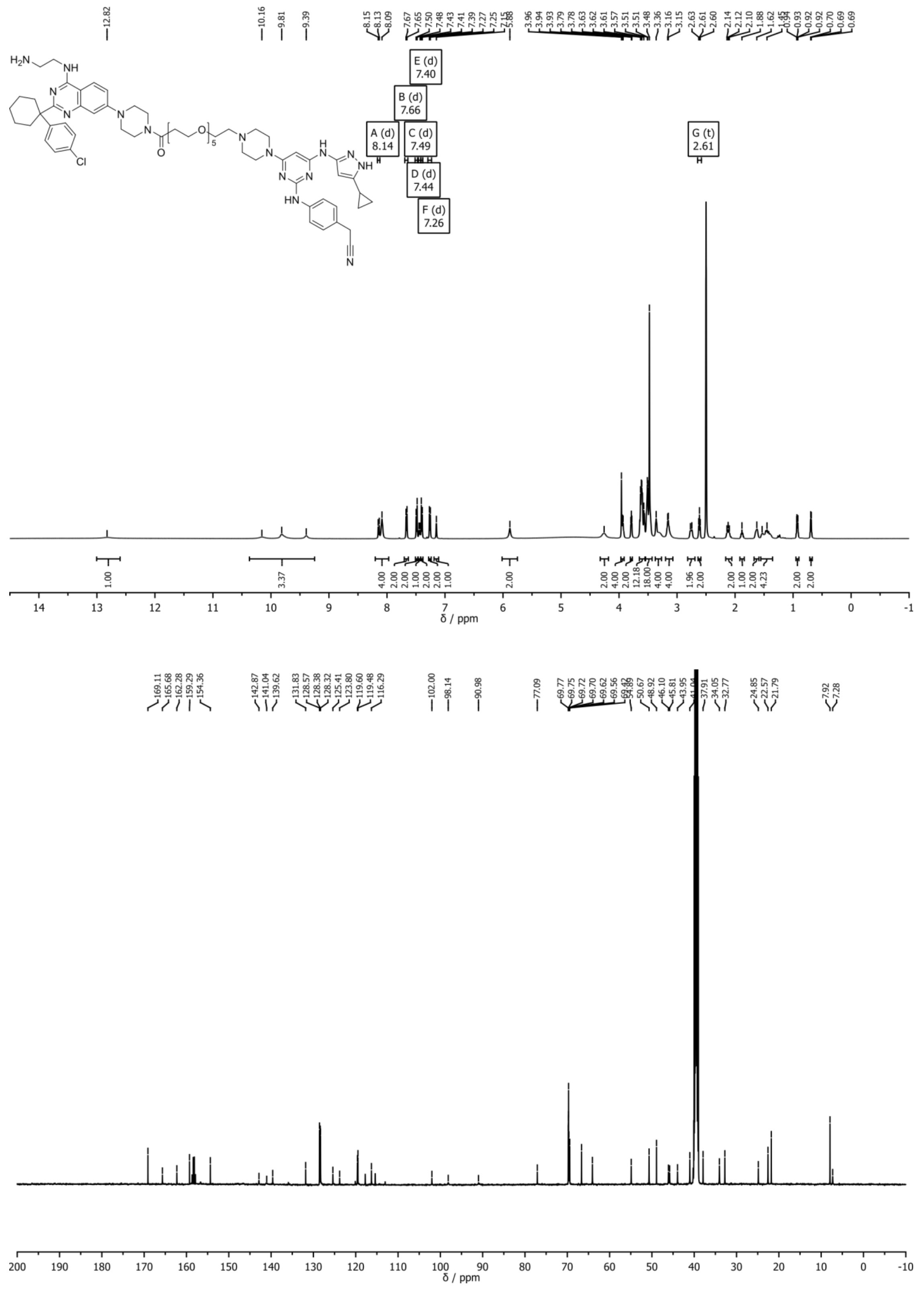

Figure S82: ^{1}H- (top) and ^{13}C-NMR (bottom) spectra (500 MHz and 126 MHz, 298 K, DMSO-d_6) and chemical structure of compound **D-1c**.

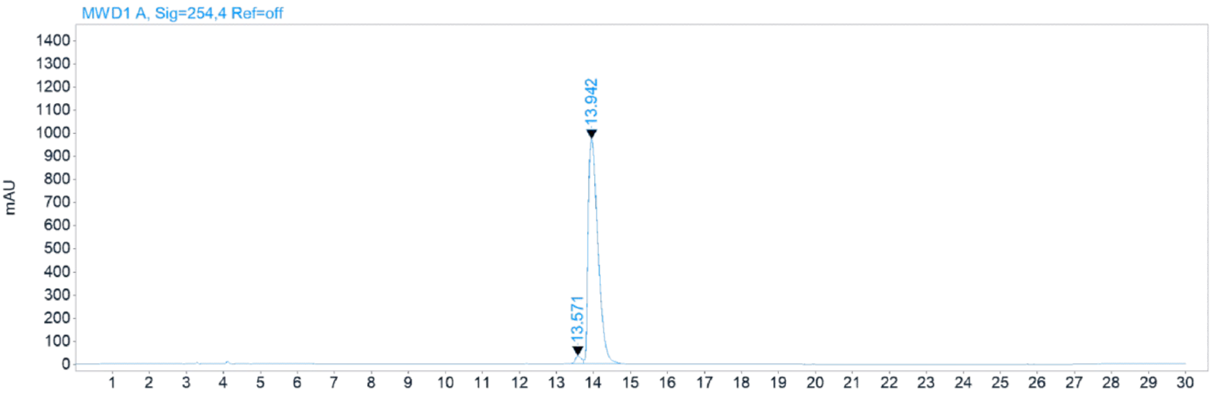

Signal: MWD1 A, Sig=254,4 Ref=off

RT [min]	Type	Width [min]	Area	Height	Area%	Name
13.571	MF	0.1936	390.5359	33.6183	2.1458	
13.942	FM	0.3054	17809.3477	972.0581	97.8542	
		Sum	18199.8835			

Figure S83: LC/MS spectra of purified compound **D-1c** at 254 nm wavelength.

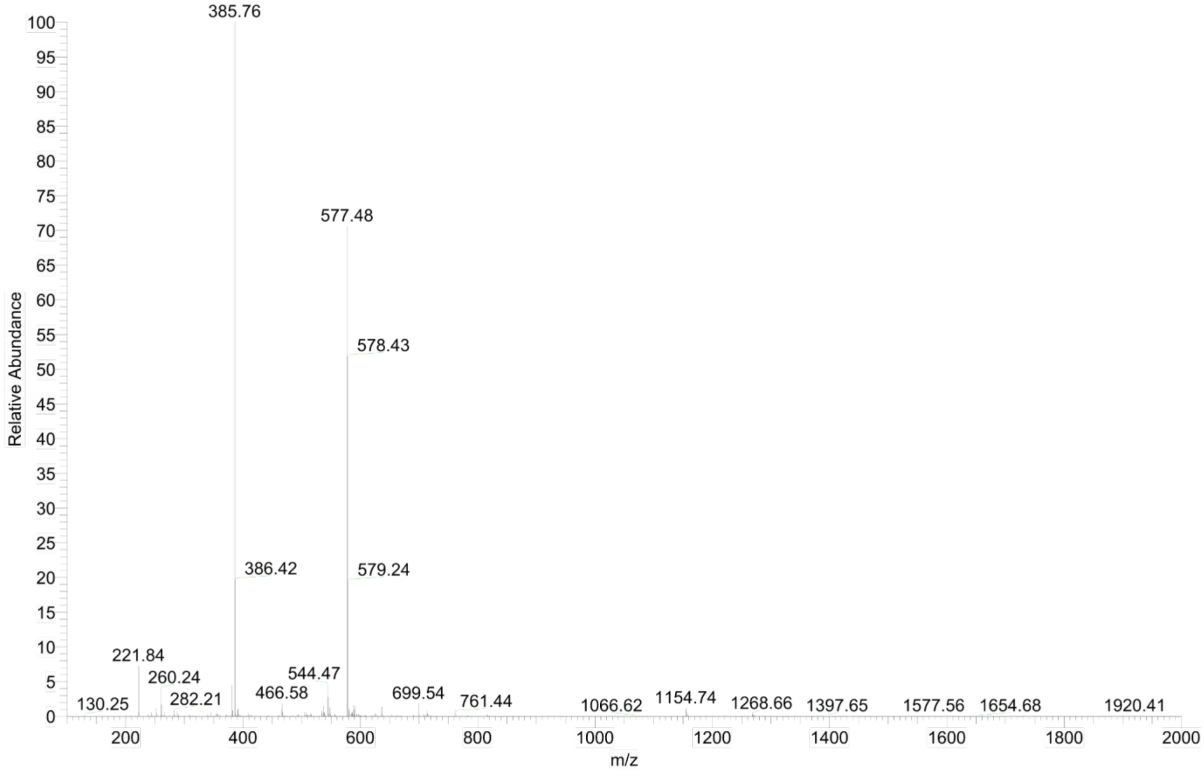

Figure S84: ESI-MS spectrum of compound **D-1c** with $[M+H]^+_{calc.}$ = 1154.61 m/z.

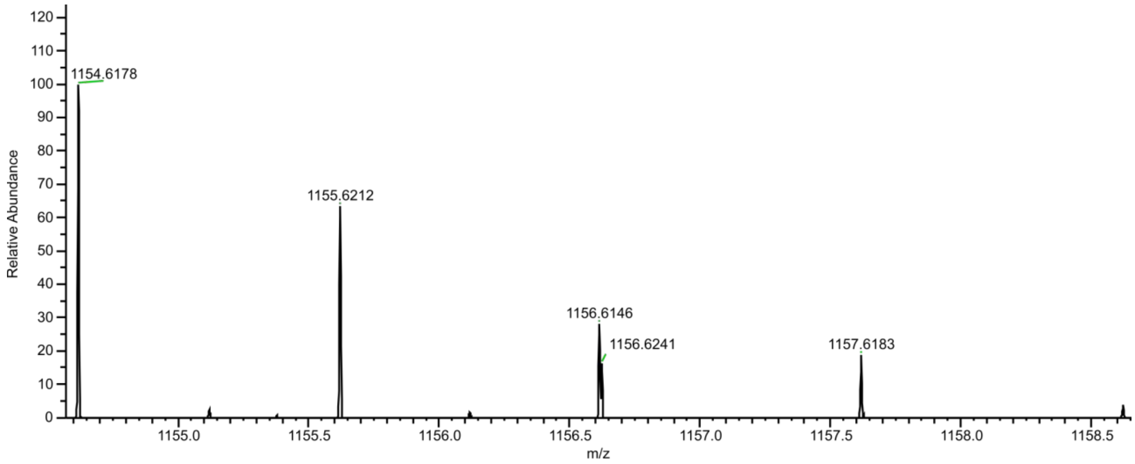

Figure S85: High-resolution mass spectrum of compound **D-1c** with $[M+H]^{+}_{calc.}$ = 1154.6177 m/z.

Ethyl 7-(4-(4-((4-(cyanomethyl)phenyl)amino)-6-((5-cyclopropyl-1H-pyrazol-3-yl)amino)pyrimidin-2-yl)piperazin-1-yl)heptanoate (S-32)

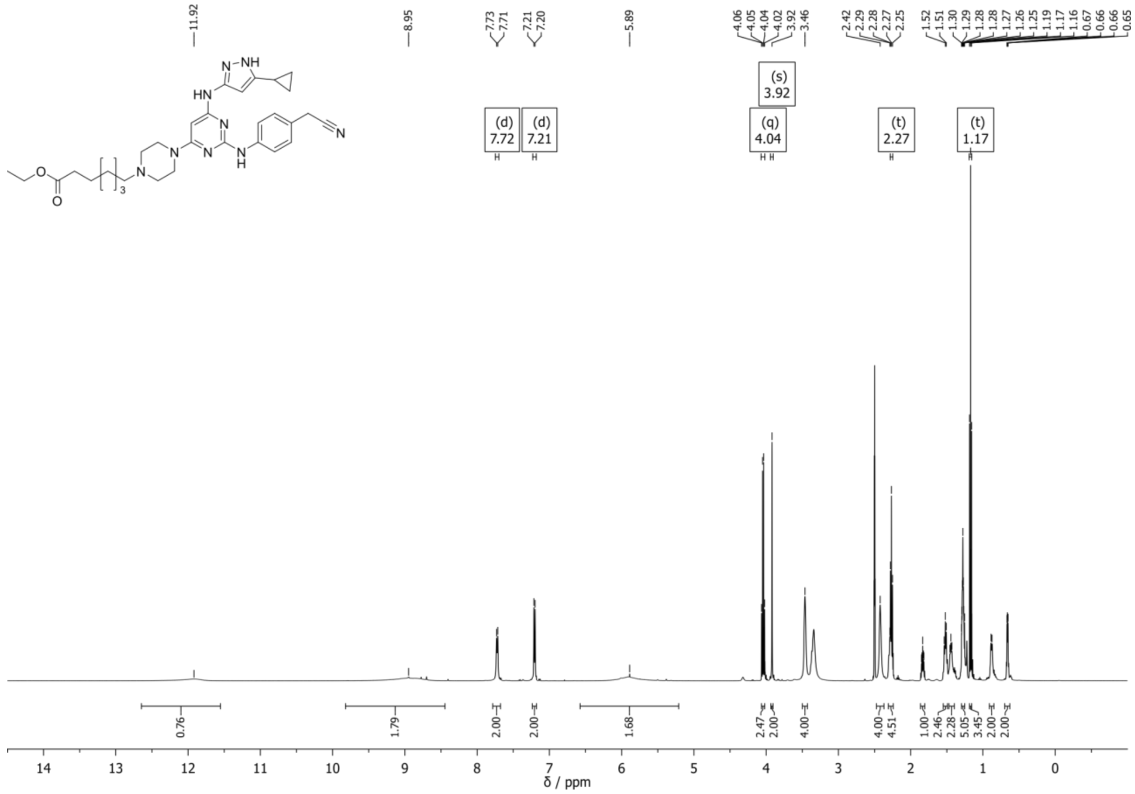

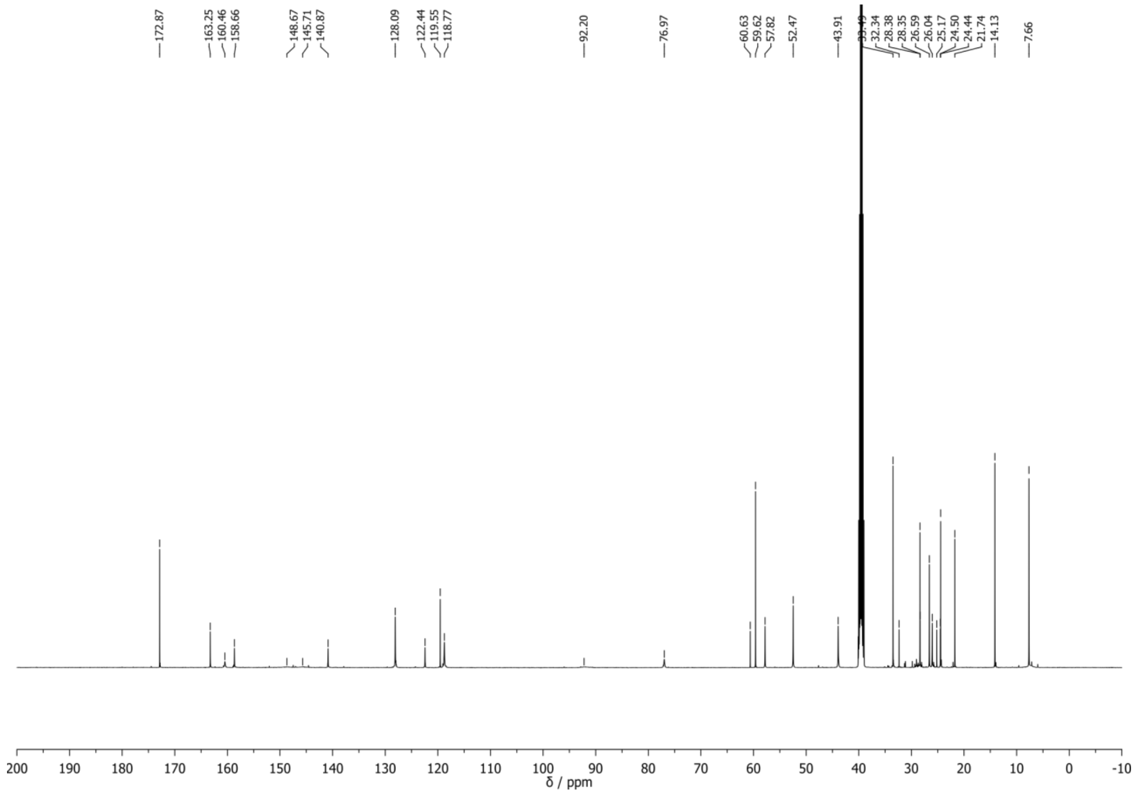

Figure S86: ^{1}H- (top) and ^{13}C-NMR (bottom) spectra (500 MHz and 126 MHz, 298 K, DMSO-d_6) and chemical structure of compound **S-32**.

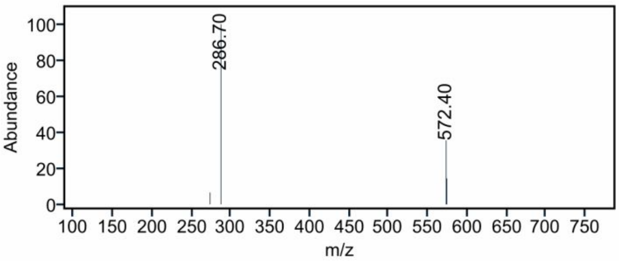

Figure S87: ESI-MS spectrum of compound **S-32** with $[M+H]^+_{calc.}$ = 572.34 m/z.

2-(4-((4-(4-(7-(4-(4-((2-aminoethyl)amino)-2-(1-(4-chlorophenyl)cyclohexyl)quinazolin-7-yl)piperazin-1-yl)-7-oxoheptyl)piperazin-1-yl)-6-((5-cyclopropyl-1*H*-pyrazol-3-yl)amino)pyrimidin-2-yl)amino)phenyl)acetonitrile (TFA salt, 1:2) (D-1d)

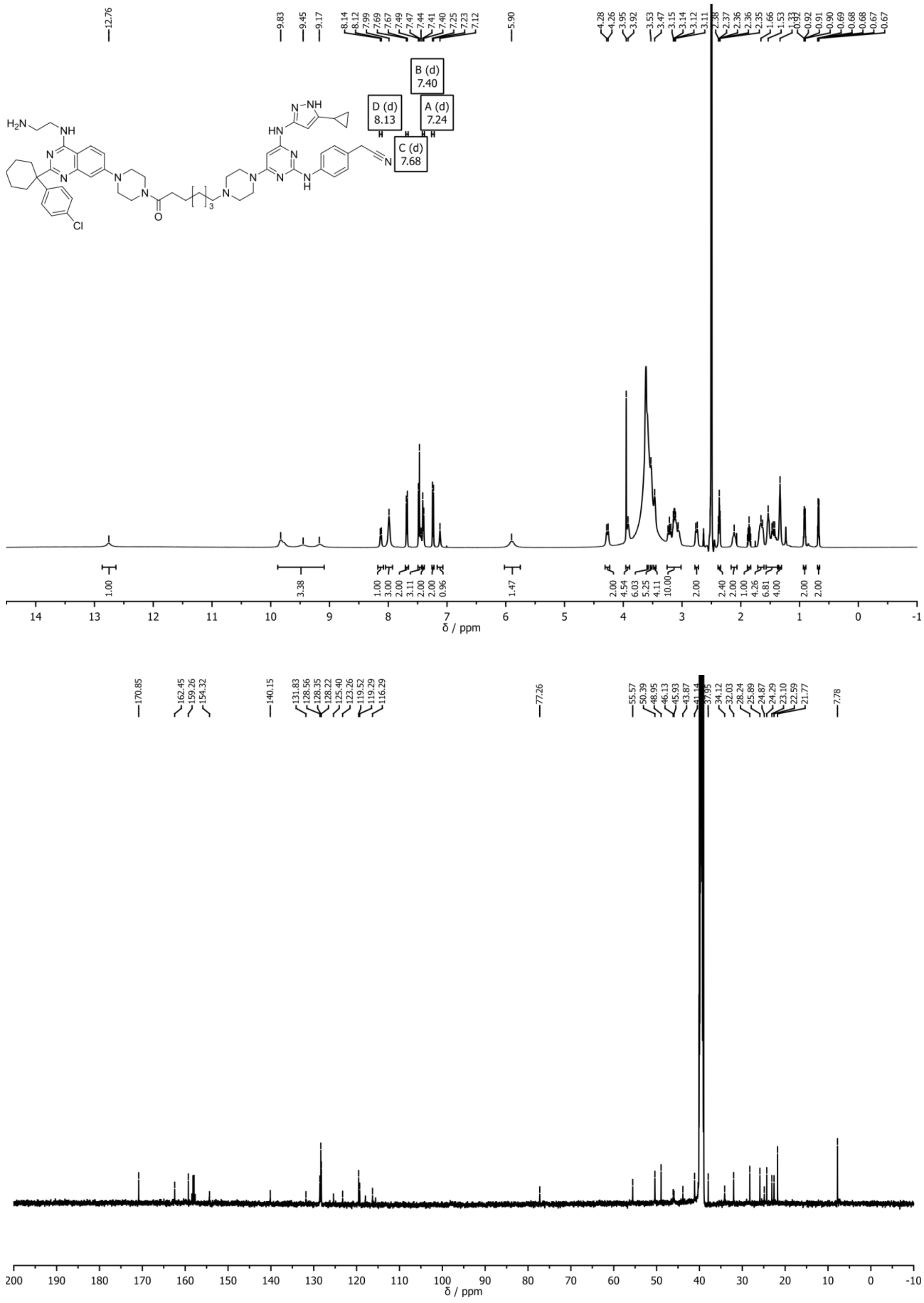

Figure S88: ^{1}H- (top) and ^{13}C-NMR (bottom) spectra (500 MHz and 126 MHz, 298 K, DMSO-d_6) and chemical structure of compound **D-1d**.

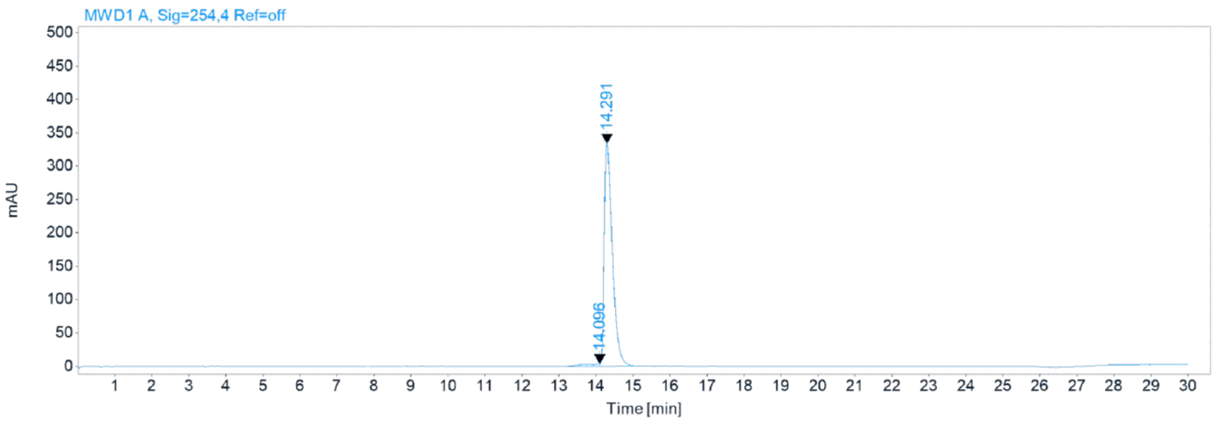

Signal: MWD1 A, Sig=254,4 Ref=off

RT [min]	Type	Width [min]	Area	Height	Area%	Name
14.096	MF	0.5923	152.2551	4.2841	2.8723	
14.291	FM	0.2565	5148.5972	334.5151	97.1277	
		Sum	5300.8523			

Figure S89: LC/MS spectra of purified compound **D-1d** at 254 nm wavelength.

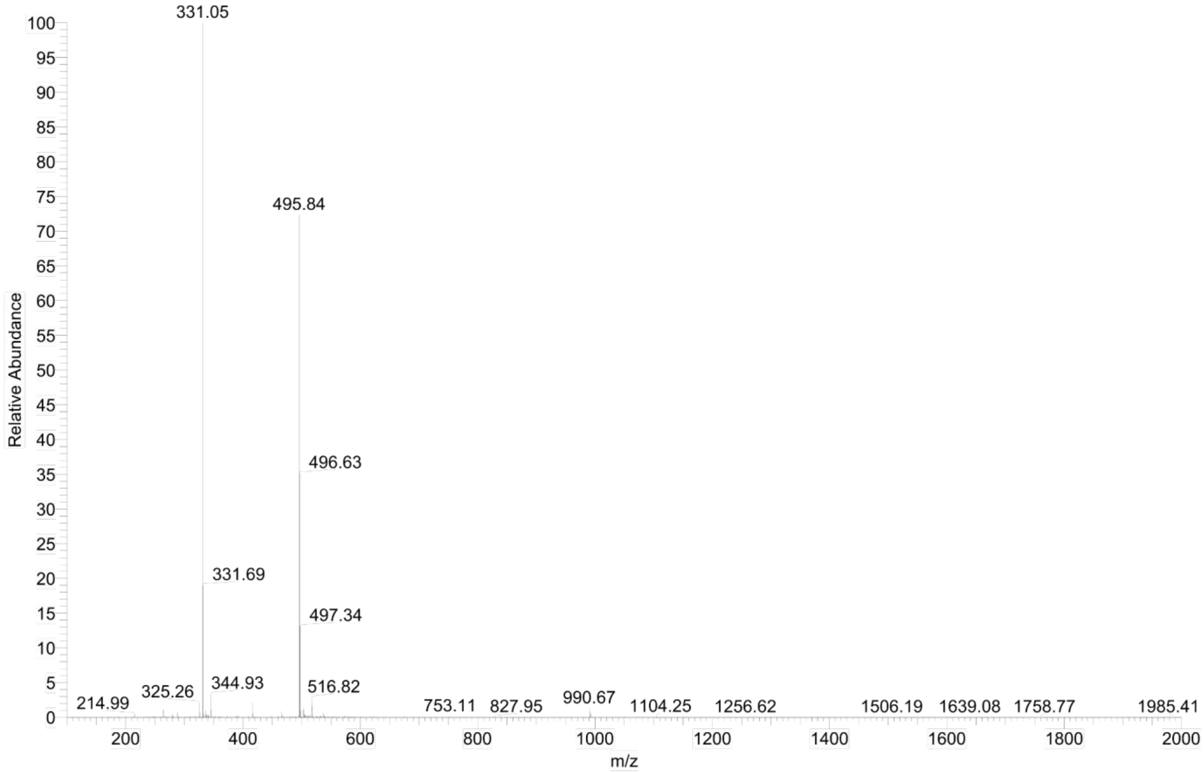

Figure S90: ESI-MS spectrum of compound **D-1d** with $[M+H]^{+}_{calc.}$ = 990.54 m/z.

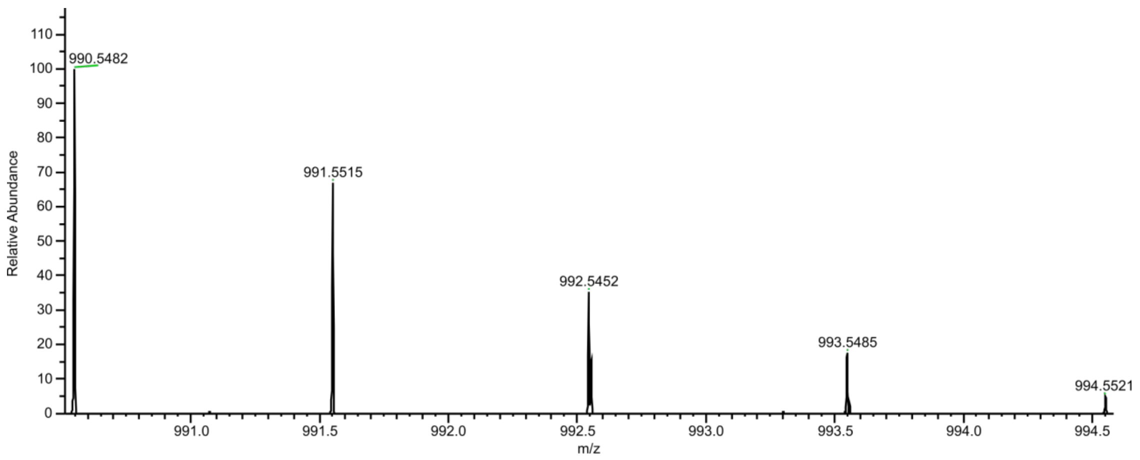

Figure S91: High-resolution mass spectrum of compound **D-1d** with $[M+H]^+_{calc.}$ = 990.5493 m/z.

Ethyl 10-(4-(2-((4-(cyanomethyl)phenyl)amino)-6-((5-cyclopropyl-1*H*-pyrazol-3-yl)amino)pyrimidin-4-yl)piperazin-1-yl)decanoate (S-33)

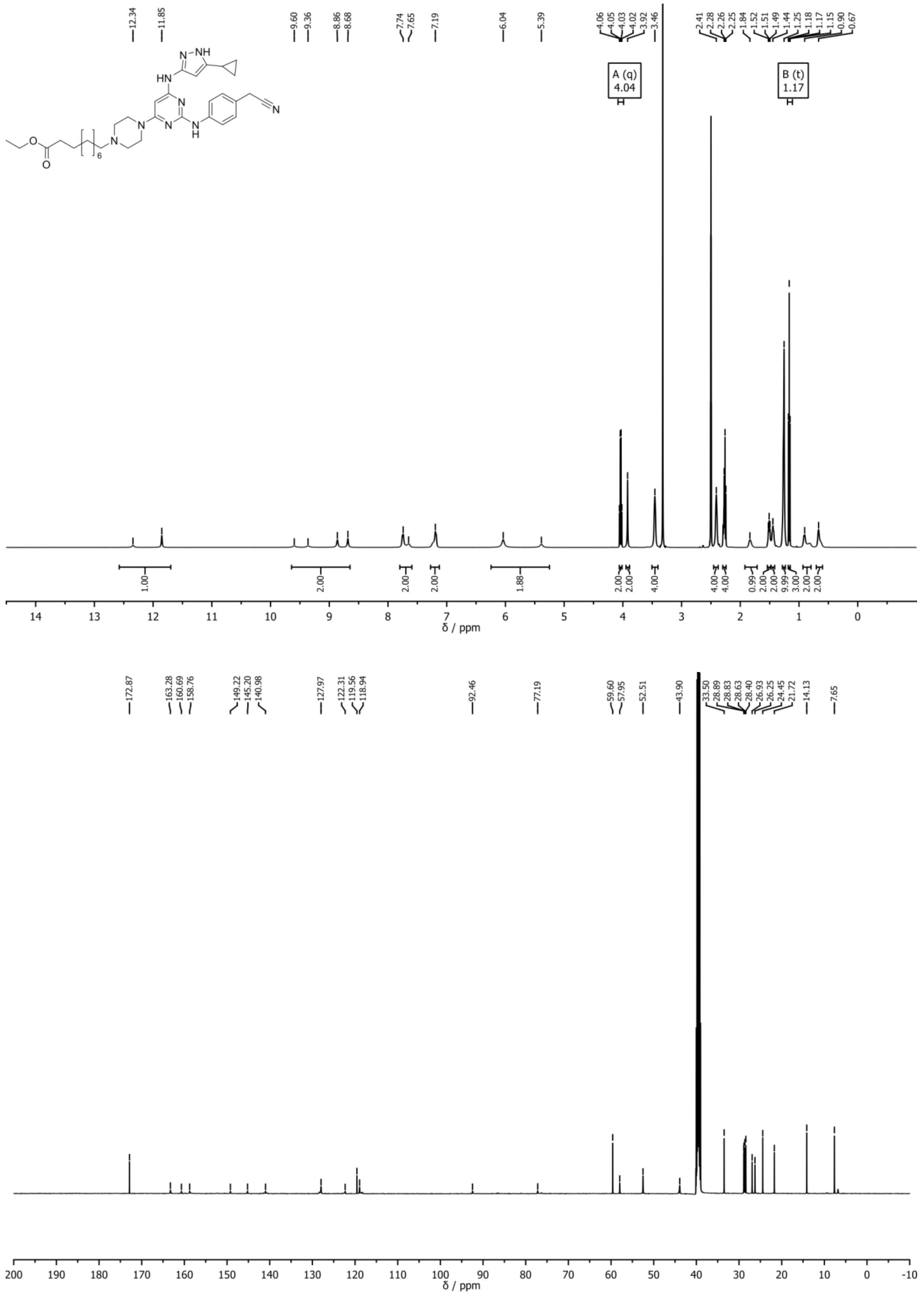

Figure S92: ^{1}H- (top) and ^{13}C-NMR (bottom) spectra (500 MHz and 126 MHz, 298 K, DMSO-d_6) and chemical structure of compound **S-33**.

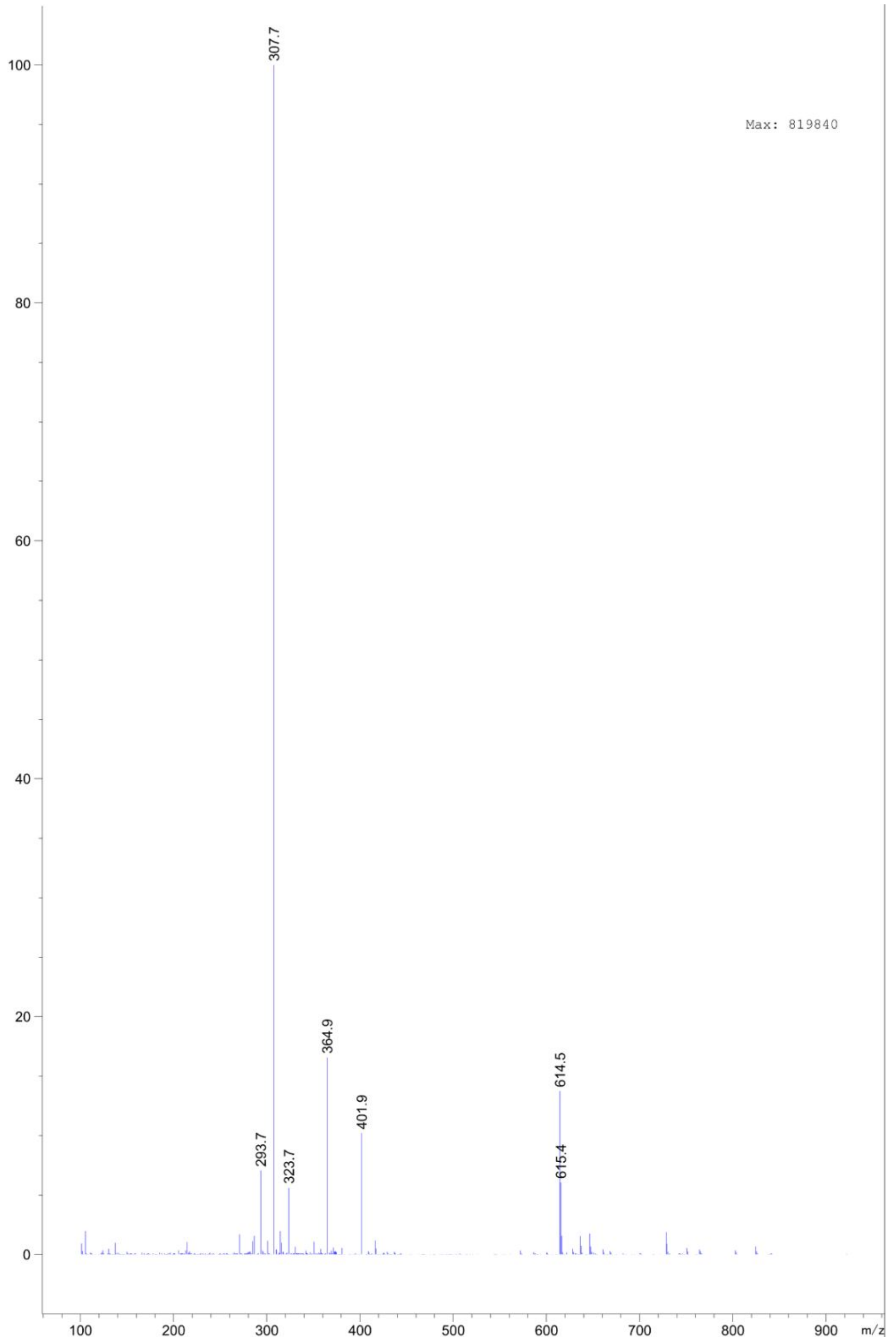

Figure S93: ESI-MS spectrum of compound **S-33** with $[M+H]^{+}_{calc.}$ = 614.39 m/z.

2-(4-((4-(4-(10-(4-(4-((2-aminoethyl)amino)-2-(1-(4-chlorophenyl)cyclohexyl)quinazolin-7-yl)piperazin-1-yl)-10-oxodecyl)piperazin-1-yl)-6-((5-cyclopropyl-1*H*-pyrazol-3-yl)amino)pyrimidin-2-yl)amino)phenyl)acetonitrile (TFA salt, 1:2) (D-1e)

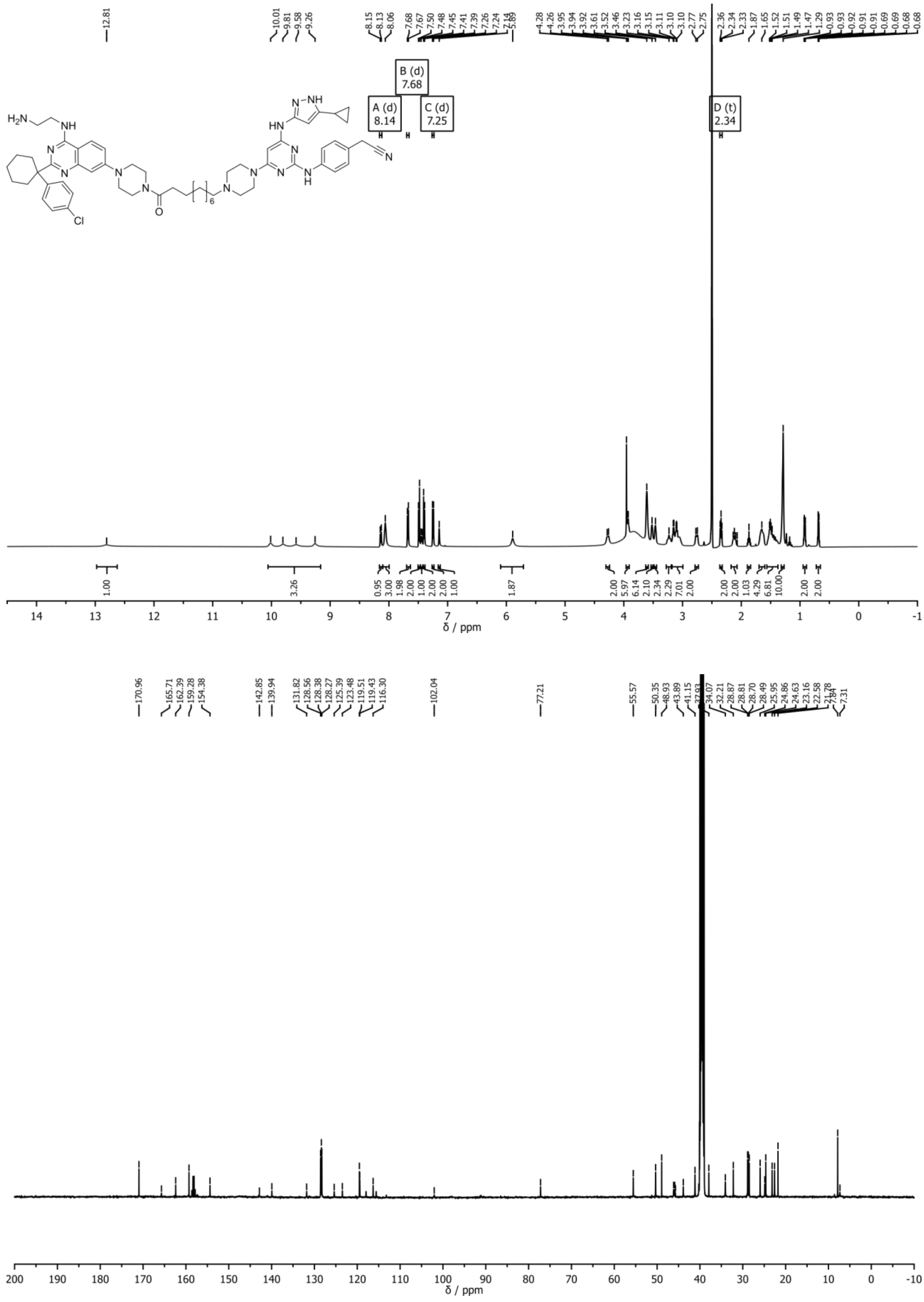

Figure S94: ^{1}H- (top) and ^{13}C-NMR (bottom) spectra (500 MHz and 126 MHz, 298 K, DMSO-d_6) and chemical structure of compound **D-1e**.

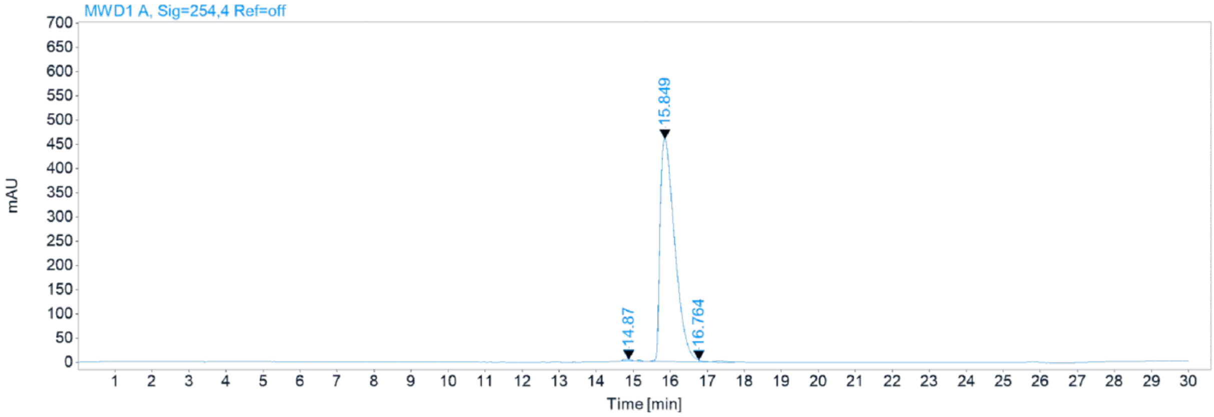

Signal: MWD1 A, Sig=254,4 Ref=off

RT [min]	Type	Width [min]	Area	Height	Area%	Name
14.870	MF	0.4322	91.7912	3.5393	0.7156	
15.849	MF	0.4569	12611.5195	460.0279	98.3250	
16.764	FM	0.4902	123.0524	4.1838	0.9594	
		Sum	12826.3631			

Figure S95: LC/MS spectra of purified compound **D-1e** at 254 nm wavelength.

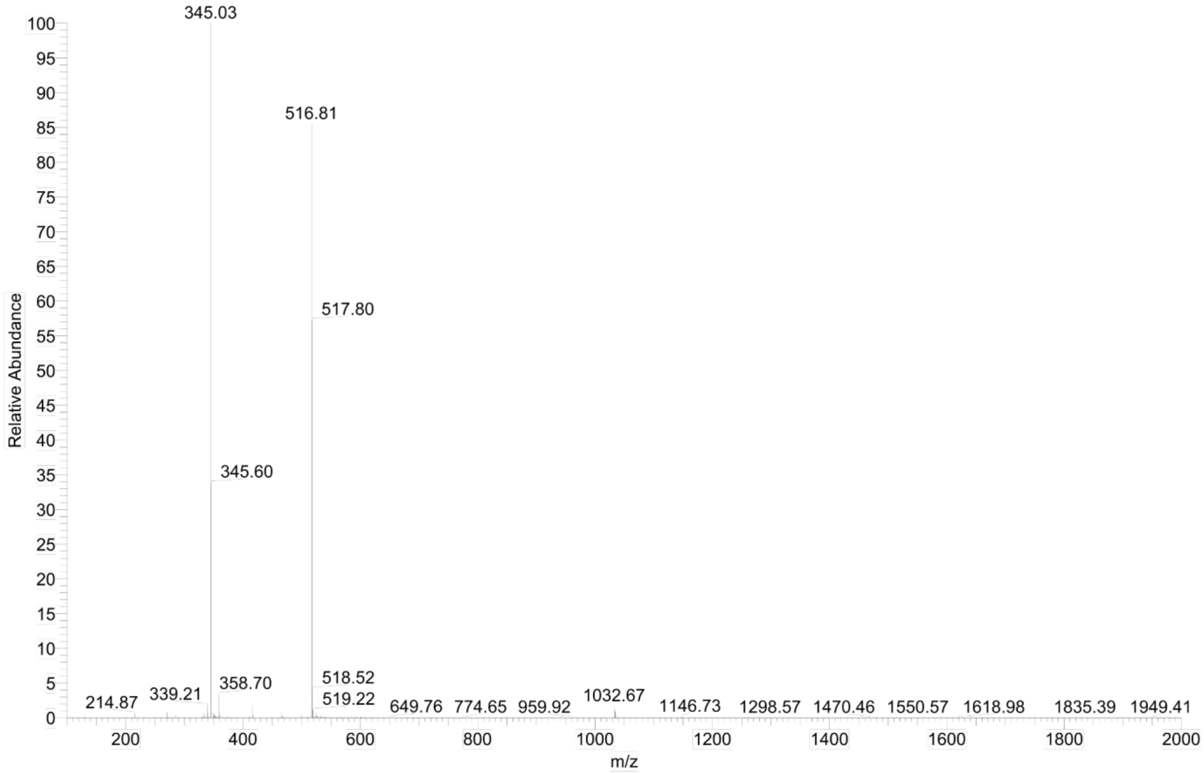

Figure S96: ESI-MS spectrum of compound **D-1e** with $[M+H]^+_{calc.}$ = 1032.59 m/z.

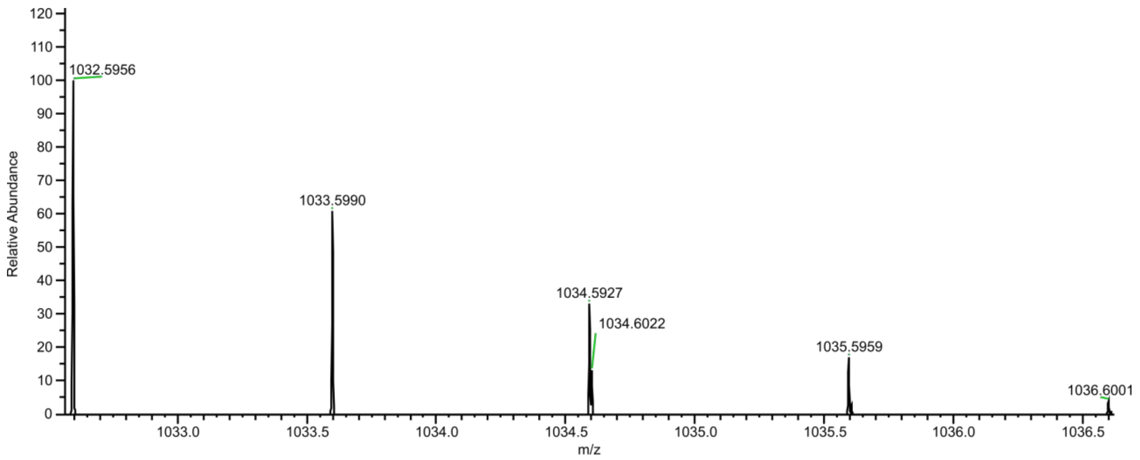

Figure S97: High-resolution mass spectrum of compound **D-1e** with $[M+H]^{+}_{calc.}$ = 1032.5962 m/z.

***Tert*-butyl 4-(4-(2-((4-(cyanomethyl)phenyl)amino)-6-((5-cyclopropyl-1*H*-pyrazol-3-yl)amino)pyrimidin-4-yl)piperazin-1-yl)-4-oxobutanoate (S-37)**

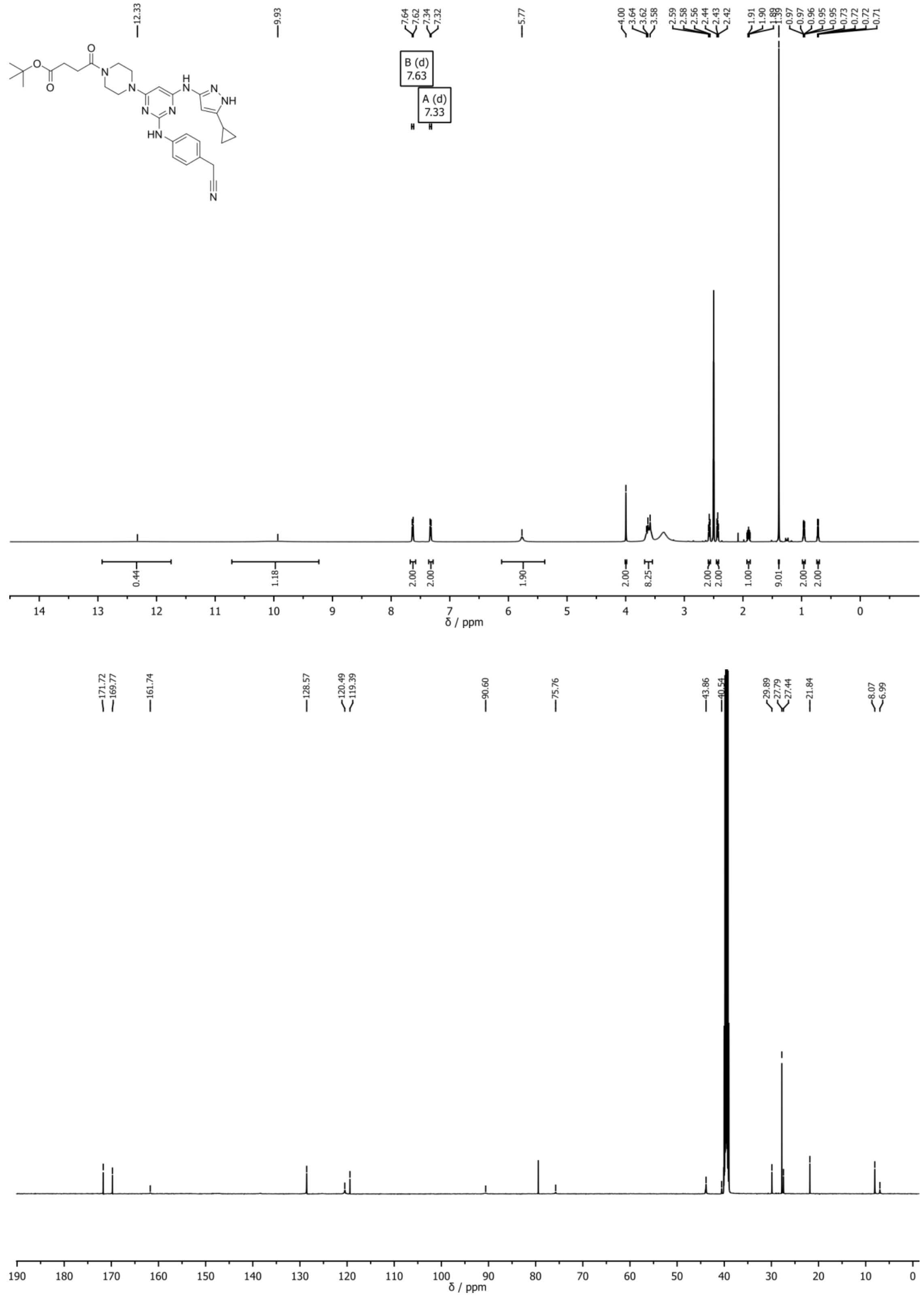

Figure S98: ^{1}H- (top) and ^{13}C-NMR (bottom) spectra (500 MHz and 126 MHz, 298 K, DMSO-d_6) and chemical structure of compound **S-37**.

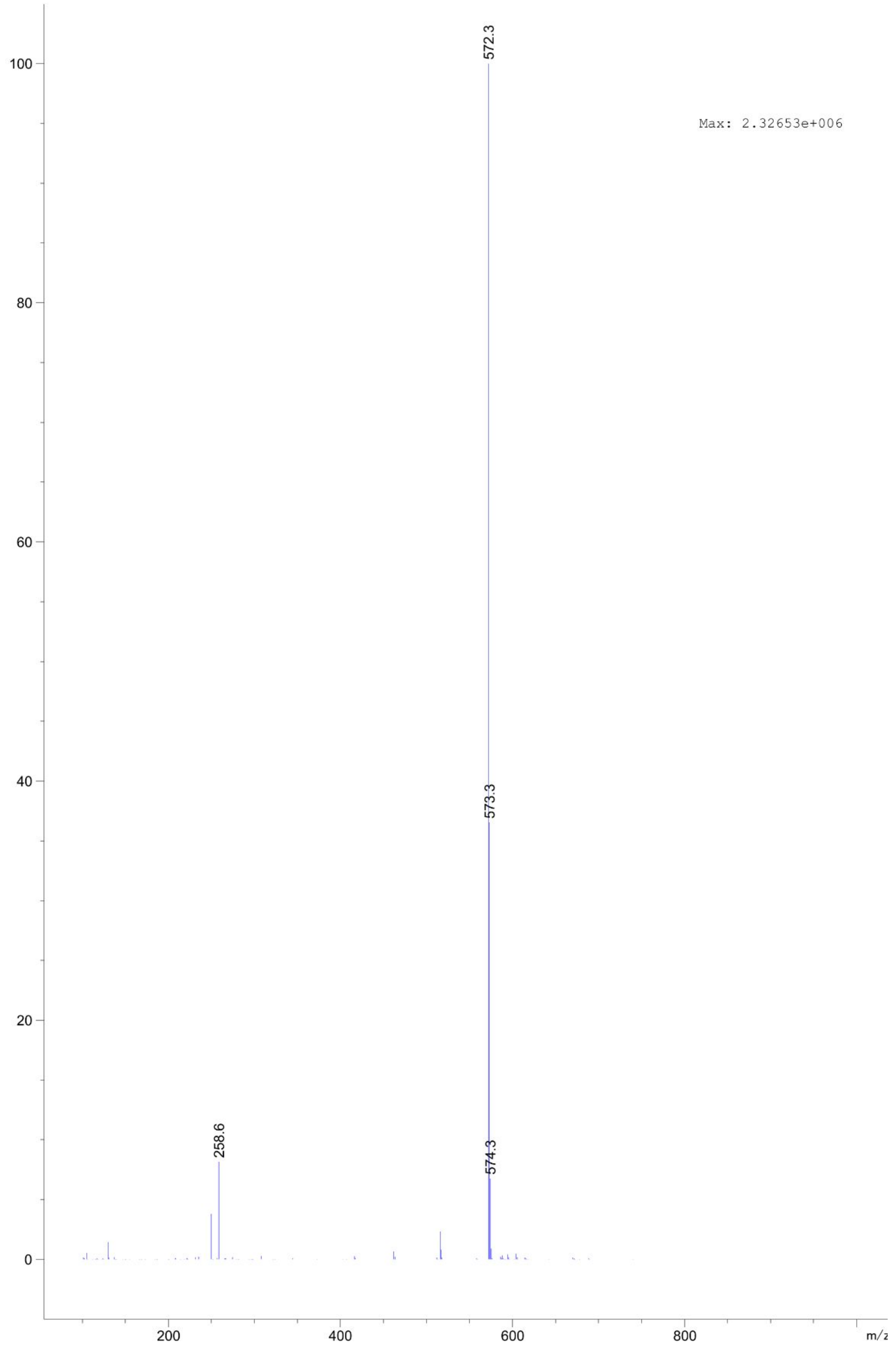

Figure S99: ESI-MS spectrum of compound **S-37** with $[M+H]^+_{calc.}$ = 572.30 m/z.

2-(4-((4-(4-(4-(4-(4-((2-aminoethyl)amino)-2-(1-(4-chlorophenyl)cyclohexyl)quinazolin-7-yl)piperazin-1-yl)-4-oxobutanoyl)piperazin-1-yl)-6-((5-cyclopropyl-1*H*-pyrazol-3-yl)amino)pyrimidin-2-yl)amino)phenyl)acetonitrile (TFA salt, 1:1) (D-1f)

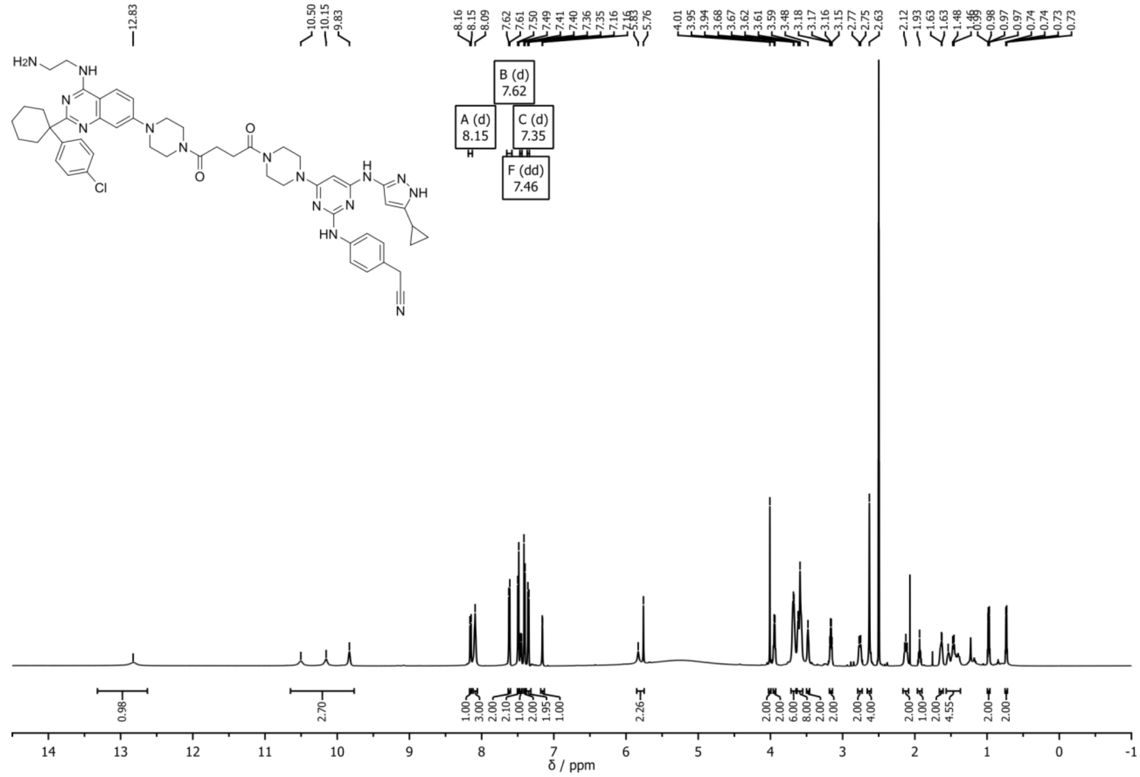

Figure S100: ^{1}H-NMR spectrum (600 MHz and 151 MHz, 298 K, DMSO-d_6) and chemical structure of compound **D-1f**.

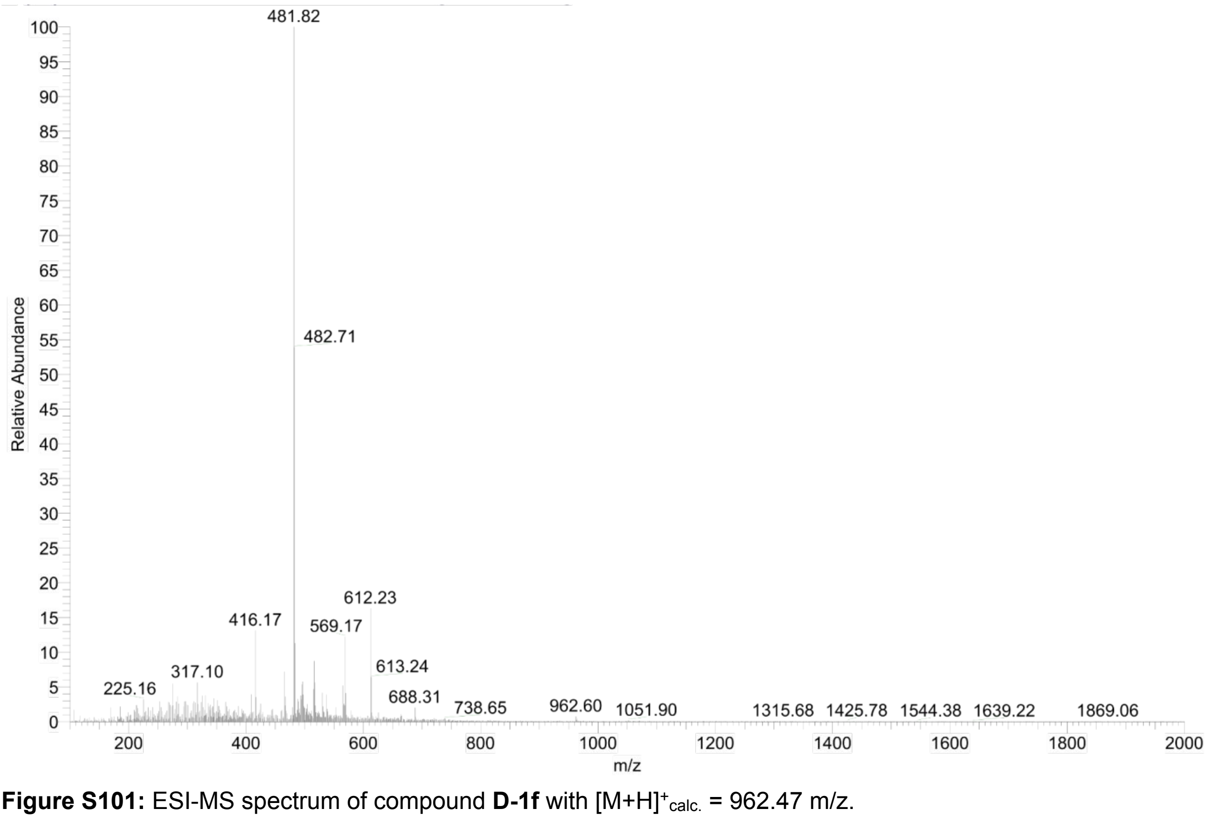

Figure S101: ESI-MS spectrum of compound **D-1f** with $[M+H]^+_{calc.}$ = 962.47 m/z.

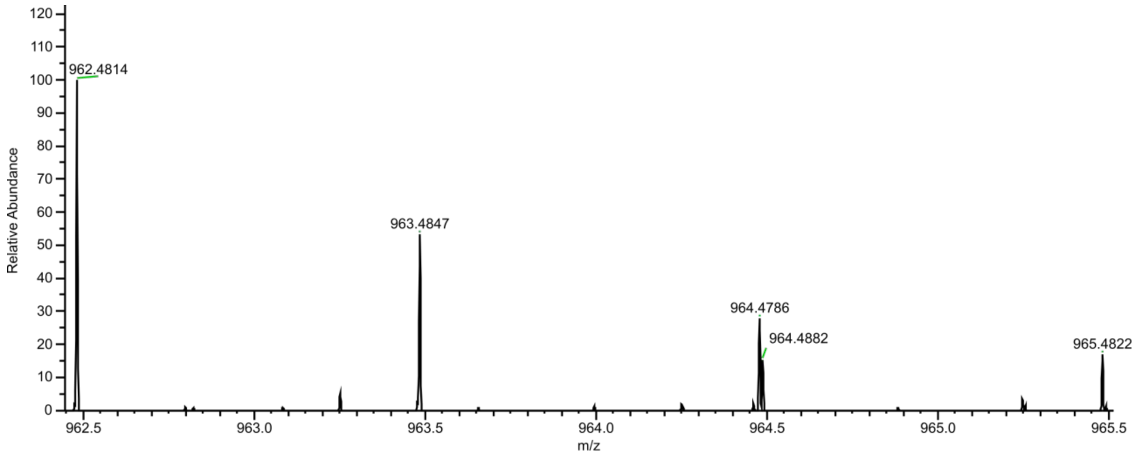

Figure S102: High-resolution mass spectrum of compound **D-1f** with $[M+H]^+_{calc.}$ = 962.4816 m/z.

7.2.2. CRBN-recruiting Promiscuous Kinase PROTACs based on Kinase Parent Inhibitor 1inh

***Tert*-butyl 3-(2-(2-((2-(2,6-dioxopiperidin-3-yl)-1,3-dioxoisoindolin-4-yl)oxy)ethoxy)-ethoxy)propanoate (S-38)**

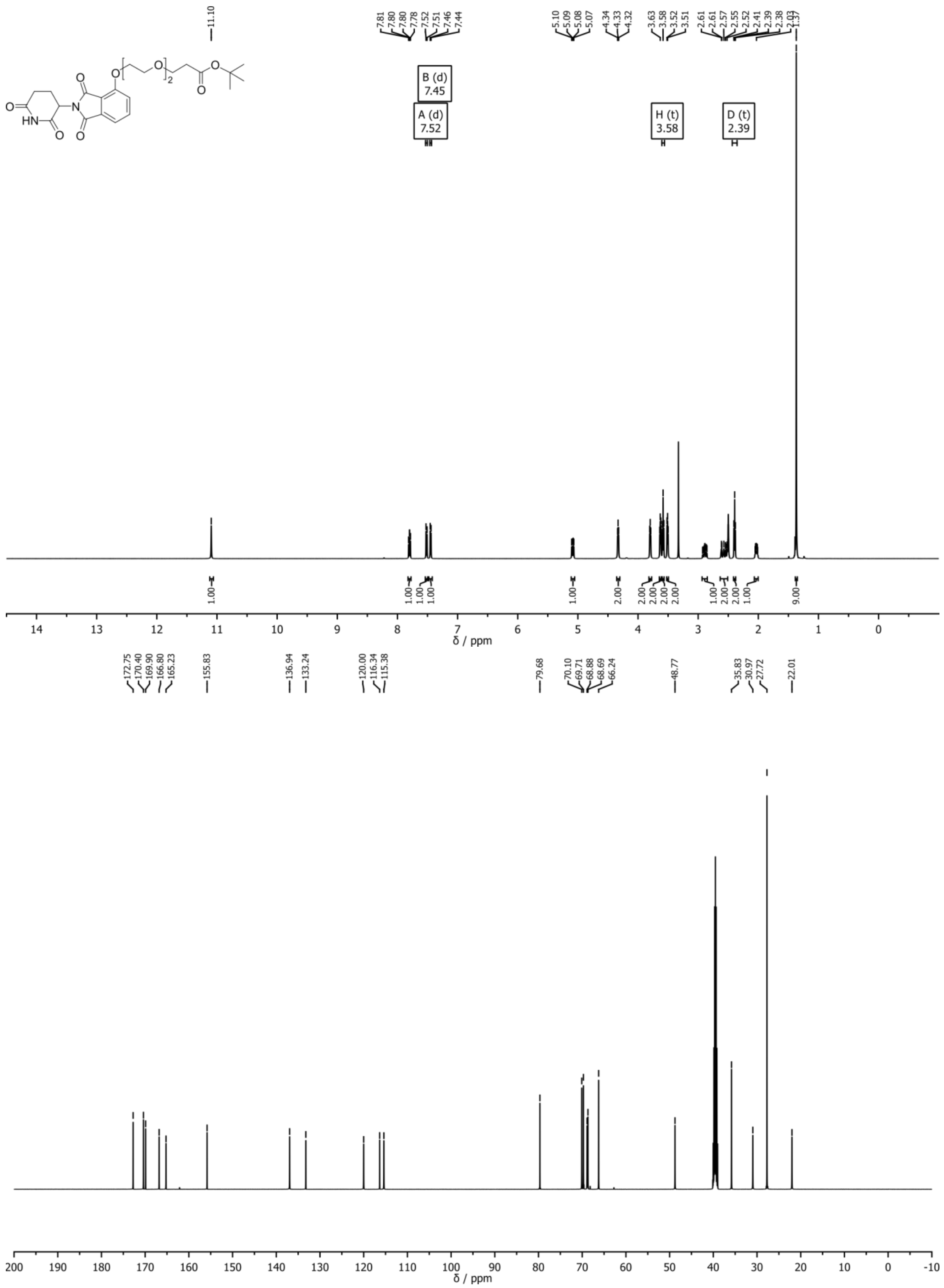

Figure S103: ^{1}H- (top) and ^{13}C-NMR (bottom) spectra (500 MHz and 126 MHz, 298 K, DMSO-d_6) and chemical structure of compound **S-38**.

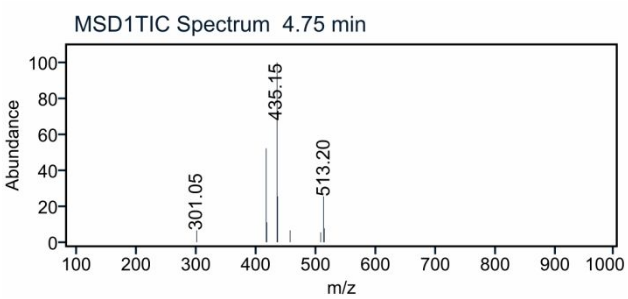

Figure S104: ESI-MS spectrum of compound **S-38** with $[M+H]^{+}_{calc.}$ = 491.20 m/z.

2-(4-((4-((5-Cyclopropyl-1H-pyrazol-3-yl)amino)-6-(4-(3-(2-(2-((2-(2,6-dioxopiperidin-3-yl)-1,3-dioxoisoindolin-4-yl)oxy)ethoxy)ethoxy)propanoyl)piperazin-1-yl)pyrimidin-2-yl)amino)phenyl)acetonitrile (C-1a)

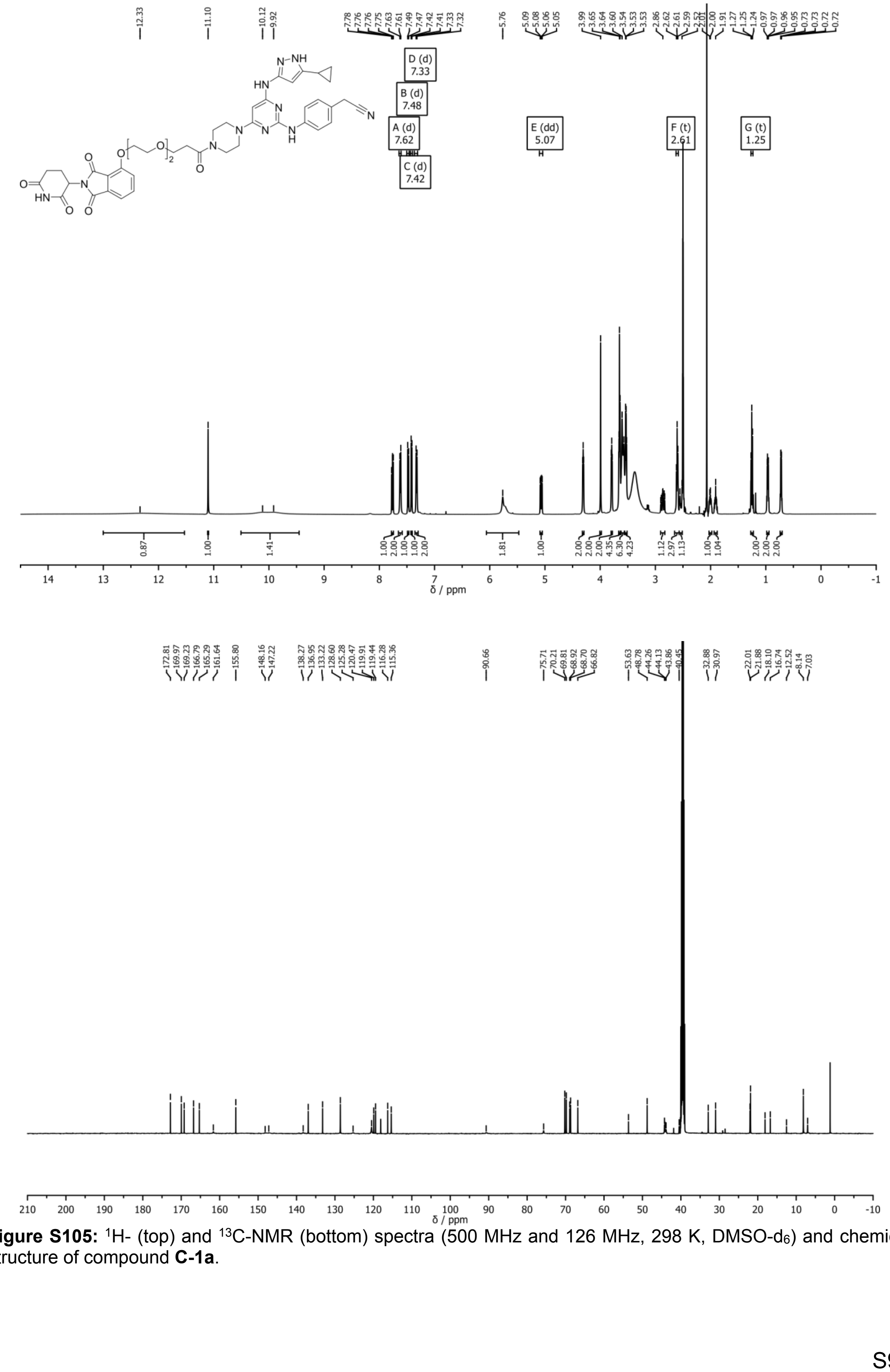

Figure S105: ^{1}H- (top) and ^{13}C-NMR (bottom) spectra (500 MHz and 126 MHz, 298 K, DMSO-d_6) and chemical structure of compound **C-1a**.

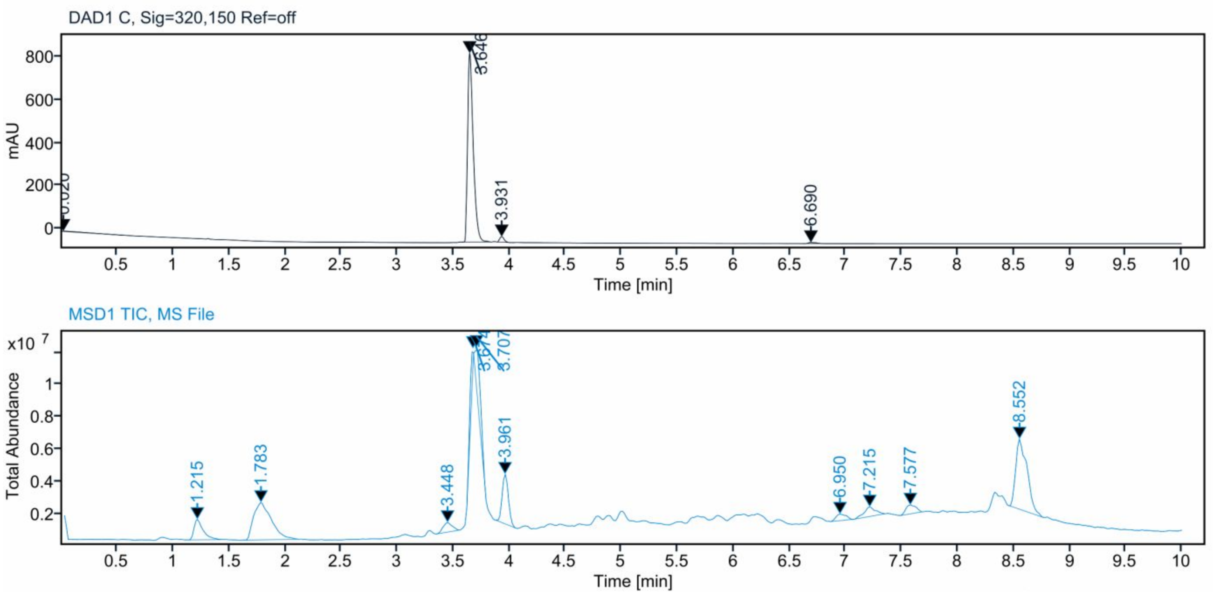

Sample Purity

Signal Description DAD1 C, Sig=320,150 Ref=off

Sample Name	Name	RT	Width	Area	Area%	Height
NM257_thicker		0.020	0.050	31.1086	0.98	1.4597
NM257_thicker		3.646	0.052	3047.2192	95.91	873.5488
NM257_thicker		3.931	0.043	70.9451	2.23	25.4071
NM257_thicker		6.690	0.088	27.9802	0.88	5.0790

Max Area% 95.907

UV Signal Purity>95% Pass

Figure S106: LC/MS spectra of purified compound **C-1a** at 320 nm.

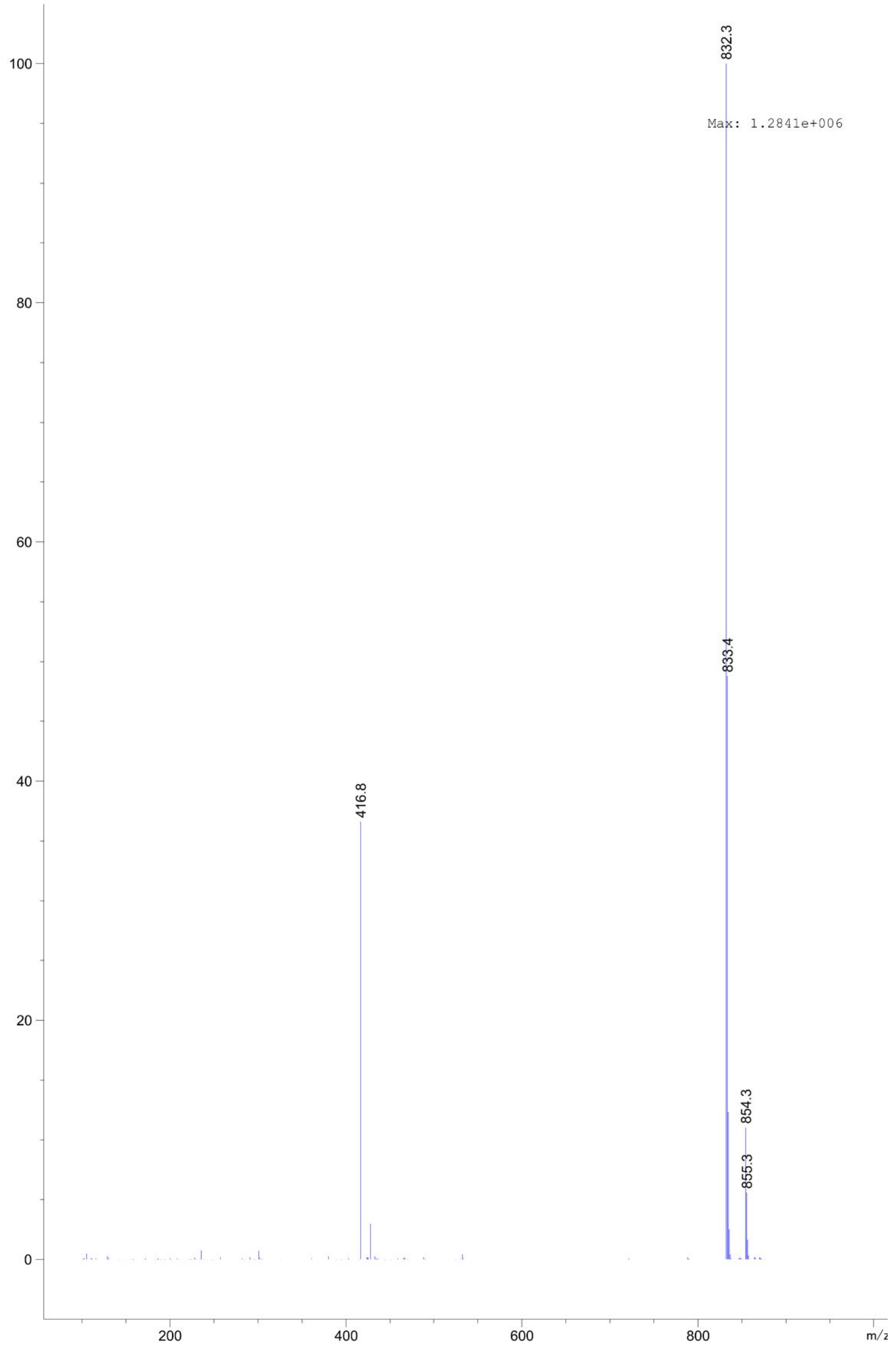

Figure S107: ESI-MS spectrum of compound **C-1a** with $[M+H]^{+}_{calc.}$ = 832.35 m/z.

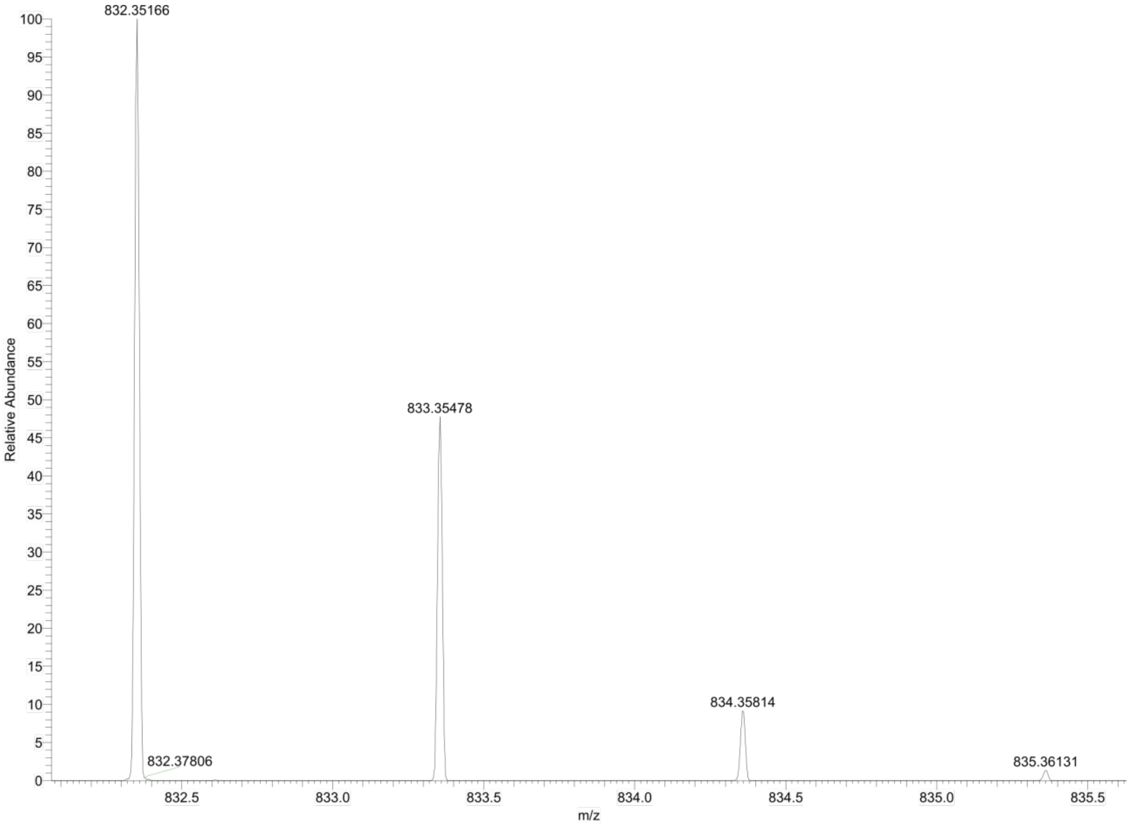

Figure S108: High-resolution mass spectrum of compound **C-1a** with $[M+H]^{+}_{calc.}$ = 832.35312 m/z.

***Tert*-butyl 3-(2-(2-(2-((2-(2,6-dioxopiperidin-3-yl)-1,3-dioxoisoindolin-4-yl)oxy)-acetamido)ethoxy)ethoxy)propanoate (S-40)**

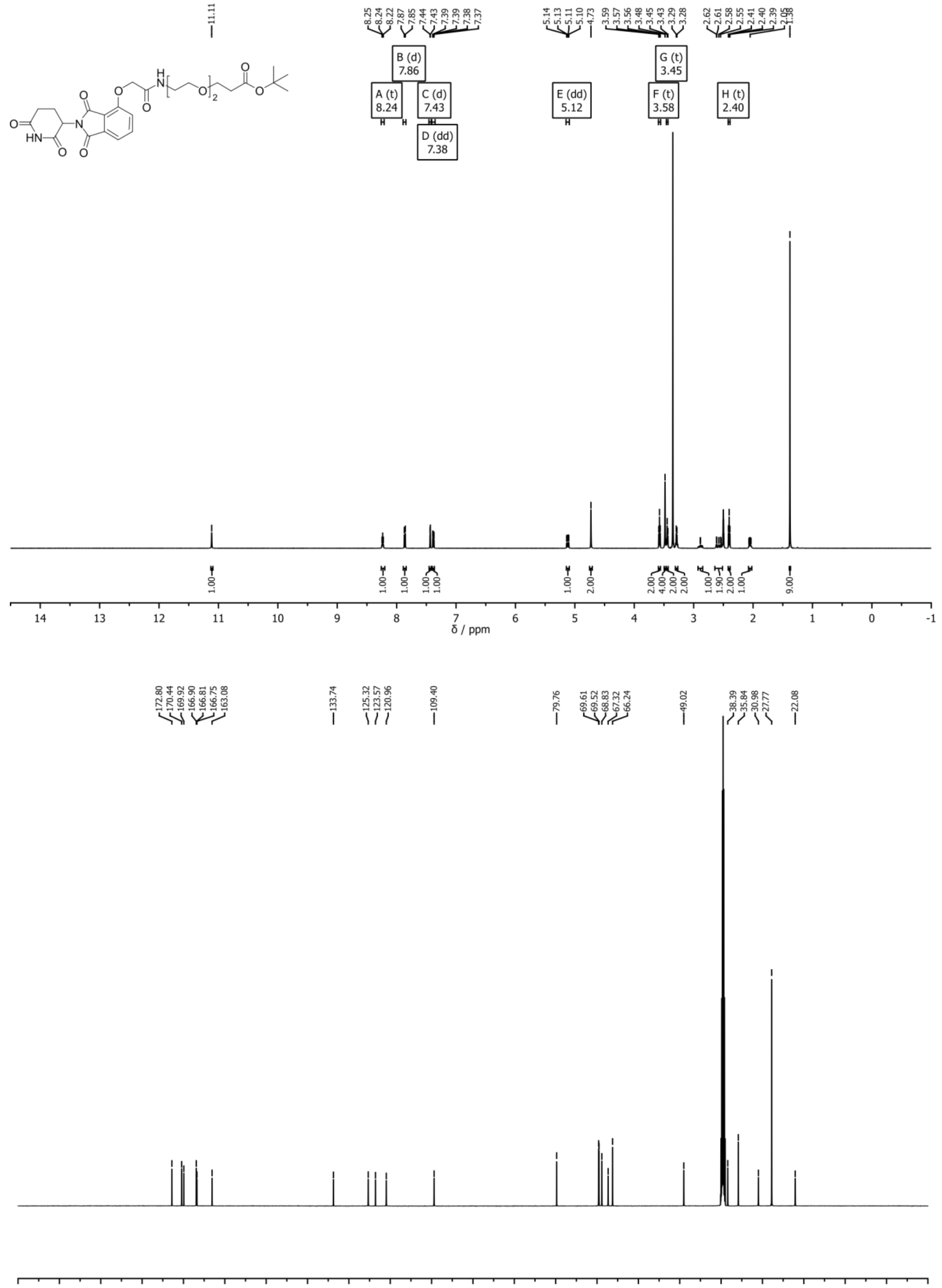

Figure S109: ^{1}H- (top) and ^{13}C-NMR (bottom) spectra (500 MHz and 126 MHz, 298 K, DMSO-d_6) and chemical structure of compound **S-40**.

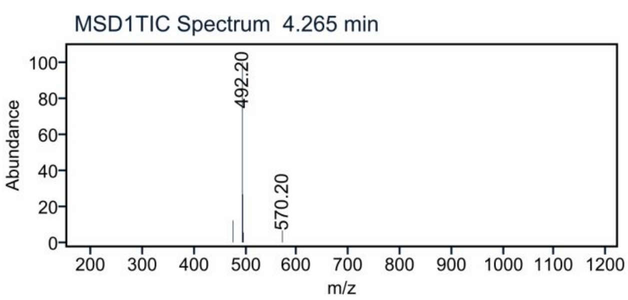

Figure S110: ESI-MS spectrum of compound **S-40** with $[M+Na]^+_{calc.}$ = 570.20 m/z.

_N_-(2-(2-(3-(4-(2-((4-(cyanomethyl)phenyl)amino)-6-((5-cyclopropyl-1_H_-pyrazol-3-yl)amino)pyrimidin-4-yl)piperazin-1-yl)-3-oxopropoxy)ethoxy)ethyl)-2-((2-(2,6-dioxopiperidin-3-yl)-1,3-dioxoisoindolin-4-yl)oxy)acetamide (C-1b)

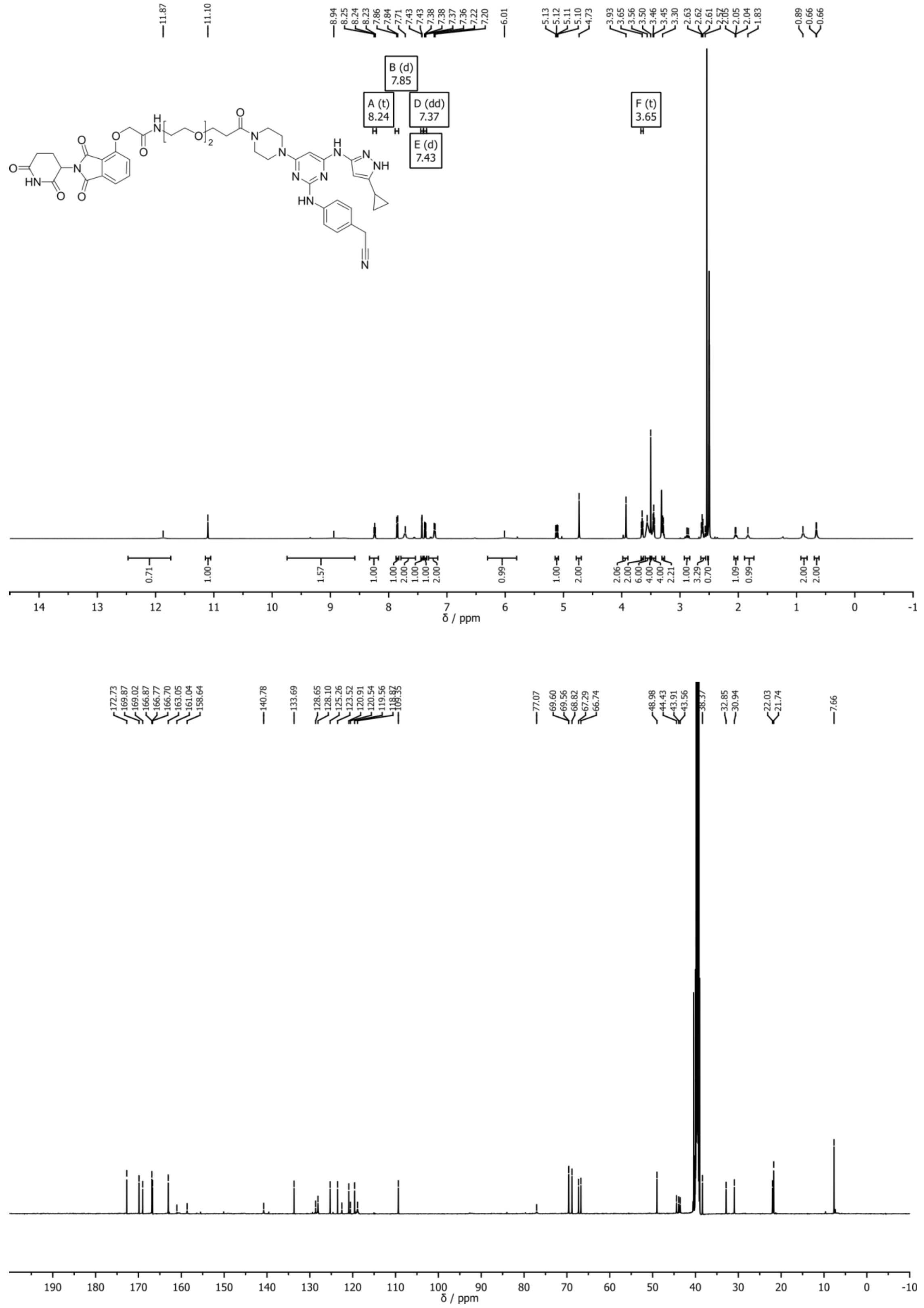

Figure S111: ^{1}H- (top) and ^{13}C-NMR (bottom) spectra (500 MHz and 126 MHz, 298 K, DMSO-d6) and chemical structure of compound **C-1b**.

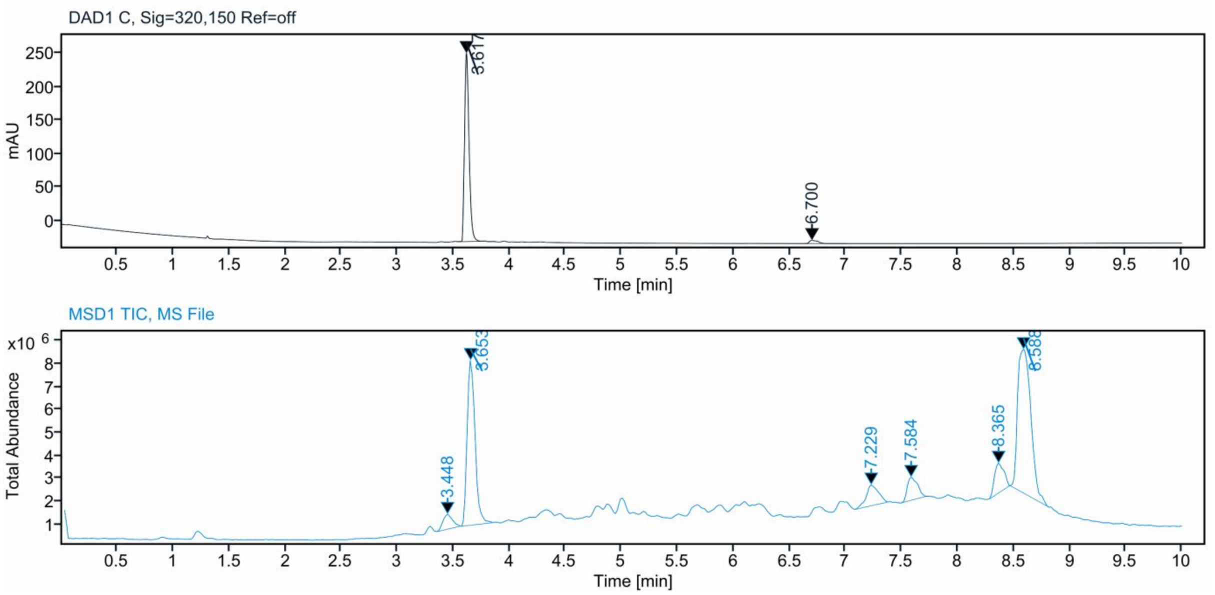

Sample Purity

Signal Description DAD1 C, Sig=320,150 Ref=off

Sample Name	Name	RT	Width	Area	Area%	Height
NM27_thicker		3.617	0.042	783.7585	96.59	281.0304
NM27_thicker		6.700	0.088	27.6382	3.41	4.9896

Max Area% 96.594

UV Signal Purity>95% Pass

Figure S112: LC/MS spectra of purified compound **C-1b** at 320 nm wavelength.

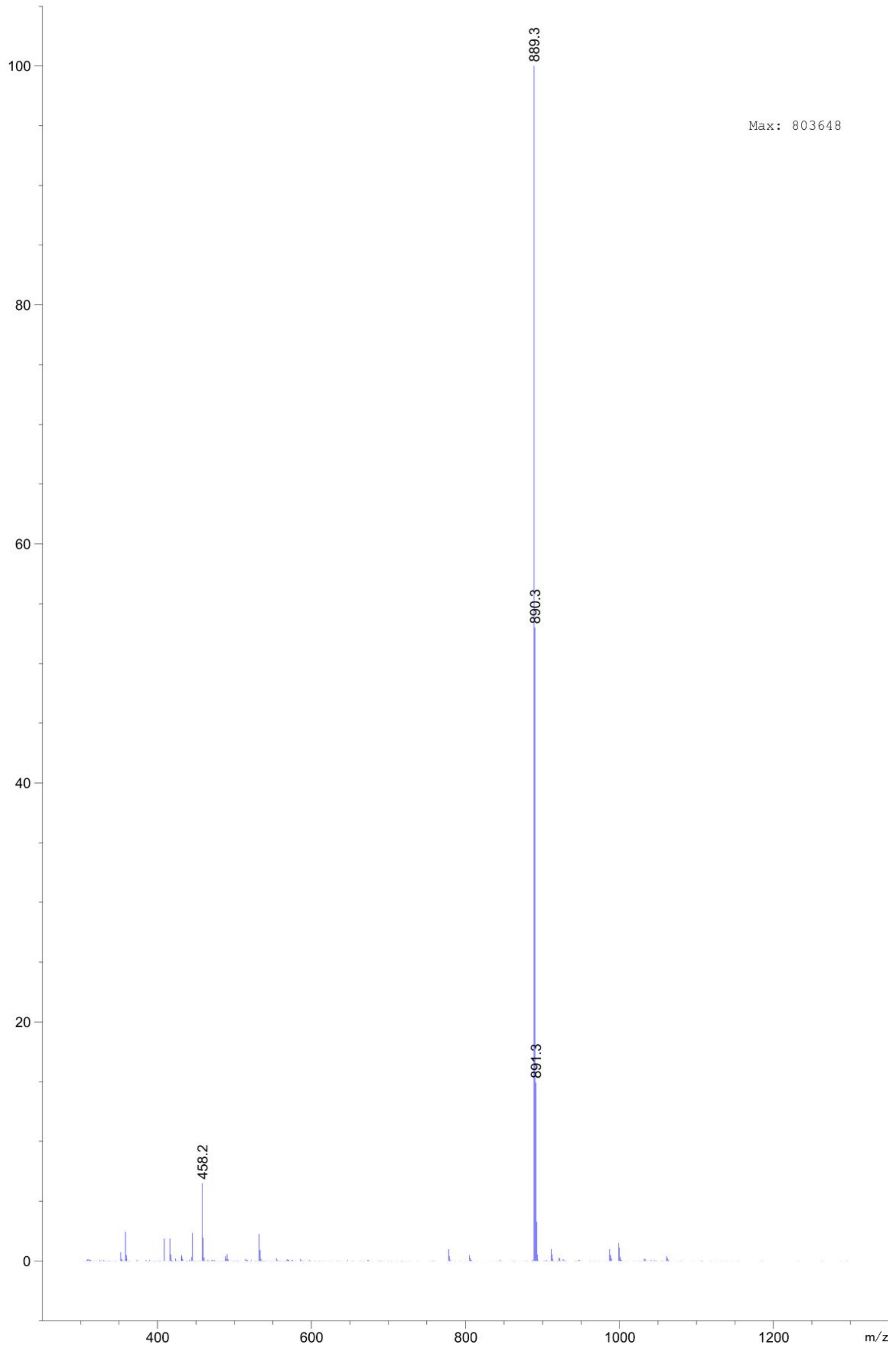

Figure S113: ESI-MS spectrum of compound **C-1b** with $[M+H]^+_{calc.}$ = 889.37 m/z.

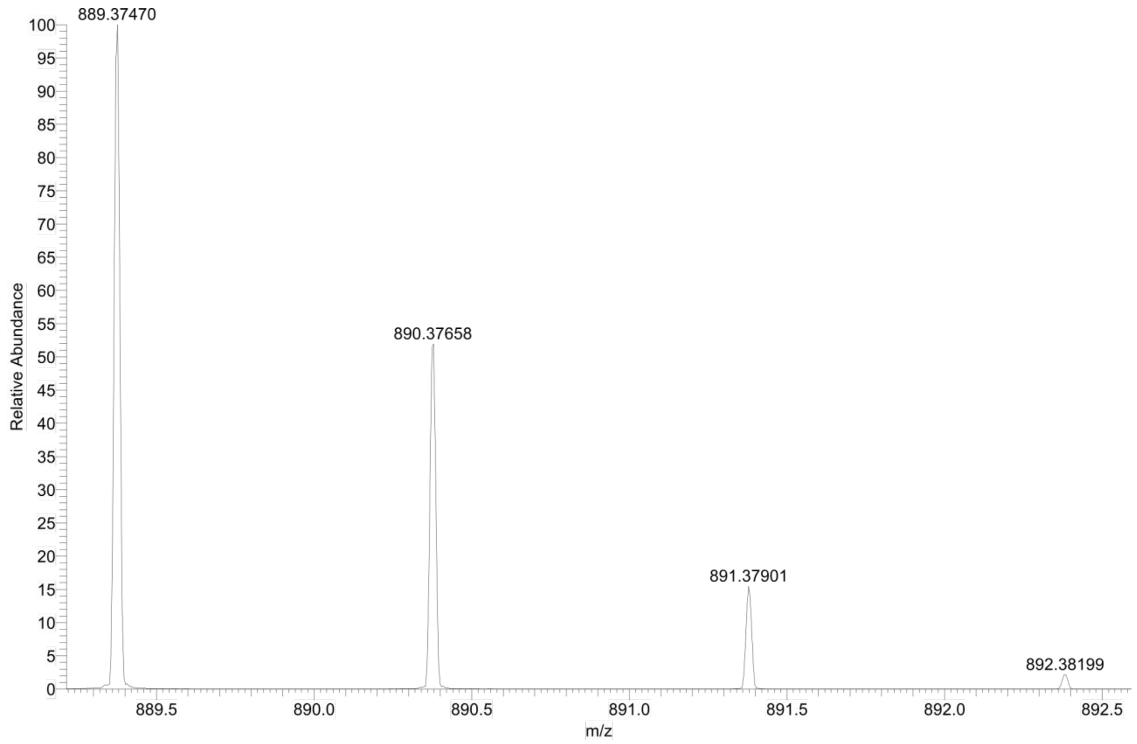

Figure S114: High-resolution mass spectrum of compound **C-1b** with $[M+H]^+_{calc.}$ = 889.37400 m/z.

***Tert*-butyl (2-(2-(2-(2-(4-(2-((4-(cyanomethyl)phenyl)amino)-6-((5-cyclopropyl-1H-pyrazol-3-yl)amino)pyrimidin-4-yl)piperazin-1-yl)ethoxy)ethoxy)ethoxy)ethyl)carbamate (S-42)**

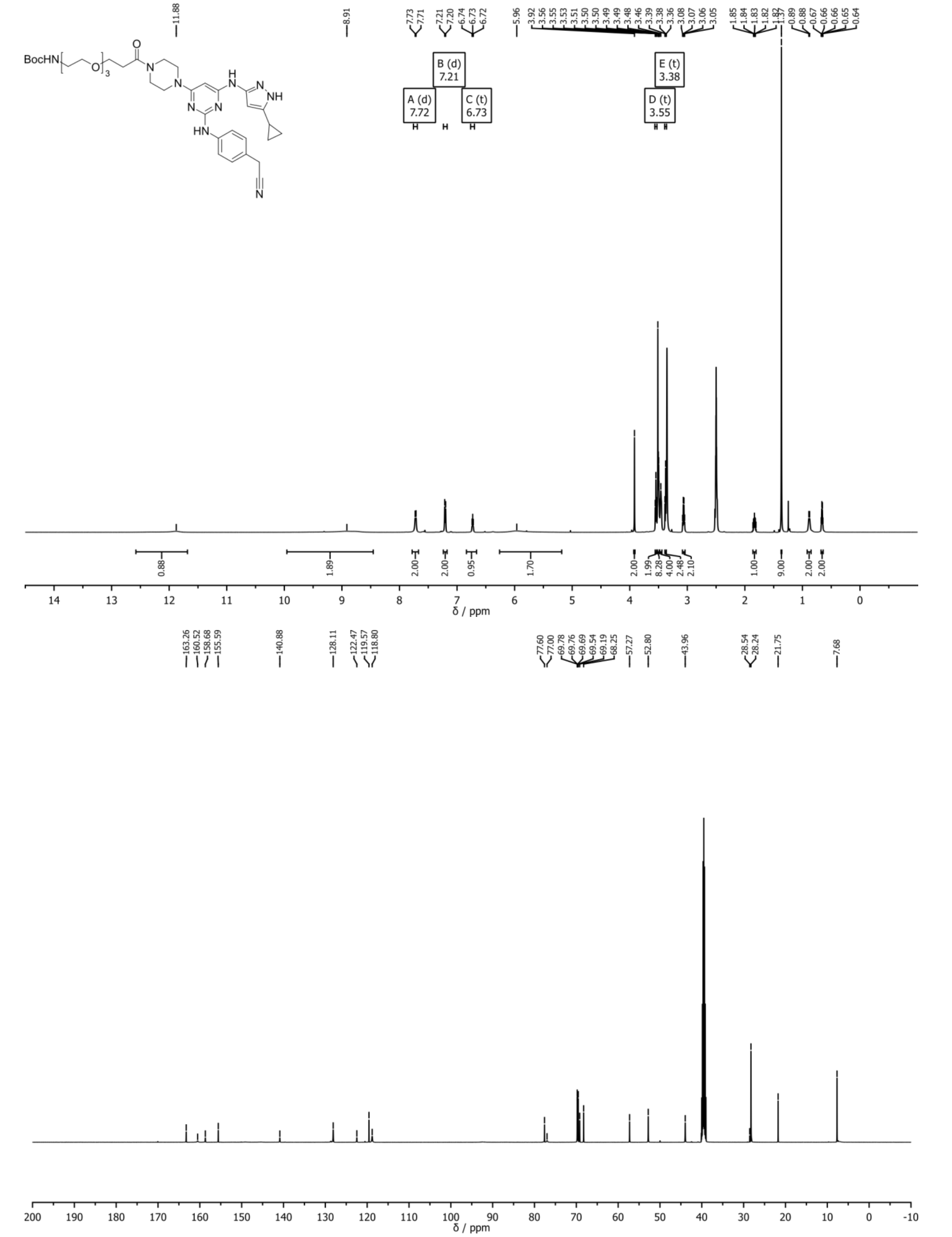

Figure S115: ^{1}H- (top) and ^{13}C-NMR (bottom) spectra (500 MHz and 126 MHz, 298 K, DMSO-d_6) and chemical structure of compound **S-42**.

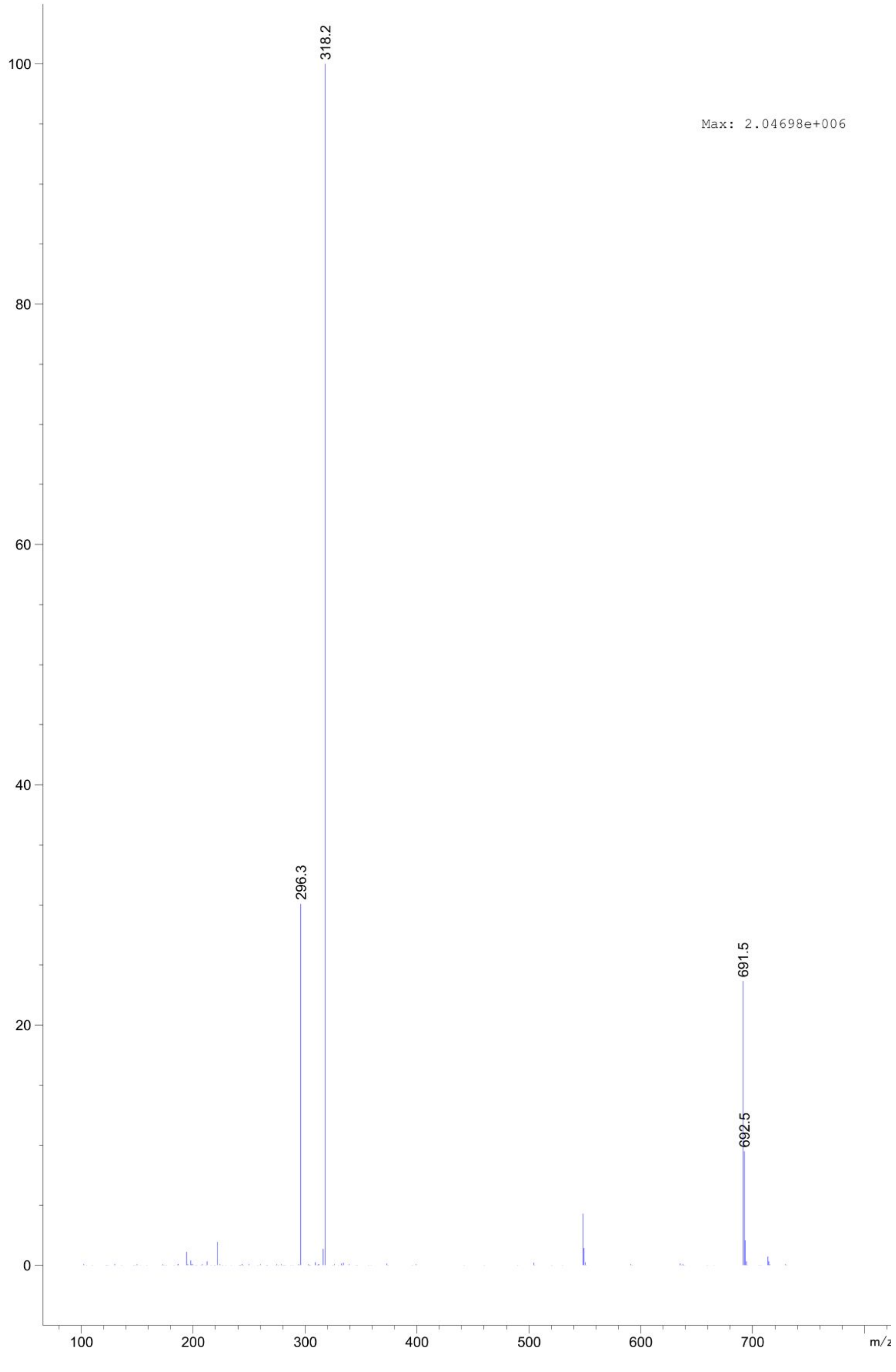

Figure S116: ESI-MS spectrum of compound **S-42** with $[M+H]^{+}_{calc.}$ = 691.40 m/z.

N-(2-(2-(2-(2-(4-(2-((4-(Cyanomethyl)phenyl)amino)-6-((5-cyclopropyl-1H-pyrazol-3-yl)-amino)pyrimidin-4-yl)piperazin-1-yl)ethoxy)ethoxy)ethoxy)ethyl)-2-((2-(2,6-dioxo-piperidin-3-yl)-1,3-dioxoisoindolin-4-yl)oxy)acetamide (C-1c)

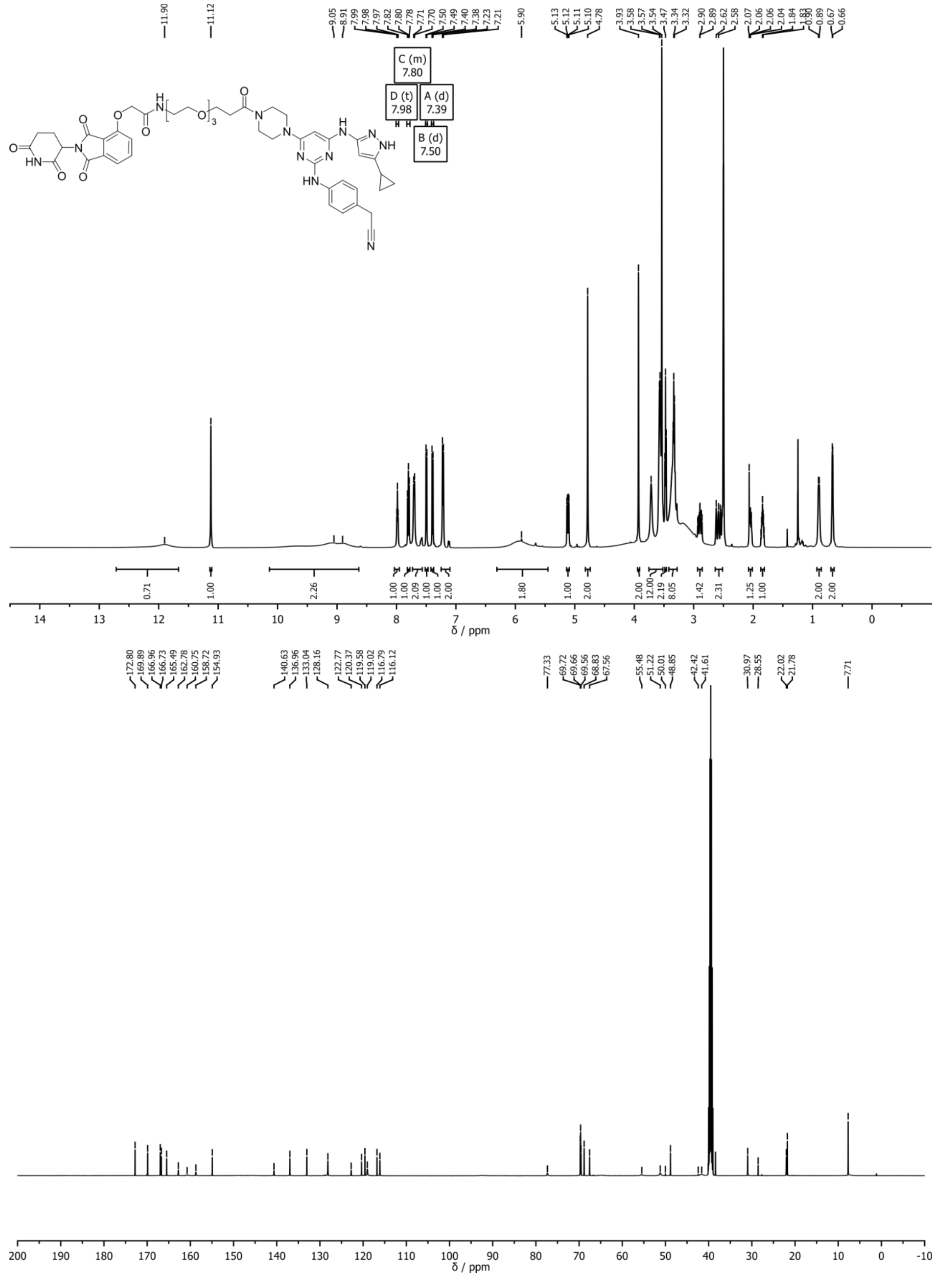

Figure S117: ^{1}H- (top) and ^{13}C-NMR (bottom) spectra (500 MHz and 126 MHz, 298 K, DMSO-d_6) and chemical structure of compound **C-1c**.

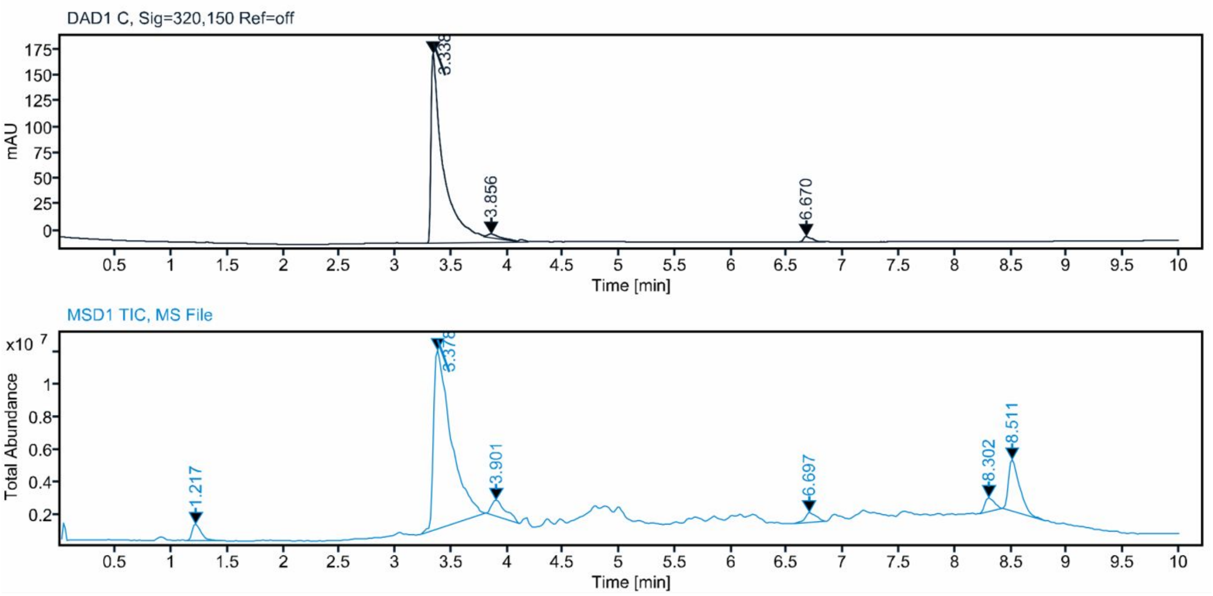

Sample Purity

Signal Description DAD1 C, Sig=320,150 Ref=off

Sample Name	Name	RT	Width	Area	Area%	Height
NM249_thin		3.338	0.087	1479.6694	96.09	183.4713
NM249_thin		3.856	0.094	31.6381	2.05	3.7701
NM249_thin		6.670	0.089	28.5013	1.85	5.3029

Max Area% 96.094

UV Signal Purity>95% Pass

Figure S118: LC/MS spectra of purified compound **C-1c** at 320 nm wavelength.

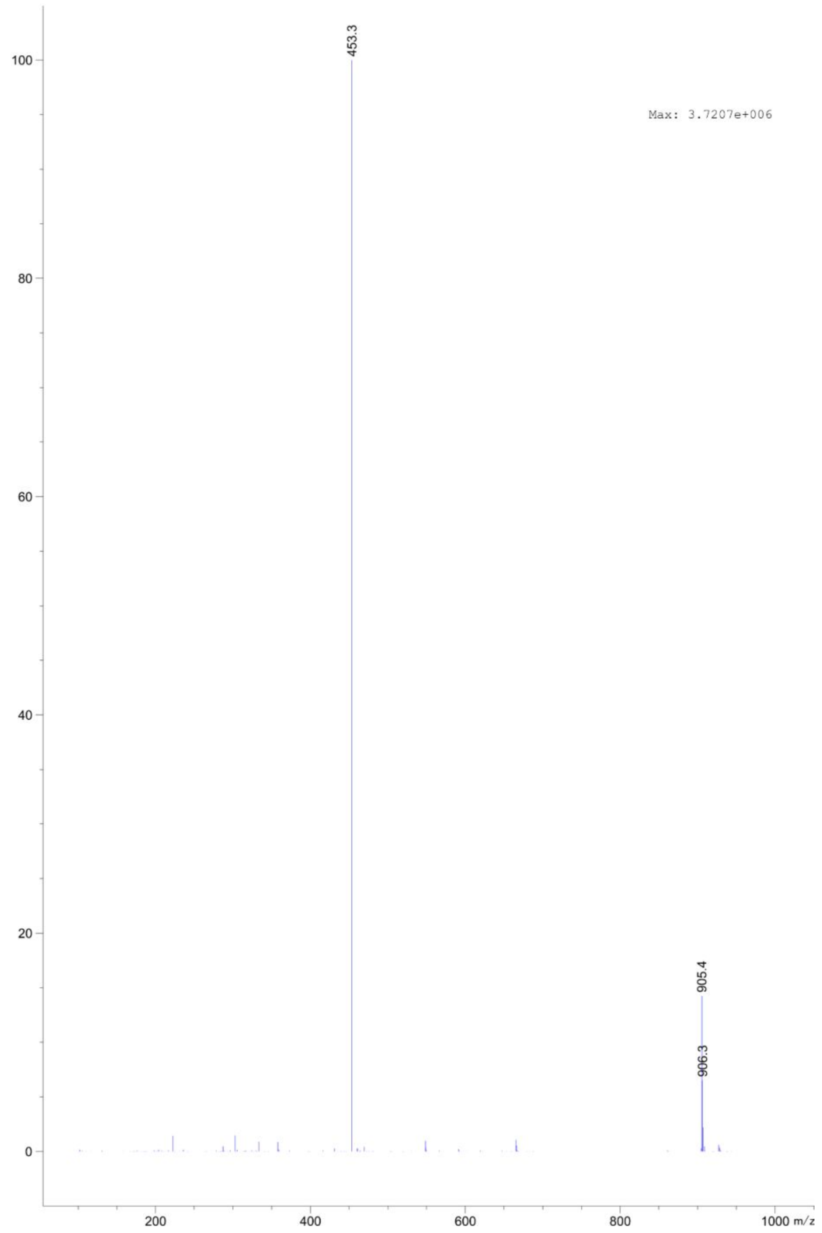

Figure S119: ESI-MS spectrum of compound **C-1c** with $[M+H]^+_{calc.}$ = 905.40 m/z.

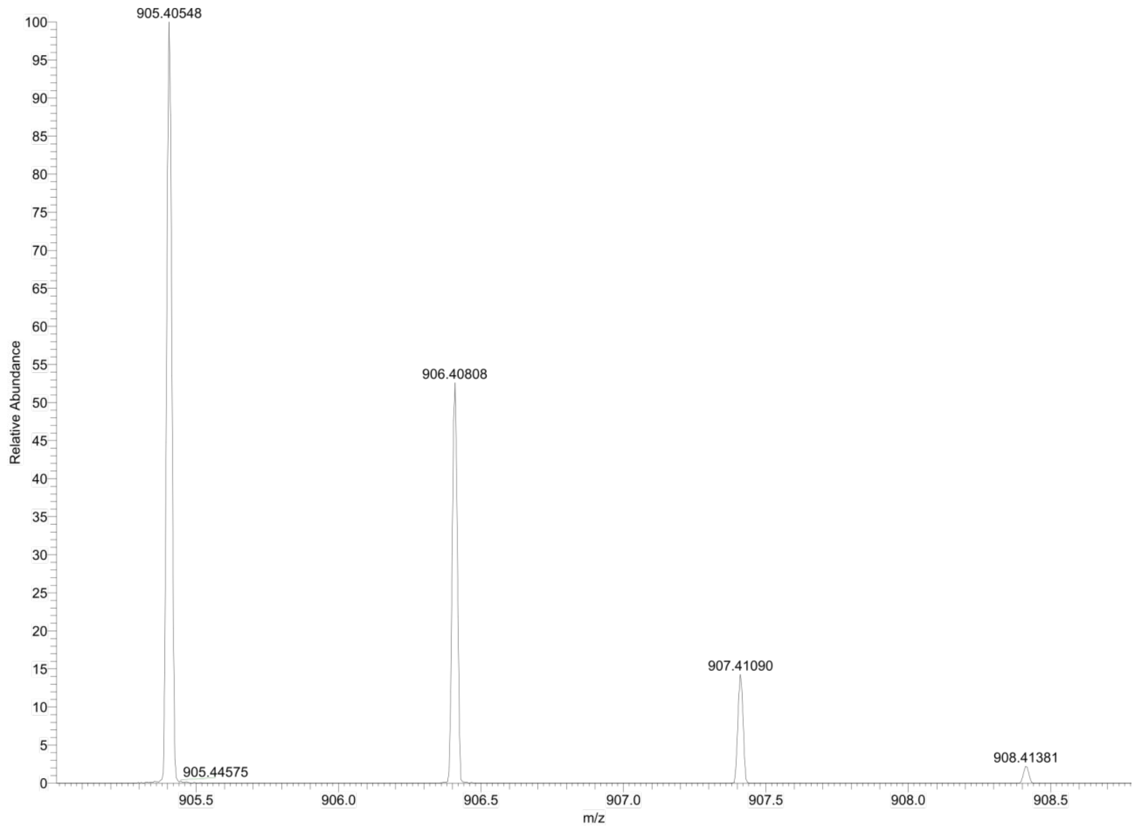

Figure S120: High-resolution mass spectrum of compound **C-1c** with $[M+H]^+_{calc.}$ = 905.40585 m/z.

***Tert*-butyl (7-(4-(2-((4-(cyanomethyl)phenyl)amino)-6-((5-cyclopropyl-1H-pyrazol-3-yl)amino)pyrimidin-4-yl)piperazin-1-yl)-7-oxoheptyl)carbamate (S-44)**

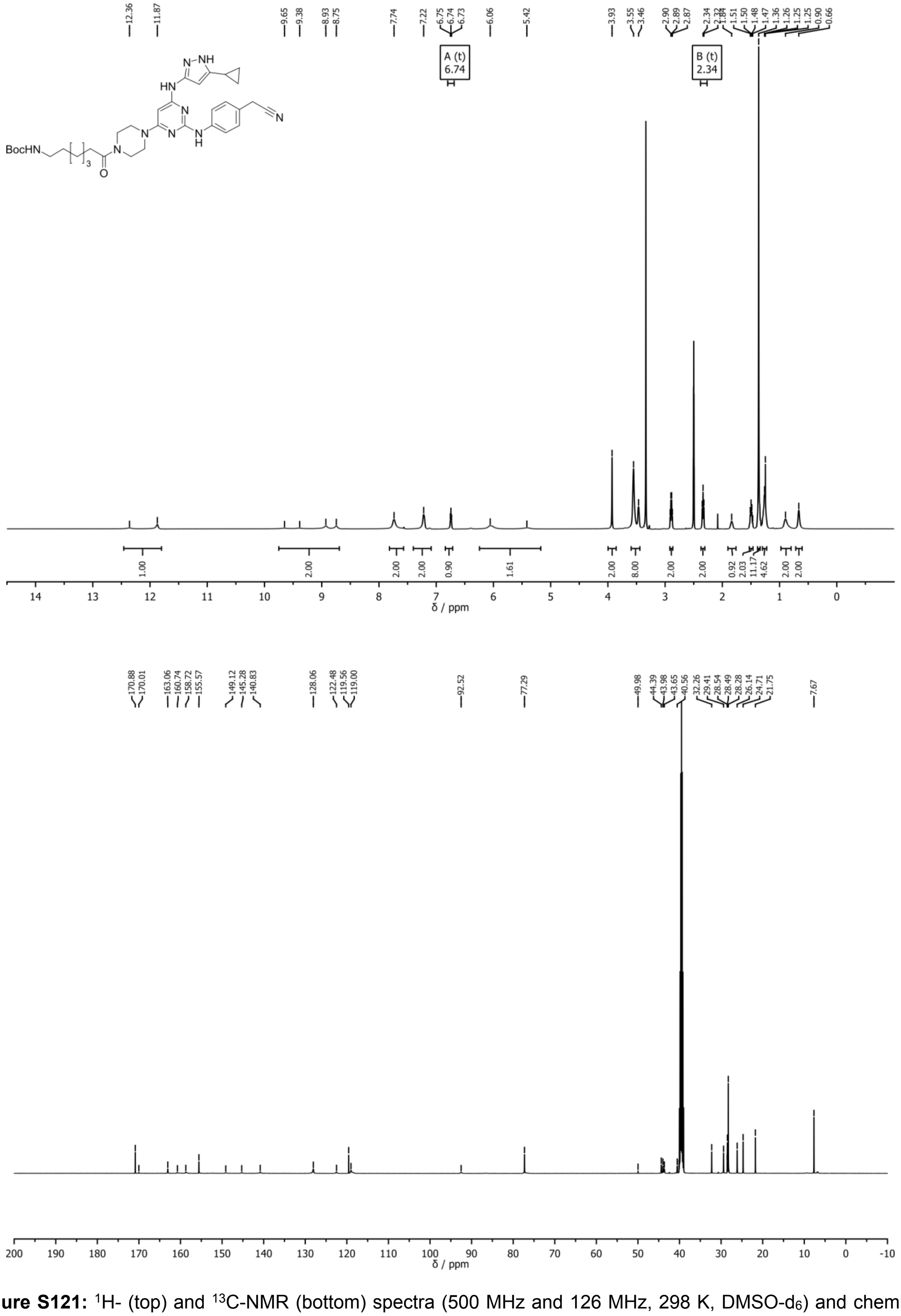

Figure S121: ^{1}H- (top) and ^{13}C-NMR (bottom) spectra (500 MHz and 126 MHz, 298 K, DMSO-d_6) and chemical structure of compound **S-44**.

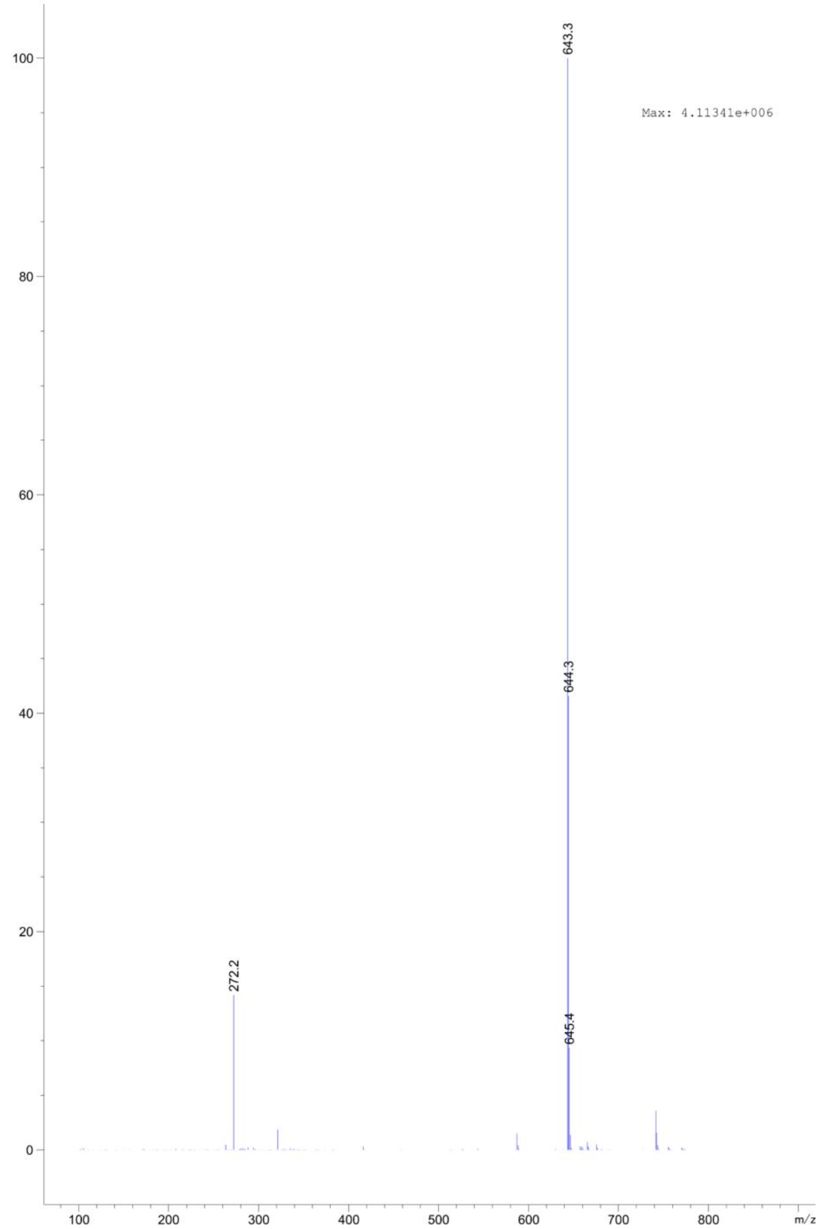

Figure S122: ESI-MS spectrum of compound **S-44** with $[M+H]^{+}_{calc.}$ = 643.38 m/z.

N-(7-(4-(2-((4-(Cyanomethyl)phenyl)amino)-6-((5-cyclopropyl-1H-pyrazol-3-yl)amino)-pyrimidin-4-yl)piperazin-1-yl)-7-oxoheptyl)-2-((2-(2,6-dioxopiperidin-3-yl)-1,3-dioxo-isoindolin-4-yl)oxy)acetamide (C-1d)

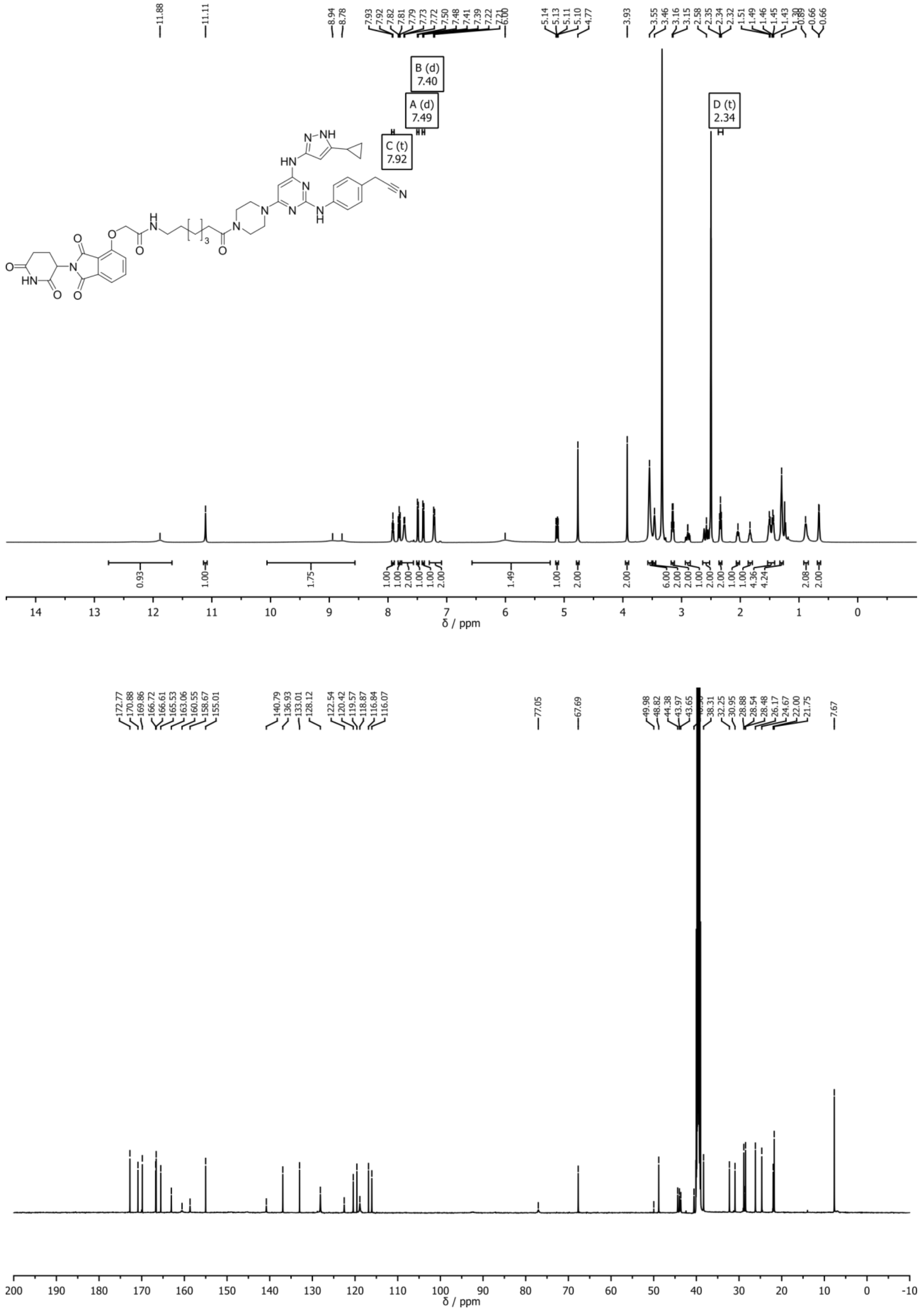

Figure S123: ^{1}H- (top) and ^{13}C-NMR (bottom) spectra (500 MHz and 126 MHz, 298 K, DMSO-d_6) and chemical structure of compound **C-1d**.

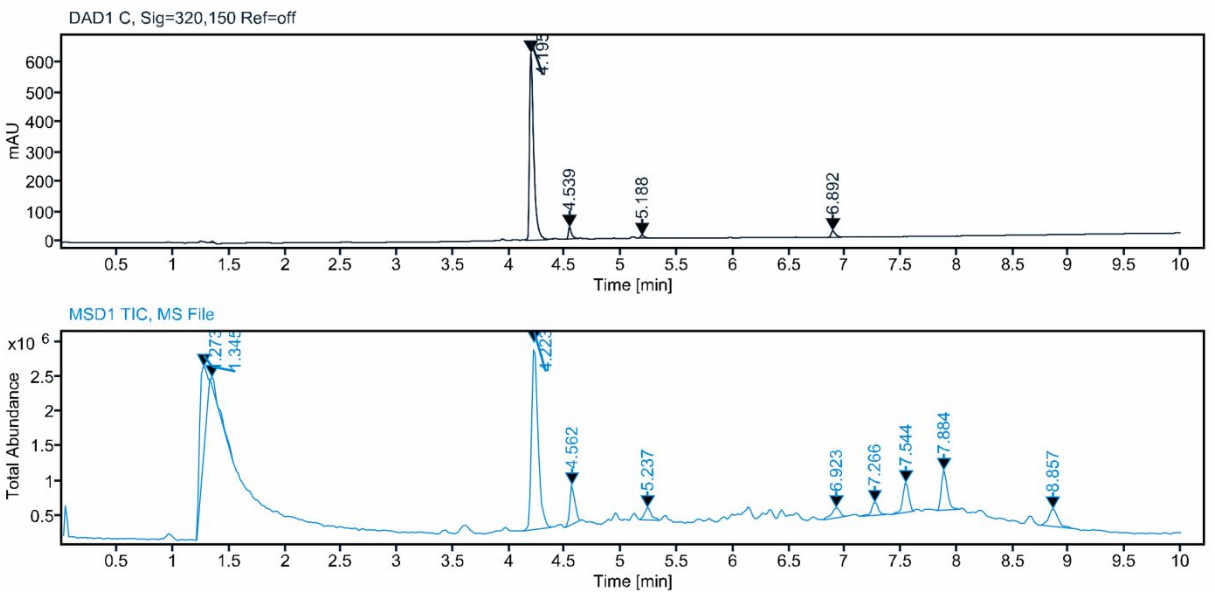

Sample Purity

Signal Description DAD1 C, Sig=320,150 Ref=off

Sample Name	Name	RT	Width	Area	Area%	Height
NM250-purity		4.195	0.039	1676.8339	90.85	627.4901
NM250-purity		4.539	0.029	78.8741	4.27	39.9436
NM250-purity		5.188	0.033	22.3128	1.21	9.6700
NM250-purity		6.892	0.042	67.6468	3.67	22.8966

Max Area% 90.852

UV Signal Purity>95% Fail

Figure S124: LC/MS spectra of purified compound **C-1d** at 320 nm wavelength.

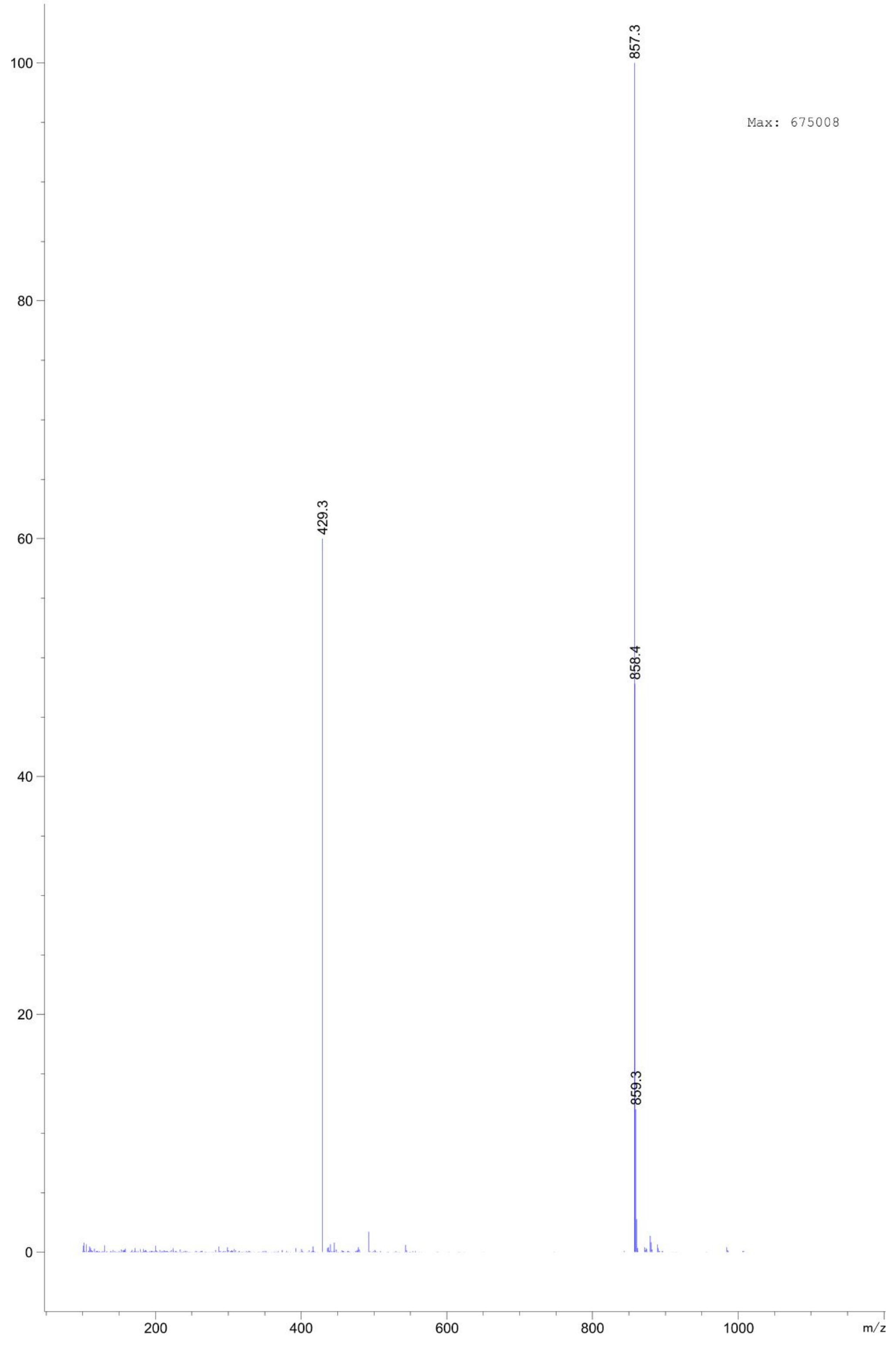

Figure S125: ESI-MS spectrum of compound **C-1d** with $[M+H]^{+}_{calc.}$ = 857.38 m/z.

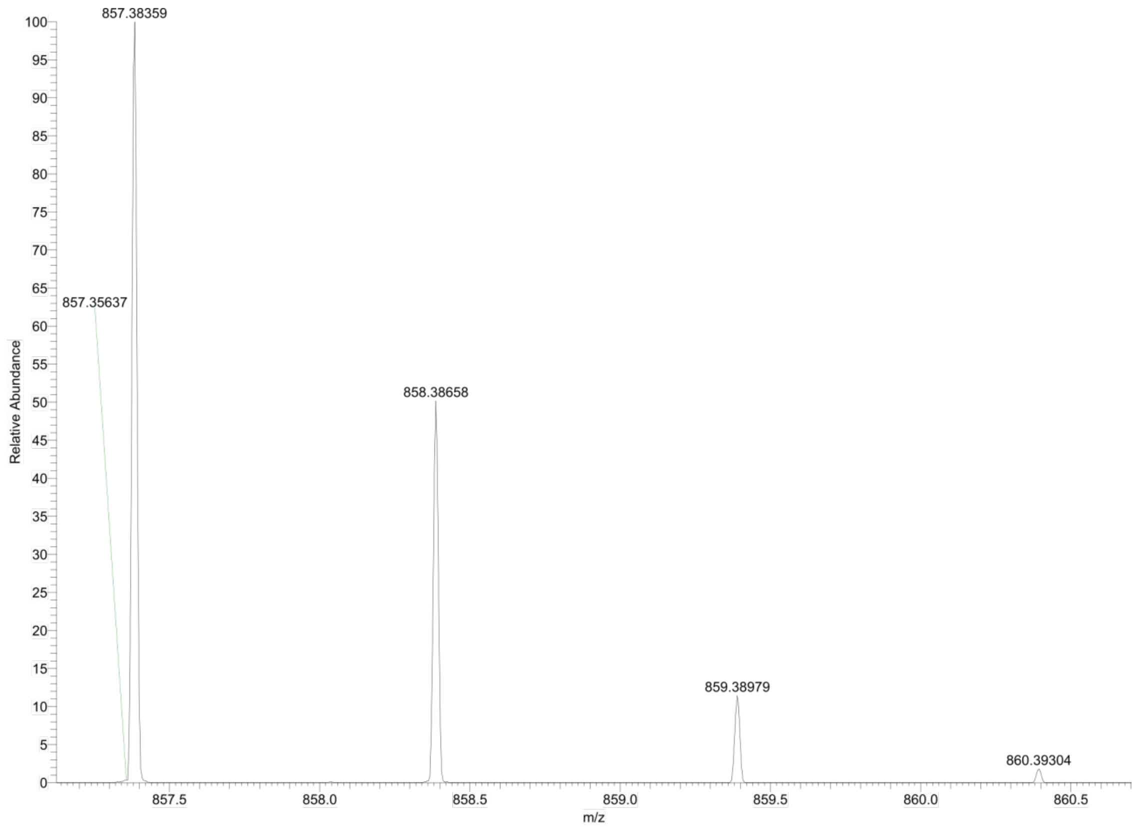

Figure S126: High-resolution mass spectrum of compound **C-1d** with $[M+H]^{+}_{calc.}$ = 857.38472 m/z.

7.2.3. DCAF1-recruiting Promiscuous Kinase PROTACs based on Kinase Parent Inhibitor 2inh

2-((2-((4-(4-(2-(2-(3-(4-(4-((2-Aminoethyl)amino)-2-(1-(4-chlorophenyl)cyclohexyl)quinazolin-7-yl)piperazin-1-yl)-3-oxopropoxy)ethoxy)ethyl)piperazin-1-yl)phenyl)amino)-5-chloropyrimidin-4-yl)amino)benzamide (D-2a)

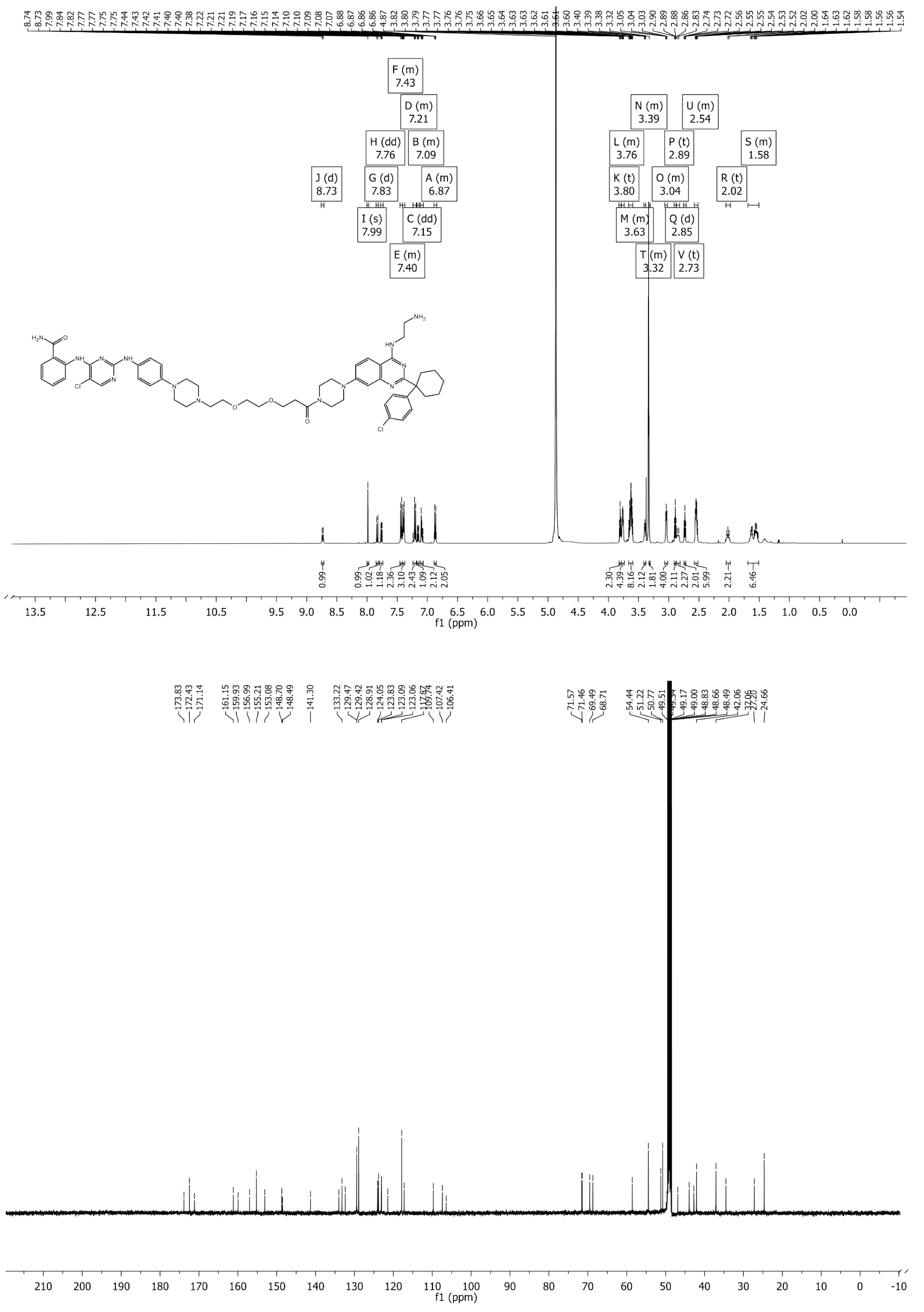

Figure S127: ^{1}H- (top) and ^{13}C-NMR (bottom) spectra (500 MHz and 126 MHz, 298 K, methanol-d_4) and chemical structure of compound **D-2a**.

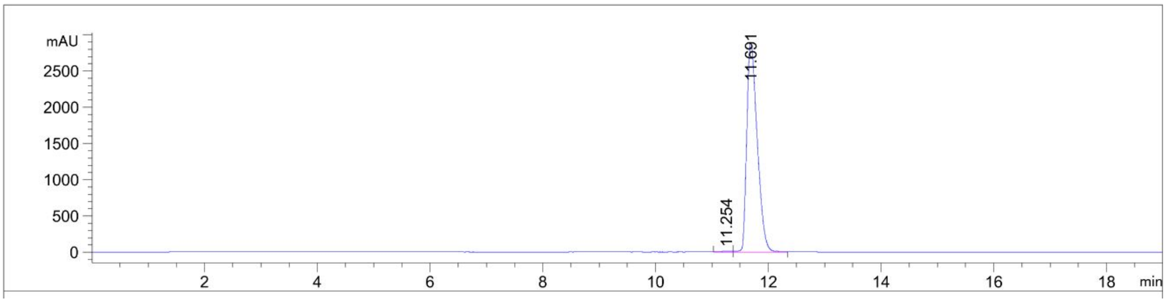

Signal 1: MWD1 A, Sig=254,4 Ref=off

Peak #	RetTime [min]	Type	Width [min]	Area [mAU*s]	Height [mAU]	Area %
1	11.254	BV	0.1876	175.09583	11.63589	0.4660
2	11.691	VV	0.1822	3.73955e4	2884.79541	99.5340
Totals :				3.75706e4	2896.43130	

Figure S128: LC/MS spectra of purified compound **D-2a** at 254 nm wavelength.

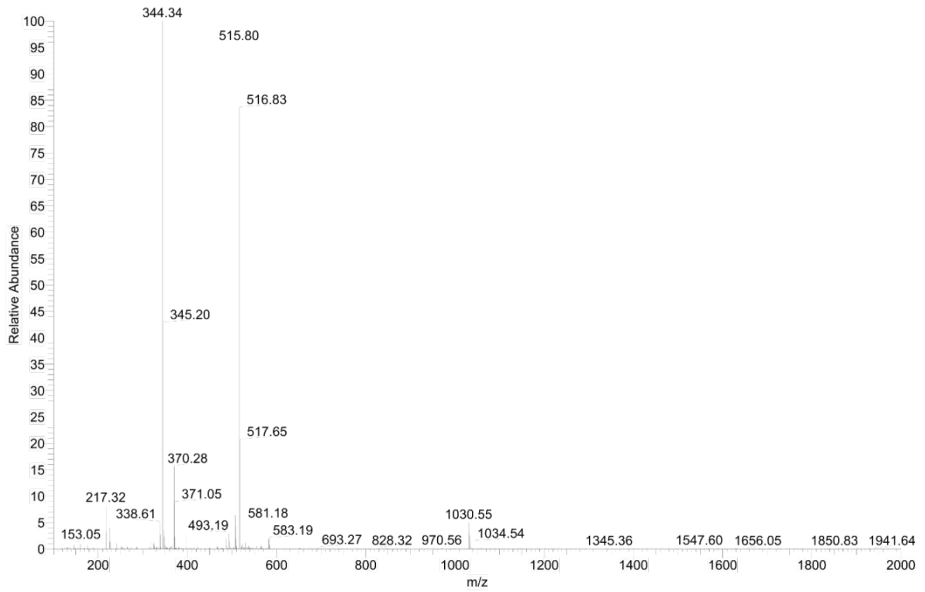

Figure S129: ESI-MS spectrum of compound **D-2a** with $[M+H]^+_{calc.}$ = 1030.48 m/z.

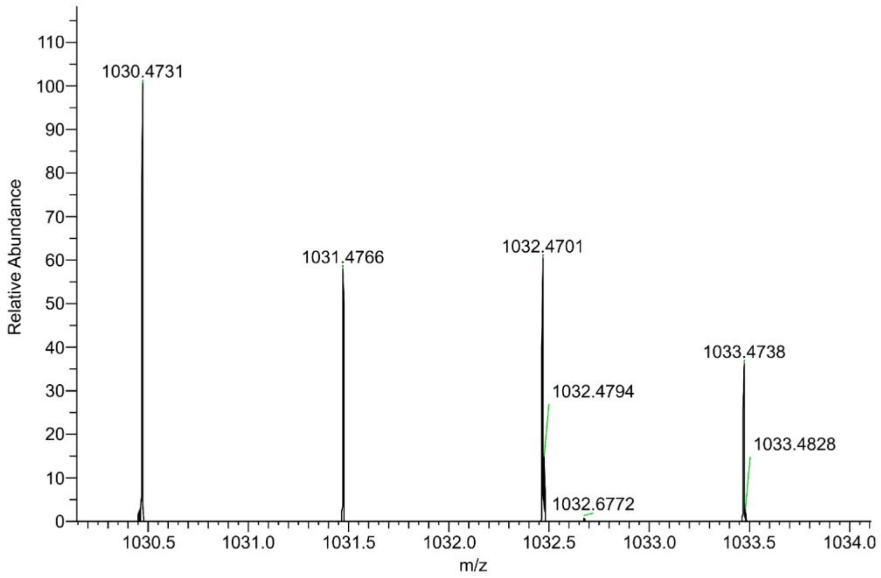

Figure S130: High-resolution mass spectrum of compound **D-2a** with $[M+H]^+_{calc.}$ = 1030.4732 m/z

Tert-butyl 3-(2-(2-(2-(4-(4-((4-((2-carbamoylphenyl)amino)-5-chloropyrimidin-2-yl)amino)phenyl)piperazin-1-yl)ethoxy)ethoxy)ethoxy)propanoate (S-46)

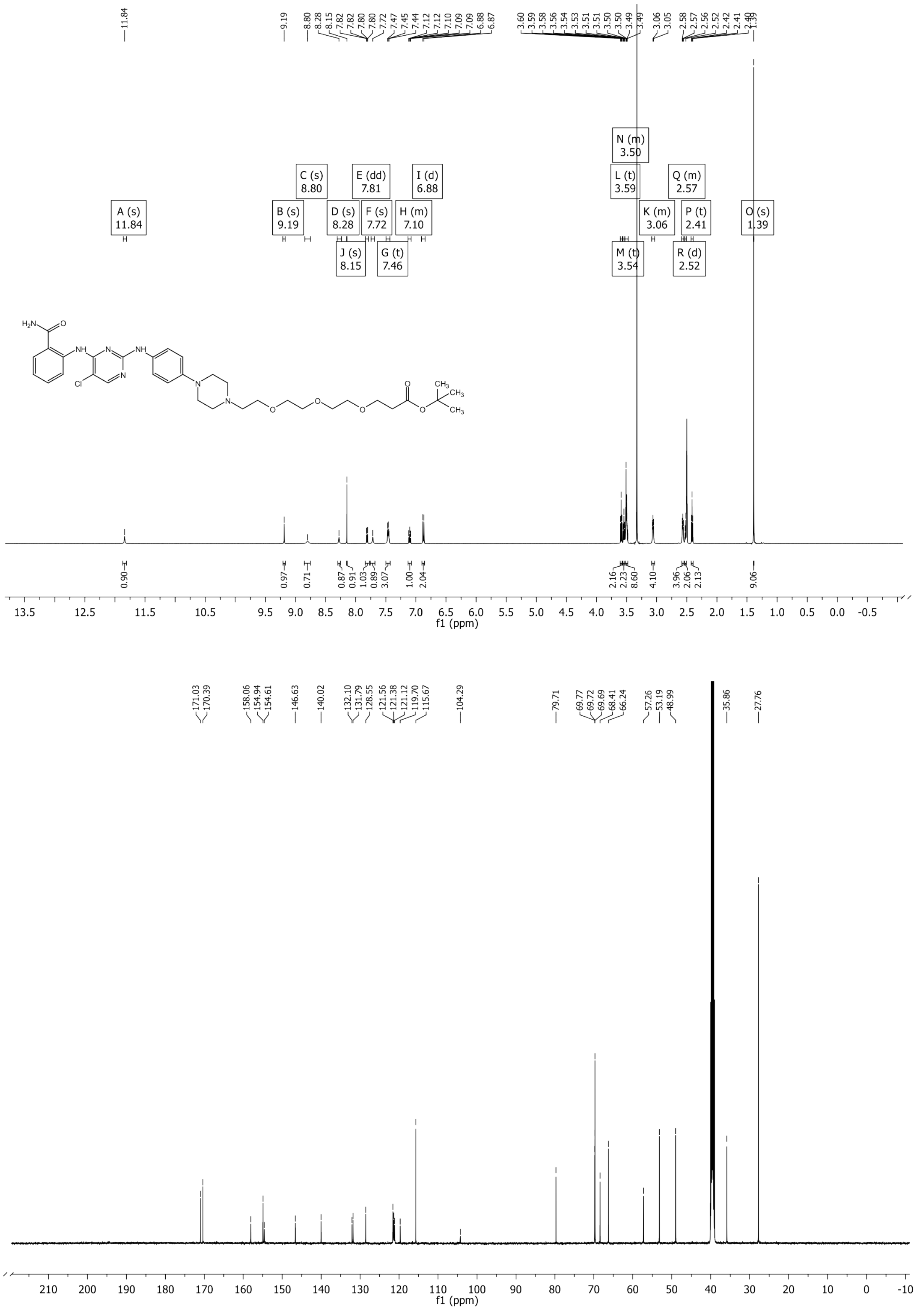

Figure S131: ^{1}H- (top) and ^{13}C-NMR (bottom) spectra (500 MHz and 126 MHz, 298 K, DMSO-d$_6$) and chemical structure of compound **S-46**.

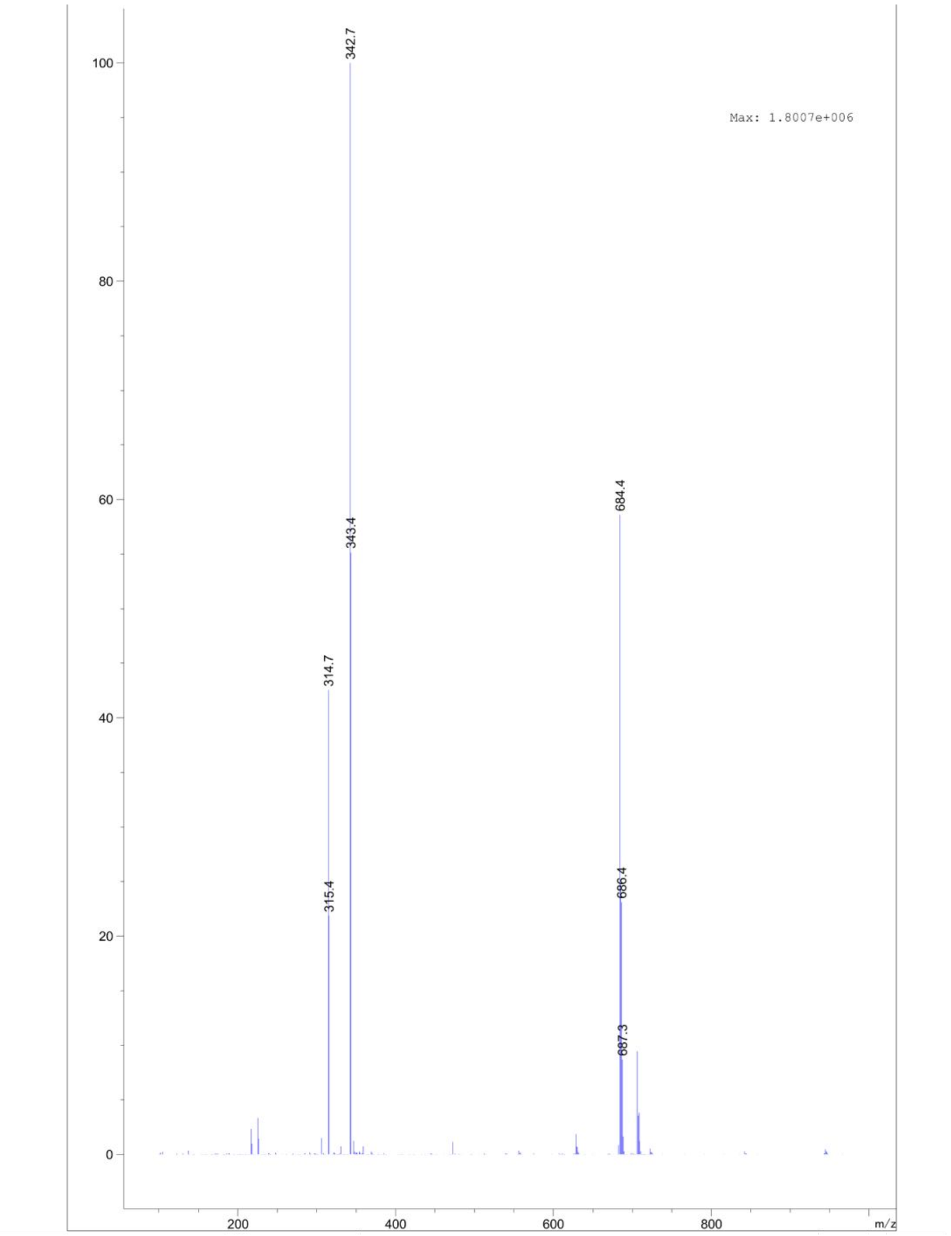

Figure S132: ESI-MS spectrum of compound **S-46** with $[M+H]^{+}_{calc.}$ = 684.4 m/z.

2-((2-((4-(4-(2-(2-(2-(3-(4-(4-((2-Aminoethyl)amino)-2-(1-(4-chlorophenyl)cyclohexyl)quinazolin-7-yl)piperazin-1-yl)-3-oxopropoxy)ethoxy)ethoxy)ethyl)piperazin-1-yl)phenyl)amino)-5-chloropyrimidin-4-yl)amino)benzamide (D-2b)

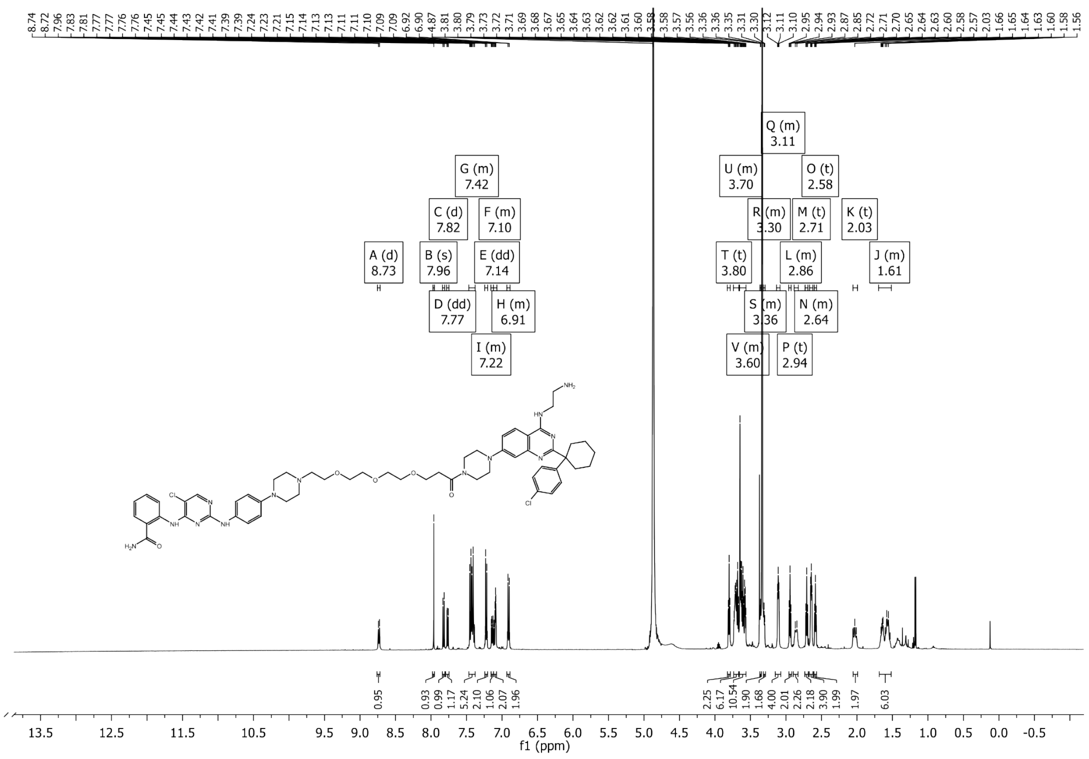

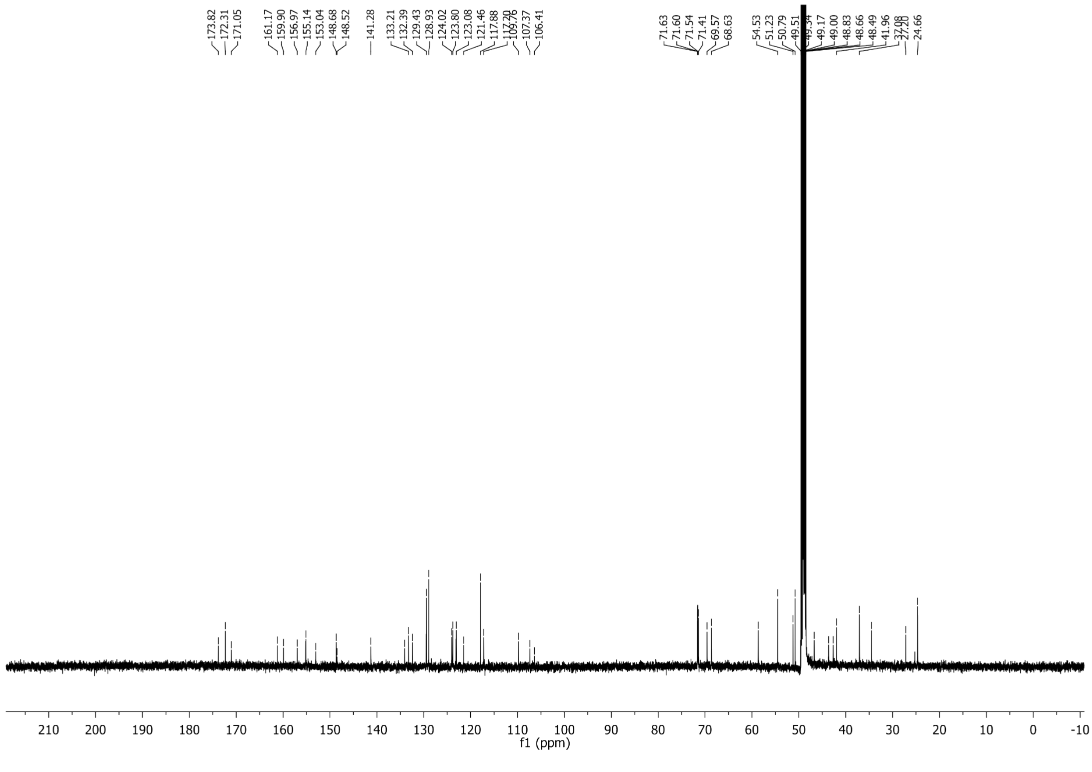

Figure S133: ^{1}H- (top) and ^{13}C-NMR (bottom) spectra (500 MHz and 126 MHz, 298 K, methanol-d_4) and chemical structure of compound **D-2b**.

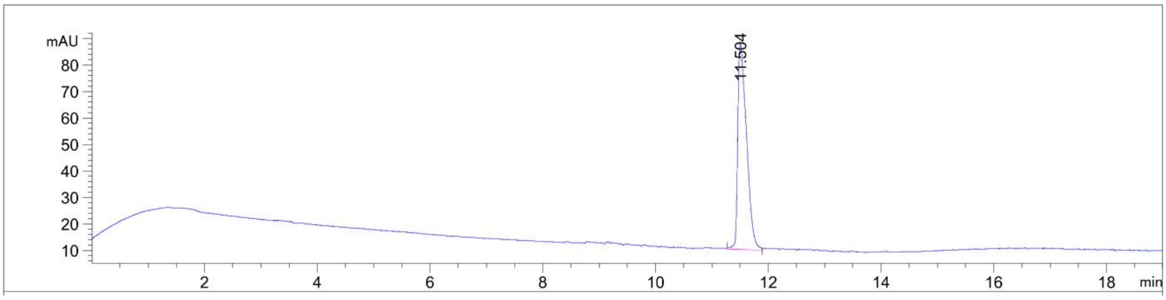

Signal 1: MWD1 A, Sig=254,4 Ref=off

Peak #	RetTime [min]	Type	Width [min]	Area [mAU*s]	Height [mAU]	Area %
1	11.504	VV	0.1527	840.31433	77.96635	100.0000
Totals :				840.31433	77.96635	

Figure S134: LC/MS spectra of purified compound **D-2b** at 254 nm wavelength.

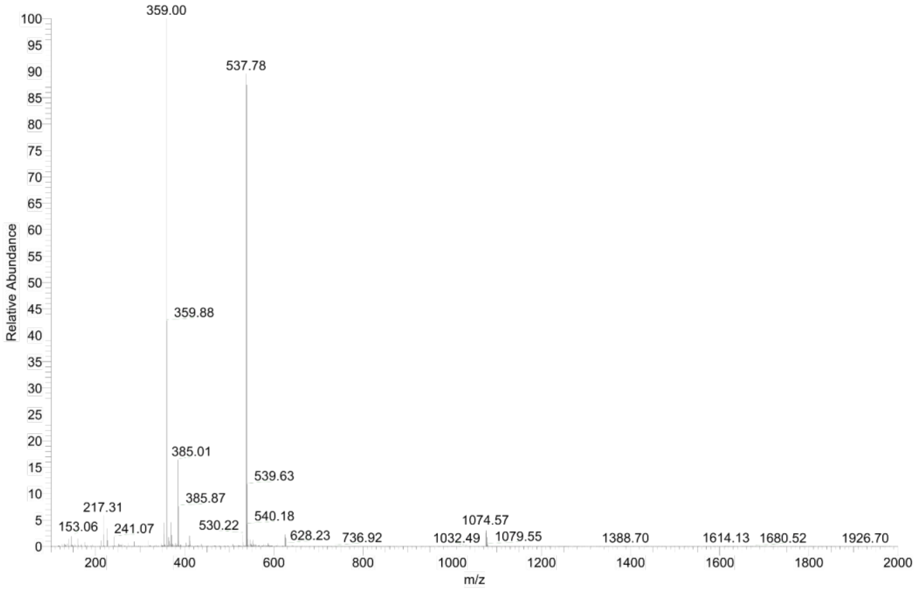

Figure S135: ESI-MS spectrum of compound **D-2b** with $[M+H]^+_{calc.}$ = 1074.50 m/z.

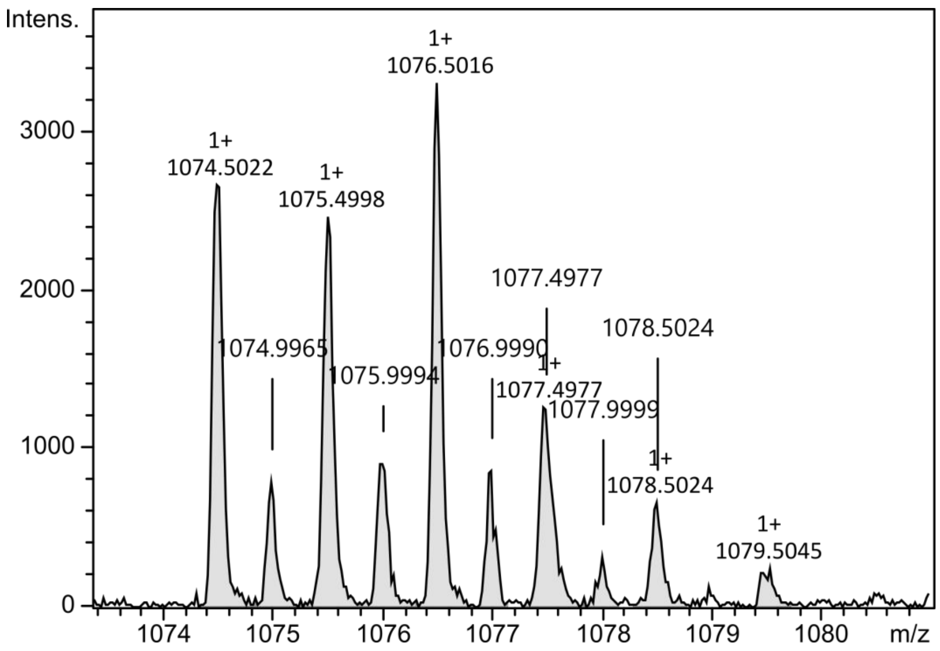

Figure S136: High-resolution mass spectrum of compound **D-2b** with $[M+H]^{+}_{calc.}$ = 1074.4994 m/z

2-((2-((4-(4-(15-(4-(4-((2-Aminoethyl)amino)-2-(1-(4-chlorophenyl)cyclohexyl)quinazolin-7-yl)piperazin-1-yl)-15-oxo-3,6,9,12-tetraoxapentadecyl)piperazin-1-yl)phenyl)amino)-5-chloropyrimidin-4-yl)amino)benzamide (D-2c)

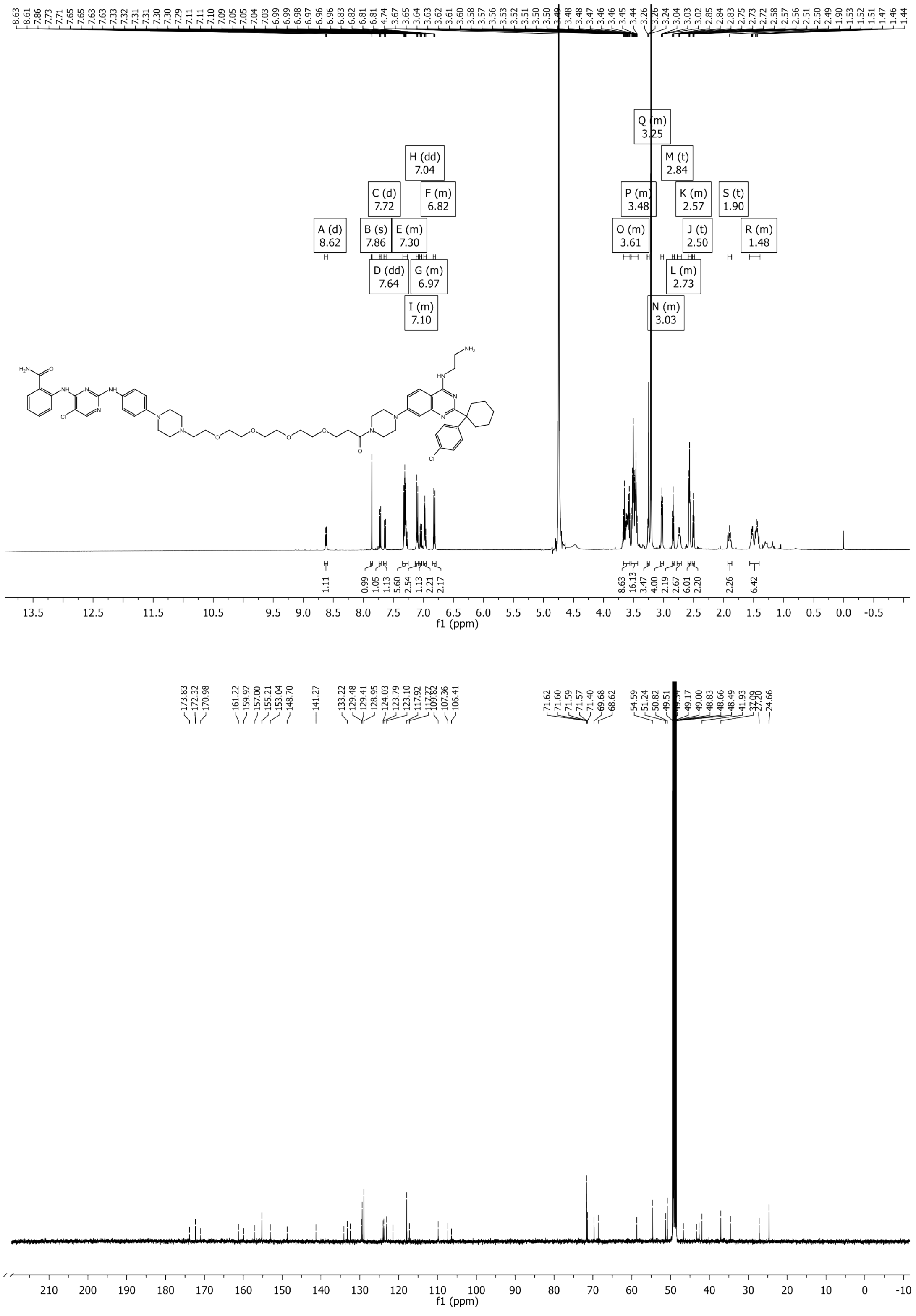

Figure S137: ^{1}H- (top) and ^{13}C-NMR (bottom) spectra (500 MHz and 126 MHz, 298 K, methanol-d$_4$) and chemical structure of compound **D-2c**.

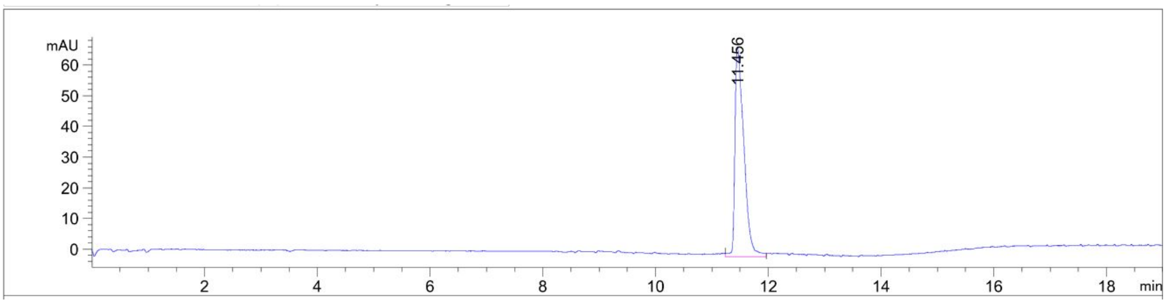


```
Signal 1: MWD1 A, Sig=254,4 Ref=off

Peak RetTime Type  Width     Area      Height     Area
  #   [min]        [min]   [mAU*s]     [mAU]        %
----|-------|----|-------|----------|----------|--------|
   1  11.456 VV    0.1557  752.30511   68.19733 100.0000

Totals :                   752.30511   68.19733
```

Figure S138: LC/MS spectra of purified compound **D-2c** at 254 nm wavelength.

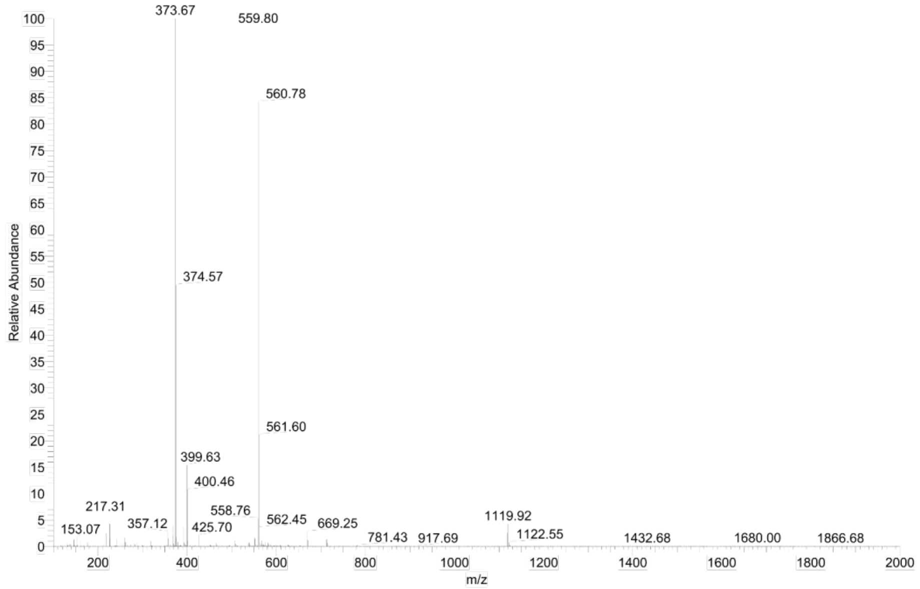

Figure S139: ESI-MS spectrum of compound **D-2c** with $[M+H]^+_{calc.}$ = 1118.53 m/z.

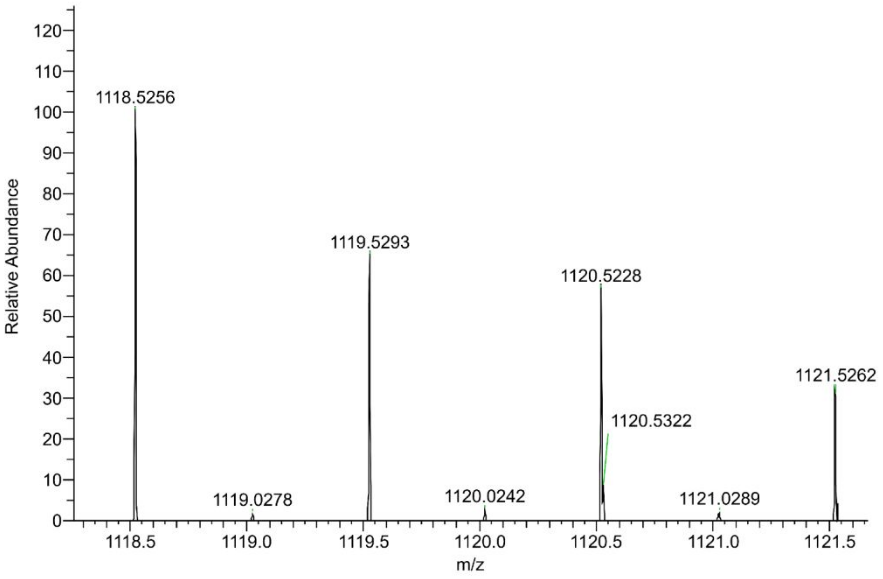

Figure S140: High-resolution mass spectrum of compound **D-2c** with $[M+H]^{+}_{calc.}$ = 1118.5256 m/z

Ethyl 5-(4-(4-((4-((2-carbamoylphenyl)amino)-5-chloropyrimidin-2-yl)amino)phenyl)piperazin-1-yl)pentanoate (S-48)

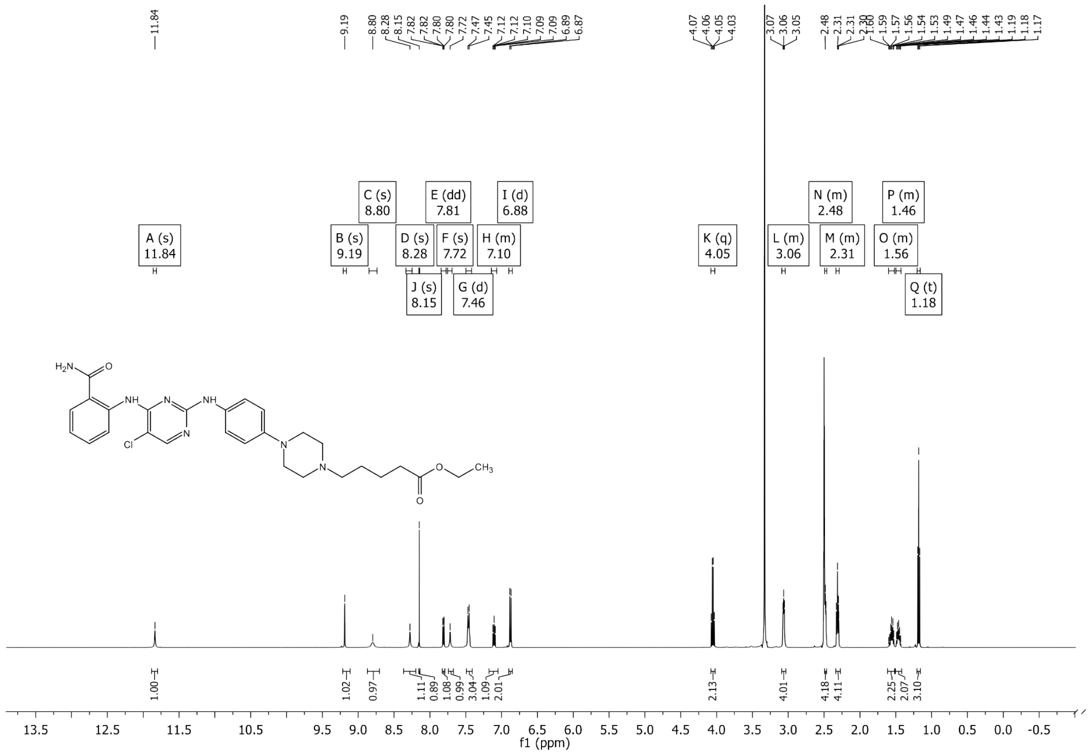

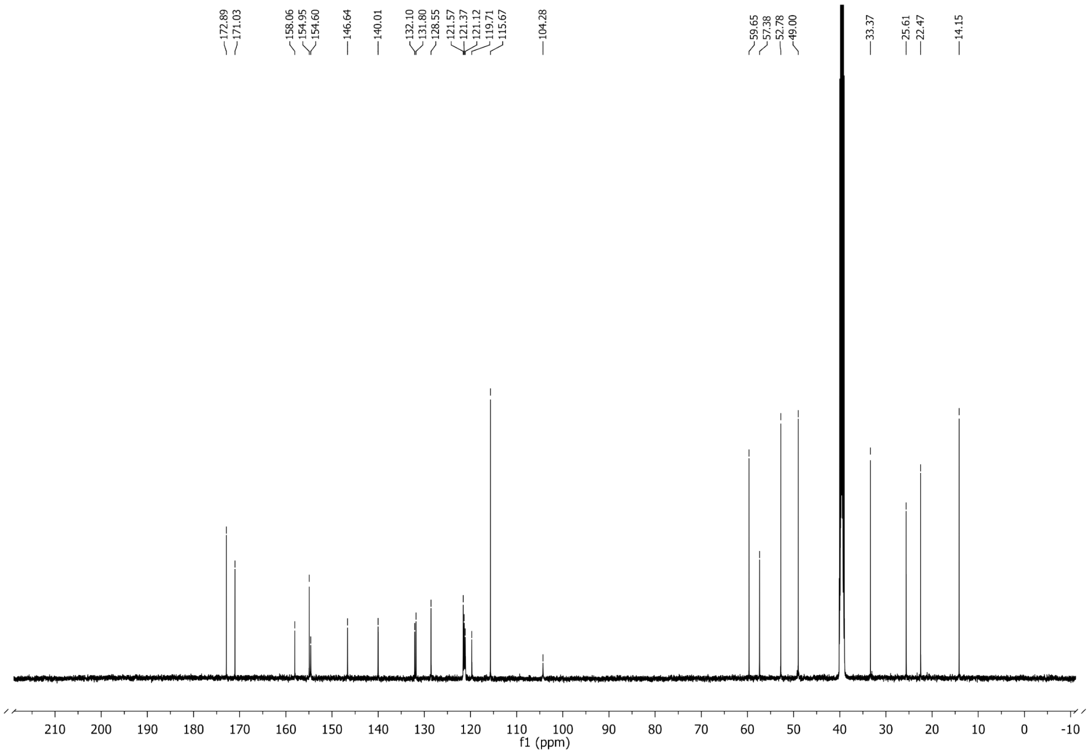

Figure S141: ^{1}H- (top) and ^{13}C-NMR (bottom) spectra (500 MHz and 126 MHz, 298 K, DMSO-d_6) and chemical structure of compound **S-48.**

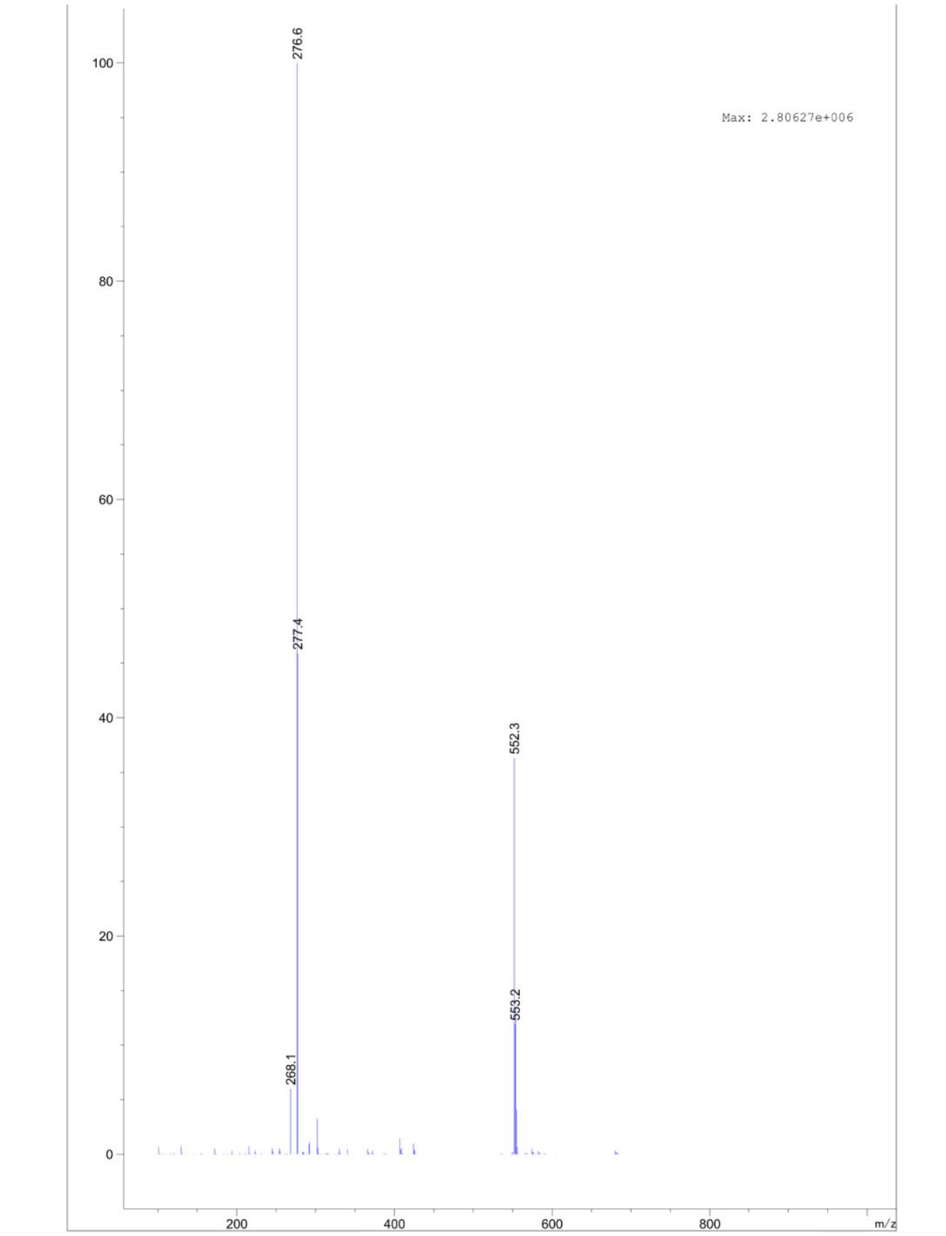

Figure S142: ESI-MS spectrum of compound **S-48** with $[M+H]^{+}_{calc.}$ = 552.3 m/z.

2-((2-((4-(4-(5-(4-(4-((2-Aminoethyl)amino)-2-(1-(4-chlorophenyl)cyclohexyl)quinazolin-7-yl)piperazin-1-yl)-5-oxopentyl)piperazin-1-yl)phenyl)amino)-5-chloropyrimidin-4-yl)amino)benzamide (D-2d)

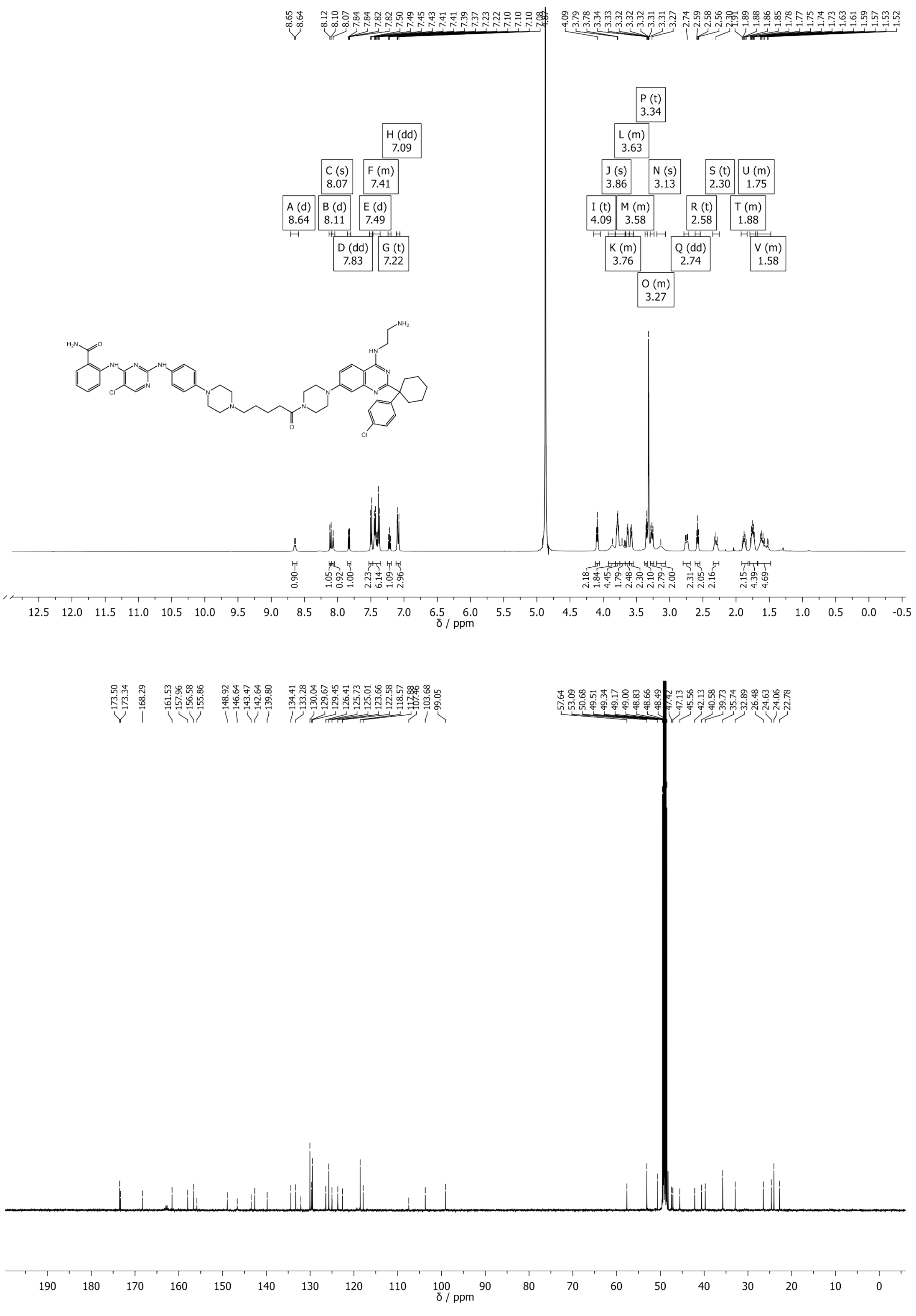

Figure S143: ^{1}H- (top) and ^{13}C-NMR (bottom) spectra (500 MHz and 126 MHz, 298 K, methanol-d_4) and chemical structure of compound **D-2d.**

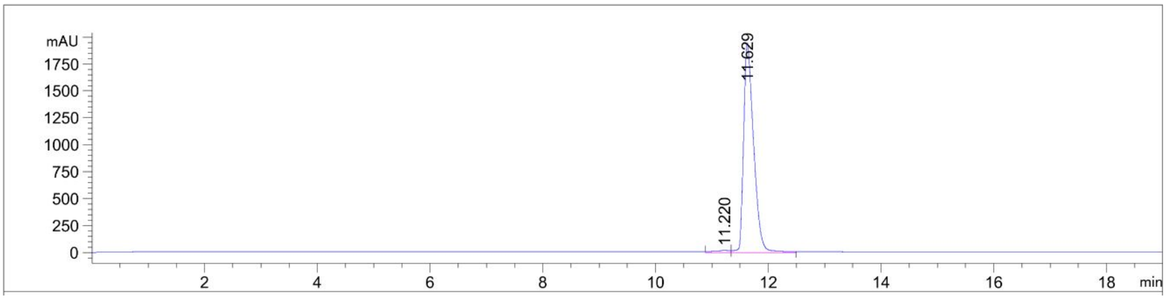

Signal 1: MWD1 A, Sig=254,4 Ref=off

Peak #	RetTime [min]	Type	Width [min]	Area [mAU*s]	Height [mAU]	Area %
1	11.220	VV	0.2551	436.96893	22.35284	1.7764
2	11.629	VV	0.1798	2.41609e4	1946.88855	98.2236
Totals :				2.45979e4	1969.24139	

Figure S144: LC/MS spectra of purified compound **D-2d** at 254 nm wavelength.

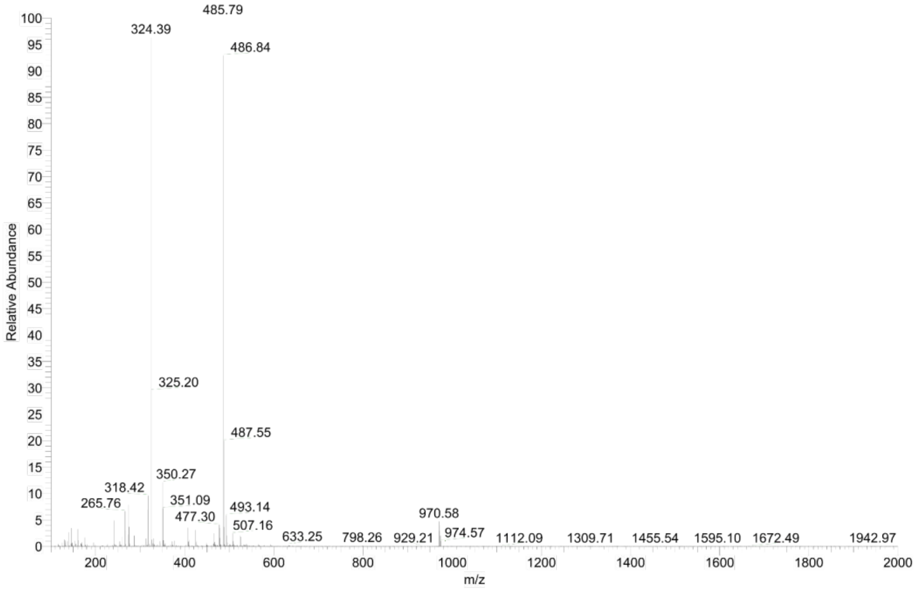

Figure S145: ESI-MS spectrum of compound **D-2d** with $[M+H]^+_{calc.}$ = 970.45 m/z.

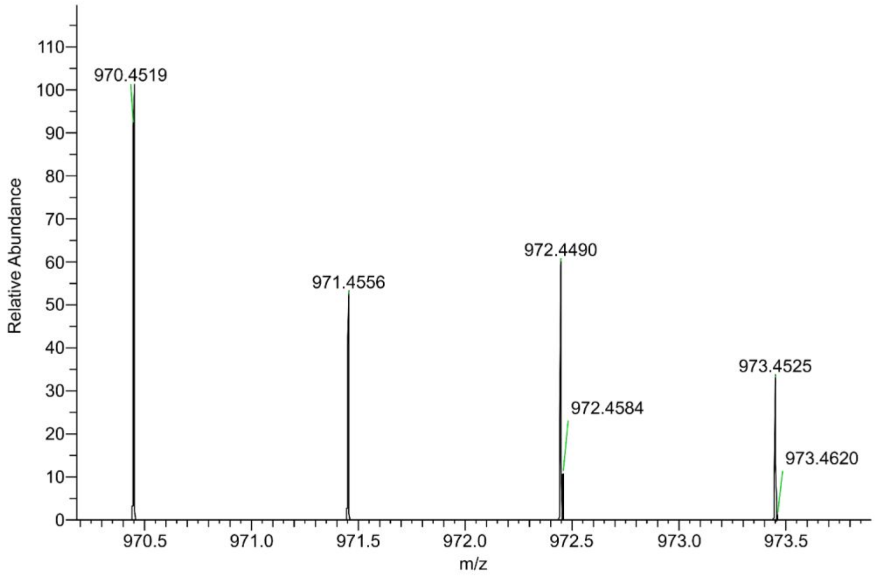

Figure S146: High-resolution mass spectrum of compound **D-2d** with $[M+H]^+_{calc.}$ = 970.4519 m/z

2-((2-((4-(4-(7-(4-(4-((2-Aminoethyl)amino)-2-(1-(4-chlorophenyl)cyclohexyl)quinazolin-7-yl)piperazin-1-yl)-7-oxoheptyl)piperazin-1-yl)phenyl)amino)-5-chloropyrimidin-4-yl)amino)benzamide (D-2e)

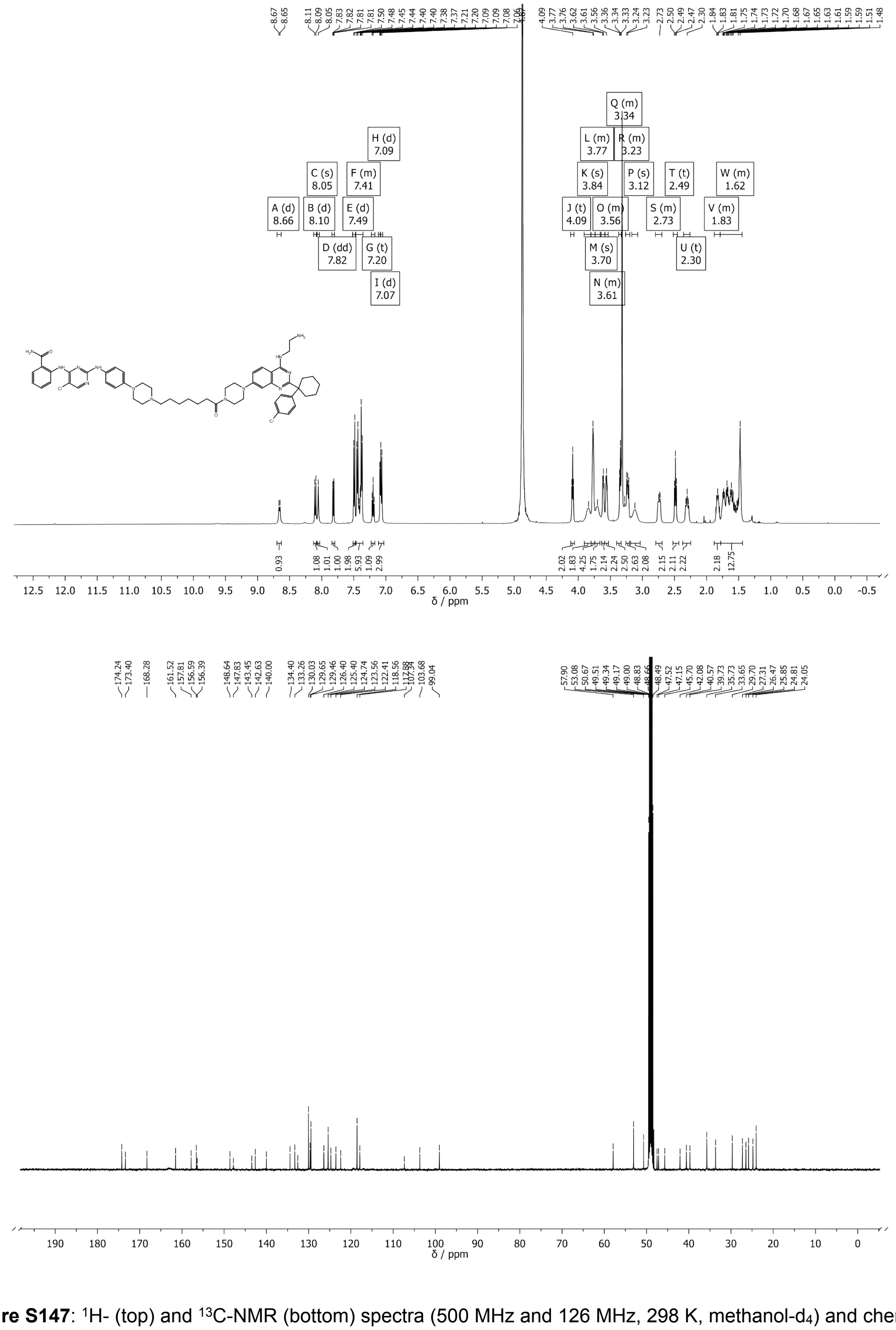

Figure S147: ^{1}H- (top) and ^{13}C-NMR (bottom) spectra (500 MHz and 126 MHz, 298 K, methanol-d_4) and chemical structure of compound **D-2e.**

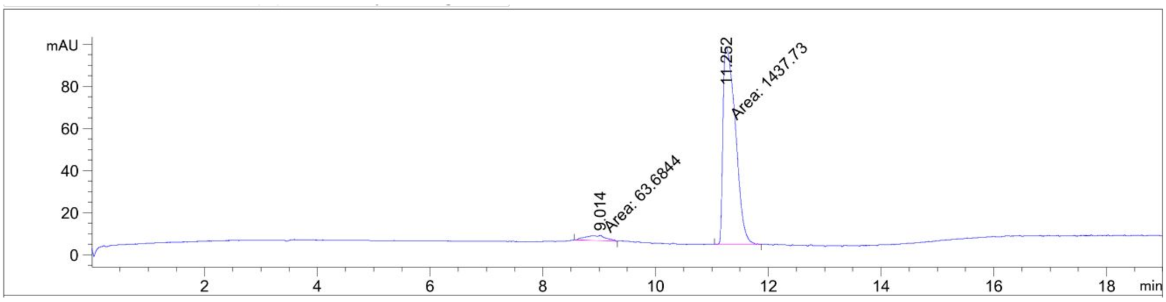

Signal 1: MWD1 A, Sig=254,4 Ref=off

Peak #	RetTime [min]	Type	Width [min]	Area [mAU*s]	Height [mAU]	Area %
1	9.014	MM	0.4150	63.68442	2.55762	4.2416
2	11.252	MM	0.2561	1437.72925	93.57780	95.7584
Totals :				1501.41367	96.13542	

Figure S148: LC/MS spectra of purified compound **D-2e** at 254 nm wavelength.

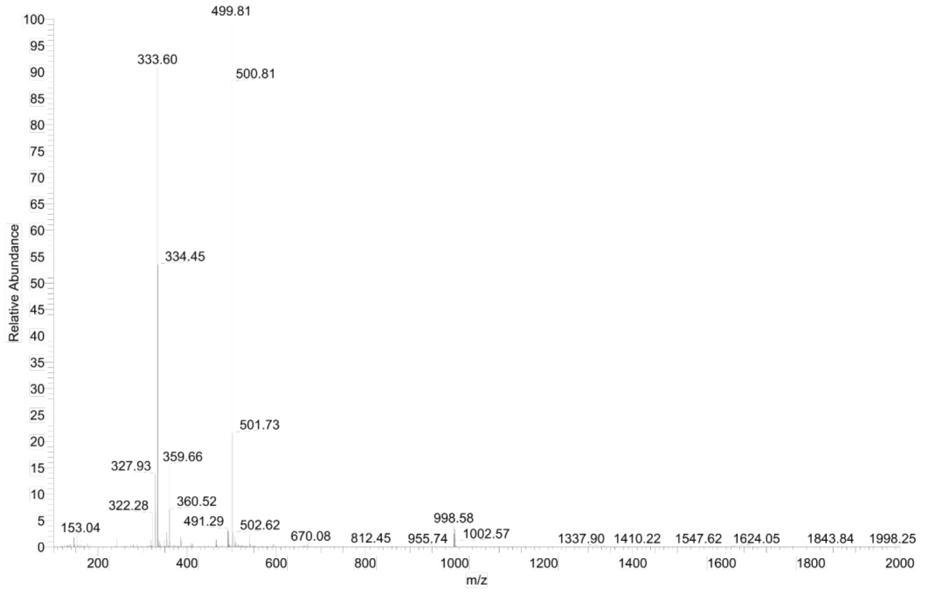

Figure S149: ESI-MS spectrum of compound **D-2e** with $[M+H]^+_{calc.}$ = 998.49 m/z.

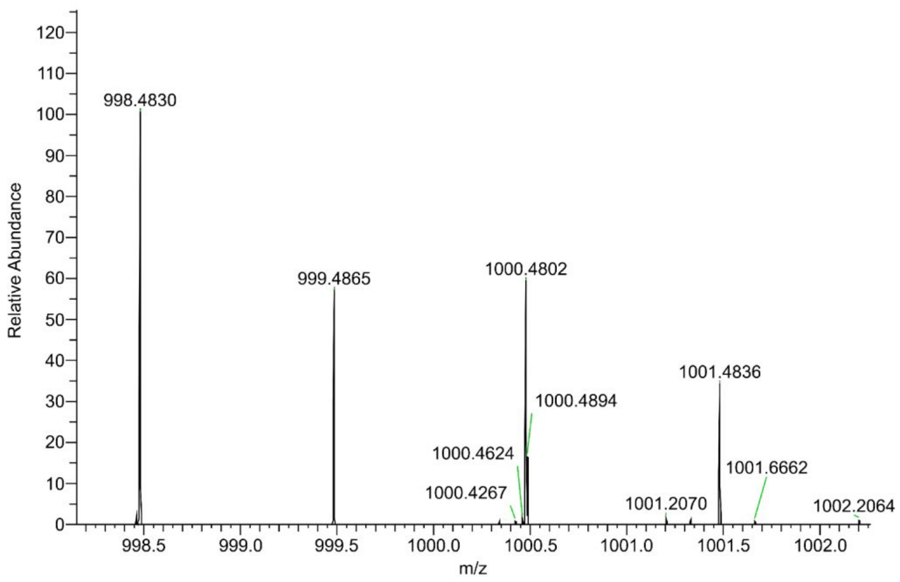

Figure S150: High-resolution mass spectrum of compound **D-2e** with $[M+H]^{+}_{calc.}$ = 998.4834 m/z

Ethyl 10-(4-(4-((4-((2-carbamoylphenyl)amino)-5-chloropyrimidin-2-yl)amino)phenyl)piperazin-1-yl)decanoate (S-50)

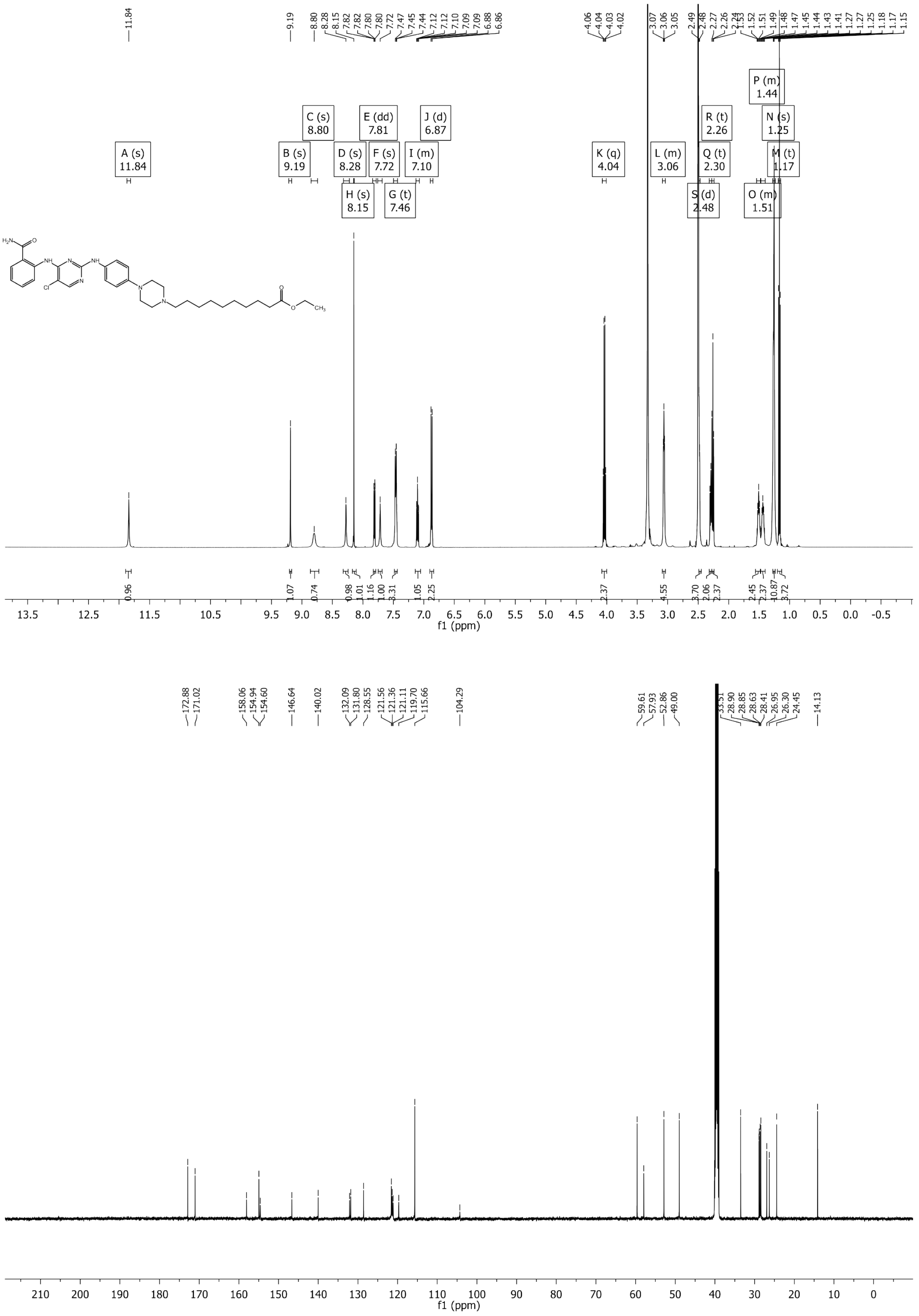

Figure S151: ^{1}H- (top) and ^{13}C-NMR (bottom) spectra (500 MHz and 126 MHz, 298 K, DMSO-d_6) and chemical structure of compound **S-50.**

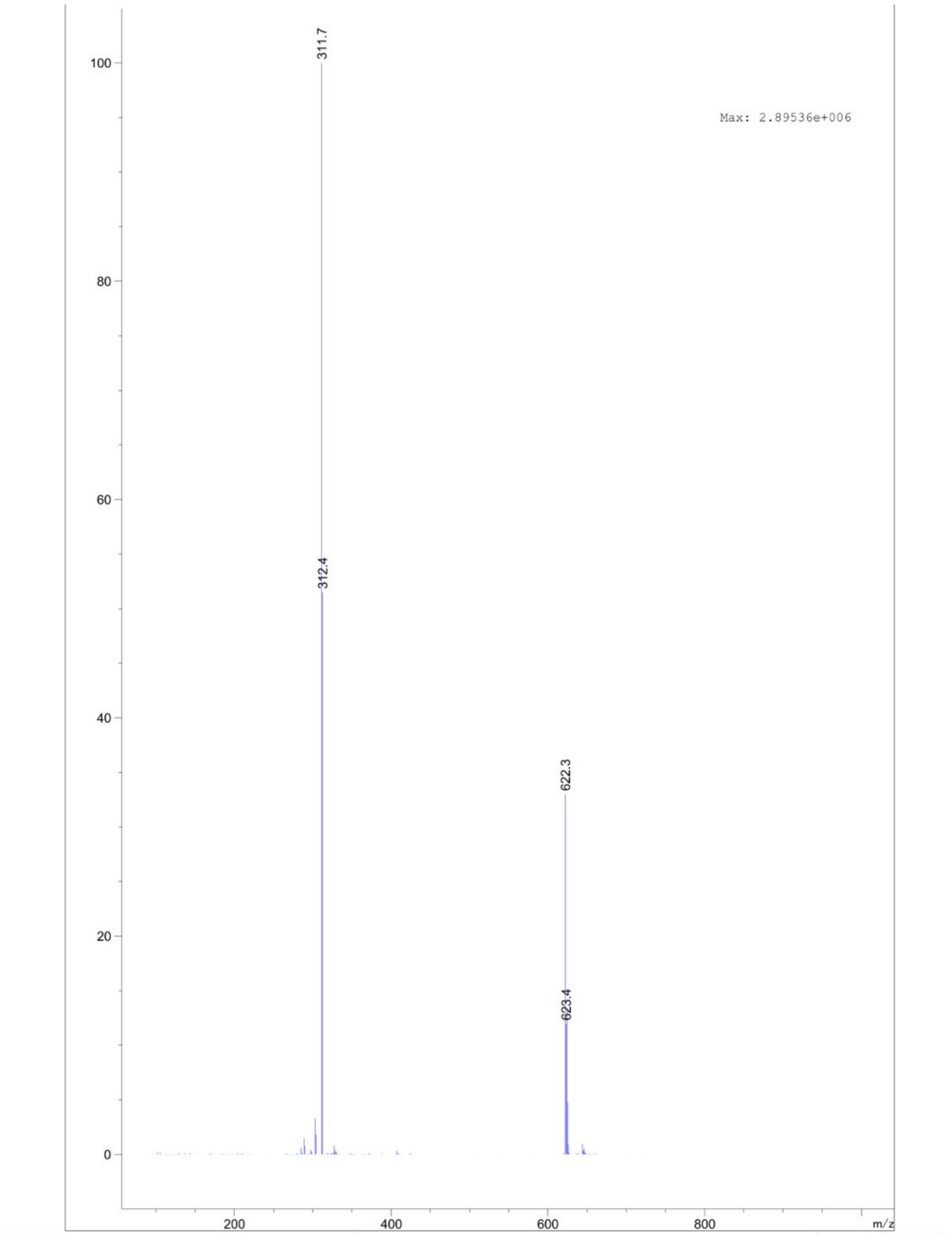

Figure S152: ESI-MS spectrum of compound **S-50** with $[M+H]^{+}_{calc.}$ = 622.3 m/z.

2-((2-((4-(4-(10-(4-(4-((2-Aminoethyl)amino)-2-(1-(4-chlorophenyl)cyclohexyl)quinazolin-7-yl)piperazin-1-yl)-10-oxodecyl)piperazin-1-yl)phenyl)amino)-5-chloropyrimidin-4-yl)amino)benzamide (D-2f)

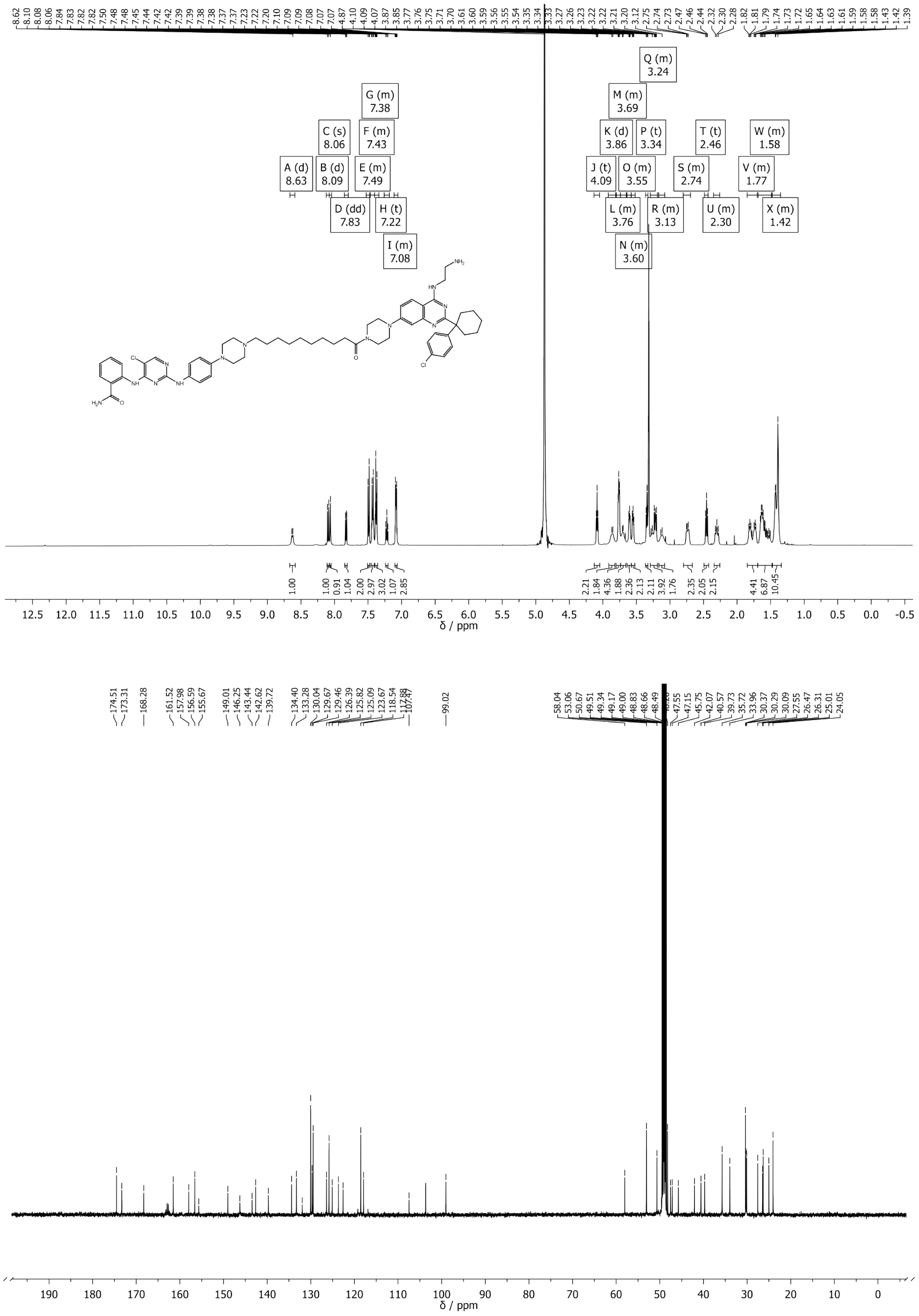

Figure S153: ^{1}H- (top) and ^{13}C-NMR (bottom) spectra (500 MHz and 126 MHz, 298 K, methanol-d_4) and chemical structure of compound **D-2f.**

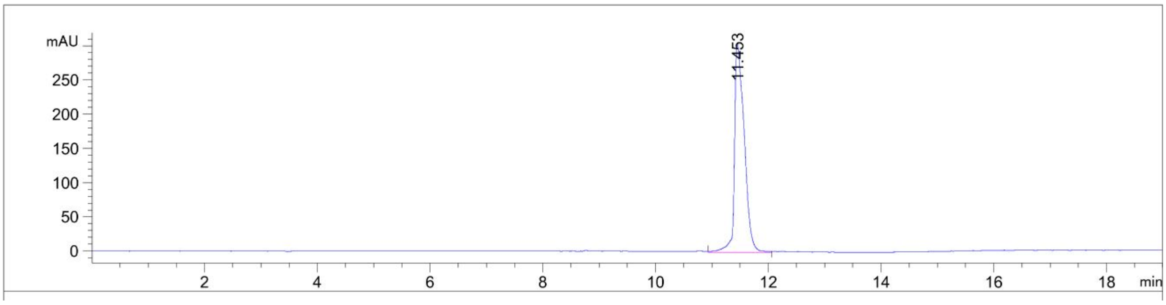

Signal 1: MWD1 A, Sig=254,4 Ref=off

Peak #	RetTime [min]	Type	Width [min]	Area [mAU*s]	Height [mAU]	Area %
1	11.453	VV	0.1654	3679.49902	305.83560	100.0000
Totals :				3679.49902	305.83560	

Figure S154: LC/MS spectra of purified compound **D-2f** at 254 nm wavelength.

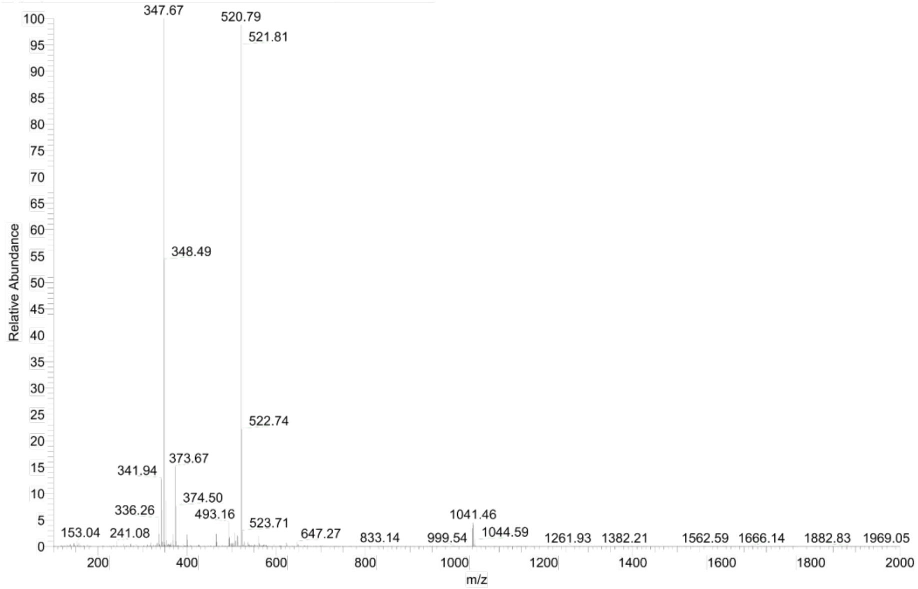

Figure S155: ESI-MS spectrum of compound **D-2f** with $[M+H]^+_{calc.}$ = 1041.53 m/z.

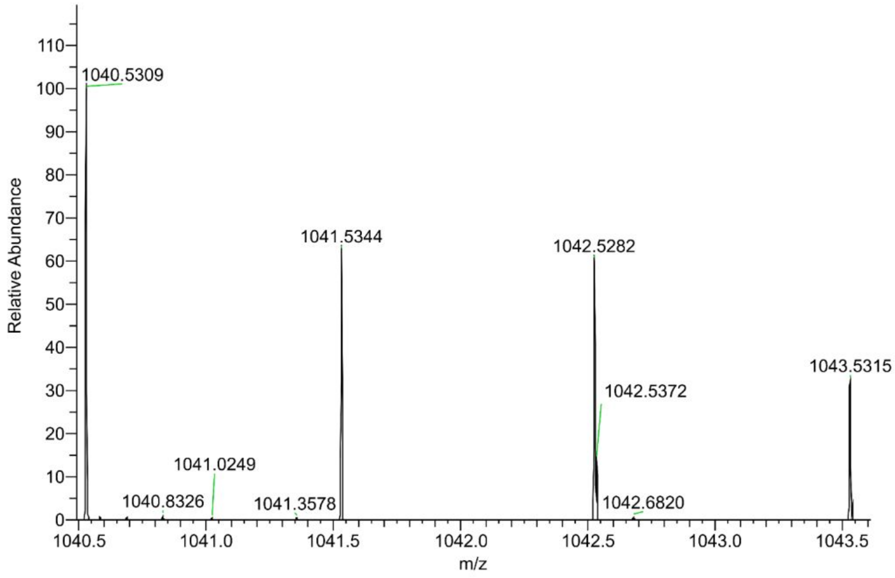

Figure S156: High-resolution mass spectrum of compound **D-2f** with $[M+H]^{+}_{calc.}$ = 1040.5303 m/z

Tert-butyl (2-((7-(4-(1-(4-(4-((4-((2-carbamoylphenyl)amino)-5-chloropyrimidin-2-yl)amino)phenyl)piperazin-1-yl)-3,6,9,12-tetraoxapentadecan-15-oyl)piperazin-1-yl)-2-(1-(4-chlorophenyl)cyclohexyl)quinazolin-4-yl)amino)ethyl)carbamate (D-2c$^{n.c.}$)

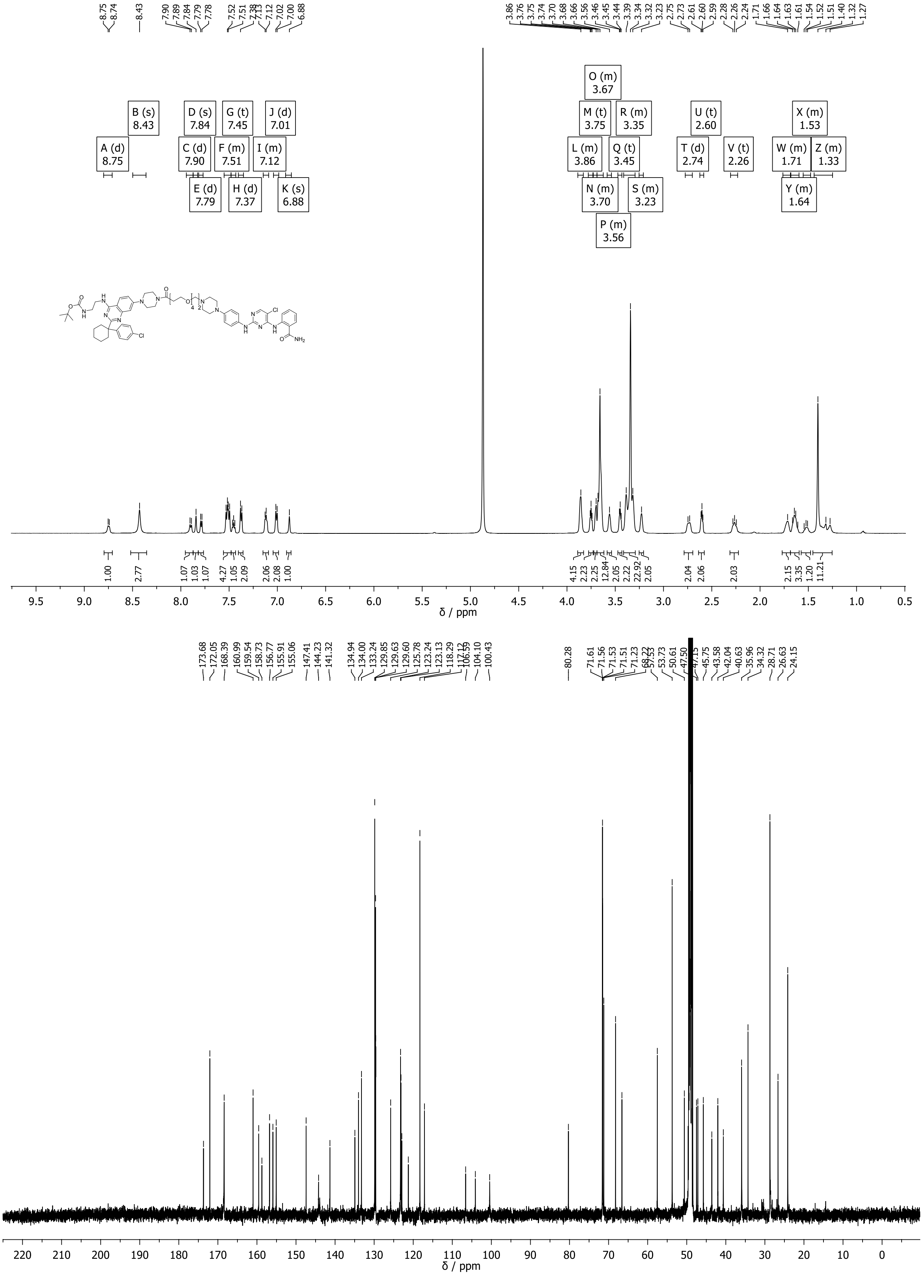

Figure S157: ^{1}H- (top) and ^{13}C-NMR (bottom) spectra (500 MHz and 126 MHz, 298 K, methanol-d$_4$) and chemical structure of compound **D-2c$^{n.c.}$**.

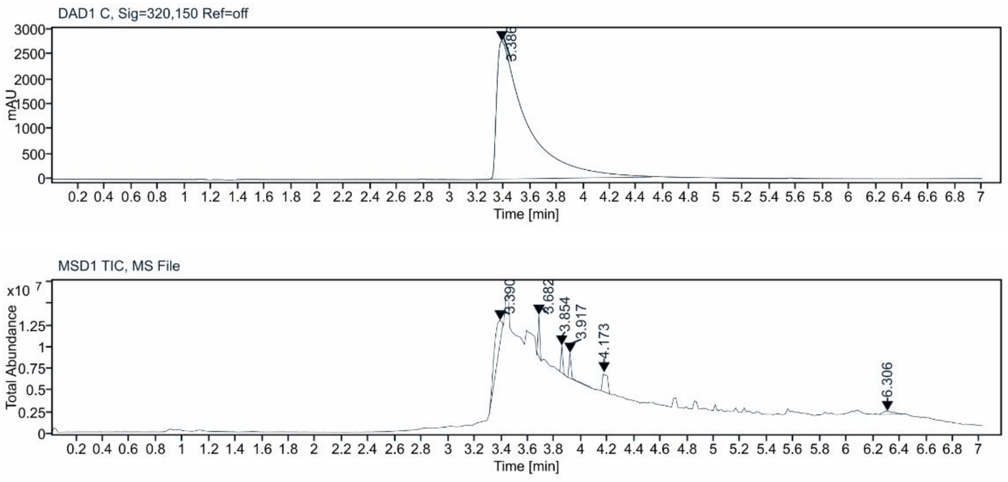

Signal Description	DAD1 C, Sig=320,150 Ref=off

Sample Name	Name	RT	Width	Area	Area%	Height
jw-620_x		3.386	0.193	43174.15 23	100.00	2760.8042

Max Area%	100.000
UV Signal Purity>95%	Pass

Figure S158: LC/MS spectra of purified compound **D-2c$^{n.c.}$** at 320 nm wavelength.

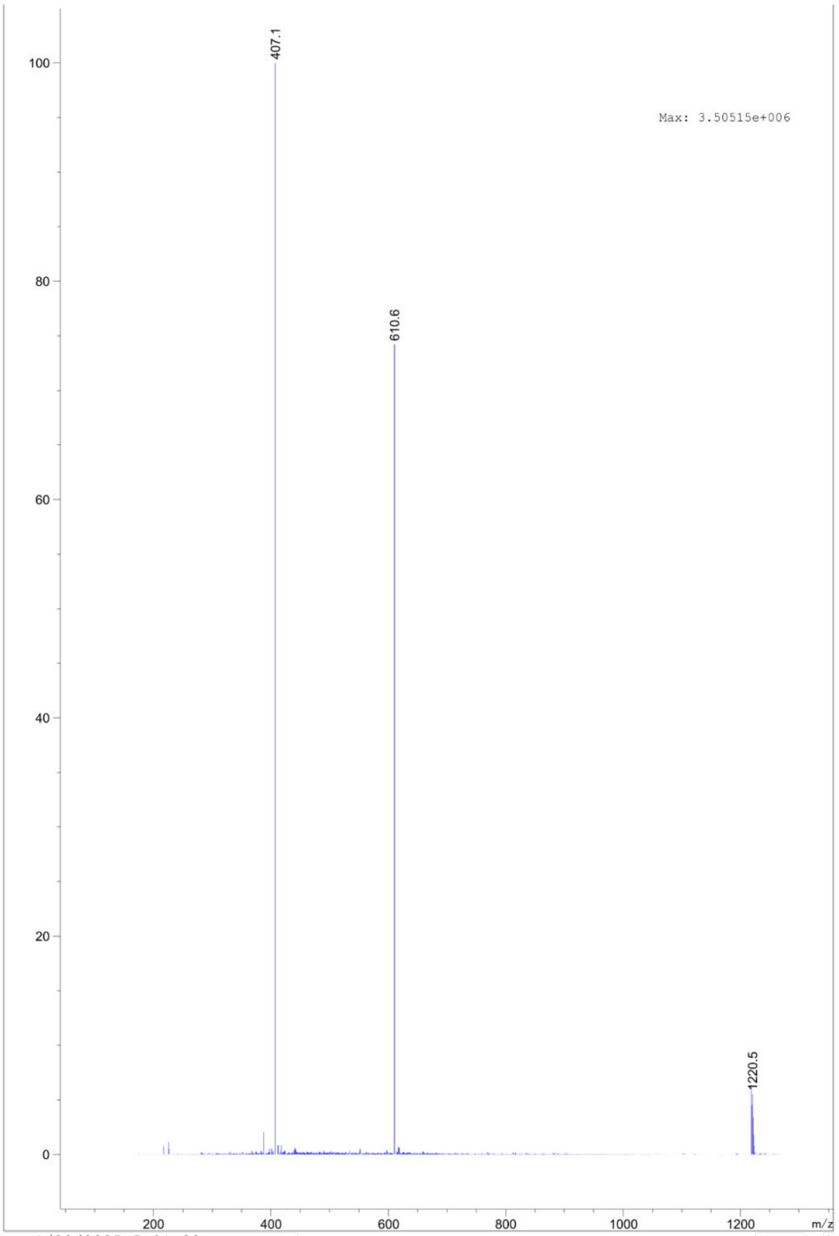

Figure S159: ESI-MS spectrum of compound **D-2c**$^{\mathbf{n.c.}}$ with $[M/2+H]^{+}_{calc.}$ = 609.8 m/z.

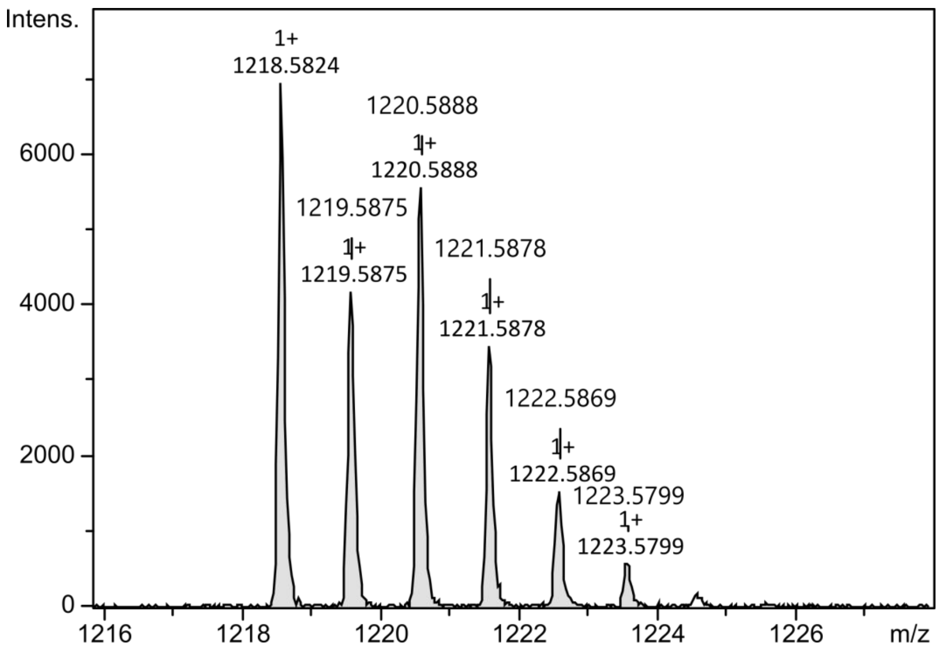

Figure S160: High-resolution mass spectrum of compound **D-2c$^{n.c.}$** with $[M+H]^{+}_{calc.}$ = 1218.5781 m/z

7.2.4. CRBN-recruiting Promiscuous Kinase PROTACs based on Kinase Parent Inhibitor 2inh

2-((5-Chloro-2-((4-(4-(3-(2-(2-((2-(2,6-dioxopiperidin-3-yl)-1,3-dioxoisoindolin-4-yl)oxy)ethoxy)ethoxy)propanoyl)piperazin-1-yl)phenyl)amino)pyrimidin-4-yl)amino)benzamide (C-2a)

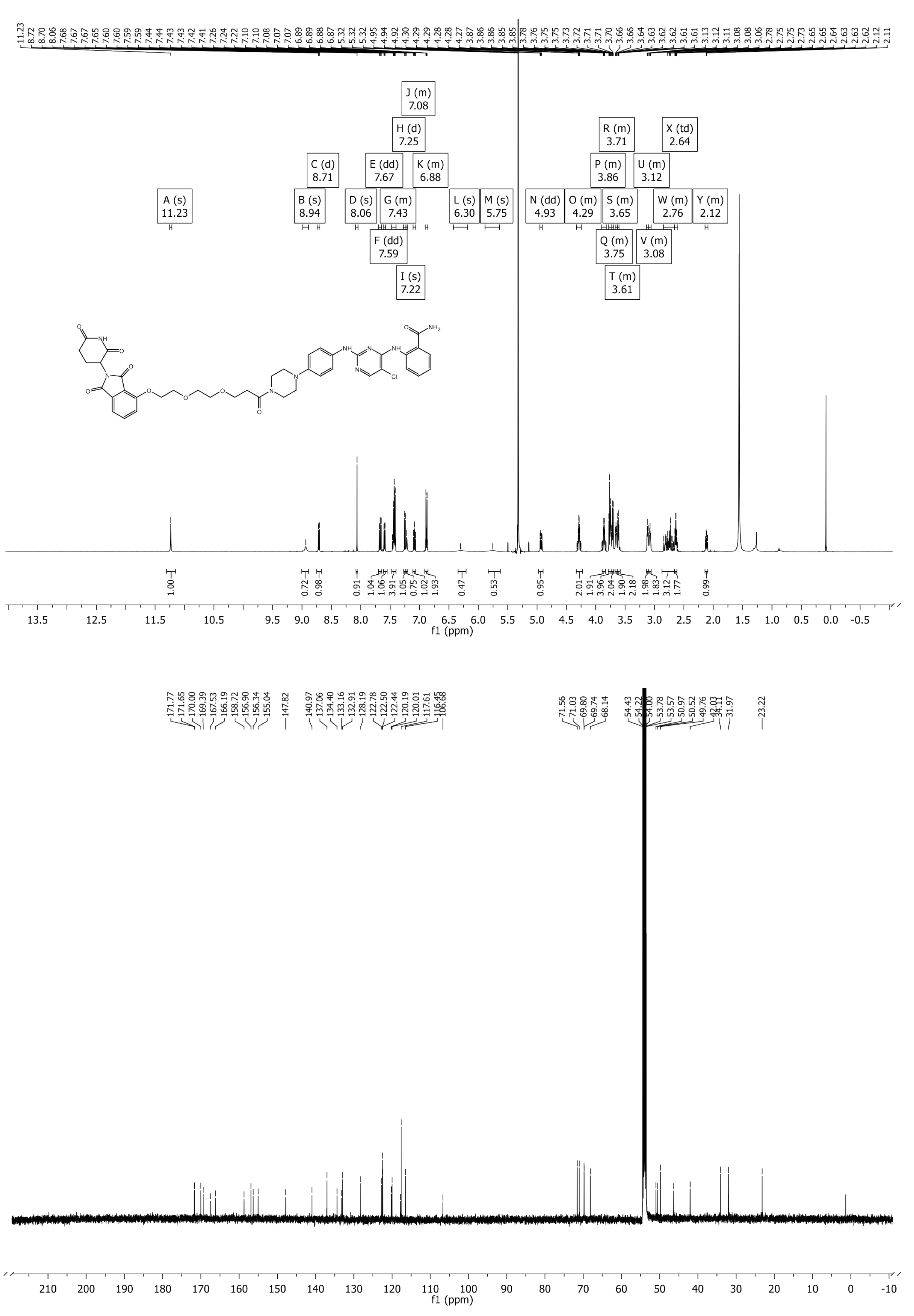

Figure S161: ^{1}H- (top) and ^{13}C-NMR (bottom) spectra (500 MHz and 126 MHz, 298 K, DCM-d_2) and chemical structure of compound **C-2a.**

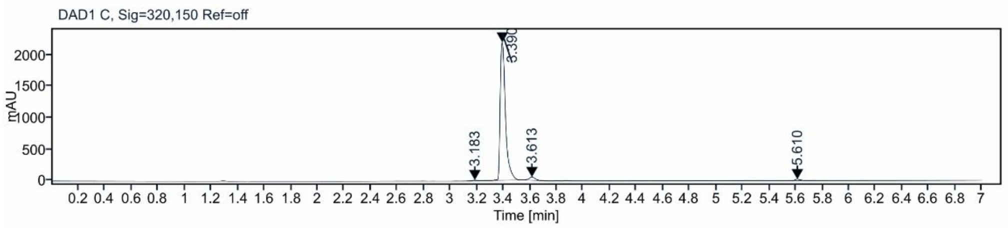

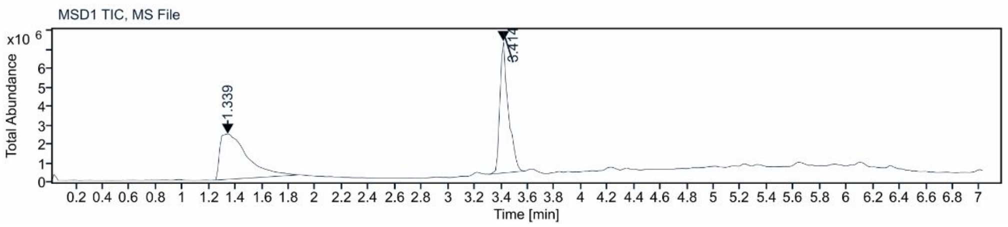

Signal Description DAD1 C, Sig=320,150 Ref=off

Sample Name	Name	RT	Width	Area	Area%	Height
jw-530		3.183	0.040	27.9179	0.44	11.4715
jw-530		3.390	0.041	6020.3848	95.82	2185.7866
jw-530		3.613	0.048	170.4435	2.71	51.3450
jw-530		5.610	0.046	64.1808	1.02	21.3371

Max Area% 95.821

UV Signal Purity>95% Pass

Figure S162: LC/MS spectra of purified compound **C-2a** at 320 nm wavelength.

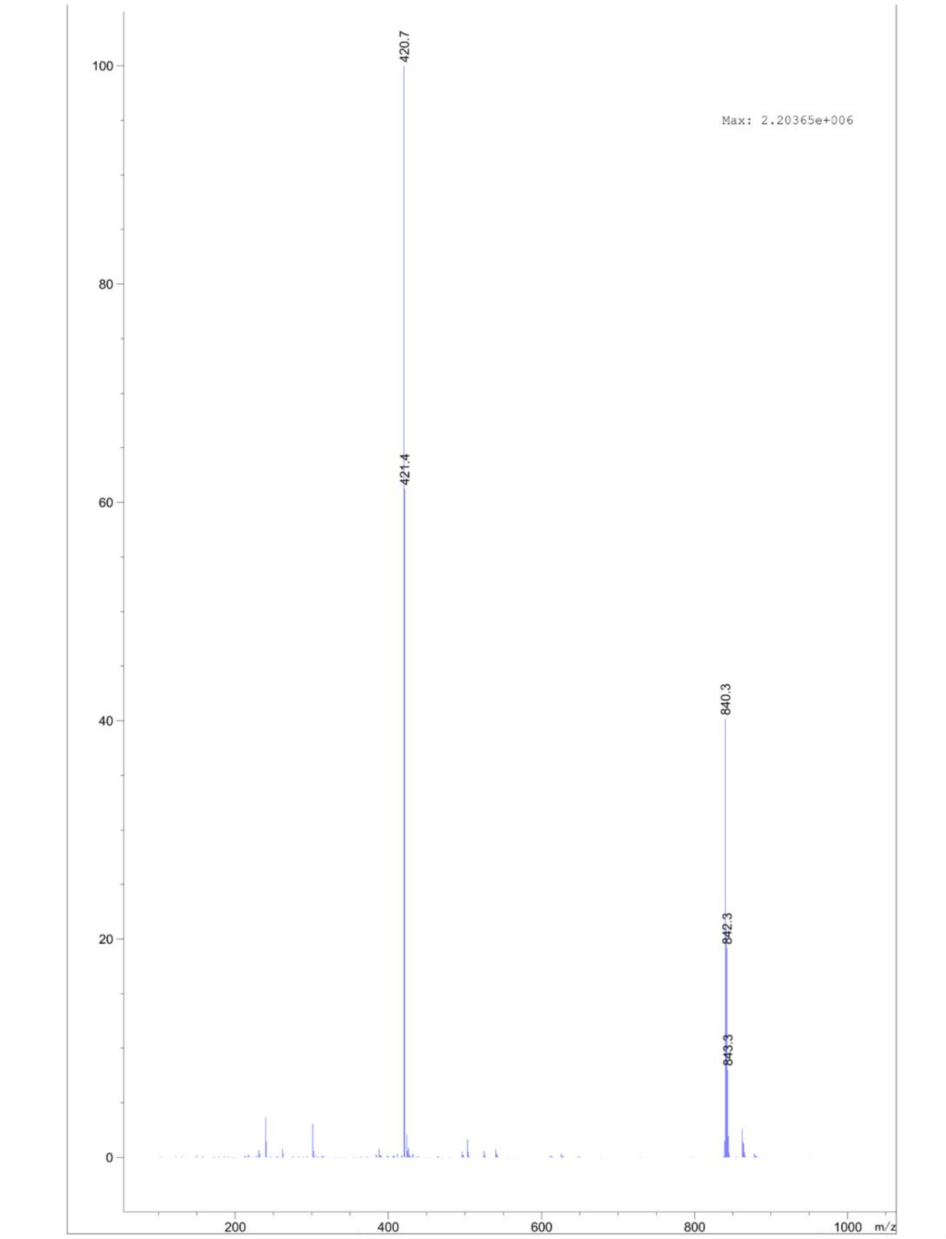

Figure S163: ESI-MS spectrum of compound **C-2a** with $[M+H]^{+}_{calc.}$ = 840.29 m/z.

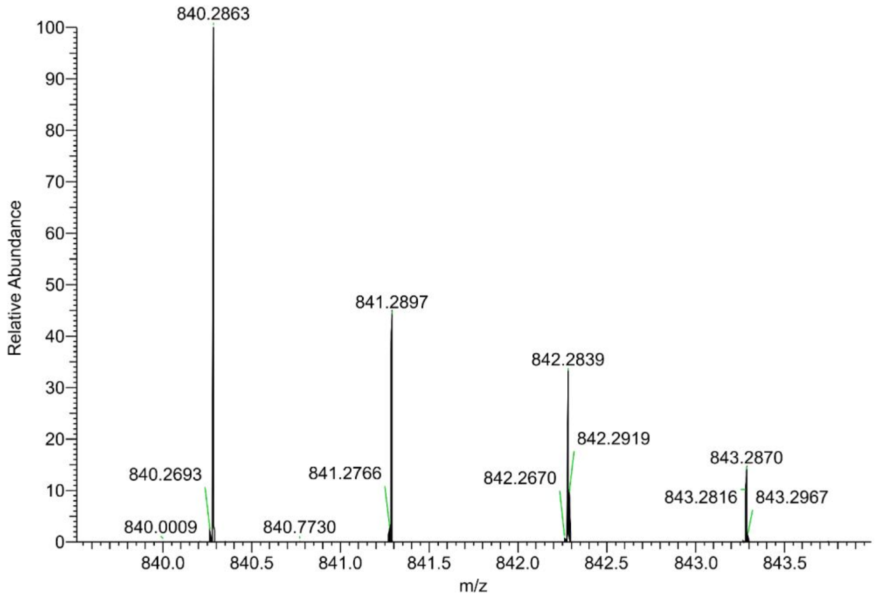

Figure S164: High-resolution mass spectrum of compound **C-2a** with $[M+H]^+_{calc.}$ = 840.2867 m/z

Tert-butyl 1-((2-(2,6-dioxopiperidin-3-yl)-1,3-dioxoisoindolin-4-yl)oxy)-3,6,9,12-tetraoxapentadecan-15-oate (S-51)

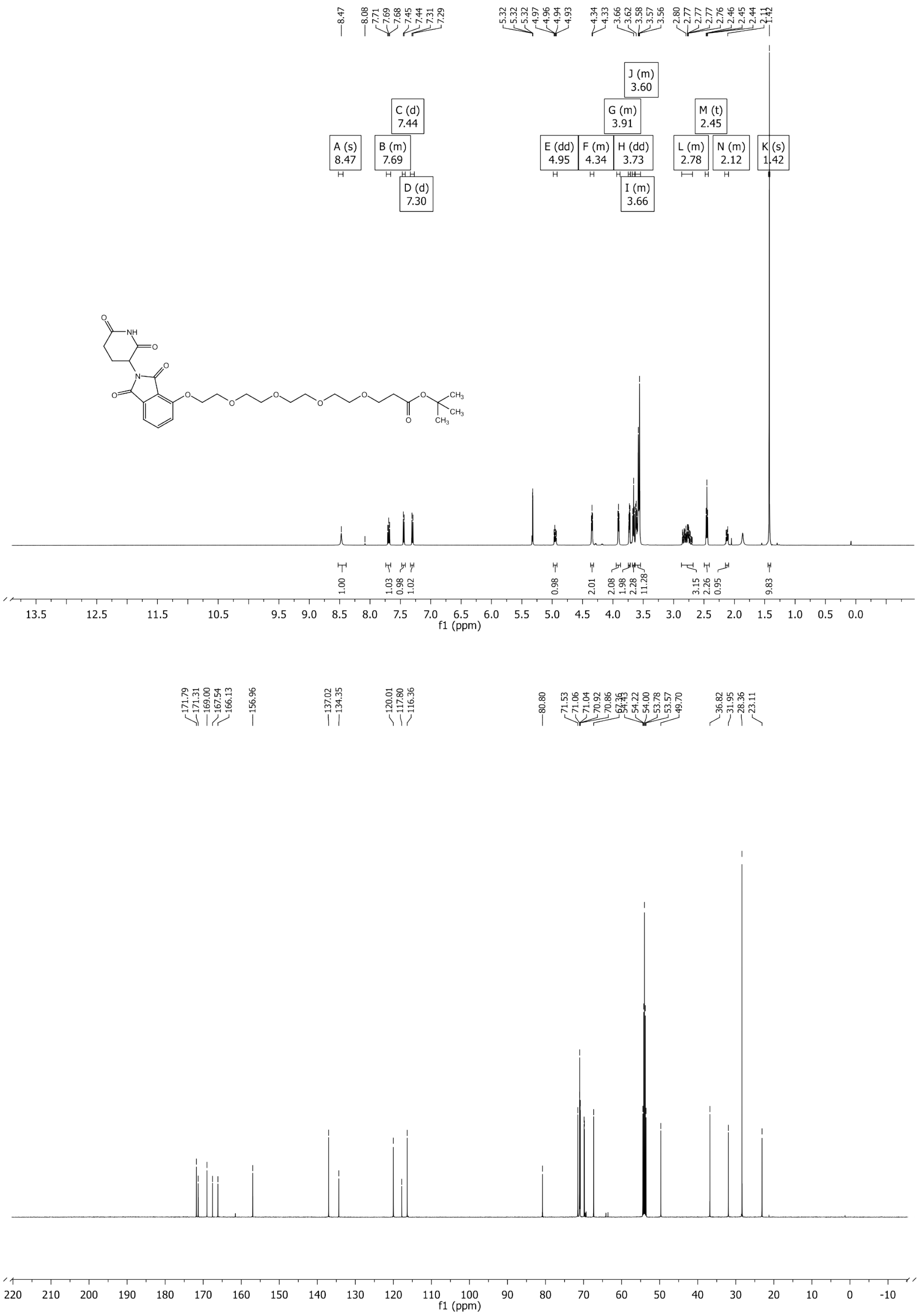

Figure S165: ^{1}H- (top) and ^{13}C-NMR (bottom) spectra (500 MHz and 126 MHz, 298 K, DCM-d_2) and chemical structure of compound **S-51.**

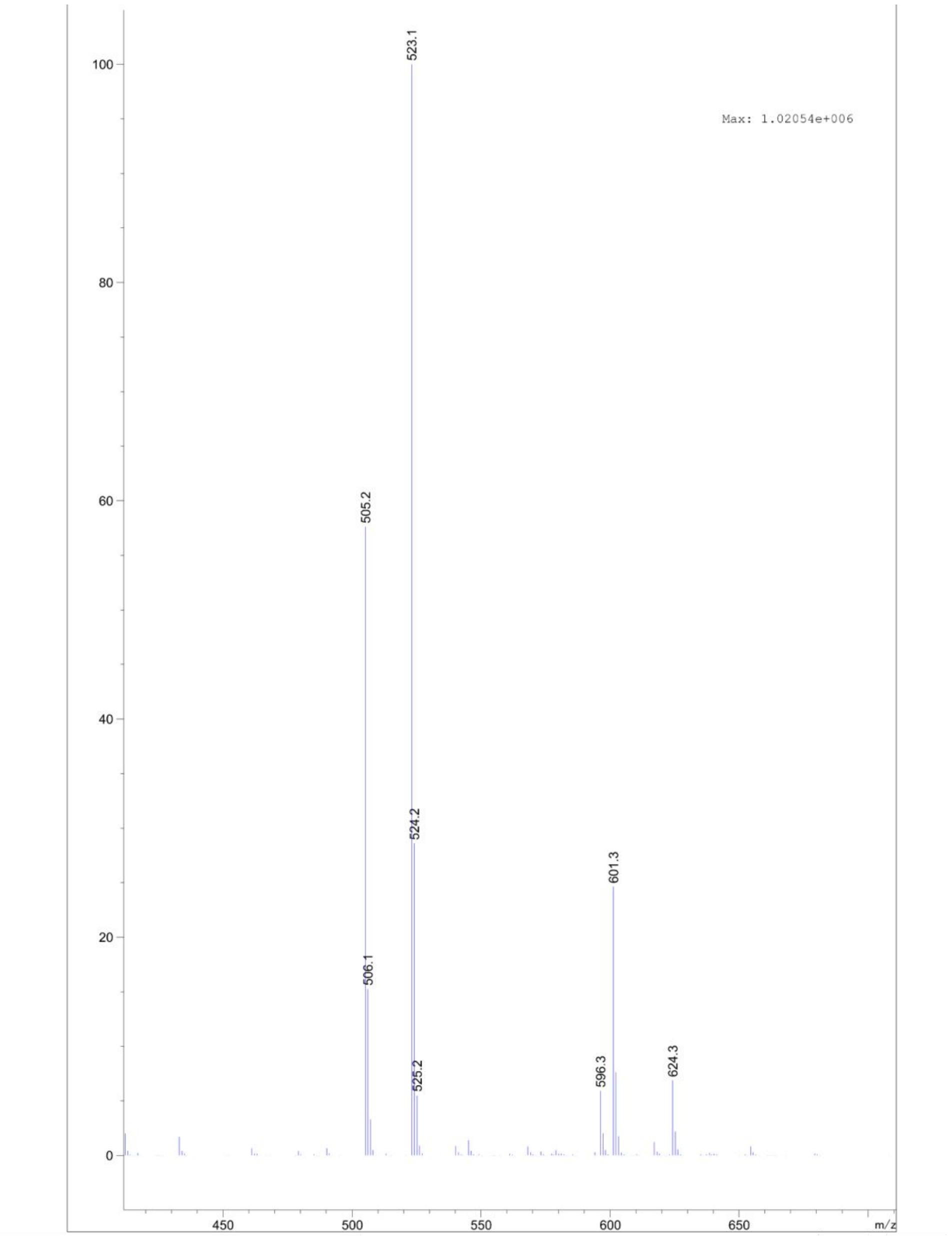

Figure S166: ESI-MS spectrum of compound **S-51** with $[M+H]^+_{calc.}$ = 601.2 m/z.

2-((5-Chloro-2-((4-(4-(1-((2-(2,6-dioxopiperidin-3-yl)-1,3-dioxoisoindolin-4-yl)oxy)-3,6,9,12-tetraoxapentadecan-15-oyl)piperazin-1-yl)phenyl)amino)pyrimidin-4-yl)amino)benzamide (C-2b)

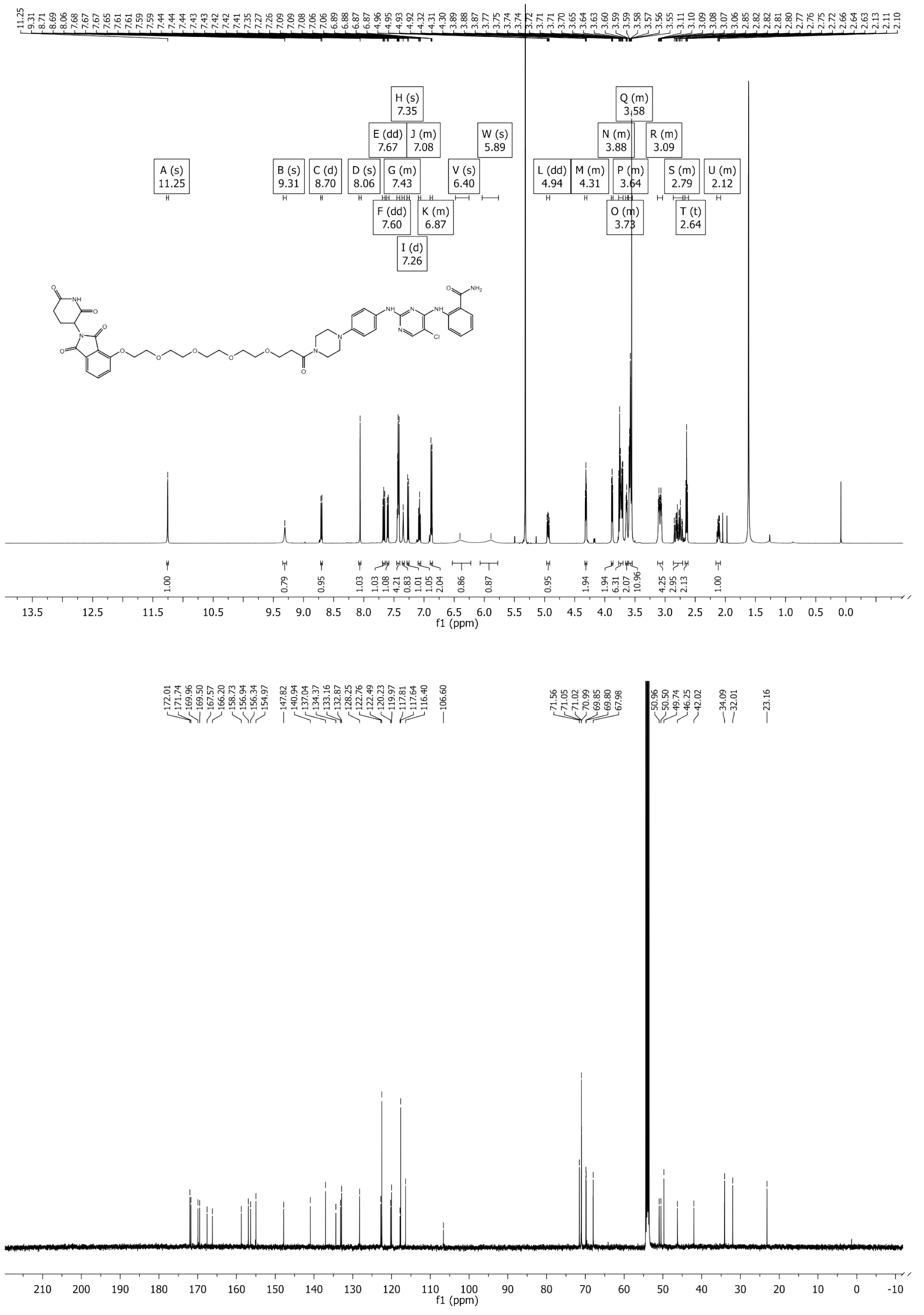

Figure S167: ^{1}H- (top) and ^{13}C-NMR (bottom) spectra (500 MHz and 126 MHz, 298 K, DCM-d_2) and chemical structure of compound **C-2b.**

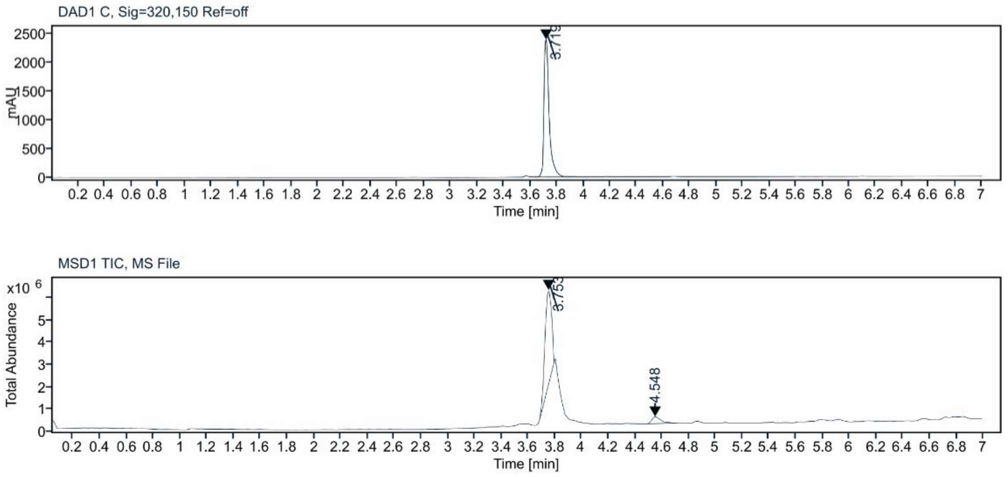

Signal Description DAD1 C, Sig=320,150 Ref=off

Sample Name	Name	RT	Width	Area	Area%	Height
jw-531		3.719	0.038	6522.6548	100.00	2397.5667

Max Area% 100.000

UV Signal Purity>95% Pass

Figure S168: LC/MS spectra of purified compound **C-2b** at 320 nm wavelength.

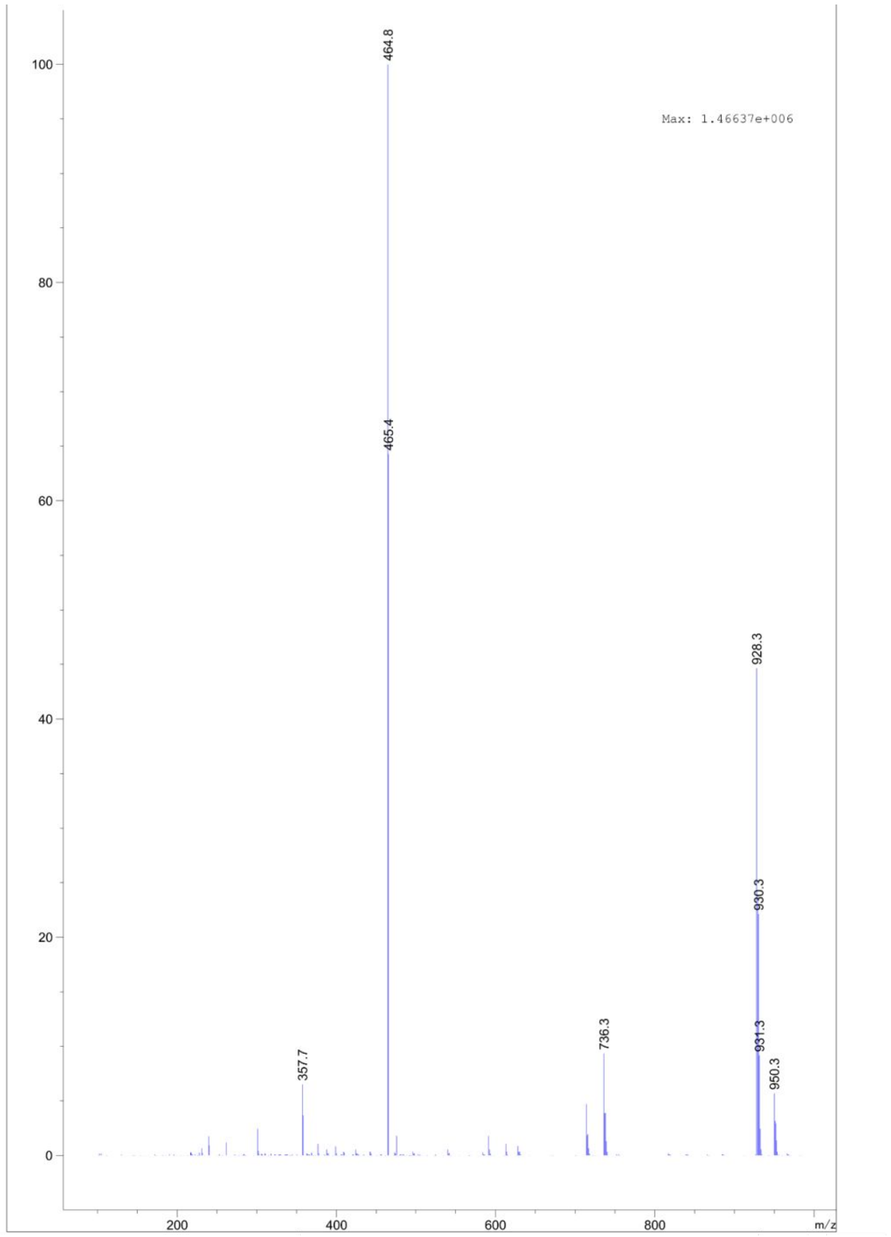

Figure S169: ESI-MS spectrum of compound **C-2b** with $[M+H]^{+}_{calc.}$ = 928.34 m/z.

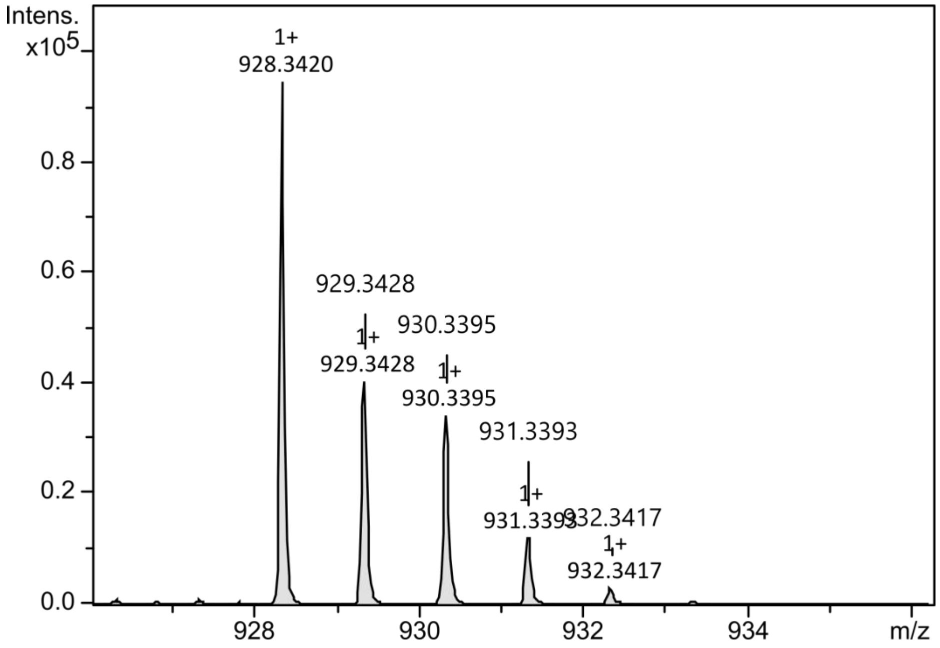

Figure S170: High-resolution mass spectrum of compound **C-2b** with $[M+H]^{+}_{calc.}$ = 928.3391 m/z

Tert-butyl (2-(2-(2-(2-(4-(4-((4-((2-carbamoylphenyl)amino)-5-chloropyrimidin-2-yl)amino)phenyl)piperazin-1-yl)ethoxy)ethoxy)ethoxy)ethyl)carbamate (S-52)

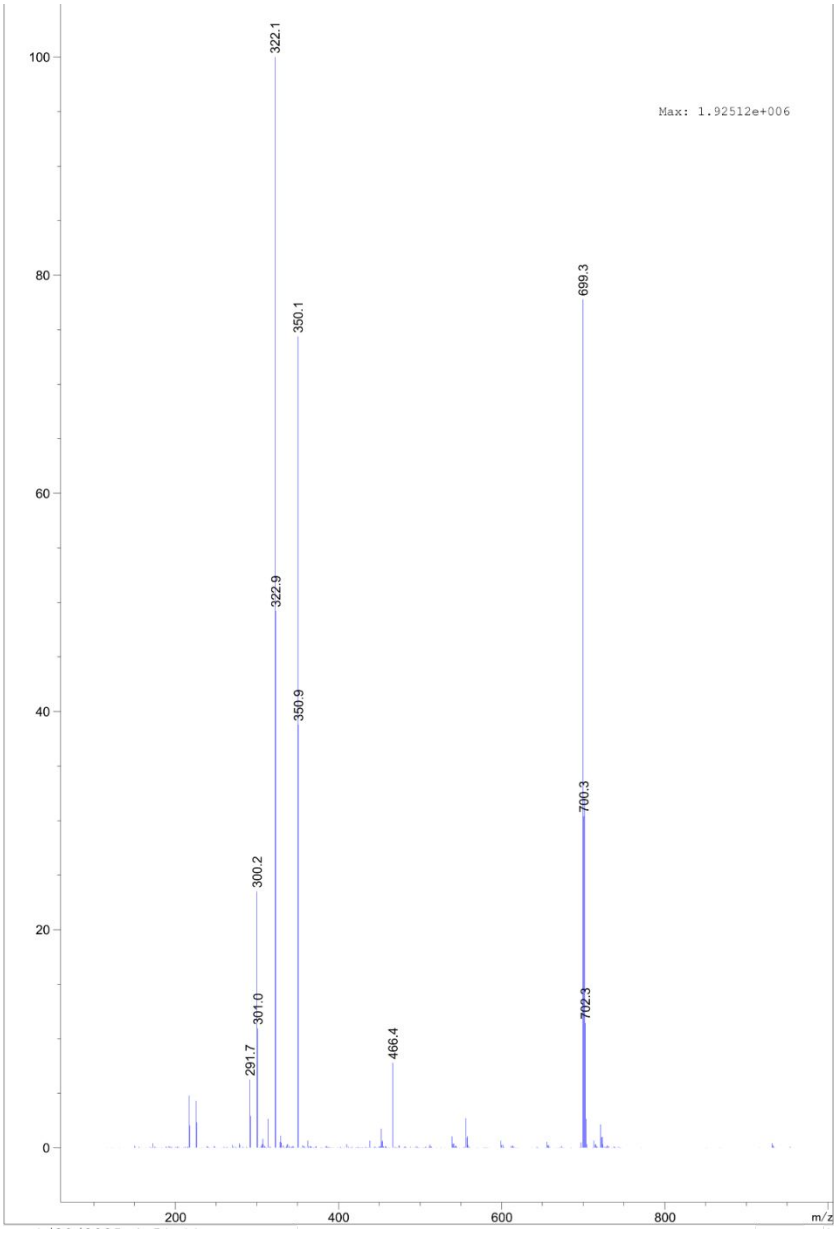

Figure S171: ESI-MS spectrum of compound **S-52** with $[M+H]^{+}_{calc.}$ = 699.3 m/z.

2-((5-Chloro-2-((4-(4-(1-((2-(2,6-dioxopiperidin-3-yl)-1,3-dioxoisoindolin-4-yl)oxy)-2-oxo-6,9,12-trioxa-3-azatetradecan-14-yl)piperazin-1-yl)phenyl)amino)pyrimidin-4-yl)amino)benzamide (C-2c)

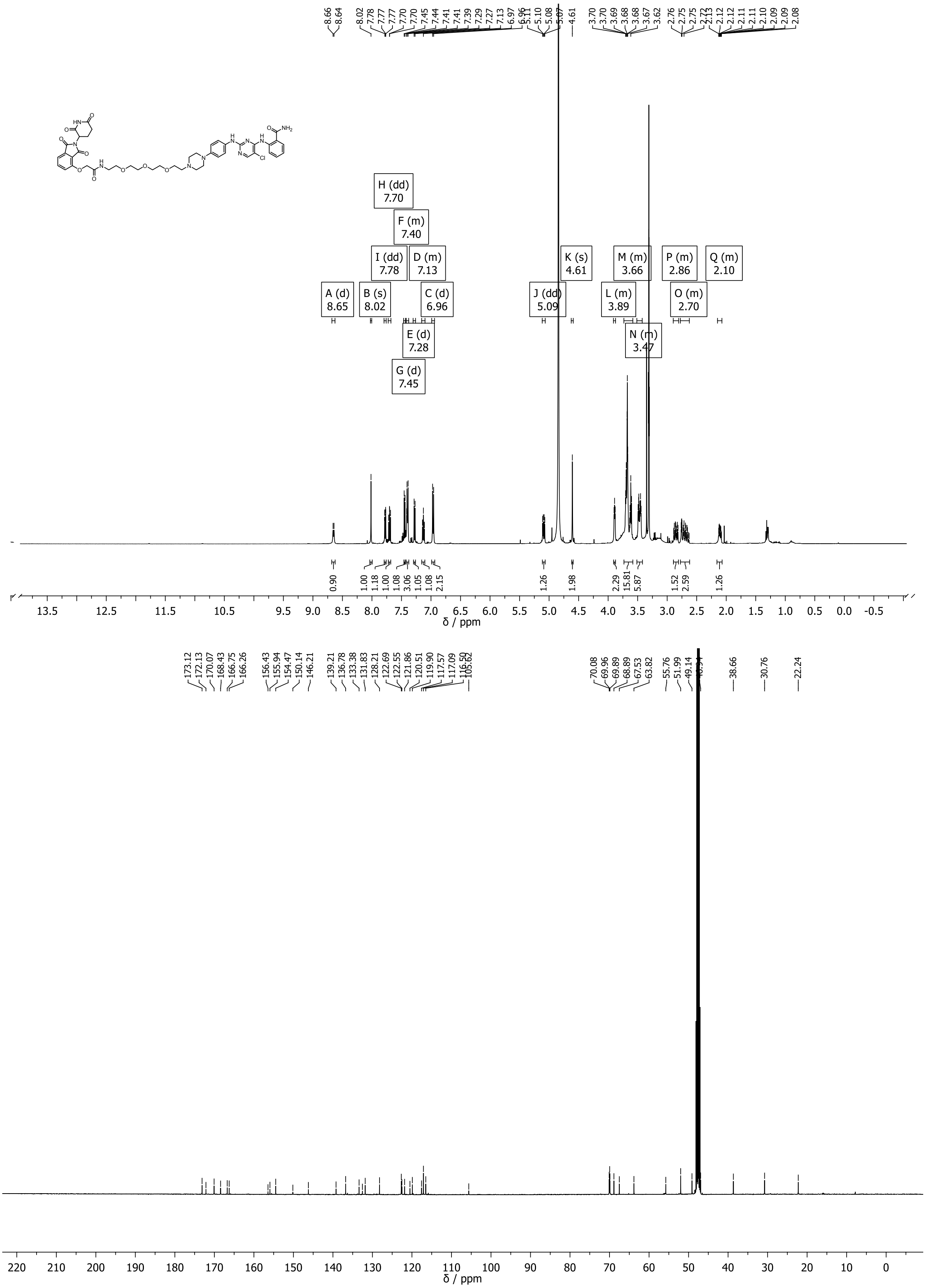

Figure S172: ^{1}H- (top) and ^{13}C-NMR (bottom) spectra (500 MHz and 126 MHz, 298 K, methanol-d_4) and chemical structure of compound **C-2c.**

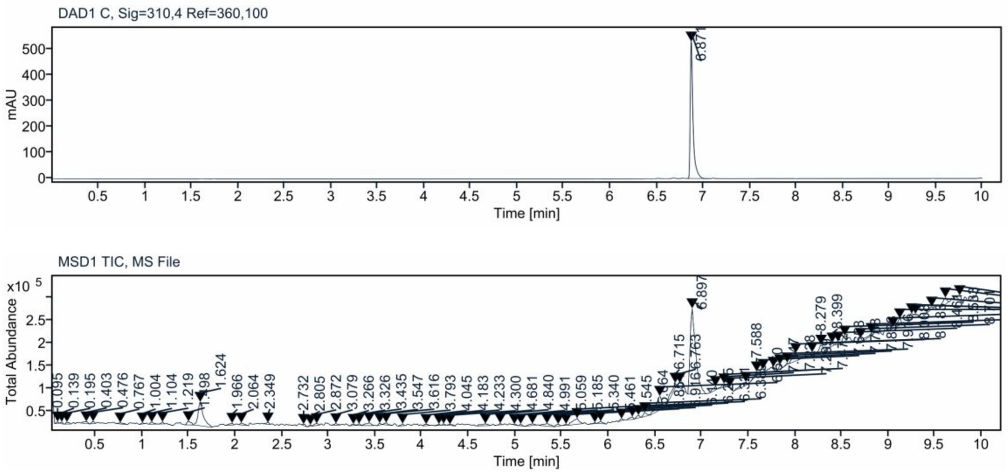

Signal Description	DAD1 C, Sig=310,4 Ref=360,100					
**Sample Name**	**Name**	**RT**	**Width**	**Area**	**Area%**	**Height**
VN2033		6.871	0.029	1111.8693	100.00	526.1561

Max Area% 100.000

UV Signal Purity>95% Pass

Figure S173: LC/MS spectra of purified compound **C-2c** at 310 nm wavelength.

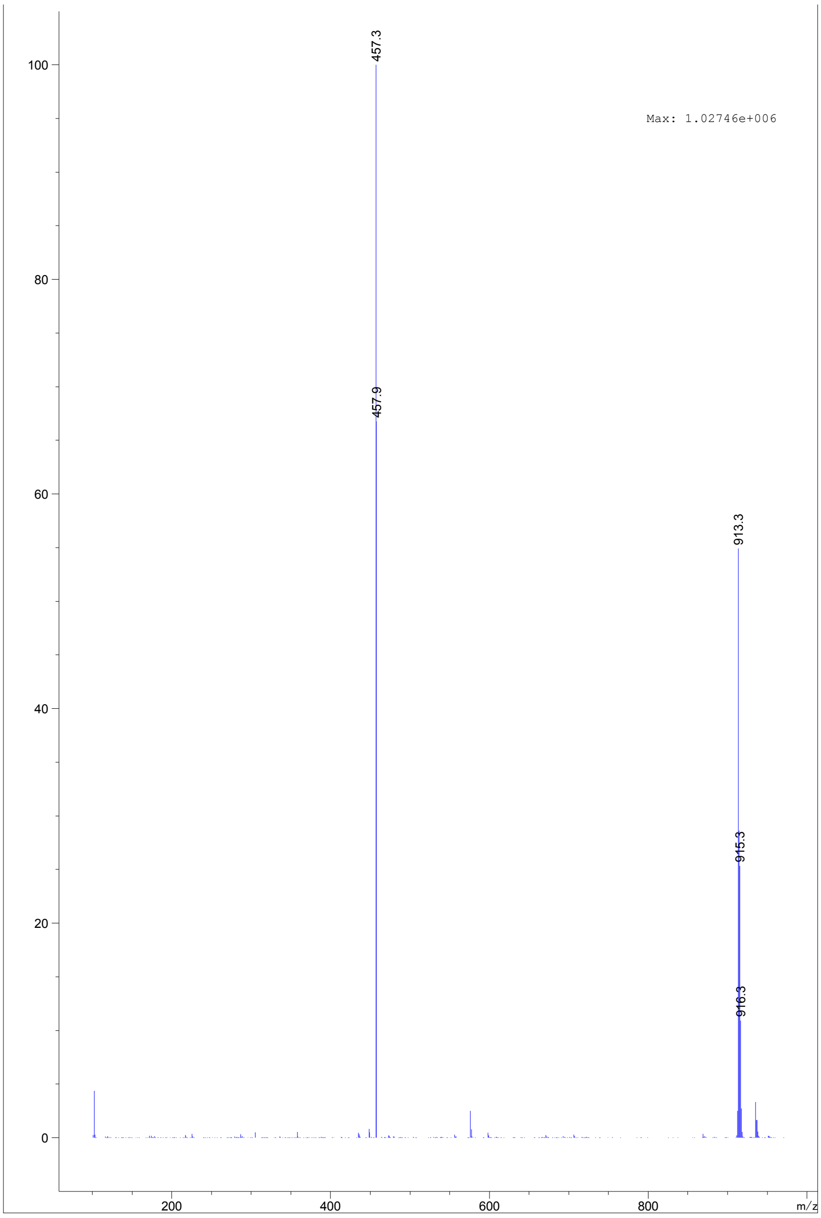

Figure S174: ESI-MS spectrum of compound **C-2c** with $[M+H]^+_{calc.}$ = 913.34 m/z.

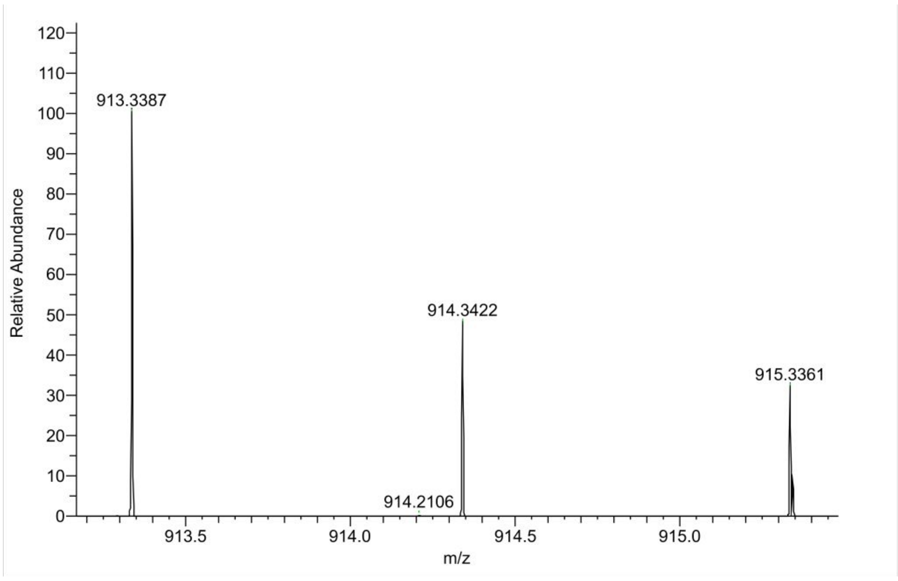

Figure S175: High-resolution mass spectrum of compound **C-2c** with $[M+H]^{+}_{calc.}$ = 913.3395 m/z.

***Tert*-butyl (7-(4-(4-((4-((2-carbamoylphenyl)amino)-5-chloropyrimidin-2-yl)amino)phenyl)piperazin-1-yl)heptyl)carbamate (S-53)**

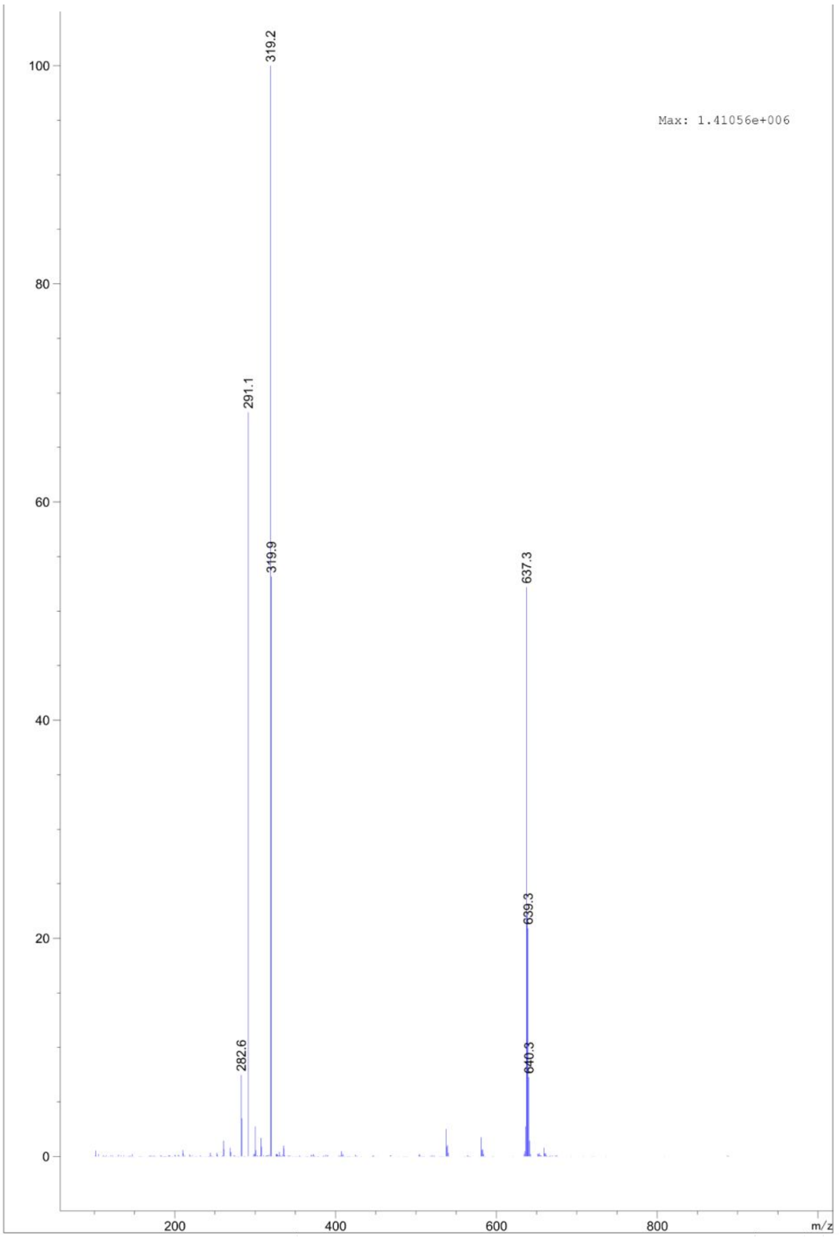

Figure S176: ESI-MS spectrum of compound **S-53** with $[M+H]^+_{calc.}$ = 637.3 m/z.

2-((5-Chloro-2-((4-(4-(7-(2-((2-(2,6-dioxopiperidin-3-yl)-1,3-dioxoisoindolin-4-yl)oxy)acetamido)heptyl)piperazin-1-yl)phenyl)amino)pyrimidin-4-yl)amino)benzamide (C-2d)

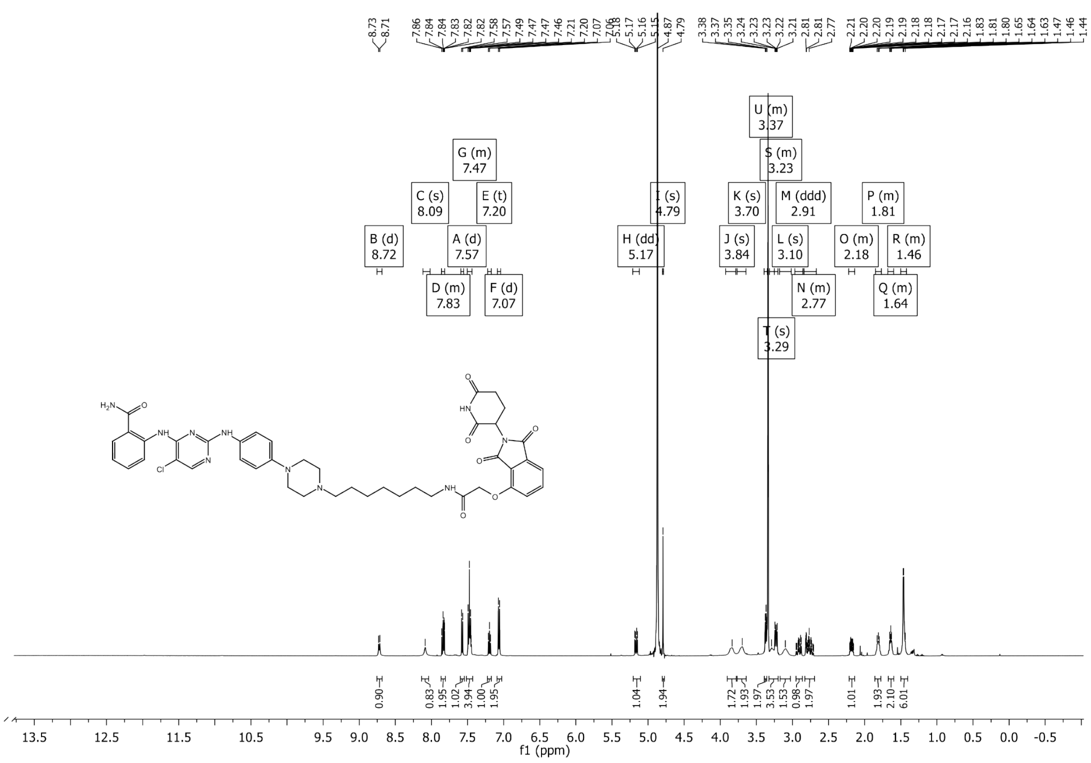

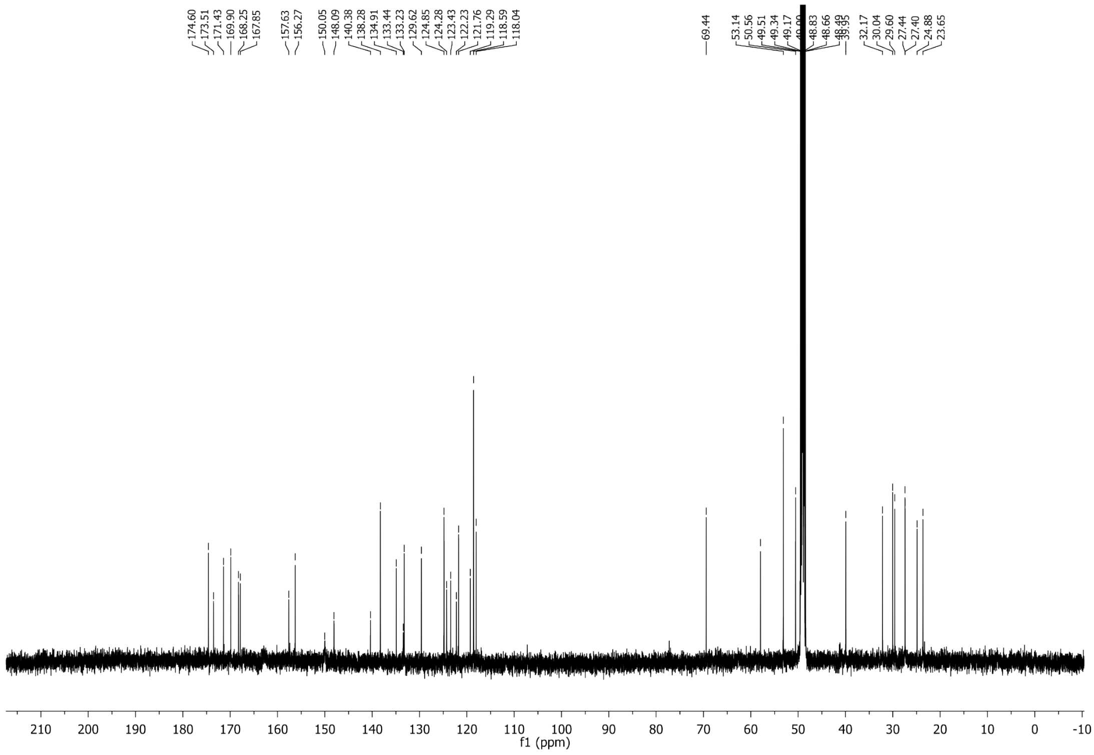

Figure S177: ^{1}H- (top) and ^{13}C-NMR (bottom) spectra (500 MHz and 126 MHz, 298 K, methanol-d_4) and chemical structure of compound **C-2d.**

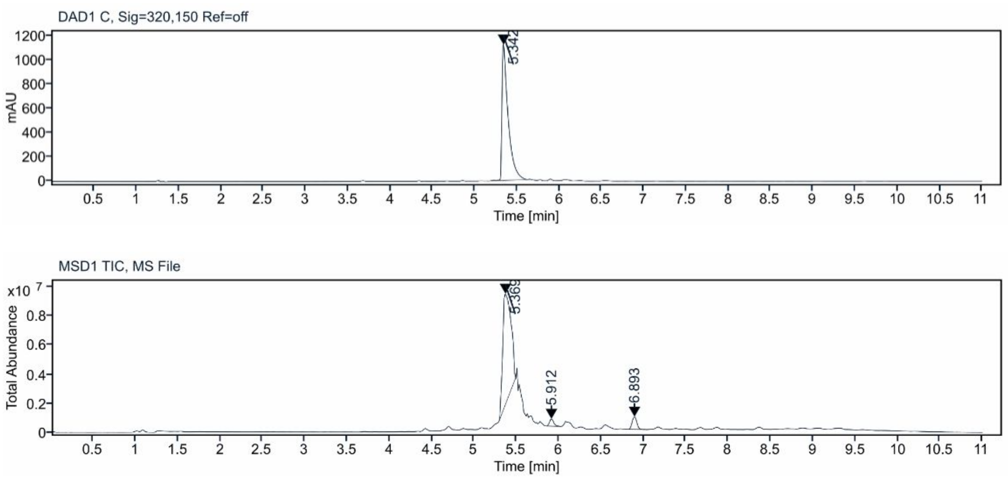

Signal Description DAD1 C, Sig=320,150 Ref=off

Sample Name	Name	RT	Width	Area	Area%	Height
jw-562_x		5.342	0.071	5651.7285	100.00	1114.5438

Max Area% 100.000

UV Signal Purity>95% Pass

Figure S178: LC/MS spectra of purified compound **C-2d** at 320 nm wavelength.

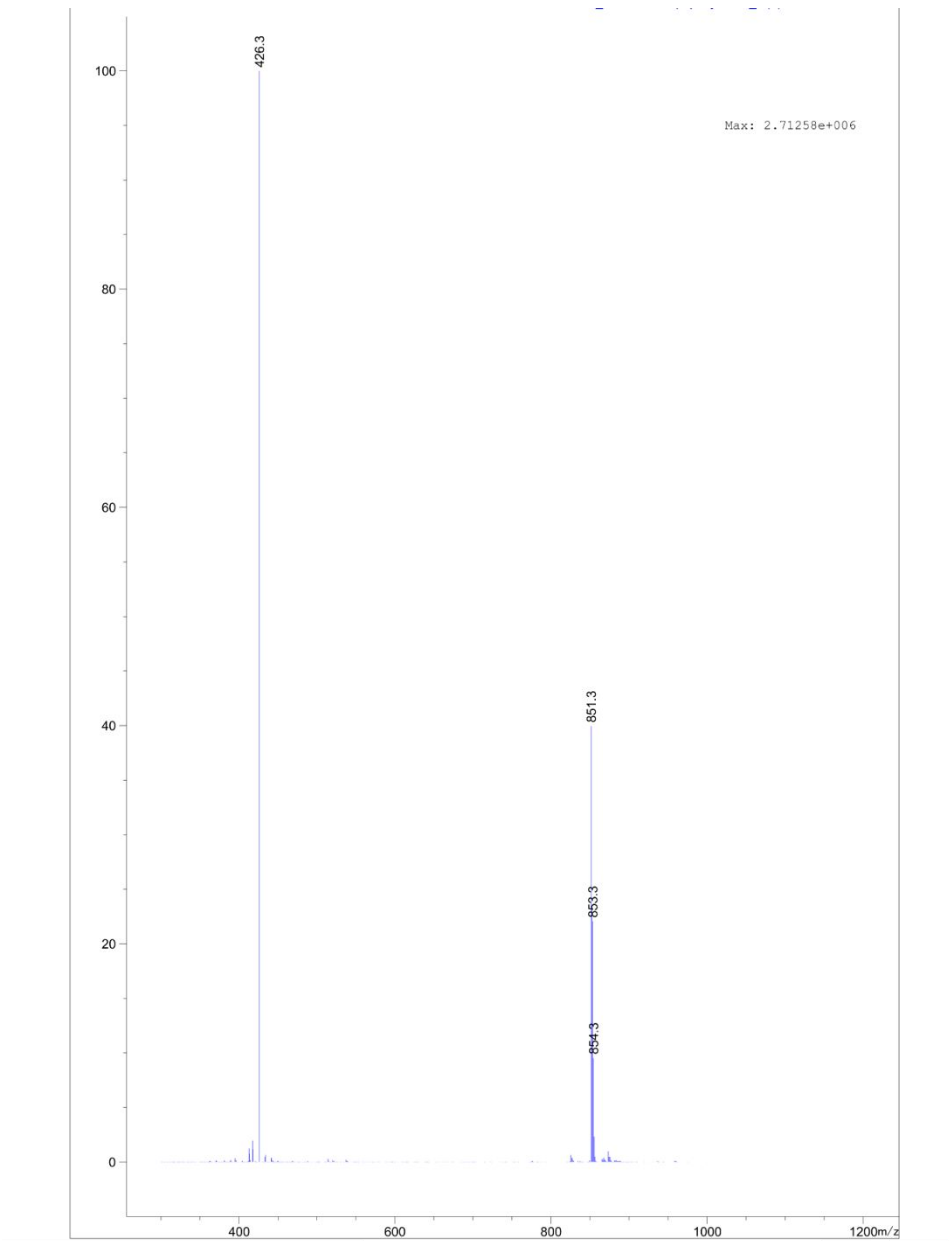

Figure S179: ESI-MS spectrum of compound **C-2d** with $[M+H]^{+}_{calc.}$ = 851.3 m/z.

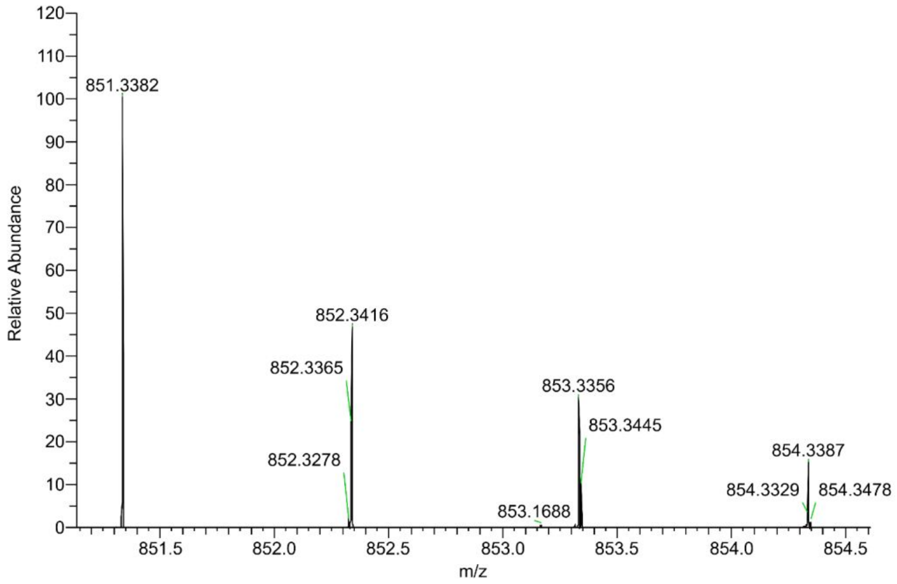

Figure S180**:** High-resolution mass spectrum of compound **C-2d** with $[M+H]^{+}_{calc.}$ = 851.3382 m/z.
